# The whole-cell pertussis vaccine imposes a broad effector B cell response in mouse heterologous prime-boost settings

**DOI:** 10.1172/jci.insight.157034

**Published:** 2022-11-08

**Authors:** Viviana Valeri, Akhésa Sochon, Clara Cousu, Pascal Chappert, Damiana Lecoeuche, Pascal Blanc, Jean-Claude Weill, Claude-Agnès Reynaud

**Affiliations:** 1 Institut Necker-Enfants Malades, INSERM U1151/CNRS UMR 8253, Université Paris Descartes, Sorbonne Paris Cité, Paris, France.; 2 Sanofi-Pasteur R&D, Marcy l’Etoile, France.

**Keywords:** Immunology, Vaccines, Adaptive immunity, Immunoglobulins, Memory

## Abstract

ÍSince the introduction of new generation pertussis vaccines, resurgence of pertussis has been observed in many developed countries. Former whole-cell pertussis (wP) vaccines are able to protect against disease and transmission but have been replaced in several industrialized countries because of their reactogenicity and adverse effects. Current acellular pertussis (aP) vaccines, made of purified proteins of *Bordetella pertussis*, are efficient at preventing disease but fail to induce long-term protection from infection. While the systemic and mucosal T cell immunity induced by the 2 types of vaccines has been well described, much less is known concerning B cell responses. Taking advantage of an inducible activation-induced cytidine deaminase fate-mapping mouse model, we compared effector and memory B cells induced by the 2 classes of vaccines and showed that a stronger and broader memory B cell and plasma cell response was achieved by a wP prime. We also observed that homologous or heterologous vaccine combinations that include at least 1 wP administration, even as a booster dose, were sufficient to induce this broad effector response, thus highlighting its dominant imprint on the B cell profile. Finally, we describe the settlement of memory B cell populations in the lung following subcutaneous wP prime vaccination.

## Graphical abstract

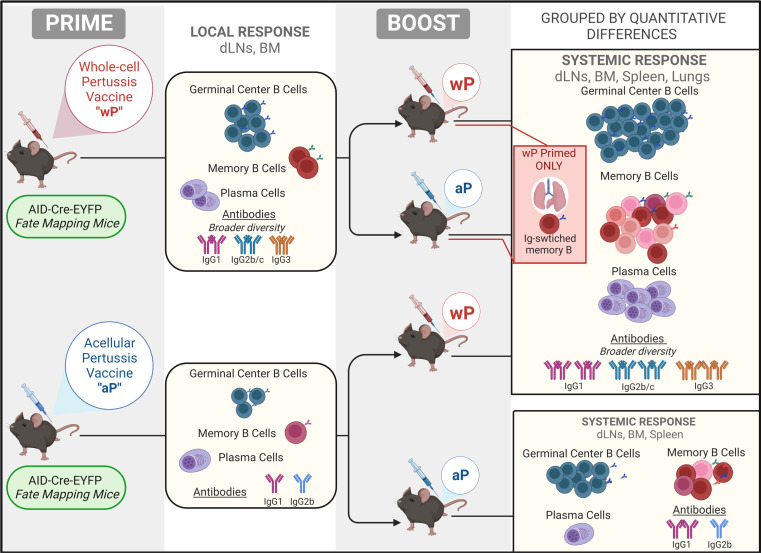

## Introduction

Whooping cough or pertussis is a vaccine-preventable severe respiratory disease caused by the Gram-negative bacteria *Bordetella pertussis* (Bp) that can affect people of all ages. The introduction of whole-cell pertussis (wP) vaccines by the mid-1940s, administrated in combination with the diphtheria and tetanus vaccines, allowed the control of infections and reduction of new cases. However, the attention was soon put on cases of severe reactions induced by this formulation. Safety concerns and decrease in vaccine coverage motivated its substitution in the late 1990s in most high-income countries with a second-generation vaccine: the acellular pertussis (aP) formulation. This is composed of 1 to 5 purified proteins of Bp: pertussis toxoid (PT), pertactin (PRN), filamentous hemagglutinin (FHA), and fimbriae 2 and 3 (Fim2,3) (1). Disease surveillance, in recent decades, showed global resurgence of pertussis incidence despite generally high vaccine coverage (2, 3). In countries that have switched from the wP to the aP vaccine, it has been hypothesized that this phenomenon may be related to the faster waning of the protection after priming doses of aP in children (4), but other factors, such as a suboptimal protection due to the limited number of antigens of the aP formulation, the spread and selection of pertactin-deficient Bp strains — a key target for opsonizing antibodies (Abs) — and improved surveillance methods and awareness, are also debated (5, 6). Both human studies and animal models show that natural infection and wP vaccination produce similar types of immunity. For instance, they polarize CD4^+^ T helper 1 (Th1) and Th17 cells, which produce IFN-γ and IL-17 as well as a population of CD4^+^CD69^+^ resident memory T cells in the lung (7, 8) that provides protection against mucosal infection, in a manner dependent on IFN-γ and IL-17 (9–12). Intranasal and pulmonary Bp immunization (but not aP vaccines) are also able to induce memory B cells in the lungs (12–14) whereas memory B cells in mucosal tissues after both aP and wP parenteral vaccination, to our knowledge, have never been studied. Bp infection and wP vaccination mostly induce Abs of the subclasses IgG2 in the mouse (IgG1 and IgG3 in humans) and IgA that are good opsonizers of the bacteria and neutralize the toxin (15). Conversely, aP vaccines induce a mixed Th1/Th2 immunity, and the majority of induced mouse IgG1 Abs (IgG4 Abs in humans) are good neutralizers for the toxin but have a reduced opsonization potency against the bacteria. Hence, while protection against the disease is conferred by the aP vaccine, colonization and passive transmission of Bp are not efficiently prevented (8, 15).

In contrast to the acknowledged role played by vaccine-induced Abs in disease protection (13, 16), there has been surprisingly little in-depth description of the memory and effector B cell responses induced by pertussis vaccines. The decay of serum Abs has been hypothesized as one of the major causes of resurgence of pertussis disease in adolescents (3); however, apart from circulating Abs, other functions covered by B cells in the context of Bp protection have been proposed. In 2000, indeed, it was shown that optimal protective immunity to Bp requires B cells, since μMT B cell–lacking mice were only partially protected and a rapid clearance of bacteria in B cell–reconstituted mice was not directly associated with a rapid increase in Ab production (17). Accordingly, after respiratory challenge with Bp in vaccinated BALB/c mice, bacteria protection was also effective in the absence of circulating Abs, but in the presence of splenic anti-Bp Ab-secreting cells (ASCs), significantly higher in wP-vaccinated mice (18). A role of memory B cells in absence of circulating Abs was also suggested in human studies: children that received a diphtheria, tetanus wP prime and a booster vaccination with diphtheria, tetanus aP vaccine at the age of 4 had stable levels of anti-PT, FHA, and PRN memory B cells and low/no correlation was determined with anti-PT, FHA, and PRN IgG Abs in the serum 5 years later (19). However, such a protective role provided by memory B cells was not confirmed in another mouse model of pertussis vaccination in which the authors showed poor maintenance and reactivation of the memory B cell pool (20). A more recent publication showed instead efficient memory B cell reactivation upon aP boost by comparing groups of people of different ages, from different countries, and injected with different prime vaccine types; moreover, the authors reported in this work a positive correlation between memory B cells and serum IgG concentrations (21).

It is widely accepted that the type of prime vaccine, irrespective of the nature of booster doses, determines the immunological pattern, especially as concerns T cell polarization in both infants and animal models (22–26). However, to our knowledge, no evidence exists so far as to whether a subsequent wP vaccination, or Bp exposure, can modify a Th2-biased immune profile associated with the aP priming. In particular, the impact of different prime:boost schedules, such as homologous or heterologous vaccine combinations, which may produce variation in the extent, quality, and persistence of B cell responses, has not been thoroughly investigated.

To explore these issues, we used a cell lineage tracing mouse model that allows a time-controlled and irreversible marking of B cells when they engage in germinal center (GC) reactions and express the *Aicda* gene (encoding the activation-induced cytidine deaminase protein, AID) (27). We report here the specific ability of the wP vaccine to produce a broader Ab isotypic response and larger populations of GC and local and systemic memory B cells, even when combined with the aP vaccine prime.

## Results

### A broader Ab response is induced by the wP prime vaccination.

**Figure 1:**
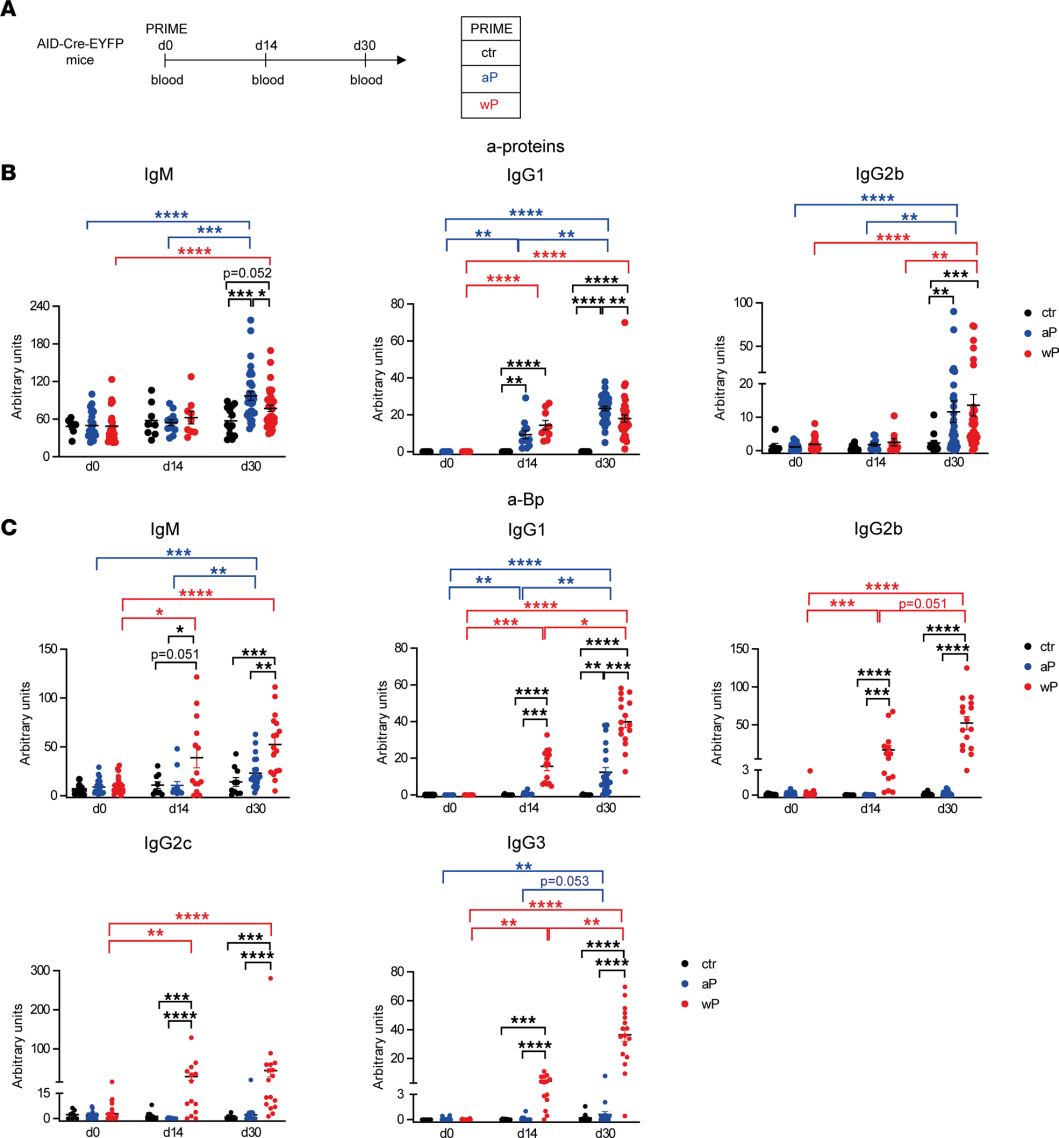
A broader Ab response is induced by the wP prime vaccination. (**A**) Blood was collected at day 0, day 14, and day 30 from AID-Cre-EYFP mice s.c. injected with aP or wP vaccines or with alum (ctr, control mice). IgM, IgG1, and IgG2b serum Ab titers against pooled proteins of the aP vaccine (PT, PRN, FHA, Fim2,3) (**B**) and IgM, IgG1, IgG2b, IgG2c, and IgG3 serum Ab titers against sonicated Bp (**C**) were detected by ELISA from serum of vaccinated and control mice. Ab titers are arbitrary values and each point in the graphs represents individual mouse data. At least 2 independent experiments were performed for each analysis. Means (±SEM) are shown. Kruskal-Wallis analysis with uncorrected Dunn’s test was performed to compare the different conditions at each time point and the different time points between the same conditions. *P < 0.05, **P < 0.01, ***P < 0.001, ****P < 0.0001.

We first compared in our AID-Cre-ERT2 × ROSA26-loxP-EYFP mouse system (27) (called AID-Cre-EYFP from now on) serum Ab levels induced by modified aP versus wP vaccine formulations that do not contain diphtheria and tetanus antigens; control mice were injected with adjuvant only (aluminum hydroxide, alum) (Figure 1A). We assessed by ELISA the level of serum Abs directed against the pool of proteins that composes the aP formulation: PT, PRN, FHA, and Fim2,3. Positive signals were obtained for the IgM, IgG1, and IgG2b isotypes. Anti-protein IgG1 Abs were detected over control mice at day 14 in both aP- and wP-vaccinated mice while IgM and IgG2 protein-specific Abs were raised later, with titers becoming significantly higher than the basal level only at day 30. At day 30, slightly higher values of protein-specific IgM and IgG1 Abs were detected in aP- versus wP-primed mice (Figure 1B). Data from single-protein ELISAs suggested that this difference may be contributed by the low/undetectable anti-PT IgG1 and IgG2b levels in wP-primed animals, which reflects the low PT protein content of this vaccine (28, 29) (Supplemental Figure 1; supplemental material available online with this article; https://doi.org/10.1172/jci.insight.157034DS1).

The wP vaccine includes a large number of bacterial antigens beyond the 4 proteins of the aP formulation. Thus, we tested the presence of serum Abs directed against the whole sonicated Bp. Higher anti-Bp IgM and IgG1 titers were detected in sera from wP-primed mice compared with the aP ones at both day 14 and day 30 (Figure 1C). Moreover, the production of IgG2b, IgG2c, and IgG3 Bp-specific Abs was uniquely observed in the wP priming context (Figure 1C). Collectively, these data indicate that the wP vaccine, unlike the aP formulation, is less efficient at inducing anti-PT Abs during the primary response but generates higher Bp-specific Ab levels with broader isotypic profiles.

### The wP prime leads to stronger and broader B cell and plasma cell responses.

**Figure 2:**
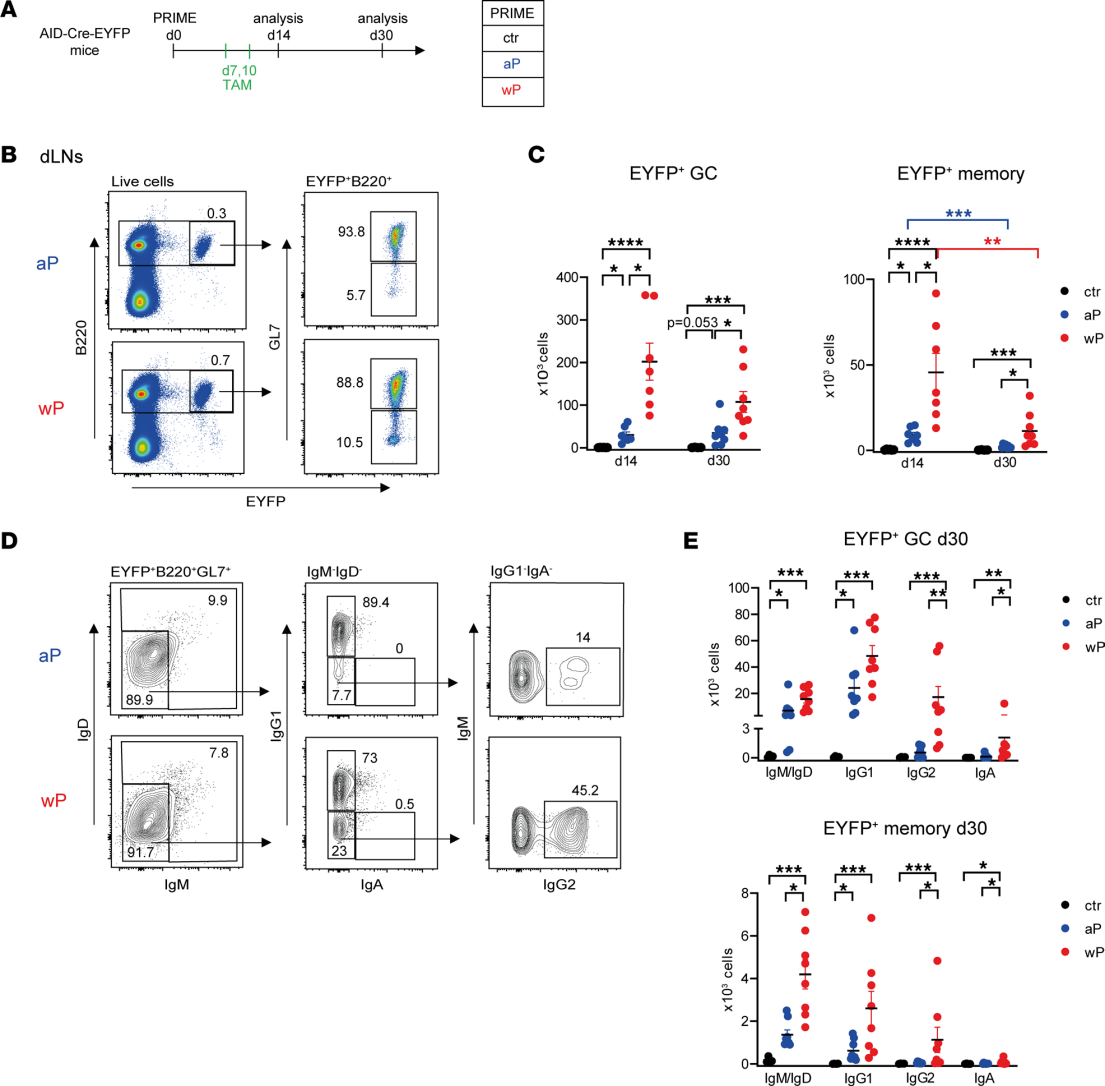
The wP prime leads to stronger GC and memory B cell responses in dLNs. (**A**) AID-Cre-EYFP mice primed either with aP or wP vaccines or controls injected with alum (ctr) received 2 doses of tamoxifen at days 7 and 10 after prime vaccination. Mice were analyzed at days 14 and 30. (**B**) B220^+^EYFP^+^ live cells from dLNs of mice primed with aP and wP vaccines were distinguished into GC (GL7^+^) and memory (GL7^–^) B cells, by flow cytometry. (**C**) Total EYFP^+^ GC and EYFP^+^ memory B cell counts in the 2 dLNs are shown in the graphs. (**D**) A representative flow cytometry profile of heavy chain isotype distribution among EYFP^+^GL7^+^ B cells is shown for the aP and wP conditions at day 30 after prime. (**E**) IgM/IgD, IgG1, IgG2, and IgA distribution in the EYFP^+^ GC and memory subsets from mice analyzed at day 30 after prime are shown in the plots. Each point represents an individual mouse analyzed at day 14 or day 30 after prime vaccination. At least 2 independent experiments were performed for each analysis. Means (±SEM) are shown. Kruskal-Wallis analysis with uncorrected Dunn’s test was performed to compare the different conditions at each time point or each Ab isotype. *P < 0.05, **P < 0.01, ***P < 0.001, ****P < 0.0001.

The AID-Cre-EYFP mouse model allows irreversible and time-controlled labeling of B cells engaged in GCs and expressing AID upon tamoxifen feeding (27). AID-Cre-EYFP mice primed as described in Figure 1A received tamoxifen at days 7 and 10, time points that cover the initiation of the GC B cell reaction, and were analyzed at days 14 and 30 (Figure 2A). We first analyzed the induction of EYFP^+^ GC and memory B cells, respectively GL7^+^ and GL7^–^, in the inguinal draining lymph nodes (dLNs) by flow cytometry (Figure 2B). Our fate-mapping model, which contains an EYFP reporter devoid of CMV immediate enhancer/β-actin that increases its accessibility, allows the labeling of only a fraction of GC-activated B cells, but these cells are limited to the GC reaction. Extrafollicular CD38^+^GL7^+^ activated B cells, enriched in IgD^+^ cells and not uniformly expressing the PNA GC marker, were indeed mostly excluded from the EYFP labeling (Supplemental Figure 2) (30). Upon prime, the EYFP^+^ population was mainly composed by GC B cells at day 14 and day 30, with GC and memory B cells present in higher numbers in wP- than aP-primed mice at the 2 time points. Moreover, wP-induced GC B cell responses developed with faster kinetics, peaking at day 14 and decreasing at day 30, while the aP-induced ones appeared steady between day 14 and day 30 (Figure 2C). Although less abundant compared with GC B cells, the EYFP^+^GL7^–^ memory B cell subset did not contain B220^+^CD138^+^CD21^lo^ plasmablasts but was conversely enriched in CCR6^+^CD93^–^ cells (surface markers associated with memory B cells and plasma cells, PCs, respectively) (Supplemental Figure 3). At the level of the spleen, no primary GC B cell response was identified in aP- or wP-vaccinated mice (Supplemental Figure 4). We determined the isotype profile of EYFP^+^ GC and memory B cells generated by the vaccination at day 30 (representative analysis is shown for EYFP^+^ GC B cells in Figure 2D). IgM^+^/IgD^+^ and IgG1^+^ EYFP^+^ GC B cell numbers were only marginally higher in the wP compared with the aP group, while IgG2^+^, and to a lower extent IgA^+^, EYFP^+^ GC B cells were significantly increased in wP mice, while being similar to control values for aP mice (Figure 2E). For the memory compartment, EYFP^+^ memory B cells were more numerous for all isotypes in wP compared with aP mice, but IgM^+^/IgD^+^ memory B cells, instead of IgG1^+^, constituted the most abundant subset (Figure 2E).

**Figure 3:**
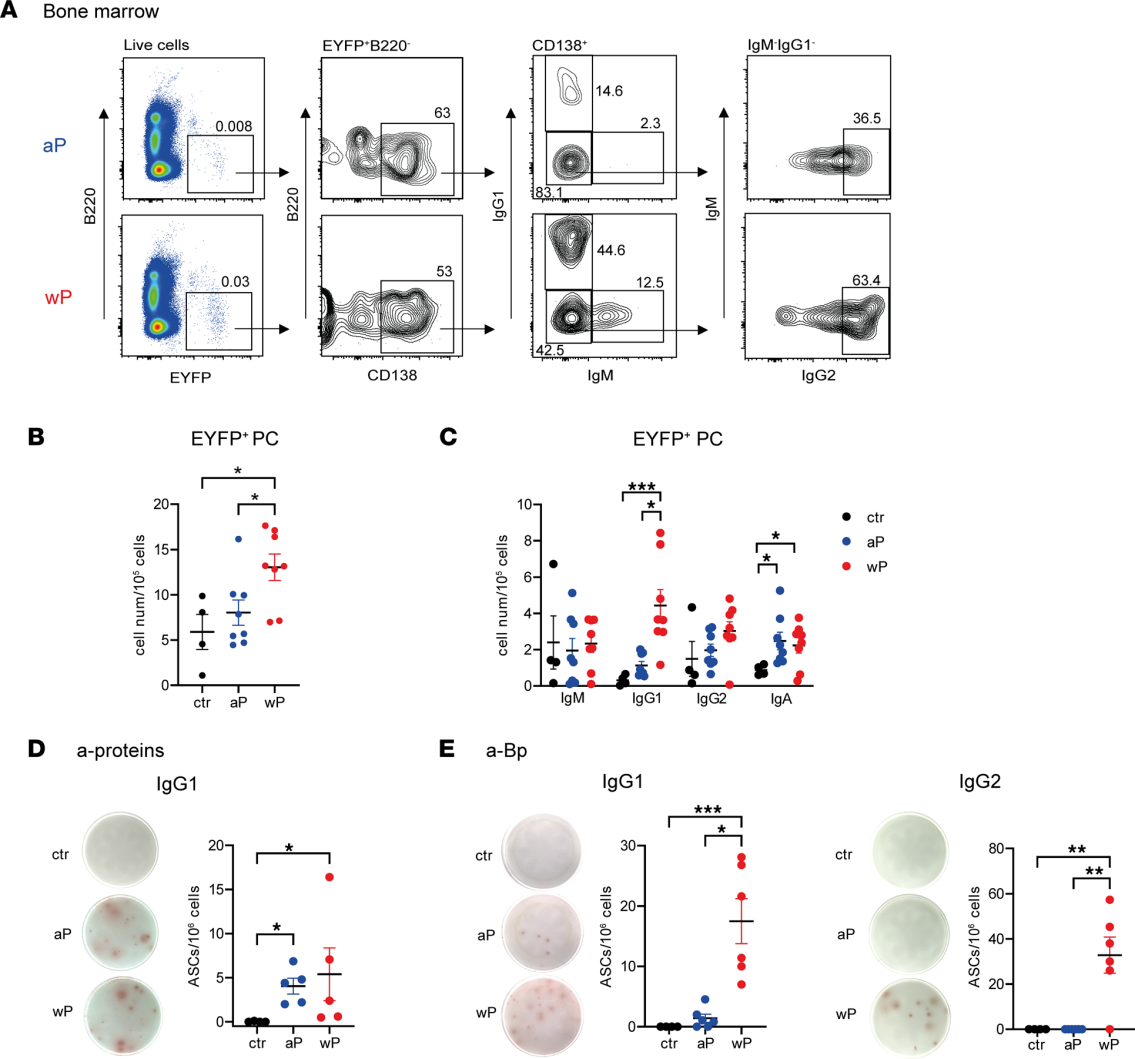
The wP prime induces a broader PC population in BM. AID-Cre-EYFP mice primed with aP or wP vaccines or controls injected with alum (ctr) received 2 doses of tamoxifen at days 7 and 10 after prime vaccination. BM cells were collected 30 days after prime vaccination. (**A**) Representative flow cytometry profile of intracellular staining for EYFP^+^ PCs in BM. B220^–^EYFP^+^ live cells were gated into CD138^+^ cells before determination of IgM, IgG1, and IgG2 distribution among PCs (a similar independent analysis was performed for IgA at the place of IgG2, not shown here). Total EYFP^+^ PC cell numbers (**B**) or EYFP^+^ PC isotype distribution (**C**) are represented in the plots. Numbers of IgG1^+^ ASCs against pooled proteins (PT, PRN, FHA, Fim2,3) (**D**) and numbers of IgG1^+^ and IgG2^+^ ASCs against sonicated Bp (**E**) were determined by ELISPOT from total BM cells. Representative spot images for each condition are shown at the left of each panel. Each point in the graphs represents an individual mouse. At least 2 independent experiments were performed for each analysis. Means (±SEM) are shown. Kruskal-Wallis analysis with uncorrected Dunn’s test was performed to compare the different conditions. *P < 0.05, **P < 0.01, ***P < 0.001.

During a primary response, GC-engaged B cells differentiate into long-lived PCs that home to the bone marrow (BM). Thus, we compared the extent of the EYFP^+^B220^–^CD138^+^ population and isotype distribution among BM PCs by flow cytometry (Figure 3A). We observed that the wP prime elicited a higher frequency of EYFP^+^ PCs than the aP condition, which, in contrast, remained similar to controls (Figure 3B). Isotype profile analysis of EYFP^+^ PCs in BM showed marked differences mainly for IgG1^+^EYFP^+^ PCs between the 2 vaccine settings (Figure 3C). To further determine the antigen specificity of the whole BM PC pool, ELISPOT assays were performed on total BM cells and revealed on plates coated either with pooled aP proteins or with sonicated Bp. In line with serum Ab analysis, similar amounts of IgG1 anti-protein ASCs were observed in both conditions (Figure 3D), whereas IgG1 anti-Bp ASC numbers were significantly higher in wP-primed mice and IgG2 anti-Bp ASCs exclusively induced by the wP immunization (Figure 3E).

### wP:wP prime:boost vaccination is highly efficient at producing secondary B cell responses, including GCs, PCs, and systemic memory B cells, shortly after the boost.

We addressed the impact of homologous or heterologous prime:boost vaccination schedules on B cell recall responses by boosting mice with the 4 different vaccine combinations at day 30. The 3 tamoxifen gavages given during the prime enabled the follow-up of persisting GCs and of memory B cells reactivated in the boost conditions (Supplemental Figure 5A). Comparable levels of EYFP^+^ GC B cells were found in dLNs in all the groups that contained at least 1 aP injection while the wP:wP combination had increased numbers of secondary EYFP^+^ GC B cells (Supplemental Figure 5B, left). A majority of IgG1^+^EYFP^+^ GC B cells was identified in each condition, followed by IgM^+^/IgD^+^ cells and IgG2^+^ GC B cells (Supplemental Figure 5B, right).

**Figure 4:**
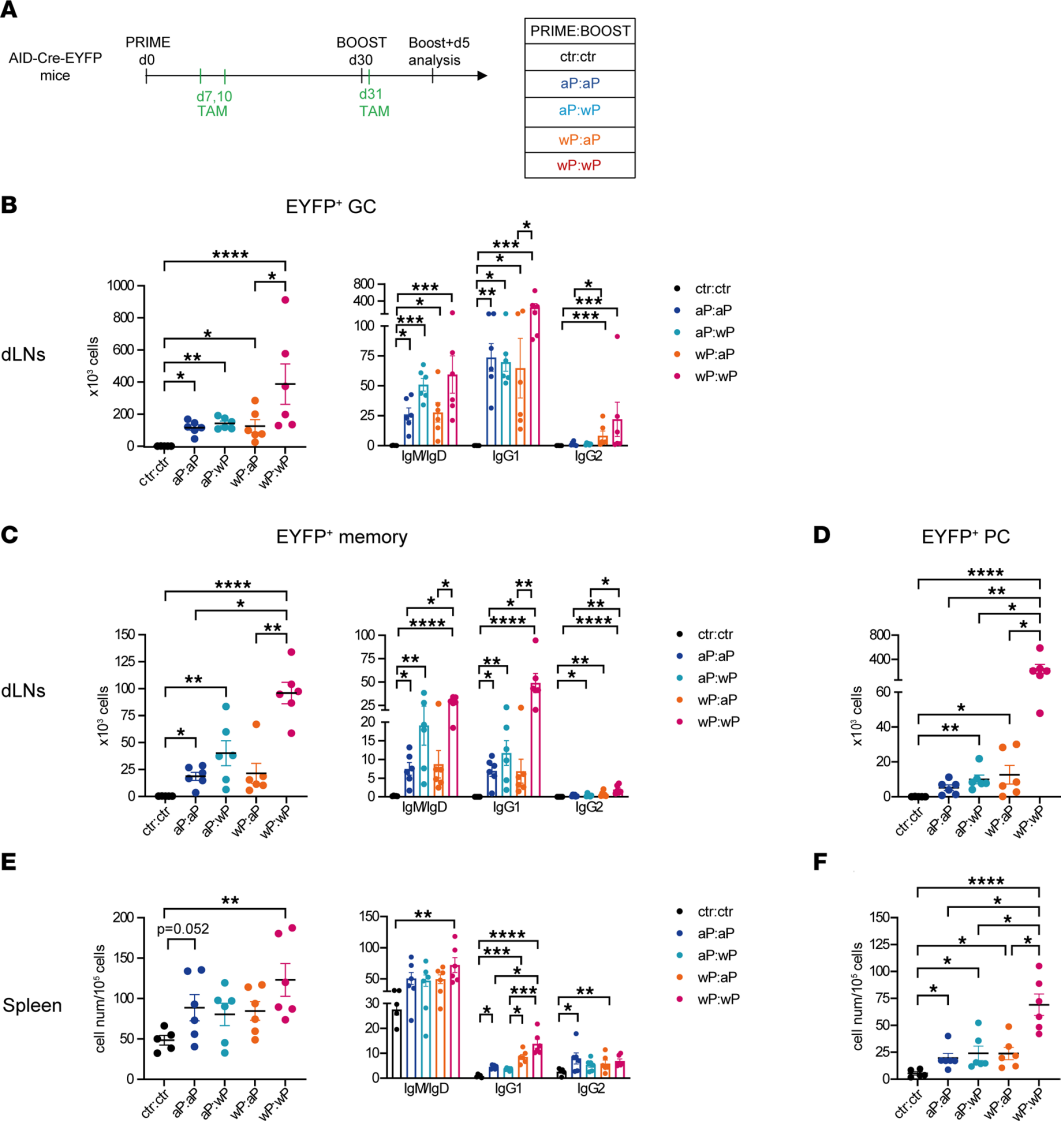
wP:wP prime:boost vaccination is highly efficient at producing secondary B cell responses, including GCs, PCs, and systemic memory B cells, shortly after the boost. (**A**) AID-Cre-EYFP mice were primed and boosted (day 30) with homologous and heterologous combinations of the aP and wP vaccines or injected with alum (ctr). Three doses of tamoxifen were administrated at days 7, 10, and 31. Mice were analyzed at day 35. Total cell numbers and isotype distribution of EYFP^+^ GC (**B**) or EYFP^+^ memory (**C**) B cells were determined by flow cytometry in dLNs. (**E**) Cell numbers (relative to 10^5^ splenocytes) and isotype distribution of EYFP^+^ memory B cells were determined by flow cytometry in the spleen. EYFP^+^ PCs were assessed by flow cytometry in dLNs (**D**) or spleen (**F**), and cell counts were reported similarly in the graphs. Each point in the plots represents an individual mouse. At least 2 independent experiments were performed for each analysis. Means (±SEM) are shown. Kruskal-Wallis analysis with uncorrected Dunn’s test was performed to compare the different conditions. *P < 0.05, **P < 0.01, ***P < 0.001, ****P < 0.0001.

Another strategy of tamoxifen feeding was used to identify both GC-engaged B cells during priming as well as naive B cells recruited upon boost: to this end, mice received 2 doses of tamoxifen during the primary reaction and 1 dose after the boost, during which newly recruited naive B cells became labeled (Figure 4A). Similar differences were identified between all groups as compared with the ones observed in the previous tamoxifen-labeling conditions, with the main difference that the IgM^+^/IgD^+^ component within the EYFP^+^ GC population increased as a result of naive B cells’ engagement into secondary GCs (Figure 4B). The ratio between GC EYFP^+^ B cells and total GC B cells was calculated for both tamoxifen feeding strategies and showed similar higher values in all vaccine settings upon after-boost tamoxifen administration, suggesting a naive B cell recruitment proportional to the memory response (Supplemental Figure 5C). The approximate 2-fold difference in total EYFP^+^ GC cell numbers between these 3 tamoxifen injection schedules furthermore suggests that the recruitment of naive and memory B cells within GC is of similar amplitude. Due to its higher labeling efficiency, this strategy was selected for further studies of memory persistence.

Among dLN EYFP^+^ memory B cells, analogous differences between the groups were also identified, with the wP:wP EYFP^+^ memory population being significantly higher than the 2 aP-boosted groups and 10 times higher than after the wP prime analyzed at day 30 (Figure 4C, compared with Figure 2C). The wP boost, even on an aP prime, appeared more efficient in the amplification of the memory B cell pool. Isotype distribution in the memory EYFP^+^ population showed the presence of IgM^+^/IgD^+^ and IgG1^+^ B cells within each group (Figure 4C). PC differentiation was usually observed upon boost as a result of memory B cell differentiation. In dLNs, PCs were detected in all conditions, but their numbers were significantly higher compared with control mice when at least 1 dose of wP vaccine was administrated, with a massive increase in wP:wP mice (Figure 4D; see Figure 3B). IgG1^+^EYFP^+^ PCs predominated over other isotypes in all groups (Supplemental Figure 6A).

The primary B cell response generates memory B cells that can further migrate toward other lymphoid sites. Accordingly, we analyzed the presence of EYFP^+^ memory B cells and PCs at the level of the spleen. Interestingly, 5 days after the boost, memory B cells were detected in the spleens of vaccinated mice and were significantly increased in the wP:wP condition (Figure 4E). The great majority of spleen memory EYFP^+^ B cells were IgM^+^/IgD^+^. However, significant levels of EYFP^+^ IgG1^+^ memory B cells were detected and were higher in the 2 wP-primed conditions, whereas similar levels of IgG2^+^ EYFP^+^ memory B cells were identified in all conditions (Figure 4E). Splenic EYFP^+^ PCs were also observed in all conditions but were again more abundant in the wP:wP group (Figure 4F and Supplemental Figure 6B). To sum up, 5 days after boost, the wP:wP vaccine combination was able to generate high secondary B cell responses, whereas a more modest increase in the other vaccine combinations was monitored at this time point.

### Persistence of GC B cells and of local and systemic memory B cells are favored by the wP vaccine.

**Figure 5:**
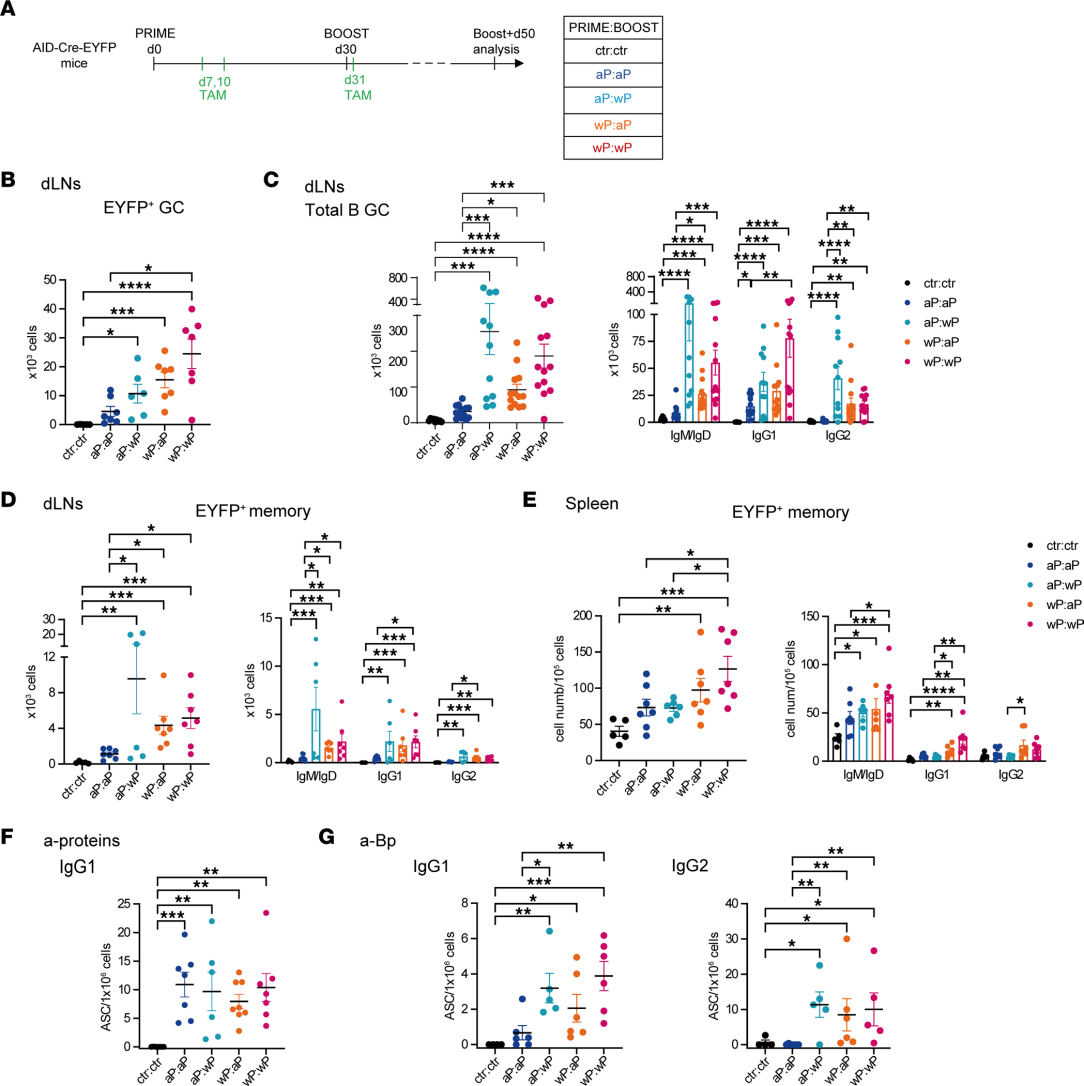
Persistence of GC B cells and local and systemic memory B cells is favored by the wP vaccine. (**A**) AID-Cre-EYFP mice were primed and boosted (day 30) with homologous and heterologous combinations of the aP and wP vaccines or injected with alum (ctr). Three doses of tamoxifen were administrated at days 7, 10, and 31. Mice were analyzed 50 days after boost injection. Cell numbers of EYFP^+^ GCs (**B**) and cell numbers and isotype distribution of total GC B cells (**C**) or EYFP^+^ memory B cells (**D**) were assessed by flow cytometry in dLNs and reported in the graphs. (**E**) Cell numbers and isotype distribution of EYFP^+^ memory B cells were assessed by flow cytometry in spleen. Numbers of IgG1^+^ ASCs against pooled proteins (PT, PRN, FHA, Fim2,3) (**F**) or numbers of IgG1^+^ and IgG2^+^ ASCs against sonicated Bp (**G**) were determined by a memory B cell ELISPOT assay performed 5 days after in vitro activation of splenocytes in the presence of IL-2 and R848. Each point in the graphs represents an individual mouse. At least 2 independent experiments were performed for each analysis. Means (±SEM) are shown. Kruskal-Wallis analysis with uncorrected Dunn’s test was performed to compare the different conditions. *P < 0.05, **P < 0.01, ***P < 0.001, ****P < 0.0001.

It has been shown that long-term protective immunity to Bp depends on memory T and B cells despite serum Ab levels’ decay (17). Hence, we analyzed the persistence of the B cell response until 50 days after boost in animals immunized as in Figure 5A. A sustained EYFP^+^ GC B cell response was detected 50 days after the recall immunization in dLNs and was particularly marked in mice that received at least 1 wP injection (Figure 5B). This was also observed upon analysis of the total GC B cell pool (non-EYFP^+^), i.e., when monitoring GC recruitment outside of the tamoxifen-labeling window (Figure 5C). Isotype profile analysis showed that IgM^+^/IgD^+^ and IgG2^+^ GCs were significantly increased in the wP-containing groups, with IgG1^+^ GCs also present in aP:aP mice (Figure 5C).

EYFP^+^B220^+^GL7^–^ memory B cell abundance and isotype distribution in the dLNs was consistent with the global GC B cell analysis, showing that accumulation of memory B cells was dependent on the wP vaccine (Figure 5D). Since no statistical difference was identified between aP:wP and wP:wP mice, it means that the lower numbers of GC and memory B cells that were generated by priming with aP vaccine are efficiently compensated by the wP boost.

For EYFP^+^ memory B cells in the spleen 50 days after boost, differences were more marked for the 2 wP-primed groups, notably for IgG1^+^ memory B cells (Figure 5E). To evaluate a sustained contribution of persistent GCs to the systemic memory pool, we analyzed the antigen specificity of the total splenic B cell pool. Splenic memory B cells were differentiated into ASCs by in vitro culture of splenocytes for 5 days in the presence of IL-2 and R848, and ELISPOT assays were then performed. Anti-protein IgG1^+^ memory B cell ELISPOT showed splenic memory B cells that were able to differentiate into protein-specific IgG1^+^ ASCs, with no difference between the groups (Figure 5F). Anti-Bp memory ELISPOT showed in contrast that the groups with 1 wP led to significantly higher IgG1^+^ ASC numbers compared with controls (Figure 5G), and the same profile was observed for IgG2^+^ anti-ASCs, which were furthermore undetectable among aP:aP differentiated memory spleen cells (Figure 5G). To sum up, the combination of aP and wP vaccines sustains longer lasting GC reaction and memory B cell responses in both dLNs and at the systemic level as compared with the aP:aP combination.

### Three different signatures of memory B cells were identified by single-cell RNA-Seq analysis in vaccinated mice.

**Figure 6:**
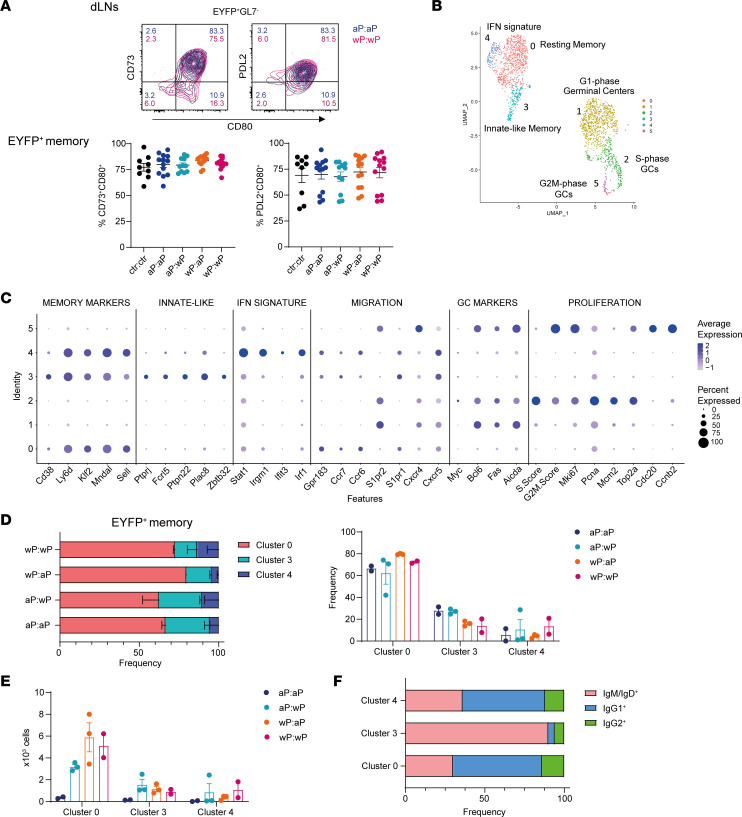
Three different signatures of memory B cells were identified by single-cell RNA-Seq analysis in vaccinated mice. (**A**) The expression of CD73, CD80, and PD-L2 was determined on dLN EYFP^+^GL7^–^ memory B cells by flow cytometry 50 days after boost from AID-Cre-EYFP mice that were primed and boosted (day 30) with homologous and heterologous combinations of the aP and wP vaccines or injected with alum (ctr). Three doses of tamoxifen were administrated at days 7, 10, and 31. Representative flow cytometry plots are shown for aP:aP and wP:wP conditions. CD73^+^CD80^+^ and PD-L2^+^CD80^+^ memory EYFP^+^ populations in dLNs are shown in the graphs for all vaccine combinations. (**B**) Uniform manifold approximation and projection (UMAP) and clustering of 707 EYFP^+^ GC and 1,962 EYFP^+^ memory sorted B cells analyzed by scRNA-Seq from dLNs of AID-Cre-EYFP mice 50 days after boost injection, and tamoxifen administration on days 7, 10, and 31, from 2 aP:aP-, 3 aP:wP-, 3 wP:aP-, and 2 wP:wP-immunized mice. (**C**) Selected gene expression for the 6 different clusters (numbered 0–5) is presented in dot plot scale on normalized unique molecular identifier (UMI) counts. Frequencies (**D**) and absolute numbers (**E**) of EYFP^+^ memory B cells within clusters 0, 3, and 4 are shown for the 4 vaccine combinations. (**F**) Isotype profile determined by flow cytometry during cell sorting is shown for the EYFP^+^ memory B cells from clusters 0, 3, and 4. Means (±SEM) are shown in panels **A**, **D**, and **E**.

Different combinations of Bp vaccines induce memory B cells with distinct isotypic profiles in both dLNs and spleen. We wondered whether the 2 classes of vaccines also induced differences in the phenotype of memory B cells. To this end, we analyzed the expression of maturation markers classically used to discriminate memory B cell subsets in mice: programmed cell death ligand 2 (PD-L2), CD73, and CD80 (31, 32). All EYFP^+^ memory B cells analyzed at 50 days after boost presented a rather homogenous profile, with a major double-positive CD73^+^CD80^+^ and PD-L2^+^CD80^+^ phenotype (Figure 6A, representative plot shown for the aP:aP and wP:wP groups; all conditions are included in the graphs; spleen memory B cell analysis is shown in Supplemental Figure 7A). By quantifying the expression of each of the 3 molecules and comparing the values obtained for the different vaccine strategies, we did not identify any difference in either dLNs or spleen (Supplemental Figure 7B). All vaccine strategies, thus, induced a similar memory B cell profile.

We further characterized memory B cells generated upon pertussis vaccination with a single-cell (sc) RNA-Seq approach, performed 7 weeks after the vaccine boost. B220^+^EYFP^+^ memory B cells from dLNs of mice immunized and tamoxifen fed as in Figure 5A were single-cell sorted, and their gene expression profile was assessed. B220^+^EYFP^+^ GC sorted B cells were used as the control population for the unsupervised clustering analysis of the scRNA-Seq data set. Six different clusters were obtained according to their gene expression profile (Figure 6, B and C, and Supplemental Table 1). Three main signatures of memory B cells were identified. These signatures shared memory-associated markers, such as *Cd38*, *Ly6d*, *Klf2*, *Mndal*, and *Sell*, and cell migration genes, such as *Gp138*, *S1pr1*, *Ccr6*, and *Ccr7*, which are all reported to be overexpressed on memory B cells with respect to GC B cells (33–35). Cluster 0 represents the largest resting memory B cell pool followed by innate-like memory cells expressing genes such as *Fcrl5*, *Ptpn22*, and *Plac8* (cluster 3) and cluster 4 with an IFN signature, marked by overexpression of *Stat1*, *Irgm1*, and *Irf1*. GC B cells were characterized by *Bcl6*, *Fas*, *Aicda*, *Cxcr4*, and *S1pr2* expression. GC B cells were further distributed into G1 phase (cluster 1), S phase (cluster 2), and G2/M phase (cluster 5) cells, according to their relative expression of proliferation-associated genes (Figure 6, B and C, and Supplemental Figure 8, A and B). For each prime:boost vaccine condition, we determined the frequency and cell numbers of sorted EYFP^+^ memory and GC cells into the different clusters. A fraction of cell-sorted memory B cells were found in cluster 1 (a component particularly enlarged in aP:aP-vaccinated mice) (Supplemental Figure 8C). However, intermediate flow cytometry GL7 expression and similarity of cluster 1 memory- and GC-specific gene expression allowed us to exclude those cells from the analysis (Supplemental Figure 8, D and E). The higher contamination of GC B cells in sorted memory B cells from aP:aP mice likely resulted from a lowered double-positive GL7 and PNA expression following 2 cell-sorting passages after an enrichment of total EYFP^+^ cells, while other immunization conditions were analyzed following a first enrichment of EYFP^+^ GC and memory cells before single-cell sorting (detailed in the Methods section). The frequency of memory B cells from cluster 0, 3, and 4 is shown in Figure 6D, where we did not identify major differences according to the vaccine strategies. However, while cluster 4 was marginally enlarged in wP-boosted mice, a trend toward higher frequency of innate-like memory B cells (cluster 3) was shown in aP-primed ones. The main difference between vaccine conditions remained the increased absolute numbers of memory B cells from all 3 clusters in the wP-containing groups (Figure 6E). We further assessed the memory isotype distribution determined by flow cytometry in the 3 clusters and obtained a reduced proportion of the IgG1 and IgG2 isotypes in the innate-like cluster 3 in favor of a predominance of IgM-unswitched memory B cells (Figure 6F). To sum up, memory B cells induced by aP and wP vaccines share a common transcriptomic profile, with the specific feature conferred by the presence of at least 1 wP injection relying on larger numbers of differentiated memory B cells with diverse Ig isotypes.

### Isotype-switched resident-like memory B cells localize in the lungs of wP-primed mice.

**Figure 7:**
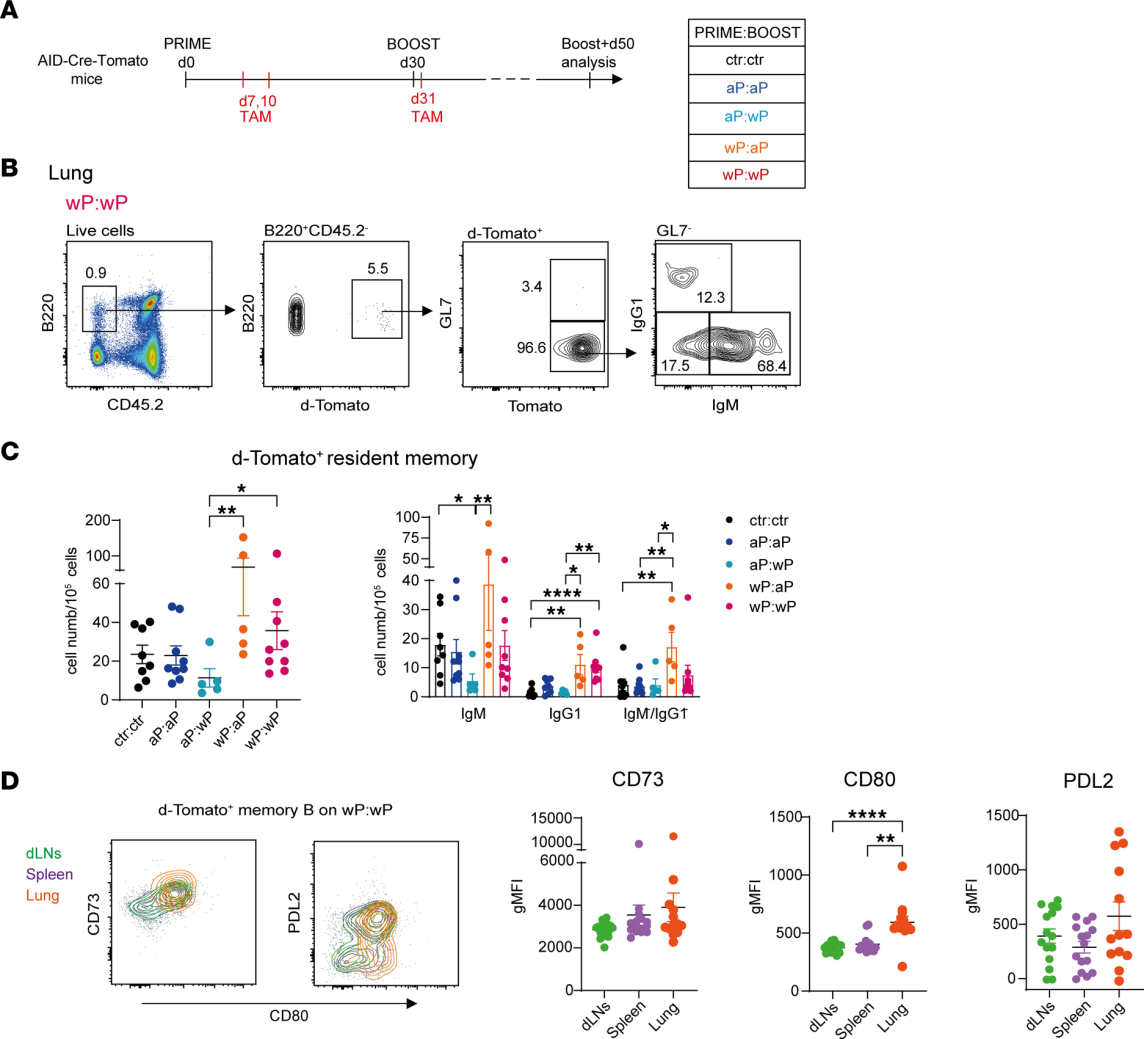
Isotype-switched resident-like memory B cells localize in the lungs of wP-primed mice. (**A**) AID-Cre-Tomato mice, primed and boosted (day 30) with homologous and heterologous aP and wP vaccine combinations, received tamoxifen at days 7, 10, and 31. Analysis was performed 50 days after boost. (**B**) Representative flow cytometry analysis of d-Tomato^+^ cells selected from B220^+^CD45.2^–^ resident lung B cells. d-Tomato^+^GL7^–^ cells were further analyzed for the expression of IgM and IgG1 membrane Ab isotypes. (**C**) Numbers of total d-Tomato^+^ resident memory cells (graph on the left) and their isotype subclasses (graph on the right) are shown. (**D**) dLN, splenic, and lung total d-Tomato^+^ memory B cells belonging to the wP:wP condition were analyzed for the expression of CD73, CD80, and PD-L2 markers. A representative flow cytometry plot is shown. Geometric MFI relative to each membrane marker is indicated in the graphs for all organs. Each point in the graphs (**C** and **D**) depicts an individual mouse. At least 2 independent experiments were performed for each analysis. Means (±SEM) are shown. Kruskal-Wallis analysis with uncorrected Dunn’s test was performed to compare the different conditions. *P < 0.05, **P < 0.01, ****P < 0.0001.

The induction of a lung mucosal immunity is a key arm to prevent the colonization and infection of Bp. Accordingly, aP vaccines have been shown to be inefficient at generating a T cell resident memory population (10). Since we observed a systemic population of memory B cells upon boost, notably at the level of the spleen (Figure 5), we further analyzed the presence of vaccine-induced memory B cells in the lungs of inoculated mice. Very rare memory B cells are generally detected in the lung tissue, and their presence largely depends on local antigen stimulation (36–39). To assess memory B cells in the lung, the AID-Cre-Tomato mouse model was chosen due to its higher labeling efficiency (see Methods). We compared both homologous and heterologous prime plus boost schedules and looked for memory B cells 50 days after boost (Figure 7A). To identify resident lymphoid cells in the lungs, and discriminate them from recirculating B cells, a fluorochrome-coupled anti-CD45.2 mAb was injected intravenously 5 minutes before organ sampling (38). Resident memory B cells are not labeled by the injected Ab and are thus identified as CD45.2^–^B220^+^GL7^–^d-Tomato^+^ (representative gating strategy is shown in Figure 7B). No significant differences were observed between control and vaccinated mice in the number of total d-Tomato^+^ resident memory cells; however, a trend toward increased cell numbers in both wP-primed groups was obtained (Figure 7C). IgG1^+^ and IgM^–^IgG1^–^ memory B cell subsets were increased in wP-primed conditions compared with control mice, a difference particularly marked in the case of the wP:aP group (Figure 7C). The EYFP^+^ memory B cell profile identified in lungs resembles the one analyzed in the spleen, and this may suggest a common origin for these memory B cells. To further characterize this resident memory population, we compared the expression of CD73, CD80, and PD-L2 in memory B cells of dLNs, spleen, and lungs belonging to the wP:wP condition. We found that these memory B cell populations were quite similar between the 3 organs, with the sole difference of higher CD80 expression in the lung microenvironment (Figure 7D). The similar profile observed therefore suggests that this lung memory population, better induced with the wP prime vaccine strategy, migrated from dLNs or spleen and became resident-like in the lung, while further upregulating the CD80 activation marker.

### Formation of large numbers of Bp-specific long-lived PCs in the BM requires 1 wP vaccination.

**Figure 8:**
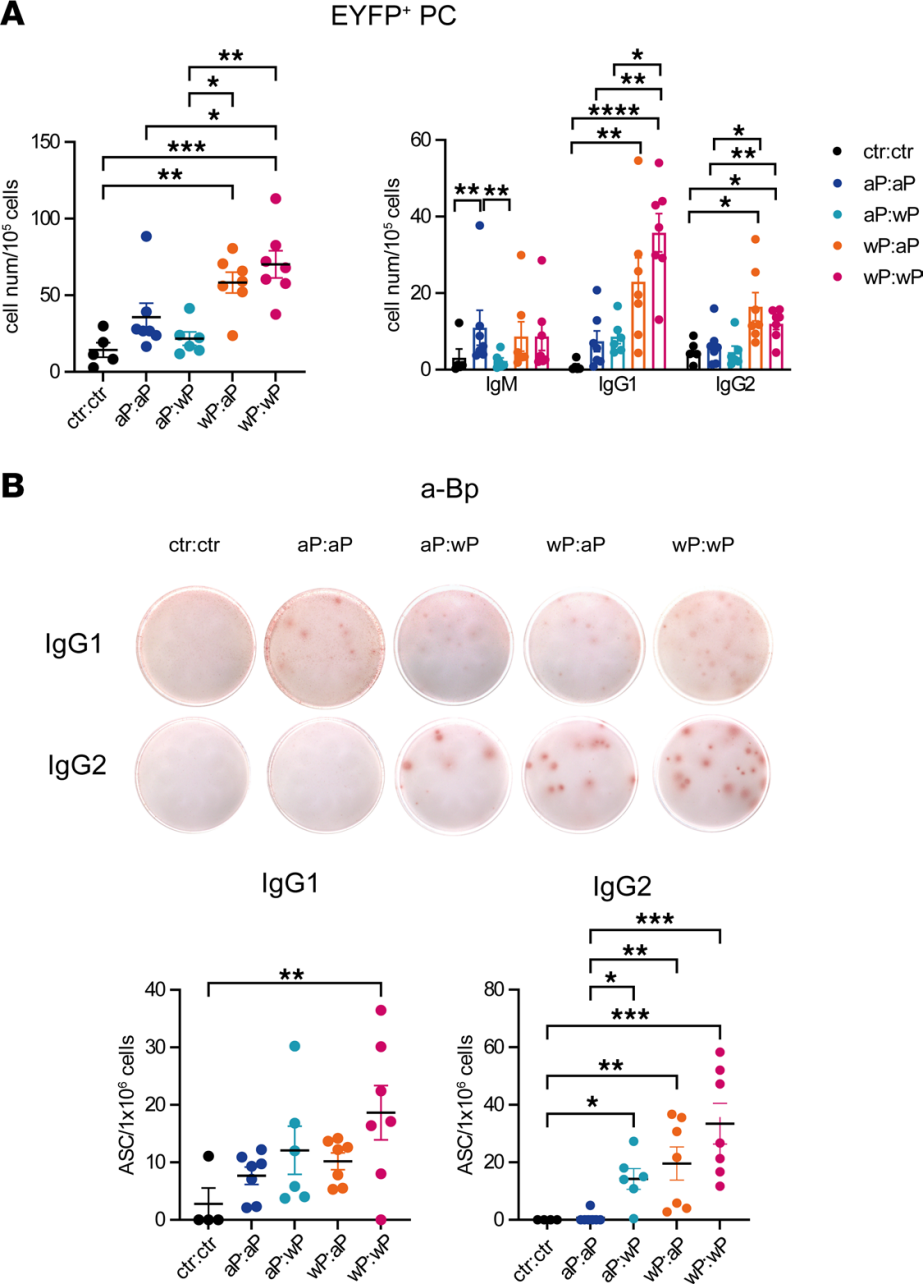
Formation of large numbers of Bp-specific long-lived PCs in the BM requires 1 wP vaccination. AID-Cre-EYFP mice were primed and boosted (day 30) with homologous and heterologous combinations of the aP and wP vaccines or injected with alum (ctr). Three doses of tamoxifen were administrated at days 7, 10, and 31. Mice were analyzed 50 days after boost injection. (**A**) Cell numbers and isotype distribution of EYFP^+^ PCs assessed by flow cytometry in BM are shown in the graphs. (**B**) Numbers of IgG1^+^ or IgG2^+^ ASCs against sonicated Bp were determined by ELISPOT performed on total BM cells. Each point in the graphs represents an individual mouse. At least 2 independent experiments were performed for each analysis. Means (±SEM) are shown. Kruskal-Wallis analysis with uncorrected Dunn’s test was performed to compare the different conditions. *P < 0.05, **P < 0.01, ***P < 0.001, ****P < 0.0001.

In mice immunized and tamoxifen fed as in Figure 5A, we assessed the settlement of EYFP^+^ long-lived PCs in the BM 50 days after the boost. The analysis showed that the 2 wP-primed groups were enriched in PCs compared with aP-primed ones (Figure 8A). These BM long-lived PCs were mostly of the IgG1 isotype, followed by IgG2, both enriched in the wP-primed groups (Figure 8A). To determine the total numbers of antigen-specific PCs, an anti-Bp PC ELISPOT was performed on BM cells. It showed abundant IgG1^+^ ASCs in the wP:wP group and a population of IgG2^+^ anti-Bp ASCs in all groups that presented at least 1 wP vaccination (Figure 8B). This increased presence of IgG2^+^ PCs at day 50 in the total BM PC population suggests a slower kinetics of the IgG2 response compared with IgG1, thus emerging outside the tamoxifen-labeling window.

Collectively, these data show that 1 dose of the wP vaccine, independent of its use in the prime or the boost, is sufficient for the production of Bp-specific long-lived PCs with broader isotypic profile.

### Production of Bp-specific Abs with broader antigen and isotypic diversity in recall responses is favored by combining wP and aP vaccines.

**Figure 9:**
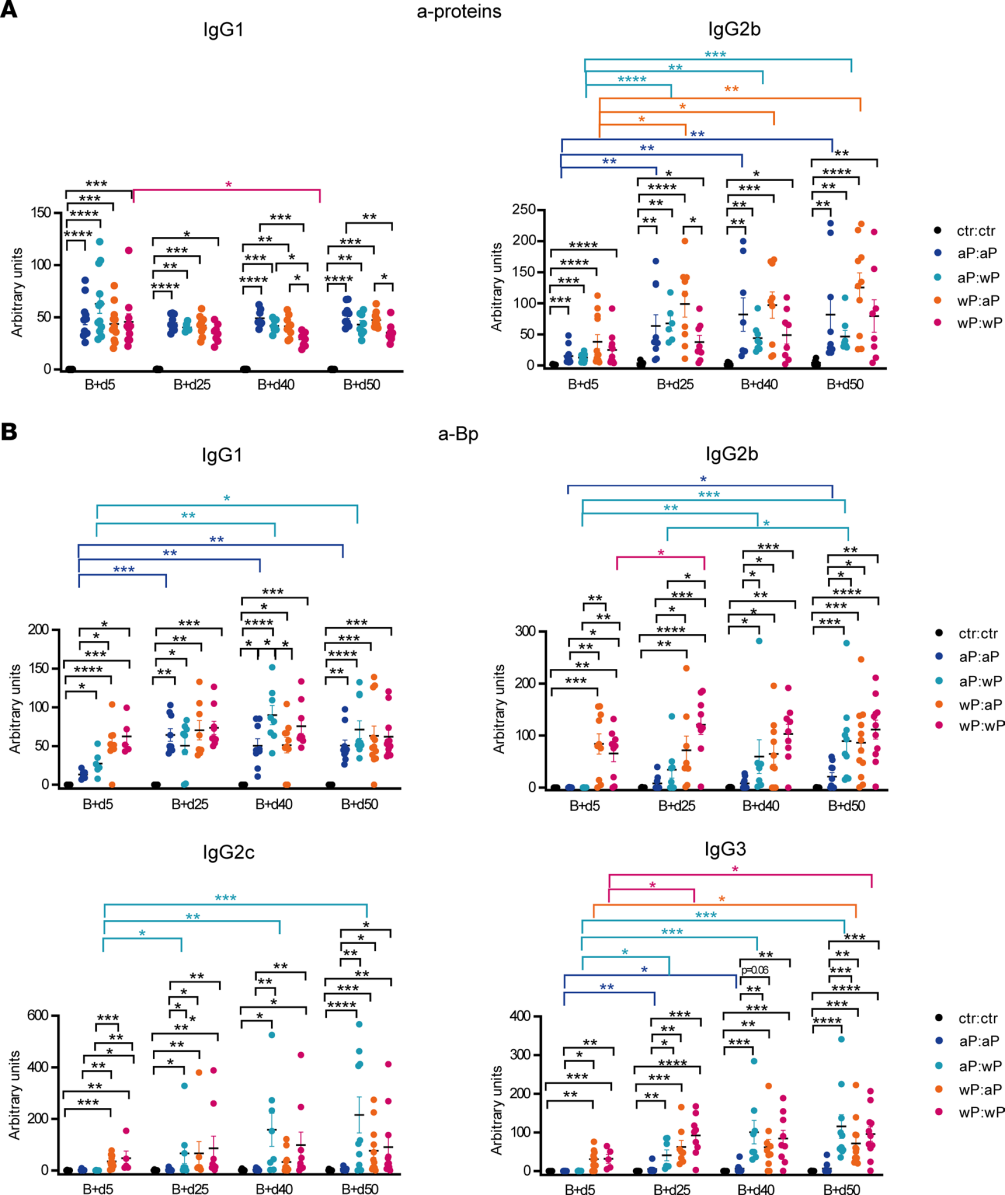
Production of Bp-specific Abs with broader antigen and isotypic diversity in recall responses is favored by combining wP and aP vaccines. AID-Cre-EYFP mice were primed and boosted (day 30) with homologous and heterologous combinations of the aP and wP vaccines or injected with alum (ctr). Blood was collected at 5, 25, 40, and 50 days after boost injection. IgG1 and IgG2b Ab titers against pooled proteins (PT, PRN, FHA, Fim2,3) (**A**) or IgG1, IgG2b, IgG2c, and IgG3 Ab titers against sonicated Bp (**B**) were detected by ELISA from serum of vaccinated and control mice. Ab titers are arbitrary values and each point in the graphs represents data from an individual mouse. At least 2 independent experiments were performed for each analysis. Means (±SEM) are shown. Kruskal-Wallis analysis with uncorrected Dunn’s test was performed to compare the different conditions at each time point and the different time points between the same condition. *P < 0.05, **P < 0.01, ***P < 0.001, ****P < 0.0001.

To determine the different kinetics and qualities of Ab production according to different prime:boost strategies, we performed a time course titration for the anti-protein– and anti-Bp–specific Abs at day 5, 25, 40, and 50 after boost. As observed in the prime response, IgG1 and IgG2b were the switched isotypes induced against the protein pool of the aP formulation (Figure 9A). During the course of the response, the wP:wP combination was the only one showing a slight drop of IgG1 anti-protein titers, with, at the end point, lower levels if compared with the 2 groups that received the aP boost. In accordance to serum analysis of the prime, lower anti-PT IgG1 Abs in wP:wP mice were identified 5 and 50 days after boost (Supplemental Figure 9), likely accounting for the lower total anti-protein Ab levels observed for the wP:wP group. IgG2b anti-proteins Abs, in contrast, increased with time in all groups, in agreement with the observed delay in the IgG2b response.

Abs against the whole sonicated Bp were also measured. At day 5 after boost, IgG1 Bp-specific Abs were more abundant in the 2 wP-primed groups, due to the higher levels of anti-Bp IgG1 Abs generated during the prime (Figure 9B; see Figure 1C). Anti-Bp IgG1 Ab levels increased later in the 2 aP-primed groups until reaching comparable levels with the wP-primed ones, the higher anti-protein Ab output compensating for the lower antigenic coverage of the aP vaccine. IgG2b, IgG2c, and IgG3 anti-Bp Abs were higher in the wP-primed groups, and, in the course of the response, the aP:wP group reached Ab levels that became similar to the 2 wP-primed groups. In contrast, the anti-Bp IgG2b, IgG2c, and IgG3 titers of the aP:aP group did not differ from controls and were significantly lower compared with all conditions that contained the wP in the prime or/and in the boost, being barely detectable for the IgG2c and IgG3 isotypes (Figure 9B). In conclusion, the aP vaccine fails to elicit the antigenic and isotypic diversity of Bp-specific Abs that 1 single dose of the wP vaccine suffices to induce.

## Discussion

The whole inactivated pertussis bacteria vaccination, which drastically reduced the disease in infants, was abandoned in most high-income countries due to the existence of adverse effects. As a consequence, it was replaced by an acellular version composed of a mixture of Bp proteins. This transition was hypothesized to have contributed to recent resurgence of the disease. While all pertussis vaccines on the market are effective in protecting against the disease, the protection they elicit has limited duration and wanes over a number of years as well as with natural infection (40, 41). In order to understand the mechanism of action of both types of pertussis vaccines, we used an AID fate-mapping mouse model that allows an in-depth description of effector and memory B cell responses.

First, we observed that, by using modified vaccine preparations, the wP formulation, compared with the aP one, produced faster and stronger local GC and memory B cell responses in the dLNs upon prime vaccination, as well as PCs in the BM with broader antigenic and isotypic diversity. In the serum, IgG1 and IgG2b aP protein-specific Abs were induced by both vaccine formulations, whereas IgG2b, IgG2c, and IgG3 anti-Bp Abs were uniquely elicited by the wP vaccine.

The type of pertussis vaccine used as a prime in infants, irrespective of the nature of booster doses, seems to determine the later immunological profile, especially as concerns T cell polarization (23–26). However, the outcome of a heterologous aP:wP prime:boost combination has not been explored in humans to our knowledge but could be investigated and compared with other vaccine settings in our mouse model.

Mice were immunized twice, s.c., at 30 days’ interval and analyzed 5 and 50 days after the boost, with a homologous aP:aP or wP:wP prime:boost schedule and with heterologous aP:wP or wP:aP combinations. The wP:wP prime:boost gave rise to a higher B cell response including GC and memory B cells and PCs in dLNs 5 days after boost. Tamoxifen was also given 1 day after the boost, allowing us to mark the recruitment of naive B cells into GCs. In all vaccine combinations, the frequency of labeled GC B cells had approximatively doubled compared with that observed upon labeling during the sole priming. These results suggested that, in these vaccine settings, the return of memory B cells into GCs was quantitatively much more robust than previously reported (42). Memory B cells, as well as PCs, were also detected at the systemic level in all boosted groups, notably in the spleen. These cells were more abundant in the wP:wP condition, and, concerning memory B cells, mostly expressed IgM at their surface.

Persistent vaccine-induced GC B cells were still observed 50 days after boost in dLNs and at higher frequencies in all conditions that contained at least 1 wP vaccine. The capacity of the aP:wP condition to induce a long-lasting activation of secondary GC B cells was the most noticeable effect. Moreover, Ab isotype expression in persistent GC B cells 50 days after boost was qualitatively different from the profile observed 5 days after boost that merely reflected the profile of the prime. Notably, the IgG2^+^ GC B cell subset increased with time and depended on the injection of the wP vaccine, in the prime or in the boost. Similarly, memory B cell populations in dLNs were enlarged in all wP-containing groups compared with the aP:aP combination. Differences in isotype expression and surface markers in mice have been attributed to functional differences of memory B cell subsets (27, 32). However, the CD73^+^, CD80^+^, and PD-L2^+^ expression pattern of the memory B cell subsets in either dLNs or spleen was very similar between the different vaccine conditions, despite their different isotypic profile, being mostly CD73^+^CD80^+^, and largely, but more variably, PD-L2^+^. Although sharing a common phenotype, pertussis vaccine–induced memory B cells segregated into 3 transcriptomic profiles: resting, innate-like unswitched, and IFN signature memory B cells. While only minor differences in the distribution of memory B cells into these 3 clusters were observed with the different vaccine schedules, all types of memory B cells remained numerically inferior in the aP:aP-vaccinated mice compared with all the other groups. The vaccine type and additional numbers of the antigens contained in the wP formulation, irrespective of the prime-dependent T cell polarization, play therefore a major role in the profile of the B cell activation.

Memory resident-like B cells were observed in the lung tissue, at a higher level in the wP prime immunization. These cells displayed higher CD80 expression as compared with their dLN and splenic counterparts. Intranasal and pulmonary pertussis immunization are able to induce memory B cells in the lungs (12–14); however, such cells have never been identified after a parenteral vaccination to our knowledge. The finding of isotype-switched memory B cells in the lungs of vaccinated mice, which represent bona fide vaccine-induced memory B cells that expanded following a wP prime, suggests increased mucosal protection conferred by this vaccine schedule. While EYFP^+^ dLN local memory B cells were increased and were more isotype diverse when the wP vaccine was used in prime and/or boost, splenic and lung peripheral memory B cells were more abundant and isotype diverse in wP-primed animals. This suggests that the migration of the fate-mapped memory B cells occurs early after boost immunization and is consistent with the delayed IgG2^+^ B cell differentiation in aP:wP mice.

In BM, an ELISPOT assay performed on total BM cells identified similar numbers of anti-Bp PCs secreting IgG1 in all conditions and more numerous IgG2-secreting PCs only in those containing at least 1 wP injection. Together, these results support the notion that in a recall response, the wP vaccine, even on aP prime, is able to restore the effect produced in wP-primed groups for what concerns B cell responses. We showed that, regardless of the combination chosen, a boost vaccination generated analogous levels of IgG1 and IgG2b anti-protein Abs in serum 5 and 50 days after boost. But, while IgG1 Abs already peaked 5 days after boost, IgG2 protein-specific Abs increased with time in all conditions. IgG2b, IgG2c, and IgG3 anti-Bp Ab titers were lower 5 days after boost in aP-primed mice but, differently from IgG1 Abs, these titers increased only in the aP:wP group and reached the same final values observed in wP-primed groups. The sole lower performance of the wP:wP vaccine was in the induction of anti-PT IgG1 Ab, likely corresponding to the lower PT content of this preparation (28, 29). Altogether, the wP boost was able to reorient the Th2-type Ab production induced by the aP priming.

In conclusion our study sustains the idea that a larger GC and memory B cell expansion can be achieved by using wP vaccines and that the imprint conferred by the wP boosting dominates in the B cell response of aP-primed subjects. The ability of the wP vaccine compared with the aP-only formulation to trigger Abs of various IgG subclasses may furthermore be decisive against Bp colonization and asymptomatic transmission, especially in case of Bp strains lacking aP antigens, such as PRN-deficient Bp (43). Additional Ab subtypes may also be beneficial through the activation of a wide set of Fcγ receptor–expressing effector cells.

Even though our work is far from a direct clinical application, these results can be instrumental for further studies aimed at establishing whether heterologous pertussis vaccine combinations could, possibly through different formulations or nasal immunization (12, 13, 44), promote a similar B cell bias with broader isotypic profile and higher and longer lasting protective potential.

## Methods

### Mouse lines.

AID reporter mice were generated in our animal facility by breeding homozygous male AID-Cre-ERT2 × ROSA26-loxP-EYFP mice or AID-Cre-ERT2 × ROSA26-loxP-d-Tomato reporter mice with WT C57BL/6 female mice and are named “AID-Cre-EYFP” or “AID-Cre-Tomato” throughout the manuscript. As the ROSA26-loxP-d-Tomato reporter displays higher accessibility to the Cre enzyme, the AID-Cre-Tomato line has higher GC labeling efficiency, which, by flow cytometry, results in 70% labeling of total GL7^+^ cells and cannot exclude activated B cells (not shown). The use of this mouse line was therefore restricted to the study of resident memory B cell subsets. Mice that were 8–14 weeks old were used in the work.

### Mouse vaccination.

Mice were s.c. injected at the base of the tail with 100 μL of aP and wP vaccine formulations, provided by Sanofi-Pasteur, Marcy l’Etoile (Lyon), which correspond to 1/5 of the human dose but do not correspond to commercial vaccines as they lack additional components like tetanus or diphtheria toxins. The aP vaccine is composed of 20 μg/mL of PT; 6 μg/mL of PRN; 10 μg/mL of Fim2,3; 10 μg/mL of FHA; and 0.66 mg/mL alum; the wP vaccine is made of 30 opacity units/mL whole-cell pertussis and 2.4 mg/mL alum. Control mice were injected with the aluminum hydroxide gel adjuvant (InvivoGen) diluted 16% in PBS.

### Tamoxifen regimen.

Doses of 10 mg/mouse Nolvadex (tamoxifen; AstraZeneca) in 300 μL of 20% Clinoleic (Baxter) were administered by gavage at indicated time points after prime and boost vaccination. Control mice received tamoxifen as well.

### Cell isolation.

Single-cell suspensions from mouse spleen and both inguinal dLNs were obtained via mechanical disruption through 40 μm cell strainer (Falcon, Corning). BM cells were obtained by flushing femurs and tibiae and passed through 40 μm cell strainer (Falcon, Corning). Spleen and BM homogenates were deprived of red blood cells trough 1× RBC Lysis Buffer (eBioscience). For lung cell analysis, mice were intravenously injected with 2 μg of Alexa Fluor 700 anti–mouse CD45.2 Ab in PBS 5 minutes prior to euthanasia. Lungs were perfused with PBS, and a single-cell suspension was prepared by using the lung dissociation kit, mouse (Miltenyi Biotec), followed by incubation on a GentleMacs Octo Dissociator with heaters (Miltenyi Biotec) according to the manufacturer’s instruction. Pulmonary leukocytes were further separated with a Percoll density gradient (Cytiva).

### Flow cytometry analysis.

Cell suspensions from spleen, inguinal dLNs, BM, and lungs were incubated for 30 minutes on ice with fluorochrome-labeled Abs or primary biotin-conjugated Abs followed by fluorochrome-labeled streptavidin in PBS supplemented with 0.5% BSA. Live/Dead viability marker (Invitrogen) was used to exclude dead cells from analysis. For intracellular staining, cells were first stained for surface antigens, then fixed and permeabilized with the Cytofix/Cytoperm kit (BD Biosciences). Cells were then resuspended in PBS containing 2% FCS and acquired on BD LSR Fortessa cytometer. Analyses were performed with FlowJo (Tree Star Inc.) software. Antibodies and staining reagents are listed in Supplemental Table 2.

### ELISA.

To determine serum Ab levels, 96-well Nunc-immuno plates (Thermo Fisher Scientific) were coated overnight at 4°C with pooled proteins composing the aP vaccine (5 μg/mL FHA, 1 μg/mL toxoid, 5 μg/mL PRN, 2 μg/mL Fim2,3) or 5 × 10^9^ CFU/mL sonicated (Bioruptor Pico; Diagenode) heat-inactivated Bp (provided by Sanofi-Pasteur) and blocked with 1% BSA. Mouse sera, collected at specified time points, were appropriately diluted and incubated 2 hours at room temperature. A serum reference, composed of a mix of immunized sera, was used as standard in all analysis. Goat anti-mouse IgM-, IgG1-, IgG2a/c-, IgG2b- IgG3-, and IgA-HRP (human ads, Southern Biotech) were used for detection. KPL TMB Microwell Peroxidase Substrate System (Seracare) and colorimetric spectrophotometry at 405 and 620 nm were used to determine OD.

### Multiplex Meso Scale Discovery assay.

Meso Scale Discovery 96-well plates were used to quantify simultaneously Ab level to 5 antigens. In each well, pertussis toxin, FHA, PRN, FIM 2/3, and heat-killed Bp antigens were printed on separated spots by Meso Scale Discovery. Plates were blocked with 1% milk. Diluted mouse serum samples, in-house reference standards, and quality controls were incubated 1 hour, 30 minutes, at 37°C. Goat anti-mouse IgG1, IgG2c, IgG2b, IgG3, and IgA detection Abs (RD-Biotech) were Sulfo-Tag–labeled according to MSD GOLD SULFO-TAG NHS-Ester kit instructions and used for detection. Electrochemiluminescence was read on MSD instrument (SECTOR). Reference standard curves (4-parameter logistic regression) were used to calculate Ab levels expressed in AU/mL.

### Cell culture and ELISPOT assay.

ASCs were enumerated by ELISPOT. For PC ELISPOT, freshly flushed BM cells were used; for memory B cell ELISPOT, splenocytes were put in culture for 5 days in complete RPMI-1640 medium (10% FCS, 10 mM HEPES, 1× nonessential amino acids, 1 mM sodium pyruvate, 5.5 × 10^–5^ M 2-mercaptoethanol, 100 U/mL penicillin, 100 μg/mL streptomycin, Gibco) supplemented with 10 ng/mL recombinant IL-2 (Peprotech) and 1 μg/mL R848 (InvivoGen) to induce memory B cell differentiation into PCs. MultiScreen HTS 96-well plates (MilliporeSigma) coated overnight at 4°C with pooled aP-composing proteins (5 μg/mL FHA, 1 μg/mL toxoid, 5 μg/mL PRN, 2 μg/mL Fim2,3) or 5 × 10^9^ CFU/mL sonicated (Bioruptor Pico; Diagenode) heat-inactivated Bp (Sanofi-Pasteur) and blocked with 1% BSA. Cells were washed and resuspended in fresh RPMI-1640 complete medium and 3-fold serial dilutions were incubated overnight at 37°C and 5% CO_2_. Goat anti-mouse IgM-, IgG1-, IgG2- (a/c + b), IgG3-, and IgA-HRP (human ads, Southern Biotech) were used for detection. HRP activity was revealed by using 3-amino-9-ethylcarbazole (BD Biosciences) following manufacturer’s instructions. Red spots corresponding to individual ASCs were quantified with an ELISPOT reader using the AID software (version 3.5; AutoImmun Diagnostika) and manually counted.

### SORT-Seq and ssRNA-Seq data analysis.

B220^+^EYFP^+^PNA^+^GL7^+^ GC and B220^+^EYFP^+^PNA^–^GL7^–^ memory B cells from dLNs of aP:wP-, wP:aP-, and wP:wP-vaccinated mice were first enriched by cell sorting, and then viable GC and memory B cells were single-cell FACS-sorted into 384-well capture plates (Single Cell Discoveries) and immediately stored at –80°C. aP:aP dLN cells were first FACS enriched as B220^+^EYFP^+^ cells, due to their lower cell frequency, and then B220^+^EYFP^+^PNA^+^GL7^+^ GC and B220^+^EYFP^+^PNA^–^GL7^–^ memory B cells were single-cell sorted. scRNA-Seq was performed according to an adapted version of the SORT-Seq protocol (45) with primers described (46). Following amplification, library preparation was done following the CEL-Seq2 protocol (47). The DNA library was paired-end sequenced on an Illumina NextSeq 500, high output, with a 1 × 75 bp Illumina kit. Reads were mapped on *Mus musculus* mm10 reference transcriptome, including mitochondrial genes with BWA-MEM (48). Data were demultiplexed as described (49). Mapping and generation of count tables were automated using *MapAndGo* script. Analysis of data was performed on R version 4.1.2 using Seurat package version 4.1.1 (50). Low-quality cells were excluded using 10% mitochondrial gene expression and 1,800 UMI thresholds. After integration and normalization used to minimize differences generated from technical preparations, principal component analyses (30 principal components) were performed on variable genes and embedded in 2-dimensional UMAP plots. Clustering was performed with *FindNeighbors* and *FindClusters* methods using 30 principal components and 0.5 resolution. *FindAllMarkers* methods of the Seurat package was used to find marker genes between clusters and calculated with the Wilcoxon rank sum test on normalized counts. Scoring of cell cycle was calculated from Seurat Package *CellCycleScoring* function. Dot plots were generated to represent average expression of selected genes of cells in different clusters on normalized counts. RNA-Seq data have been deposited in the ArrayExpress database at EMBL-EBI (www.ebi.ac.uk/arrayexpress) under accession number E-MTAB-12156.

### Statistics.

Results are shown as mean (±SEM). To assess statistical significance, Kruskal-Wallis analysis with uncorrected Dunn’s test was performed with GraphPad Prism 9 Software. *P* < 0.05 was considered statistically significant.

### Study approval.

All experimental protocols were approved by the Ethics Committee of Paris Descartes University (CEEA34, Paris, France) and validated by the French Ministry of Research.

## Author contributions

VV, PB, JCW, and CAR designed the experiments. VV and AS performed most of the experiments, together with DL. PB performed the serum multiplex assay. CC performed scRNA-Seq experiments, together with PC for the scRNA-Seq analysis. VV, JCW, and CAR wrote the paper. All the authors critically reviewed the manuscript.

## Supplementary Material

Supplemental data

## Version 1 09/22/2022

In-Press Preview

## Version 2 11/08/2022

Electronic publication
