# Supplementary material for: The whole-cell pertussis vaccine imposes a broad effector B cell response in mouse heterologous prime-boost settings
Source: JCI Insight. 2022 Nov 8;7(21):e157034. doi: 10.1172/jci.insight.157034 (PMC9675447; doi:10.1172/jci.insight.157034)
Supplement: Supplemental data [file jciinsight-7-157034-s020.pdf]

The whole-cell pertussis vaccine imposes a broad effector B-cell response in mouse heterologous prime-boost settings

Viviana Valeri[1], Akhésa Sochon[1*], Clara Cousu[1*], Pascal Chappert[1], Damiana Lecoeuche[1], Pascal Blanc[2**], Jean-Claude Weill[1**] and Claude-Agnès Reynaud[1**]

1 : Institut Necker-Enfants Malades, INSERM U1151/CNRS UMR 8253, Université Paris Descartes, Sorbonne Paris Cité, 75993 Paris Cedex 14, France.

2 : Sanofi-Pasteur R&D, Marcy l'Etoile, France

* These authors equally contributed to the work

** Shared senior authorship

Corresponding author: Viviana Valeri, Institut Necker-Enfants Malades, INSERM U1151, Faculté de Médicine Paris Descartes, 150-156 rue de Vaugirard, 75015 Paris, phone (+33) 0614968400, email viviana.valeri@inserm.fr

Conflict of interest: Outside of the submitted work, J.-C.W. received consulting fees from Institut Mérieux. P.B. is employed by Sanofi-Pasteur and may hold shares and/or stock options in the company. Other authors have nothing to disclose.

Supplemental figure 1

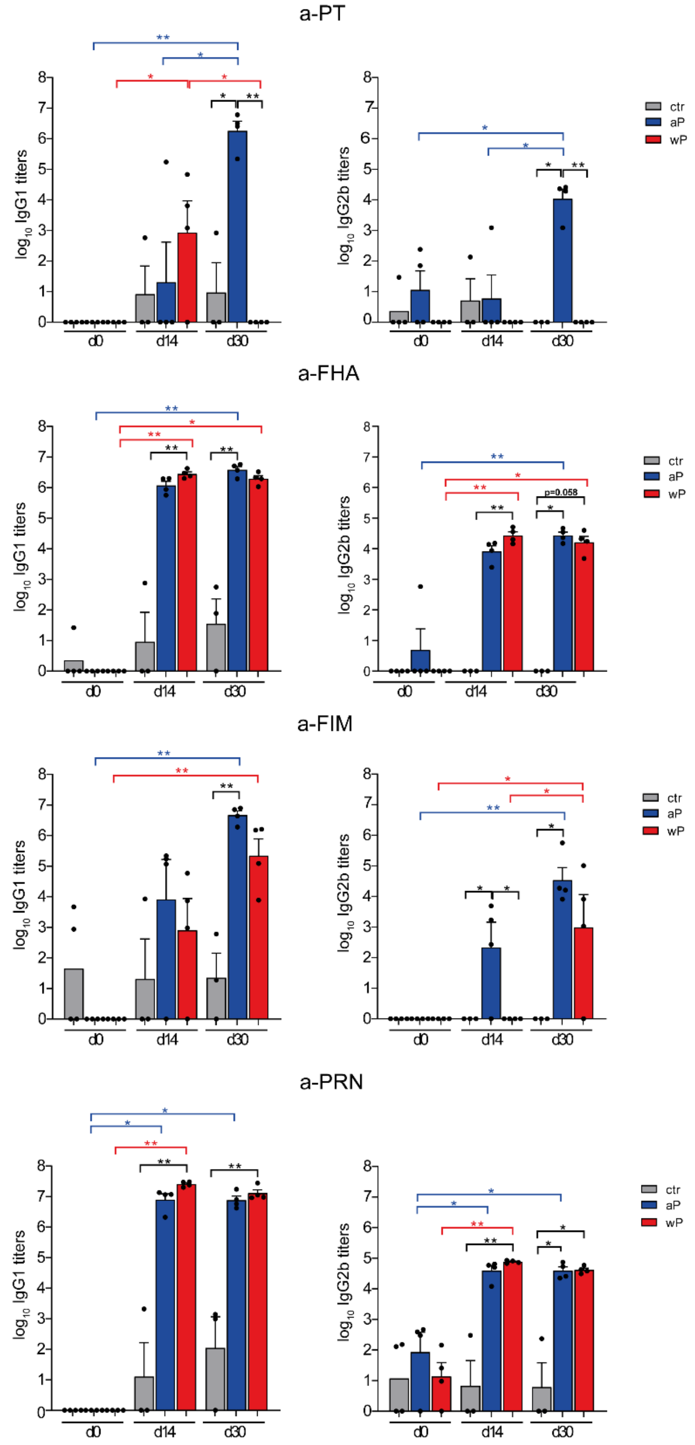

Supplemental figure 1: The wP-prime is inefficient at inducing anti-Pertussis Toxin (PT) IgG1^{+} and IgG2b^{+} antibodies. Blood was collected at d0, d14 and d30 from AID-Cre-EYFP mice s.c. injected either with aP (acellular Pertussis) or wP (whole-cell Pertussis) vaccines, or with Alum (ctr=control mice). Multiplex MSD® assay was performed to detect IgG1 and IgG2b titers directed against each single aP protein: PT, PRN, FHA, Fim2,3. Each point in the graphs represents an arbitrary unit expressed in log_{10} relative to an individual mouse. A representative experiment is shown. Means (±SEM) are shown. Kruskal-Wallis analysis with uncorrected Dunn's test was performed to compare the different conditions at each time point and the different time points between the same condition. *$p<0.05$, **$p<0.01$.

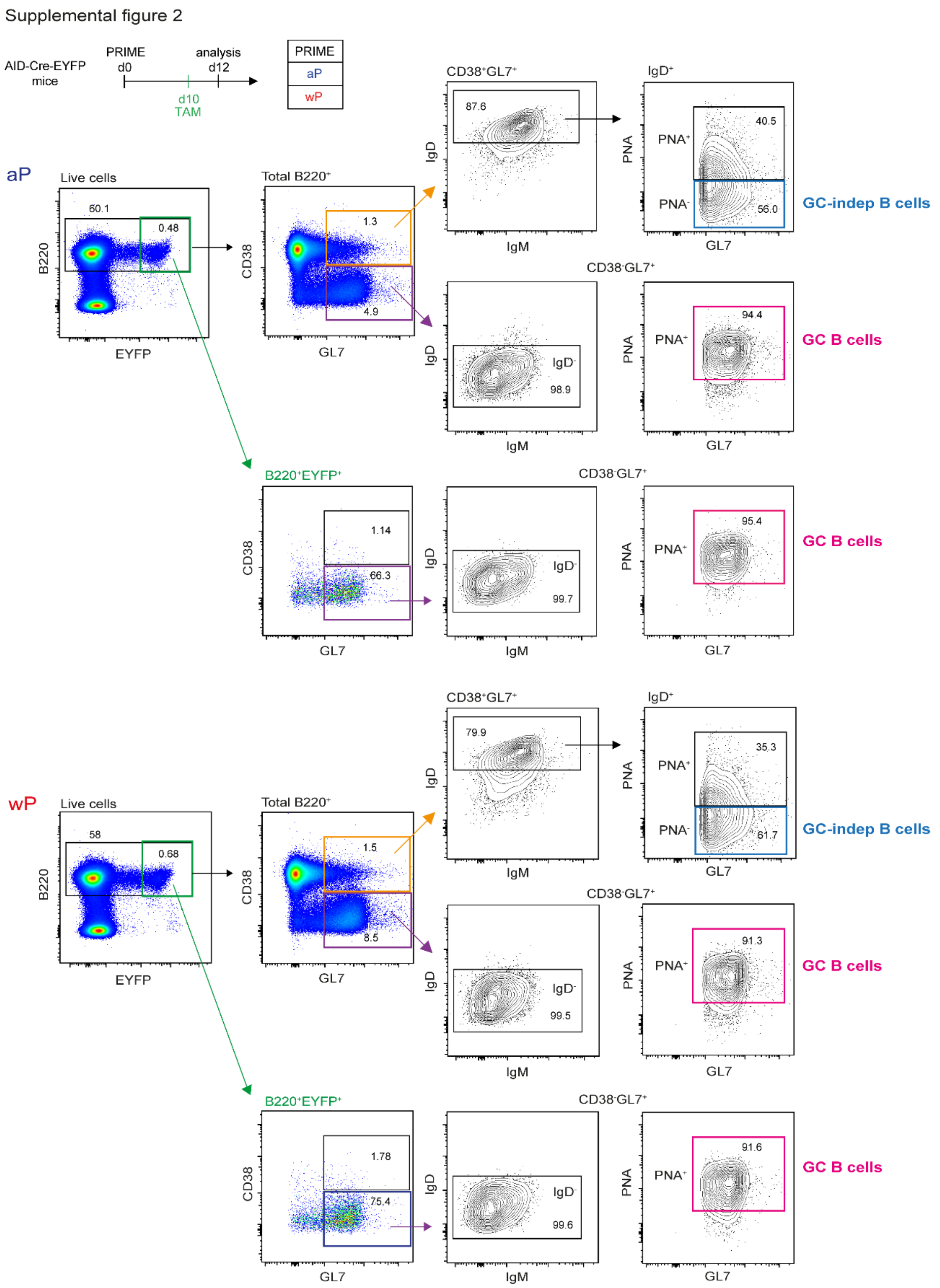

Supplemental figure 2: The AID-Cre-EYFP fate mapping model does not mark GC-independent, activated B cells. AID-Cre-EYFP mice were s.c. injected either with aP or wP vaccines, received tamoxifen gavage on d10 and dLNs were analyzed on d12. Representative flow cytometry plots for both aP- and wP-vaccinated mice show CD38 versus GL7 labeling on total B220^{+} B cells. Germinal center (GC)-independent B cells can be identified among the CD38^{+}GL7^{+} and IgD^{+} cell population. Those cells are largely PNA^{-} cells. CD38^{-}GL7^{+} cells represent GC B cells which are IgD^{-} and PNA^{+} cells. The EYFP^{+} labeled GL7^{+} B cell population consists almost entirely of CD38^{-}, IgD^{-} and PNA^{+} GC B cells.

Supplemental figure 3

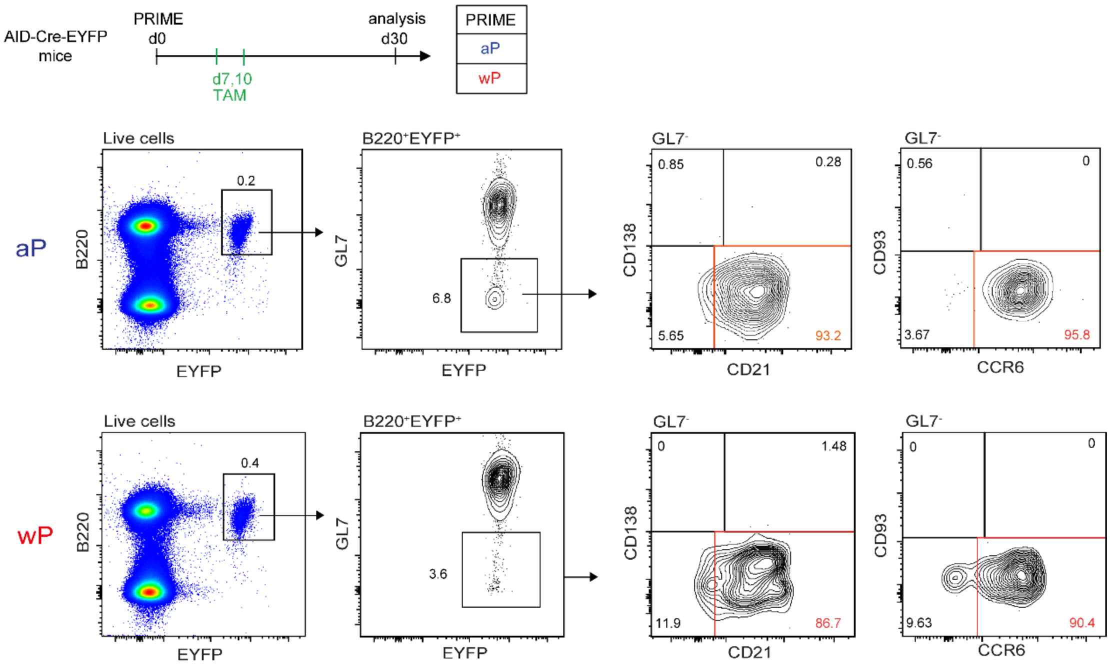

Supplemental figure 3: B220$^+$EYFP$^+$GL7$^-$ memory B cells do not include plasmablasts. AID-Cre-EYFP mice were primed with aP or wP vaccines, received two doses of tamoxifen at d7 and 10 after prime vaccination and dLNs were analyzed on d30. B220$^+$EYFP$^+$GL7$^-$ gated memory B cells were analyzed by flow cytometry for the expression of CD138, CD21, CD93 and CCR6 markers. Two representative analyses show that the memory B cell population is largely CD21$^+$CD138$^-$ and CCR6$^+$CD93$^-$.

Supplemental figure 4

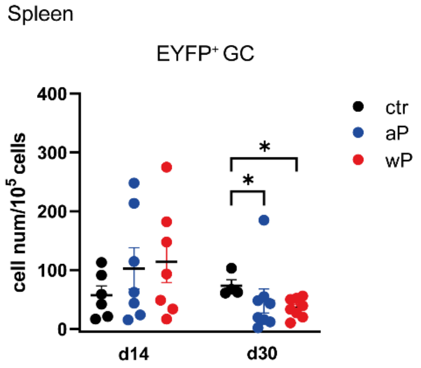

Supplemental figure 4: No primary GC B cell response is identified in spleen of vaccinated mice. AID-Cre-EYFP mice were primed with aP or wP vaccines or injected with Alum (ctr) and received two doses of tamoxifen at d7 and 10 after prime vaccination. Mouse spleens were analyzed at d14 and 30 and EYFP$^+$ GC B cell numbers (relative to 10^5 splenocytes) are shown in the graph. At least two independent experiments were performed for the analysis. Each point in the charts represents an individual mouse. Means (±SEM) are shown. Kruskal-Wallis analysis with uncorrected Dunn's test was performed to compare the different conditions at each time point. *$p<0.05$.

Supplemental figure 5

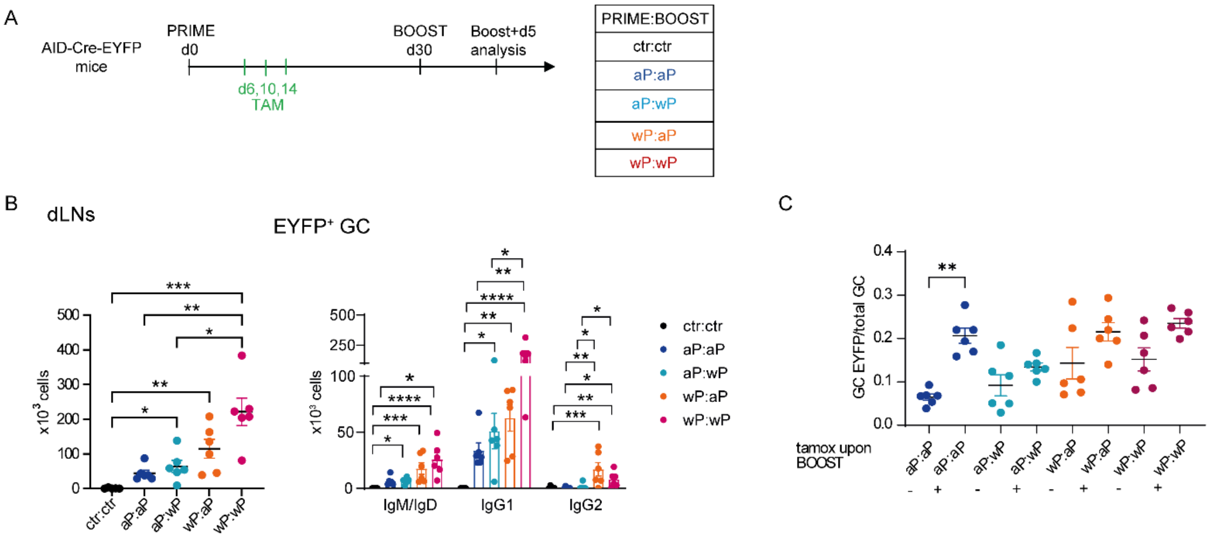

Supplemental figure 5: A comparable amount of naïve B cell recruitment between the aP and wP primed groups is observed upon boost. (A) AID-Cre-EYFP mice were primed and boosted (day 30) with homologous and heterologous combinations of the aP and wP vaccines or injected with Alum. Three doses of tamoxifen were administrated at d6, 10 and 14. Mice were analyzed at d35. **(B)** The graphs show cell numbers and isotype distribution of EYFP^{+} GC cells determined by flow cytometry in dLNs. **(C)** Ratios between EYFP^{+} GC and total GC B cells in dLNs from two different tamoxifen strategies are reported in the plot. Each point in the graphs represents an individual mouse. At least two independent experiments were performed for each analysis. Means (±SEM) are shown. Kruskal-Wallis analysis with uncorrected Dunn's test was performed to compare the different conditions. *p<0.05, **p<0.01, ***p<0.001. ns=not statistically significant.

Supplemental figure 6

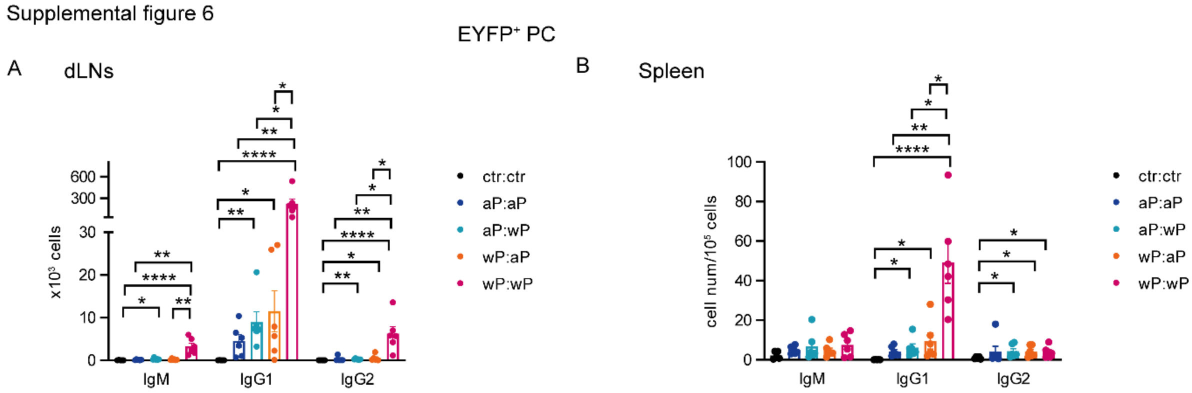

Supplemental figure 6: IgG1^{+} PCs are predominant compared to other isotypes. AID-Cre-EYFP mice were primed and boosted (day 30) with homologous and heterologous combinations of the aP and wP vaccines or injected with Alum (ctr). Three doses of tamoxifen were administrated at d7, 10 and 31. Mice were analyzed at d35. EYFP^{+} PCs were assessed by flow cytometry in dLNs **(A)** or spleen **(B)** and isotype distribution is reported in the graphs. Each point depicts an individual mouse and at least two independent experiments were performed for each analysis. Means (±SEM) are shown. Kruskal-Wallis analysis with uncorrected Dunn's test was performed to compare the different conditions. *p<0.05, **p<0.01, ****p<0.0001.

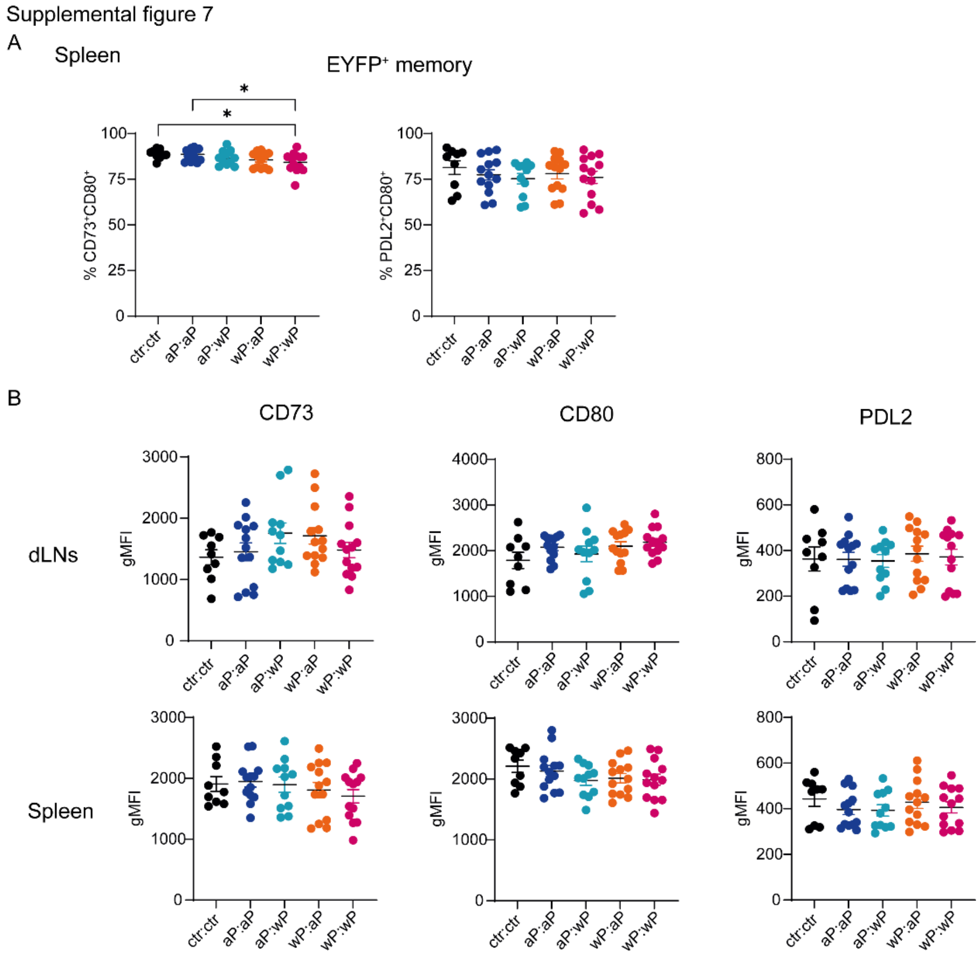

Supplemental figure 7: The phenotype of vaccine-induced memory B cells in dLNs and the spleen is homogenous between the groups. The expression of CD73, CD80 and PDL2 was determined on EYFP$^+$GL7$^-$ memory cells by flow cytometry 50 days after boost from AID-Cre-EYFP mice that were primed and boosted (day 30) with homologous and heterologous combinations of the aP and wP vaccines or injected with Alum (ctr). Three doses of tamoxifen were administrated at d7, 10 and 31. **(A)** CD73$^+$CD80$^+$ and PDL2$^+$CD80$^+$ splenic memory EYFP$^+$ populations are shown in the graphs. **(B)** gMFI are indicated in the graphs for each membrane marker and for all vaccine combinations for both dLNs and spleen. Each point depicts an individual mouse and at least two independent experiments were performed for each analysis. Means (±SEM) are shown. Kruskal-Wallis analysis with uncorrected Dunn's test was performed to compare the different conditions. *$p<0.05$.

Supplemental figure 8

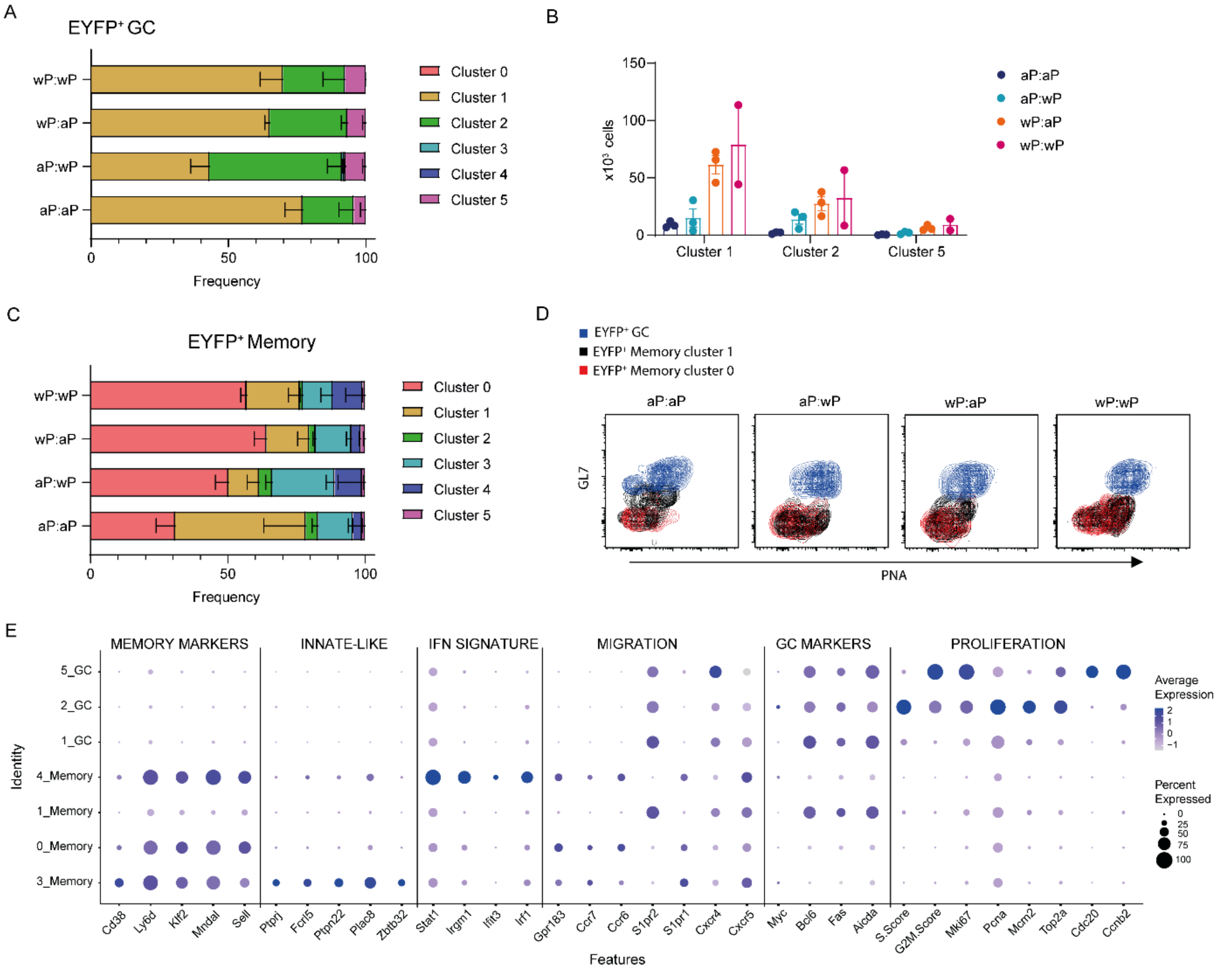

Supplemental figure 8: Distribution of GC and memory B cells into 6 clusters by sc RNA-seq analysis. (A) Frequency of EYFP+ GC B cells in the different clusters are shown for the four vaccine combinations. **(B)** EYFP+ GC B cell absolute numbers are shown for clusters 1, 2 and 4. **(C)** Frequencies of EYFP+ memory B cells in the different clusters are shown for the four vaccine combinations. **(D)** GL7 *versus* PNA flow cytometry profile is determined for EYFP+ GC (in blue), EYFP+ memory cluster 1 (in black) and EYFP+ memory cluster 0 (in red) for the four vaccine combinations. (**E**) Selected gene expression of both EYFP+ memory and GC cells for the different clusters is presented in Dot Plot scale on normalized UMI counts, highlighting the similarity of GC and memory B cells from cluster 1.

Supplemental figure 9

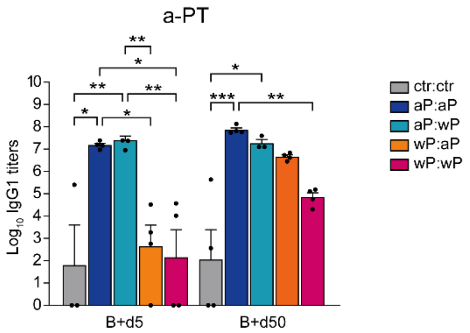

Supplemental figure 9: The wP:wP prime:boost combination is less efficient at inducing anti-Pertussis Toxin (PT) IgG1$^+$ antibodies during a recall response. Blood was collected 5 and 50 days after boost from AID-Cre-EYFP mice that were primed and boosted (day 30) with homologous and heterologous combinations of the aP and wP vaccines or injected with Alum (ctr). Multiplex MSD® assay was performed to detect IgG1 titers directed against PT. Each point in the graphs represents an arbitrary unit expressed in log_{10} relative to an individual mouse. A representative experiment is shown. Means (±SEM) are shown. Kruskal-Wallis analysis with uncorrected Dunn’s test was performed to compare the different conditions at each time point. *p<0.05, ** p<0.01, ***p<0.001.

Supplemental table 1: List of differentially expressed genes

See pdf file: “Supplemental table 1. List of differentially expressed genes.pdf”

Supplemental table 2: List of antibodies and reagents used in the work

Antibody	Fluorochrome, clone	Manufacturer	Catalog number	Batch
Anti-mouse B220	APC-efluor780, clone RA3-6B2	eBioscience	47-0452-82	2272766
Anti-mouse CD138	PE-Cy7, clone 281-2	Biolegend	142514	B291316
Anti-mouse CD45.2	AlexaFluor 700, clone 104	Biolegend	109821	
Anti-mouse CD73	PE-Cy7, clone eBioTY/11.8	eBiosciences	25-0731-82	2016450
Anti-mouse CD80	APC, clone 16-10A1	Biolegend	104714	B227918
Anti-mouse CD80	BV510, clone 16-10A1	Biolegend	104741	B331397
Anti-mouse GL7	Efluor450, clone GL7	eBioscience	48-5902-82	2062737
Anti-mouse GL7	PerCP-Cy5.5, clone GL7	Biolegend	144610	B326932
Anti-mouse IgA	PE, clone 11-44-2	eBioscience	12-5994-81	2252069
Anti-mouse IgA	Biotin, clone RMA-1	Sony	2635015	155924
Anti-mouse IgD	PerCP-Cy5.5, clone 11-26c2a	BD Biosciences	564273	9066574
Anti-mouse IgG1	APC, clone X56	BD Biosciences	550874	58836
Anti-mouse IgG2a	PE, clone RMG2a-62	Biolegend	407108	B300880
Anti-mouse IgG2b	PE, clone RMG2b-1	Biolegend	406708	B321175
Anti-mouse IgM	BV605, clone RMM-1	Biolegend	406523	B323099
Anti-mouse PDL2	BV510, clone TY25	BD Biosciences	740194	9268285
Anti-mouse PDL2	Biotin, clone TY25	eBioscience	13-5986-82	B230798
Anti-mouse PNA	Biotin	Vector Laboratories	B-1075	ZD1101
**Other staining reagents**	**(Fluorochrome)**	**Manufacturer**	**Catalog number**	**Batch**
Live/Dead staining kit	Excitation 405nm	Invitrogen	L34957	2145008
Live/Dead staining kit	Excitation UV 350nm	Invitrogen	L34961	2264488
7AAD viability solution		Biolegend	420403	
Streptavidin	BV785	Biolegend	405249	B230798
Streptavidin	PE-Cy7	Sony	2626030	178342
1X RBC Lysis Buffer		eBioscience	00-4333-57	

p_val	avg_log2FC	pct.1	pct.2	p_val_adj	cluster	gene
1,6E-145	1,230958	0,901	0,281	2E-141	0	Mndal
4,4E-128	-1,79087	0,397	0,811	5,5E-124	0	Igj
4,7E-125	-1,51427	0,086	0,646	5,9E-121	0	Rgs13
1,1E-120	1,309692	0,749	0,24	1,3E-116	0	Sell
4E-118	1,037446	0,994	0,739	4,9E-114	0	Shisa5
1,4E-116	-1,52203	0,487	0,822	1,7E-112	0	Basp1
9E-114	-1,37216	0,159	0,671	1,1E-109	0	Mef2b
2,5E-109	1,024092	0,814	0,295	3,1E-105	0	Ifi203
1,1E-108	0,996173	0,939	0,535	1,4E-104	0	Gimap4
9,4E-108	1,093507	0,726	0,236	1,2E-103	0	Klf2
1,3E-107	1,179154	0,857	0,428	1,6E-103	0	Cmah
4,9E-99	0,967934	0,849	0,355	6,12E-95	0	Ly6d
4,52E-98	0,95967	0,706	0,247	5,6E-94	0	Capg
7,05E-98	-0,95247	1	0,999	8,73E-94	0	Cfl1
1,26E-92	-1,13456	0,13	0,59	1,56E-88	0	S1pr2
3,78E-91	0,955561	0,772	0,301	4,67E-87	0	Pml
4,19E-88	0,834378	0,62	0,178	5,18E-84	0	S100a10
4,93E-84	0,843936	1	0,957	6,1E-80	0	Malat1
1,44E-83	-1,06206	0,159	0,6	1,78E-79	0	Bcl6
8,26E-79	0,780385	0,936	0,745	1,02E-74	0	Btg1
1,04E-77	0,839504	0,483	0,111	1,29E-73	0	Gpr183
1,56E-76	-1,06969	0,162	0,583	1,93E-72	0	8430410A17Rik
7,23E-76	0,736021	0,426	0,082	8,95E-72	0	Ccr6
5,14E-74	-0,96916	0,494	0,804	6,37E-70	0	Txn1
7,97E-74	0,572115	1	0,993	9,87E-70	0	B2m
5,27E-71	0,752539	0,584	0,183	6,52E-67	0	Bcl2
8,03E-69	0,821085	0,943	0,758	9,95E-65	0	Macf1
8,28E-69	-1,37274	0,22	0,592	1,03E-64	0	Hmgb2
2,19E-68	-0,80503	0,07	0,45	2,71E-64	0	Eaf2
5,72E-68	0,763115	0,457	0,112	7,08E-64	0	Zfp318
6,1E-67	0,64707	0,622	0,211	7,56E-63	0	Gm1966
9,57E-65	-0,69103	0,897	0,969	1,19E-60	0	Nap1l1
1,24E-63	-0,71949	0,049	0,406	1,54E-59	0	Lipc
1,73E-63	0,585272	1	0,97	2,15E-59	0	H2-K1
1,09E-62	0,665348	0,487	0,144	1,34E-58	0	Itgb7
9,62E-62	-0,68446	0,039	0,384	1,19E-57	0	Nuggc
1,29E-61	-0,93604	0,265	0,633	1,6E-57	0	Aicda
9,51E-61	0,824525	0,79	0,502	1,18E-56	0	Lmo2
4,09E-60	-0,73899	0,296	0,674	5,06E-56	0	Ppp4r2
4,93E-60	0,79108	0,777	0,461	6,11E-56	0	Bank1
2,07E-59	-0,75133	0,497	0,793	2,56E-55	0	Arpc5l
2,21E-59	-0,85516	0,213	0,583	2,74E-55	0	Dck
1,27E-58	-0,59497	0,996	0,998	1,58E-54	0	Actg1
2,1E-58	0,726274	0,664	0,292	2,6E-54	0	Sub1
3,24E-58	-0,78767	0,778	0,916	4,02E-54	0	Cd24a
1,55E-57	0,736845	0,626	0,29	1,92E-53	0	Anxa6
1,68E-57	-0,58604	0,977	0,994	2,08E-53	0	Ms4a1
2,17E-57	-0,95239	0,203	0,547	2,69E-53	0	Gcsam
2,96E-57	0,6469	0,483	0,153	3,67E-53	0	Serpinb1a

8,24E-57	-0,7678	0,638	0,846	1,02E-52	0	Gapdh
2,63E-56	-1,17118	0,162	0,502	3,26E-52	0	Stmn1
3,42E-56	-0,65673	0,077	0,418	4,23E-52	0	Rassf6
4,6E-56	0,777161	0,699	0,367	5,7E-52	0	Fam65b
5,66E-55	0,657514	0,446	0,131	7E-51	0	Cd55
1,26E-54	0,386912	1	1	1,56E-50	0	H2-Aa
1,76E-54	0,771255	0,62	0,296	2,18E-50	0	Add3
4,96E-54	-0,88093	0,314	0,643	6,14E-50	0	Mtf2
1,24E-53	-0,77912	0,196	0,547	1,54E-49	0	Mbd4
4,15E-53	-0,66384	0,207	0,557	5,14E-49	0	Glrx3
5,57E-53	-0,88856	0,371	0,69	6,89E-49	0	Klhl6
6,48E-53	0,496477	0,999	0,984	8,03E-49	0	H2-D1
1,67E-52	0,672669	0,616	0,271	2,06E-48	0	Snn
2,38E-52	0,656015	0,407	0,112	2,95E-48	0	Arhgef18
5,07E-52	-0,55128	0,025	0,321	6,27E-48	0	Rgs10
5,83E-52	0,378106	1	0,999	7,22E-48	0	Rps24
9,1E-52	-0,6426	0,094	0,418	1,13E-47	0	Dstn
1,49E-51	-0,73599	0,439	0,741	1,84E-47	0	Hmgn1
2,88E-51	0,455814	1	0,998	3,57E-47	0	Ly6e
3,89E-51	0,725494	0,612	0,283	4,82E-47	0	B3gnt5
9,43E-51	-0,82969	0,47	0,739	1,17E-46	0	Top1
1,09E-50	0,692797	0,762	0,483	1,35E-46	0	Gimap6
3,91E-49	-0,65278	0,188	0,527	4,84E-45	0	Dcaf12
4,17E-49	0,567093	0,287	0,053	5,16E-45	0	Myo1f
1,45E-48	-0,72351	0,664	0,866	1,79E-44	0	Pou2af1
4,31E-48	0,702685	0,543	0,237	5,34E-44	0	Map3k1
7,3E-48	0,832611	0,672	0,379	9,04E-44	0	Dock10
8,01E-48	-0,67076	0,168	0,49	9,92E-44	0	2700029M09Rik
9,68E-48	0,669647	0,817	0,607	1,2E-43	0	Scd1
1E-47	0,537465	0,296	0,059	1,24E-43	0	Kctd14
7,36E-47	-1,02902	0,613	0,806	9,11E-43	0	Hmgn2
5,59E-46	0,740368	0,677	0,383	6,92E-42	0	Txnip
9,1E-46	-0,79514	0,184	0,498	1,13E-41	0	Lpp
1,09E-45	0,621945	0,73	0,428	1,35E-41	0	Gimap3
2,26E-45	-0,6078	0,242	0,573	2,8E-41	0	Rfc1
5,13E-45	-0,64579	0,661	0,858	6,35E-41	0	Slc25a5
6,25E-45	-1,10015	0,536	0,759	7,74E-41	0	H2afz
9,22E-45	0,650633	0,91	0,73	1,14E-40	0	Mycbp2
2,32E-44	-0,68948	0,528	0,782	2,87E-40	0	Rhoh
5,44E-44	-1,05981	0,128	0,417	6,73E-40	0	Mki67
6,78E-44	0,458277	1	0,991	8,4E-40	0	Rps21
1,99E-43	0,749668	0,672	0,396	2,47E-39	0	Itga4
2,57E-43	-0,67868	0,343	0,645	3,18E-39	0	Apobec1
2,93E-43	-0,64849	0,114	0,413	3,63E-39	0	Rgs2
6,18E-43	0,593776	0,496	0,213	7,65E-39	0	Lrrc33
1,24E-42	0,62407	0,848	0,675	1,54E-38	0	Stk17b
1,28E-42	0,510031	0,428	0,15	1,58E-38	0	Hhex
2,82E-42	-0,88375	0,207	0,496	3,49E-38	0	Mcm6
3,22E-42	-0,76859	0,475	0,713	3,99E-38	0	Ran
7,09E-42	0,73389	0,772	0,566	8,78E-38	0	B4galnt1

3E-41	0,591651	0,361	0,112	3,72E-37	0	Scml4
6,52E-41	-0,52873	0,065	0,333	8,07E-37	0	Sh2b2
7,67E-41	-0,56663	0,154	0,45	9,5E-37	0	Dap
1,47E-40	-0,4739	0,035	0,286	1,82E-36	0	Mybl1
4,51E-40	0,747113	0,568	0,296	5,58E-36	0	Dgka
1,53E-39	-0,476	0,07	0,337	1,89E-35	0	Ccnd3
1,6E-39	-0,52139	0,106	0,385	1,98E-35	0	Xrcc1
4,07E-39	0,424476	0,219	0,035	5,04E-35	0	Fam46a
4,51E-39	0,661415	0,681	0,423	5,59E-35	0	Itm2b
7,67E-39	-0,57609	0,143	0,432	9,5E-35	0	Lpin2
1,17E-38	-0,58739	0,981	0,984	1,45E-34	0	Ucp2
1,44E-38	0,59519	0,501	0,235	1,79E-34	0	Ier5
1,88E-38	0,510027	0,229	0,041	2,32E-34	0	Sspn
4,18E-38	-0,70343	0,291	0,579	5,17E-34	0	Hpse
4,48E-38	0,543224	0,491	0,229	5,55E-34	0	Tspan32
5,7E-38	0,57416	0,949	0,857	7,06E-34	0	Zfp36l1
1,09E-37	0,843314	0,754	0,564	1,35E-33	0	Fcer2a
1,09E-37	-0,58368	0,864	0,94	1,35E-33	0	Ppia
1,34E-37	-0,48006	0,941	0,965	1,66E-33	0	Myl6
2,15E-37	0,531711	0,526	0,262	2,67E-33	0	Myl12b
2,58E-37	-0,48984	0,074	0,328	3,19E-33	0	Cecr2
2,89E-37	0,426431	0,286	0,072	3,58E-33	0	Cnn3
3,46E-37	-0,63666	0,396	0,667	4,28E-33	0	Tmem131
4,72E-37	-0,65683	0,346	0,627	5,84E-33	0	Marcksl1
7,98E-37	-0,50789	0,193	0,472	9,88E-33	0	Dbi
1,37E-36	-0,49331	0,987	0,995	1,69E-32	0	Pabpc1
1,87E-36	-0,55249	0,216	0,506	2,31E-32	0	Odc1
3,53E-36	-0,5479	0,162	0,441	4,37E-32	0	Ell3
4,53E-36	-0,64181	0,409	0,679	5,61E-32	0	Mbd2
6,09E-36	-0,47699	0,029	0,252	7,54E-32	0	H1fx
7,07E-36	0,604539	0,659	0,417	8,76E-32	0	Gimap8
1,15E-35	0,508745	0,543	0,276	1,42E-31	0	Cyb561a3
2,81E-35	-0,42767	0,991	0,994	3,48E-31	0	Rpl28
2,86E-35	-0,58081	0,622	0,805	3,54E-31	0	Ywhae
5,99E-35	0,616476	0,625	0,366	7,42E-31	0	Evi2b
9,66E-35	-0,43593	0,045	0,274	1,2E-30	0	Endou
1,75E-34	0,484553	0,409	0,166	2,17E-30	0	2900060B14Rik
2,5E-34	0,418843	0,358	0,123	3,09E-30	0	Sbk1
2,8E-34	-0,44058	0,113	0,374	3,46E-30	0	Mtmr14
3,21E-34	-0,46701	0,072	0,311	3,97E-30	0	Gadd45b
6,59E-34	-0,59005	0,478	0,732	8,16E-30	0	Erp44
8,59E-34	-0,44769	0,974	0,988	1,06E-29	0	Arpc2
1,67E-33	-0,47206	0,945	0,977	2,07E-29	0	Calm1
1,81E-33	-0,56268	0,672	0,844	2,24E-29	0	Anp32b
3,14E-33	-0,56428	0,329	0,59	3,88E-29	0	Pgk1
4,57E-33	-0,52083	0,226	0,499	5,66E-29	0	Helq
6,2E-33	-0,45033	0,107	0,358	7,68E-29	0	Havcr1
6,26E-33	0,416546	0,996	0,971	7,75E-29	0	Rpl37a
9,78E-33	-0,49649	0,12	0,37	1,21E-28	0	C130026I21Rik
1,04E-32	-0,50163	0,393	0,671	1,29E-28	0	Polr1d

1,13E-32	-0,54308	0,514	0,744	1,4E-28	0	Ppp1ca
1,32E-32	-0,36118	0,042	0,258	1,64E-28	0	Neil1
1,43E-32	0,5285	0,365	0,135	1,77E-28	0	S1pr1
2,9E-32	-0,53754	0,419	0,673	3,59E-28	0	Tcea1
4,54E-32	-0,50476	0,086	0,32	5,62E-28	0	Mcm3
6,21E-32	-0,51241	0,643	0,838	7,69E-28	0	Atp5b
6,47E-32	-0,52052	0,517	0,743	8,01E-28	0	Prdx1
8,79E-32	-0,56584	0,672	0,845	1,09E-27	0	Mzb1
8,92E-32	0,470511	0,925	0,819	1,1E-27	0	D4Wsu53e
8,98E-32	-0,52884	0,588	0,783	1,11E-27	0	Ube2d2a
9,74E-32	-0,466	0,132	0,384	1,21E-27	0	Bzw2
1,08E-31	-0,43441	0,114	0,359	1,34E-27	0	Smagp
1,32E-31	-0,33793	0,028	0,23	1,63E-27	0	Ada
1,84E-31	-0,45028	0,319	0,594	2,28E-27	0	Mtss1
2,23E-31	0,657459	0,849	0,722	2,76E-27	0	Btla
2,36E-31	0,575981	0,542	0,305	2,92E-27	0	Stk10
2,87E-31	-0,5941	0,075	0,298	3,55E-27	0	Cks2
3,27E-31	-0,78932	0,122	0,355	4,04E-27	0	Top2a
3,75E-31	0,483469	0,888	0,774	4,64E-27	0	Ctss
5,51E-31	-0,47749	0,894	0,934	6,82E-27	0	Rbm3
7,26E-31	0,507251	0,88	0,748	8,99E-27	0	Faim3
8,35E-31	0,463947	0,367	0,15	1,03E-26	0	Ppcs
9,19E-31	-0,52159	0,216	0,48	1,14E-26	0	Anxa2
1,07E-30	-0,41031	0,122	0,367	1,32E-26	0	Hmgn3
1,82E-30	0,437867	0,399	0,168	2,26E-26	0	Cdkn1b
1,86E-30	-1,08387	0,484	0,682	2,3E-26	0	Pcna
1,9E-30	-0,33467	0,012	0,195	2,35E-26	0	Gnb4
2,09E-30	-0,4145	0,039	0,241	2,59E-26	0	Gatm
2,41E-30	-0,50666	0,271	0,535	2,98E-26	0	Cmpk1
4,46E-30	0,680474	0,645	0,435	5,52E-26	0	Vim
6,16E-30	-0,34997	0,007	0,183	7,63E-26	0	Gm2447
7,88E-30	-0,51059	0,704	0,84	9,76E-26	0	Atp5a1
8,11E-30	0,596471	0,529	0,304	1E-25	0	Clic4
1,69E-29	-0,64035	0,1	0,322	2,09E-25	0	Mcm5
1,7E-29	-0,40725	0,064	0,278	2,1E-25	0	Cnst
5,91E-29	-0,36529	0,042	0,241	7,32E-25	0	Stxbp1
6,57E-29	0,380207	0,999	0,983	8,13E-25	0	Rpl38
6,83E-29	-0,51878	0,352	0,587	8,46E-25	0	Rbbp7
7,44E-29	-0,52075	0,396	0,653	9,21E-25	0	Foxo1
7,69E-29	0,484036	0,412	0,189	9,52E-25	0	Man1a
1,55E-28	-0,45217	0,68	0,839	1,92E-24	0	Clic1
1,58E-28	0,554691	0,6	0,377	1,95E-24	0	Filip1l
2,17E-28	-0,49767	0,599	0,796	2,68E-24	0	Atp5g3
2,98E-28	-0,4512	0,101	0,322	3,69E-24	0	Dut
5,19E-28	-0,44639	0,062	0,264	6,42E-24	0	Cdca7
5,34E-28	0,34374	0,248	0,073	6,61E-24	0	Fxyd5
6,24E-28	0,542901	0,548	0,325	7,73E-24	0	Mylip
6,63E-28	-0,50074	0,283	0,542	8,21E-24	0	Psip1
6,91E-28	-0,44424	0,217	0,463	8,56E-24	0	Taf9
1E-27	-0,28476	1	1	1,24E-23	0	Actb

1,17E-27	0,450942	0,388	0,172	1,45E-23	0	Itpr1
1,86E-27	-0,84595	0,094	0,3	2,3E-23	0	2810417H13Rik
1,99E-27	-0,50667	0,225	0,465	2,46E-23	0	Slbp
2,3E-27	-0,39525	0,048	0,241	2,85E-23	0	Nmral1
3,64E-27	-0,60882	0,457	0,671	4,51E-23	0	Bach2
4,94E-27	-0,56315	0,714	0,841	6,11E-23	0	Hmgb1
7,87E-27	-0,31424	0,065	0,268	9,74E-23	0	Amz2
8,58E-27	0,382753	0,246	0,075	1,06E-22	0	Ccr7
1,09E-26	-0,55402	0,209	0,436	1,35E-22	0	Pafah1b3
1,13E-26	0,264603	0,168	0,031	1,4E-22	0	Rnf144a
1,45E-26	-0,44278	0,104	0,314	1,8E-22	0	Mcm2
2,49E-26	0,463417	0,601	0,405	3,08E-22	0	Acap1
2,65E-26	-0,45844	0,474	0,715	3,28E-22	0	Pold4
2,92E-26	-0,46536	0,413	0,643	3,61E-22	0	Psmb7
4,4E-26	-0,45235	0,23	0,476	5,45E-22	0	Pnp
5,27E-26	0,364312	0,264	0,087	6,52E-22	0	Ckap4
5,62E-26	0,438978	0,701	0,476	6,96E-22	0	Samd9l
6,88E-26	-0,43442	0,477	0,707	8,52E-22	0	Cox6b1
8,01E-26	-0,56334	0,612	0,773	9,91E-22	0	Hnrnpab
8,09E-26	-0,46089	0,065	0,256	1E-21	0	Dhfr
8,36E-26	-0,40889	0,043	0,223	1,04E-21	0	Uhrf1
8,42E-26	-0,3932	0,077	0,279	1,04E-21	0	Cyp51
8,62E-26	0,369091	0,303	0,113	1,07E-21	0	Il10rb
1,82E-25	-0,44813	0,294	0,537	2,26E-21	0	Rere
1,89E-25	-0,51509	0,484	0,704	2,35E-21	0	Ldha
2,29E-25	0,549998	0,623	0,407	2,84E-21	0	Abca1
2,58E-25	0,310257	1	0,996	3,19E-21	0	Ddx5
2,89E-25	-0,41953	0,201	0,434	3,58E-21	0	Polr2g
3,13E-25	0,487864	0,536	0,327	3,88E-21	0	Ubl3
4,94E-25	-0,3467	0,043	0,222	6,11E-21	0	Efnb1
8,17E-25	0,416957	0,841	0,67	1,01E-20	0	Ly6a
8,3E-25	0,366902	0,307	0,119	1,03E-20	0	Abcg1
9,17E-25	-0,51079	0,423	0,64	1,14E-20	0	Scaf11
9,48E-25	-0,44212	0,339	0,582	1,17E-20	0	Ssbp3
1,15E-24	-0,52192	0,123	0,326	1,42E-20	0	Asf1b
1,17E-24	-0,35678	0,106	0,314	1,44E-20	0	Fut8
1,61E-24	-0,42318	0,519	0,737	1,99E-20	0	Cox6c
1,69E-24	-0,38571	0,978	0,983	2,09E-20	0	Hnrnpa2b1
2,14E-24	0,374506	0,42	0,209	2,65E-20	0	Esyt1
2,26E-24	0,348271	0,248	0,083	2,79E-20	0	Il27ra
4,45E-24	-0,44035	0,287	0,523	5,51E-20	0	Anp32e
4,66E-24	-0,45116	0,238	0,464	5,76E-20	0	Tpi1
4,78E-24	-0,35086	0,054	0,232	5,92E-20	0	Cpne5
4,84E-24	-0,30401	0,062	0,247	5,99E-20	0	Parp8
4,91E-24	0,469087	0,41	0,21	6,08E-20	0	Chd2
5,28E-24	-0,38757	0,116	0,324	6,53E-20	0	Psat1
5,48E-24	-0,36045	0,119	0,33	6,79E-20	0	Dtx1
5,66E-24	-0,4572	0,367	0,586	7,01E-20	0	Erh
5,71E-24	-0,40168	0,696	0,84	7,07E-20	0	Srsf3
6,57E-24	-0,40956	0,204	0,434	8,14E-20	0	Atad1

7E-24	-0,40528	0,965	0,977	8,67E-20	0	Hspa8
7,1E-24	0,384832	0,442	0,23	8,79E-20	0	Gmfg
8,08E-24	0,301988	0,245	0,08	1E-19	0	Gpr174
1,03E-23	-0,43233	0,204	0,417	1,27E-19	0	Bcl7a
1,08E-23	0,410211	0,591	0,378	1,34E-19	0	Psap
1,09E-23	-0,42325	0,67	0,812	1,35E-19	0	Capzb
1,47E-23	-0,45226	0,038	0,201	1,82E-19	0	Cenpe
1,97E-23	0,359579	0,328	0,14	2,43E-19	0	Phf1
2,02E-23	-0,4145	0,441	0,672	2,5E-19	0	Usp7
2,05E-23	0,405758	0,509	0,291	2,54E-19	0	Mllt6
2,15E-23	-0,38092	0,084	0,272	2,66E-19	0	H2afx
3E-23	-0,45595	0,981	0,981	3,72E-19	0	Laptm5
3,1E-23	-0,36357	0,193	0,417	3,84E-19	0	Fas
3,17E-23	-0,45893	0,451	0,654	3,92E-19	0	Mdh1
3,48E-23	0,449706	0,907	0,823	4,3E-19	0	Eif4a2
3,55E-23	-0,37017	0,228	0,457	4,4E-19	0	Ube2n
4,67E-23	-0,43487	0,939	0,968	5,78E-19	0	Pfn1
5,05E-23	-0,4254	0,374	0,605	6,25E-19	0	Syvn1
5,07E-23	0,327274	0,283	0,108	6,28E-19	0	Cd97
6,09E-23	0,28821	0,242	0,083	7,54E-19	0	Ier2
6,75E-23	-0,31662	0,046	0,215	8,36E-19	0	Smco4
7,35E-23	-0,46257	0,113	0,303	9,1E-19	0	Mcm4
7,6E-23	-0,41166	0,577	0,771	9,41E-19	0	Tnfaip8
7,76E-23	-0,37474	0,357	0,597	9,6E-19	0	Shfm1
1,05E-22	-0,37998	0,093	0,281	1,3E-18	0	Ezh2
1,15E-22	-0,39059	0,175	0,389	1,42E-18	0	Whsc1
1,18E-22	-0,25413	0,004	0,138	1,46E-18	0	Ncapg
1,7E-22	-0,38004	0,155	0,365	2,1E-18	0	Eif2ak3
1,76E-22	-0,40368	0,213	0,436	2,18E-18	0	Atp8a1
1,78E-22	-0,31309	0,035	0,193	2,2E-18	0	Dtl
1,89E-22	-0,34973	0,101	0,294	2,34E-18	0	Ildr1
1,91E-22	-0,35268	0,358	0,6	2,37E-18	0	Cct5
1,92E-22	-0,30705	0,091	0,283	2,38E-18	0	4833439L19Rik
2,55E-22	0,302995	0,249	0,089	3,16E-18	0	Setd1b
2,57E-22	0,35138	0,307	0,129	3,18E-18	0	Sema4b
2,61E-22	0,282616	0,197	0,057	3,23E-18	0	Peg13
2,89E-22	-0,37037	0,403	0,628	3,57E-18	0	Eif3k
2,95E-22	0,396418	0,261	0,097	3,65E-18	0	Kbtbd11
3,16E-22	-0,37473	0,242	0,466	3,91E-18	0	Vdac3
4,03E-22	0,525927	0,626	0,463	4,99E-18	0	Flna
4,3E-22	-0,69719	0,133	0,322	5,32E-18	0	Nrgn
4,64E-22	-0,39046	0,387	0,611	5,75E-18	0	Lpxn
4,78E-22	-0,33049	0,13	0,33	5,92E-18	0	Nudt21
5,36E-22	-0,38842	0,299	0,524	6,63E-18	0	Azin1
6,45E-22	0,3172	0,223	0,073	7,99E-18	0	Plbd1
7,2E-22	0,286505	0,148	0,031	8,91E-18	0	Fam101b
1,02E-21	-0,28374	0,08	0,262	1,26E-17	0	Gng12
1,03E-21	-0,41549	0,214	0,432	1,27E-17	0	Snrpd1
1,04E-21	0,449471	0,372	0,186	1,29E-17	0	Pecam1
1,04E-21	-0,38535	0,287	0,512	1,29E-17	0	Phf6

1,09E-21	0,262608	1	0,999	1,35E-17	0	Rpl13a
1,16E-21	-0,41654	0,706	0,842	1,44E-17	0	Spib
1,26E-21	0,260622	0,206	0,063	1,56E-17	0	Gm15987
1,44E-21	0,459149	0,493	0,307	1,78E-17	0	Ppp3ca
1,5E-21	-0,38419	0,37	0,59	1,86E-17	0	Cox5b
1,58E-21	-0,42351	0,362	0,584	1,95E-17	0	Pgam1
1,62E-21	-0,38619	0,3	0,528	2E-17	0	Cerk
1,86E-21	0,311331	0,268	0,105	2,3E-17	0	Pdcd4
1,87E-21	-0,3469	0,025	0,169	2,32E-17	0	Cdk1
2,1E-21	-0,32468	0,174	0,387	2,6E-17	0	Eif1ax
2,18E-21	-0,30779	0,019	0,159	2,7E-17	0	Cks1b
2,19E-21	-0,38955	0,394	0,634	2,72E-17	0	Atp5j
2,76E-21	-0,35588	0,217	0,433	3,42E-17	0	Pkig
3,25E-21	-0,40416	0,332	0,537	4,02E-17	0	Eif4a1
4,5E-21	-0,35709	0,219	0,435	5,57E-17	0	Arl5a
6,56E-21	0,365463	0,328	0,15	8,12E-17	0	Lgals9
7,23E-21	-0,35461	0,241	0,452	8,95E-17	0	Sdhb
8,84E-21	-0,48506	0,514	0,705	1,09E-16	0	Crip1
1,33E-20	-0,33804	0,33	0,555	1,65E-16	0	Gnb1
1,4E-20	-0,39341	0,204	0,409	1,74E-16	0	H2afv
1,5E-20	-0,35327	0,117	0,304	1,85E-16	0	Slamf7
1,5E-20	0,340191	0,239	0,09	1,86E-16	0	Pxdc1
1,74E-20	-0,39352	0,342	0,559	2,15E-16	0	Psma2
1,94E-20	-0,37618	0,204	0,408	2,4E-16	0	Bid
2,3E-20	0,405454	0,781	0,685	2,84E-16	0	Gm9846
2,47E-20	-0,36938	0,441	0,648	3,06E-16	0	Akr1a1
2,76E-20	-0,34428	0,186	0,389	3,41E-16	0	Naa40
3,47E-20	0,362354	0,343	0,168	4,29E-16	0	Clec2g
3,47E-20	-0,35716	0,345	0,568	4,3E-16	0	Arpc3
4,43E-20	-0,36742	0,301	0,512	5,49E-16	0	Cdk2ap2
4,48E-20	-0,36472	0,458	0,644	5,55E-16	0	Oaz1
4,63E-20	-0,35065	0,329	0,546	5,73E-16	0	Cox5a
5,28E-20	-0,34091	0,187	0,387	6,53E-16	0	Cbx5
5,92E-20	0,383306	0,378	0,193	7,33E-16	0	Emp3
6,13E-20	-0,43438	0,478	0,673	7,59E-16	0	Gna13
8,16E-20	0,27392	0,135	0,028	1,01E-15	0	Gpr82
9,08E-20	-0,40671	0,193	0,388	1,12E-15	0	Dnmt1
9,82E-20	-0,27988	0,068	0,233	1,22E-15	0	Tipin
1,1E-19	-0,35655	0,083	0,248	1,36E-15	0	Pla2g12a
1,14E-19	0,386655	0,358	0,182	1,41E-15	0	Mgat4a
1,2E-19	-0,30683	0,091	0,263	1,49E-15	0	Lst1
1,3E-19	0,428144	0,767	0,663	1,6E-15	0	Smg1
1,33E-19	-0,30376	0,997	0,998	1,64E-15	0	Rplp0
1,83E-19	-0,30954	0,306	0,531	2,26E-15	0	Psma1
1,91E-19	-0,30973	0,054	0,205	2,37E-15	0	Hells
1,99E-19	0,349443	0,339	0,166	2,46E-15	0	Neat1
2,32E-19	-0,33703	0,343	0,571	2,87E-15	0	1700021K19Rik
2,43E-19	0,447548	0,5	0,326	3,01E-15	0	Chd7
3,2E-19	-0,25543	0,052	0,205	3,96E-15	0	2510009E07Rik
3,65E-19	0,415655	0,709	0,563	4,52E-15	0	Gimap1

3,86E-19	-0,34227	0,219	0,417	4,78E-15	0	Rps27l
4,83E-19	-0,28895	0,091	0,259	5,98E-15	0	Msh6
5,41E-19	0,305204	0,362	0,181	6,69E-15	0	Notch2
5,43E-19	-0,41654	0,209	0,398	6,72E-15	0	Lck
5,98E-19	0,343251	0,962	0,921	7,4E-15	0	Rpl36
6,51E-19	-0,34376	0,417	0,626	8,06E-15	0	Snrpe
7,06E-19	-0,32568	0,193	0,393	8,74E-15	0	Cpsf2
7,7E-19	-0,38649	0,601	0,752	9,54E-15	0	Sumo2
8,33E-19	-0,29263	0,135	0,316	1,03E-14	0	Plxnb2
9,11E-19	-0,33978	0,812	0,901	1,13E-14	0	Arpc1b
1,14E-18	0,418254	0,709	0,576	1,41E-14	0	Snx2
1,28E-18	0,442985	0,436	0,263	1,59E-14	0	Rasgrp2
1,45E-18	-0,38084	0,297	0,498	1,79E-14	0	Mapre2
1,47E-18	-0,38955	0,3	0,506	1,82E-14	0	Ube2j1
1,49E-18	-0,31113	0,167	0,36	1,84E-14	0	Eif2a
1,55E-18	-0,29057	0,099	0,267	1,92E-14	0	Abr
1,59E-18	-0,33151	0,106	0,277	1,97E-14	0	Mad2l1
1,84E-18	0,268836	0,191	0,064	2,28E-14	0	Rhbdf1
2,03E-18	0,284145	0,264	0,111	2,52E-14	0	Bhlhe41
2,04E-18	-0,38183	0,472	0,667	2,52E-14	0	Hnrnpa0
2,12E-18	-0,27013	1	0,999	2,62E-14	0	Rpl41
2,19E-18	-0,36523	0,167	0,351	2,72E-14	0	Tgfbr1
2,21E-18	-0,35666	0,532	0,732	2,73E-14	0	Eif3h
2,38E-18	-0,26441	0,071	0,226	2,95E-14	0	Lmnb1
2,38E-18	-0,34797	0,206	0,395	2,95E-14	0	Topbp1
2,56E-18	-0,33184	0,142	0,323	3,17E-14	0	Emid1
2,81E-18	-0,36956	0,232	0,424	3,48E-14	0	Sh3bgrl
2,82E-18	-0,39134	0,567	0,726	3,49E-14	0	Cox6a1
2,86E-18	-0,36882	0,02	0,143	3,54E-14	0	Ccna2
2,89E-18	0,383561	0,251	0,104	3,58E-14	0	A630033H20Rik
2,91E-18	-0,25276	0,055	0,202	3,6E-14	0	Itgb3
2,96E-18	-0,39432	0,094	0,254	3,66E-14	0	Lig1
3,12E-18	0,545361	0,835	0,712	3,87E-14	0	Il2rg
3,21E-18	-0,36786	0,461	0,671	3,98E-14	0	Lrmp
3,39E-18	0,509094	0,287	0,134	4,2E-14	0	Ms4a4c
4,3E-18	-0,34836	0,355	0,559	5,32E-14	0	Sec61b
4,86E-18	-0,29487	0,026	0,153	6,02E-14	0	Cdca3
5,09E-18	-0,31958	0,294	0,491	6,3E-14	0	Lsm4
5,77E-18	-0,33211	0,301	0,507	7,14E-14	0	Pomp
6,26E-18	-0,28573	0,125	0,3	7,75E-14	0	Mrps7
6,26E-18	0,398389	0,82	0,708	7,76E-14	0	Hmha1
6,97E-18	0,356954	0,252	0,108	8,63E-14	0	Satb1
9,54E-18	0,467857	0,691	0,567	1,18E-13	0	Mll5
1,39E-17	-0,33642	0,454	0,653	1,72E-13	0	Hn1
1,72E-17	0,365889	0,407	0,237	2,13E-13	0	Gimap7
1,92E-17	-0,26295	0,072	0,223	2,37E-13	0	Rnaseh2b
1,93E-17	-0,31531	0,252	0,447	2,4E-13	0	Tkt
2E-17	-0,31679	0,314	0,521	2,47E-13	0	Csnk2b
2,07E-17	-0,32613	0,354	0,561	2,57E-13	0	Minos1
2,08E-17	-0,25145	0,042	0,176	2,57E-13	0	Mthfd2

2,13E-17	0,255871	0,21	0,078	2,64E-13	0	Ski
2,5E-17	0,318141	0,229	0,092	3,1E-13	0	Arap2
3,68E-17	-0,29964	0,057	0,196	4,55E-13	0	Idi1
4,37E-17	-0,41449	0,67	0,792	5,41E-13	0	Bptf
4,5E-17	-0,31724	0,228	0,425	5,57E-13	0	Smarca4
4,55E-17	0,342519	0,445	0,275	5,64E-13	0	Rabac1
4,58E-17	0,355952	0,839	0,728	5,67E-13	0	Prkcb
4,73E-17	-0,33166	0,546	0,722	5,86E-13	0	Epn1
4,8E-17	-0,31797	0,416	0,614	5,94E-13	0	Tomm20
5,02E-17	-0,27044	0,245	0,446	6,22E-13	0	Ktn1
5,09E-17	-0,27004	0,093	0,249	6,3E-13	0	Dusp10
5,31E-17	-0,30756	0,23	0,421	6,58E-13	0	Mrpl18
5,4E-17	0,307695	0,268	0,123	6,69E-13	0	Gm2a
6,36E-17	0,422244	0,73	0,589	7,87E-13	0	Lbh
6,78E-17	-0,31658	0,228	0,417	8,4E-13	0	Psma5
6,86E-17	-0,29366	0,091	0,246	8,49E-13	0	Btnl2
6,9E-17	-0,48075	0,107	0,26	8,54E-13	0	Eif5a2
6,91E-17	0,323823	0,309	0,155	8,56E-13	0	Tcp11l2
7,15E-17	-0,34259	0,222	0,409	8,86E-13	0	Trim59
7,26E-17	-0,30072	0,361	0,572	8,98E-13	0	Atp5f1
7,39E-17	-0,39167	0,4	0,583	9,15E-13	0	Cycs
7,81E-17	-0,26554	0,151	0,324	9,67E-13	0	Uchl3
1,1E-16	-0,30668	0,291	0,486	1,36E-12	0	Rbm38
1,12E-16	0,408645	0,533	0,366	1,38E-12	0	Ncf1
1,17E-16	-0,32879	0,396	0,589	1,45E-12	0	Nedd8
1,47E-16	-0,33621	0,359	0,55	1,83E-12	0	Lbr
1,81E-16	-0,28376	0,562	0,749	2,24E-12	0	Atp5c1
1,91E-16	-0,32408	0,583	0,755	2,36E-12	0	Hnrnpk
2,14E-16	-0,4303	0,22	0,391	2,65E-12	0	Mcm7
2,22E-16	-0,47784	0,103	0,253	2,75E-12	0	Rrm2
2,25E-16	-0,28989	0,174	0,35	2,79E-12	0	Arpc1a
2,3E-16	0,349187	0,196	0,074	2,85E-12	0	Osbpl5
2,36E-16	-0,36005	0,391	0,583	2,92E-12	0	Eif2s2
2,37E-16	0,252076	0,155	0,048	2,94E-12	0	Hopx
2,49E-16	-0,3708	0,768	0,84	3,08E-12	0	Hnrnpa3
2,62E-16	-0,30968	0,268	0,461	3,24E-12	0	Cxcr4
2,97E-16	-0,35755	0,375	0,569	3,68E-12	0	Pitpnc1
3,25E-16	0,355061	0,399	0,236	4,03E-12	0	Tmem173
3,37E-16	-0,33631	0,1	0,252	4,18E-12	0	Prim1
3,42E-16	0,328138	0,503	0,328	4,23E-12	0	2310034O05Rik
3,82E-16	-0,35853	0,293	0,481	4,73E-12	0	Ndufc2
4,03E-16	-0,30285	0,446	0,638	4,98E-12	0	Hnrnpd
4,44E-16	0,42906	0,872	0,803	5,5E-12	0	Lax1
4,85E-16	-0,2783	0,029	0,146	6,01E-12	0	Cdca8
5,25E-16	-0,28681	0,232	0,417	6,5E-12	0	Ndufs2
5,7E-16	0,348473	0,528	0,358	7,06E-12	0	S100a11
6,16E-16	-0,30995	0,641	0,801	7,63E-12	0	Serf2
6,35E-16	-0,31694	0,393	0,578	7,86E-12	0	Snrpb
6,51E-16	0,252658	0,142	0,041	8,06E-12	0	Mfhas1
6,55E-16	-0,36643	0,328	0,509	8,11E-12	0	Akt1

6,71E-16	0,251519	0,994	0,987	8,31E-12	0	Rps14
6,87E-16	-0,26732	0,168	0,341	8,5E-12	0	Ahcy
6,9E-16	-0,30286	0,217	0,398	8,54E-12	0	Dynlt3
7,69E-16	-0,27468	0,135	0,296	9,52E-12	0	Ranbp1
9,69E-16	-0,30067	0,294	0,481	1,2E-11	0	Sfpi1
9,94E-16	-0,30302	0,112	0,264	1,23E-11	0	Osbpl3
1,13E-15	-0,28974	0,317	0,513	1,4E-11	0	Ube2k
1,19E-15	-0,28275	0,307	0,502	1,47E-11	0	Dynlrb1
1,22E-15	-0,31397	0,37	0,57	1,51E-11	0	Hprt
1,35E-15	-0,27308	0,28	0,481	1,67E-11	0	Abracl
1,36E-15	-0,28324	0,11	0,263	1,69E-11	0	Rfc3
1,58E-15	0,375878	0,81	0,724	1,96E-11	0	Hvcn1
1,66E-15	-0,34487	0,565	0,733	2,05E-11	0	Pcbp1
1,8E-15	-0,30985	0,433	0,629	2,23E-11	0	Clta
1,82E-15	-0,28247	0,91	0,942	2,25E-11	0	Psmb8
1,86E-15	0,405668	0,519	0,364	2,3E-11	0	Mll3
1,9E-15	-0,35788	0,72	0,826	2,35E-11	0	Ccdc50
1,95E-15	0,265374	0,242	0,107	2,42E-11	0	Ddx58
1,96E-15	-0,2763	0,139	0,301	2,43E-11	0	Nasp
2,11E-15	-0,30788	0,151	0,311	2,62E-11	0	Hat1
2,14E-15	0,30946	0,375	0,21	2,65E-11	0	Fgd2
2,18E-15	-0,30143	0,486	0,688	2,69E-11	0	Map4k1
2,19E-15	-0,33074	0,807	0,881	2,71E-11	0	Arpc4
2,24E-15	0,276589	0,307	0,158	2,77E-11	0	Tle3
2,25E-15	-0,31409	0,13	0,285	2,78E-11	0	Ncapd2
2,43E-15	-0,28142	0,057	0,185	3,01E-11	0	Racgap1
2,54E-15	-0,35025	0,459	0,638	3,15E-11	0	Cyfip2
3,04E-15	-0,32069	0,542	0,707	3,76E-11	0	Cox4i1
3,23E-15	-0,29999	0,325	0,518	4E-11	0	Hipk3
3,94E-15	-0,41512	0,057	0,182	4,88E-11	0	Ccnb2
4,69E-15	-0,28801	0,254	0,435	5,81E-11	0	Usmg5
4,7E-15	-0,31463	0,525	0,709	5,82E-11	0	Ikzf1
5,34E-15	-0,36762	0,187	0,356	6,61E-11	0	Lacc1
5,48E-15	-0,27971	0,233	0,41	6,79E-11	0	Trim35
5,69E-15	-0,41779	0,384	0,555	7,04E-11	0	Wdr92
6,07E-15	-0,36352	0,417	0,604	7,51E-11	0	Cs
6,07E-15	-0,33943	0,401	0,591	7,52E-11	0	Tceb2
6,31E-15	0,372487	0,455	0,3	7,81E-11	0	Ankrd44
6,75E-15	-0,32789	0,287	0,46	8,35E-11	0	Aldh2
6,96E-15	-0,33684	0,29	0,472	8,62E-11	0	Edem1
7,09E-15	-0,31628	0,393	0,58	8,78E-11	0	Lsm14a
8,49E-15	-0,27807	0,232	0,413	1,05E-10	0	Dars
8,64E-15	-0,29418	0,036	0,149	1,07E-10	0	Clspn
8,85E-15	-0,29205	0,68	0,832	1,1E-10	0	Tma7
9,4E-15	-0,32555	0,252	0,428	1,16E-10	0	Rraga
9,56E-15	-0,25069	0,341	0,549	1,18E-10	0	Cbfb
1,03E-14	-0,26507	0,084	0,222	1,28E-10	0	Fen1
1,31E-14	-0,34032	0,662	0,785	1,63E-10	0	Dynll1
1,4E-14	-0,31788	0,948	0,965	1,73E-10	0	Hsp90ab1
1,45E-14	-0,29132	0,362	0,542	1,8E-10	0	Rbbp4

1,54E-14	-0,27378	0,243	0,423	1,91E-10	0	Rfc2
1,69E-14	-0,31554	0,5	0,681	2,09E-10	0	Cct2
1,71E-14	-0,31294	0,438	0,61	2,12E-10	0	Psmb4
1,75E-14	-0,29303	0,254	0,421	2,16E-10	0	Ube2h
1,76E-14	-0,26092	0,307	0,487	2,18E-10	0	Tcp1
1,91E-14	0,418441	0,593	0,458	2,37E-10	0	Ralgps2
1,95E-14	-0,32559	0,449	0,619	2,41E-10	0	Vcp
2,02E-14	-0,27162	0,145	0,3	2,5E-10	0	Aars
2,06E-14	-0,38085	0,809	0,881	2,55E-10	0	Eif5a
2,4E-14	0,316376	0,236	0,107	2,97E-10	0	Cd38
2,54E-14	-0,26859	0,051	0,17	3,14E-10	0	Tcf19
2,71E-14	-0,30601	0,367	0,548	3,36E-10	0	Hspa4
2,88E-14	-0,27058	0,978	0,995	3,57E-10	0	Eif1
2,94E-14	0,377037	0,754	0,638	3,65E-10	0	Pisd-ps1
3,19E-14	-0,28374	0,274	0,447	3,95E-10	0	eGFP
3,37E-14	-0,261	0,293	0,476	4,18E-10	0	Slc25a4
3,4E-14	-0,31068	0,242	0,413	4,21E-10	0	Itgb1
3,87E-14	-0,3011	0,641	0,781	4,79E-10	0	Sfpq
3,92E-14	0,325599	0,499	0,338	4,85E-10	0	Trim34a
4,31E-14	0,348064	0,352	0,208	5,34E-10	0	Smarca2
4,43E-14	-0,40525	0,816	0,884	5,49E-10	0	Cnn2
4,66E-14	-0,27909	0,291	0,461	5,76E-10	0	Ap2s1
5,11E-14	-0,26341	0,78	0,883	6,33E-10	0	Gdi2
5,16E-14	0,339382	0,404	0,259	6,39E-10	0	Malt1
5,59E-14	-0,26461	0,23	0,406	6,92E-10	0	Lsm6
5,86E-14	-0,25562	0,033	0,14	7,25E-10	0	Kif23
8,49E-14	0,353242	0,742	0,649	1,05E-09	0	Srrm2
8,95E-14	-0,27424	0,229	0,397	1,11E-09	0	Fundc2
9,24E-14	-0,25166	0,175	0,332	1,14E-09	0	Mrpl33
9,62E-14	-0,28215	0,222	0,388	1,19E-09	0	Stap1
1,09E-13	-0,29919	0,409	0,575	1,34E-09	0	Eif4h
1,23E-13	-0,25115	0,132	0,275	1,53E-09	0	Dennd3
1,25E-13	0,289018	0,622	0,461	1,55E-09	0	Pyhin1
1,28E-13	-0,27736	0,154	0,306	1,59E-09	0	2700094K13Rik
1,64E-13	-0,25049	0,068	0,19	2,03E-09	0	Birc5
1,65E-13	-0,25995	0,183	0,344	2,04E-09	0	Ehd4
1,69E-13	-0,29426	0,383	0,546	2,09E-09	0	Cbx3
1,69E-13	-0,34403	0,125	0,261	2,09E-09	0	Rrm1
1,84E-13	-0,32268	0,281	0,45	2,28E-09	0	Rev3l
2,06E-13	0,35009	0,352	0,213	2,55E-09	0	Ifngr2
2,12E-13	-0,26672	0,094	0,226	2,63E-09	0	Hmgcs1
2,28E-13	-0,32776	0,796	0,87	2,83E-09	0	Ybx1
2,36E-13	-0,3342	0,787	0,877	2,93E-09	0	Sypl
2,74E-13	-0,3096	0,475	0,636	3,39E-09	0	Ppp2ca
3,05E-13	-0,31133	0,429	0,599	3,78E-09	0	Vdac2
4,18E-13	0,329557	0,788	0,691	5,18E-09	0	Iqgap1
4,32E-13	-0,29241	0,214	0,368	5,35E-09	0	Phf5a
4,33E-13	-0,27586	0,658	0,791	5,36E-09	0	Hnrnpf
4,54E-13	0,261796	0,959	0,94	5,62E-09	0	Rpl36a
4,57E-13	-0,25816	0,343	0,517	5,66E-09	0	Atp5j2

4,69E-13	-0,2609	0,304	0,48	5,8E-09	0	Psmd8
4,92E-13	-0,29483	0,252	0,419	6,09E-09	0	Anapc16
5,87E-13	-0,2596	0,849	0,923	7,27E-09	0	Grb2
6,13E-13	-0,32882	0,18	0,328	7,59E-09	0	Vpreb3
6,45E-13	-0,28313	0,406	0,577	7,98E-09	0	Dbnl
6,72E-13	-0,25837	0,27	0,436	8,32E-09	0	Cdk4
7,22E-13	-0,30126	0,561	0,715	8,94E-09	0	Syk
7,41E-13	-0,31079	0,42	0,575	9,17E-09	0	Paics
8,52E-13	-0,25703	0,243	0,407	1,06E-08	0	Cuta
1,04E-12	-0,26474	0,125	0,262	1,29E-08	0	Pim1
1,17E-12	-0,26924	0,294	0,468	1,44E-08	0	Nme1
1,42E-12	-0,287	0,239	0,396	1,76E-08	0	Brwd1
1,47E-12	0,374094	0,549	0,417	1,82E-08	0	Tlr1
1,51E-12	-0,28013	0,574	0,731	1,87E-08	0	Purb
1,51E-12	0,283842	0,978	0,946	1,87E-08	0	Cd22
1,7E-12	0,296109	0,606	0,472	2,1E-08	0	Nfkbia
1,73E-12	-0,33942	0,377	0,537	2,14E-08	0	Rftn1
1,76E-12	-0,25022	0,168	0,318	2,18E-08	0	Cyb5b
1,87E-12	-0,26895	0,388	0,553	2,32E-08	0	Taldo1
1,98E-12	-0,26608	0,33	0,504	2,45E-08	0	Atp5o
2E-12	0,384242	0,612	0,487	2,48E-08	0	Sfrs18
2,04E-12	-0,25921	0,988	0,988	2,52E-08	0	Coro1a
2,33E-12	0,314662	0,423	0,284	2,89E-08	0	Zmym5
2,45E-12	-0,25005	0,123	0,257	3,03E-08	0	Pqlc3
2,7E-12	-0,29269	0,393	0,562	3,34E-08	0	Syngr2
2,82E-12	-0,2775	0,143	0,283	3,49E-08	0	Nt5e
2,92E-12	-0,26856	0,484	0,649	3,62E-08	0	Tpm4
3,16E-12	0,383206	0,348	0,217	3,91E-08	0	St8sia4
3,16E-12	-0,26071	0,326	0,491	3,92E-08	0	Atp5k
3,22E-12	0,28018	0,359	0,219	3,99E-08	0	Zfp36l2
3,39E-12	0,348666	0,478	0,342	4,19E-08	0	Gns
3,86E-12	-0,26372	0,162	0,303	4,78E-08	0	Ogfrl1
5,5E-12	-0,26789	0,912	0,943	6,81E-08	0	H3f3b
6,02E-12	-0,27367	0,261	0,417	7,45E-08	0	AI662270
6,31E-12	0,308523	0,388	0,252	7,81E-08	0	Pbxip1
7,37E-12	-0,30629	0,8	0,869	9,12E-08	0	Limd2
7,43E-12	-0,27119	0,241	0,388	9,19E-08	0	Dek
7,44E-12	0,345544	0,452	0,319	9,21E-08	0	Cytip
7,73E-12	0,379099	0,412	0,281	9,57E-08	0	Pde4b
8,06E-12	0,25655	0,351	0,218	9,98E-08	0	Arap1
9,66E-12	-0,46014	0,462	0,609	1,2E-07	0	Dusp2
1,01E-11	-0,26231	0,099	0,219	1,25E-07	0	Ngfrap1
1,05E-11	-0,25715	0,681	0,794	1,3E-07	0	Btf3
1,31E-11	-0,28992	0,222	0,372	1,63E-07	0	Lta
1,34E-11	-0,26178	0,652	0,782	1,66E-07	0	Slc25a3
1,39E-11	0,410728	0,32	0,205	1,72E-07	0	Parm1
1,6E-11	-0,33433	0,748	0,814	1,99E-07	0	Trp53i11
1,76E-11	0,280677	0,329	0,198	2,18E-07	0	Ugcg
2,03E-11	-0,33971	0,071	0,181	2,52E-07	0	Egr1
2,06E-11	-0,28679	0,536	0,662	2,55E-07	0	Rhog

2,65E-11	-0,25077	0,303	0,456	3,28E-07	0	Uqcrq
3,06E-11	-0,27812	0,21	0,347	3,79E-07	0	Samd1
3,07E-11	-0,27667	0,206	0,348	3,8E-07	0	Ube2s
3,23E-11	-0,27275	0,699	0,827	4E-07	0	Atp5g2
3,97E-11	-0,2511	0,354	0,514	4,91E-07	0	Uqcrc2
4,86E-11	-0,29147	0,323	0,472	6,01E-07	0	Eif2s1
4,91E-11	0,286462	0,564	0,437	6,08E-07	0	Hsp90b1
5,11E-11	-0,25343	0,181	0,319	6,32E-07	0	Ndufab1
5,36E-11	-0,25569	0,79	0,878	6,63E-07	0	Set
5,4E-11	-0,28621	0,074	0,179	6,68E-07	0	Tpx2
6,55E-11	0,25551	0,267	0,15	8,11E-07	0	Fam120b
6,9E-11	0,312989	0,588	0,466	8,55E-07	0	Cd47
7,57E-11	0,263653	0,284	0,167	9,38E-07	0	Rap1gds1
8,02E-11	0,264527	0,23	0,121	9,93E-07	0	Trim24
9,41E-11	-0,26605	0,393	0,542	1,17E-06	0	Ghitm
9,65E-11	-0,2581	0,362	0,517	1,19E-06	0	Cox17
9,7E-11	0,29418	0,468	0,337	1,2E-06	0	Sptbn1
9,95E-11	-0,27487	0,616	0,741	1,23E-06	0	Plekha2
1,23E-10	0,373678	0,516	0,407	1,52E-06	0	Tgfbr2
1,43E-10	0,288258	0,714	0,621	1,78E-06	0	H2-T23
1,6E-10	0,333432	0,907	0,881	1,98E-06	0	Mef2c
1,69E-10	0,314466	0,617	0,511	2,09E-06	0	Wdr26
2,18E-10	0,268057	0,939	0,904	2,69E-06	0	Ptpn6
2,27E-10	0,331758	0,474	0,355	2,81E-06	0	Rasa3
2,29E-10	0,36677	0,333	0,219	2,84E-06	0	Csnk1g3
2,55E-10	-0,25972	0,652	0,768	3,16E-06	0	Hnrnpu
3,39E-10	-0,2849	0,687	0,785	4,2E-06	0	Sp110
4,25E-10	0,290562	0,378	0,255	5,27E-06	0	4930523C07Rik
4,4E-10	-0,29398	0,096	0,201	5,44E-06	0	Ube2c
4,4E-10	0,314238	0,928	0,854	5,45E-06	0	Mir5109
5,67E-10	-0,27636	0,413	0,558	7,01E-06	0	Cd2ap
5,79E-10	0,270404	0,345	0,222	7,17E-06	0	Zmym2
7,37E-10	0,269973	0,796	0,72	9,13E-06	0	Ltb
7,65E-10	-0,28861	0,359	0,499	9,47E-06	0	Ndufa12
1,26E-09	0,313686	0,439	0,321	1,56E-05	0	Ikbkb
1,28E-09	0,261144	0,326	0,209	1,59E-05	0	Clec2d
1,37E-09	-0,59318	0,726	0,789	1,7E-05	0	Tuba1b
1,79E-09	0,284412	0,319	0,208	2,21E-05	0	Iqsec1
1,91E-09	0,267526	0,422	0,3	2,36E-05	0	Gramd1a
1,95E-09	0,272852	0,9	0,827	2,42E-05	0	Apoe
2,35E-09	0,274748	0,471	0,344	2,91E-05	0	Ctsc
2,47E-09	0,329346	0,461	0,342	3,06E-05	0	Cbfa2t3
2,87E-09	-0,26609	0,51	0,626	3,56E-05	0	Hdac7
3,47E-09	-0,31691	0,448	0,583	4,29E-05	0	Smc4
3,49E-09	0,31136	0,416	0,311	4,32E-05	0	Nxpe3
4,15E-09	0,302012	0,412	0,3	5,14E-05	0	Reep3
4,48E-09	0,329458	0,775	0,728	5,54E-05	0	Bcl11a
5,04E-09	0,274074	0,632	0,542	6,23E-05	0	Tapbp
5,19E-09	0,287929	0,394	0,285	6,43E-05	0	Stat6
5,22E-09	0,289926	0,681	0,584	6,46E-05	0	Tbc1d10c

1,4E-08	0,262854	0,299	0,195	0,000174	0	Ints4
1,41E-08	0,311173	0,47	0,375	0,000175	0	Tnrc6b
1,45E-08	0,281265	0,683	0,597	0,00018	0	Ciita
1,89E-08	0,293368	0,62	0,541	0,000234	0	Arhgap4
2,32E-08	0,30008	0,53	0,436	0,000288	0	Rasal3
2,99E-08	0,282445	0,514	0,429	0,00037	0	Slc44a2
3,3E-08	0,263701	0,728	0,665	0,000409	0	Smap2
3,75E-08	0,263945	0,345	0,242	0,000465	0	Ppp3cc
3,78E-08	0,257353	0,3	0,197	0,000468	0	Hps3
4,74E-08	0,253048	0,359	0,254	0,000586	0	Cbx7
4,9E-08	-0,44756	0,952	0,939	0,000607	0	Ptma
4,97E-08	0,262728	0,665	0,59	0,000616	0	H2-Oa
5,08E-08	0,323614	0,642	0,579	0,000628	0	Ddx3x
5,75E-08	-0,25571	0,123	0,221	0,000712	0	Cenpa
7,77E-08	0,291552	0,457	0,359	0,000962	0	Icosl
7,89E-08	-0,25103	0,172	0,28	0,000977	0	Cdc14b
8,1E-08	0,252753	0,587	0,486	0,001003	0	Foxp1
8,79E-08	-0,27126	0,412	0,537	0,001088	0	Tmpo
1,05E-07	-0,33509	0,42	0,539	0,001298	0	Mif
1,15E-07	0,309916	0,343	0,243	0,001418	0	Trim25
1,7E-07	0,274009	0,588	0,501	0,002101	0	Wdfy4
1,71E-07	0,275191	0,384	0,28	0,002113	0	Ssh2
1,85E-07	0,272819	0,401	0,298	0,002284	0	Zfp217
1,87E-07	0,288105	0,697	0,628	0,002319	0	Blk
1,93E-07	0,279657	0,401	0,3	0,002394	0	Scd2
3,17E-07	-0,25746	0,567	0,678	0,003923	0	Tcof1
3,44E-07	0,273124	0,604	0,532	0,004253	0	Crlf3
4,13E-07	0,264919	0,478	0,392	0,005113	0	Dync1h1
4,33E-07	0,292482	0,439	0,345	0,005364	0	Kif21b
4,35E-07	0,258464	0,794	0,737	0,005389	0	Grk6
5,7E-07	0,256399	0,351	0,256	0,007052	0	Cd84
1,1E-06	0,275751	0,667	0,596	0,013611	0	Clk1
1,25E-06	0,259592	0,401	0,311	0,015516	0	Tax1bp1
1,82E-06	0,285322	0,526	0,44	0,022572	0	March1
1,93E-06	0,266596	0,309	0,223	0,023953	0	Mdn1
2,5E-06	0,300972	0,455	0,382	0,030969	0	Rapgef6
2,93E-06	0,283372	0,436	0,351	0,036243	0	Mia3
3,2E-06	0,28094	0,549	0,482	0,039667	0	Gpr18
3,63E-06	0,331951	0,464	0,385	0,044943	0	Stk24
4,05E-06	0,270387	0,381	0,294	0,050118	0	Cd2
4,52E-06	0,257033	0,506	0,433	0,055984	0	Ppp1r18
4,66E-06	0,258588	0,567	0,487	0,057662	0	Akap8
9,88E-06	-0,25136	0,171	0,256	0,122356	0	Slpi
3,45E-05	0,297812	0,432	0,366	0,42742	0	Trim26
5,13E-05	0,26213	0,53	0,476	0,634717	0	Zmiz2
0,000181	0,284585	0,428	0,373	1	0	Add1
2,1E-139	-1,8579	0,118	0,699	2,6E-135	1	Mndal
3,3E-129	1,146317	0,967	0,566	4,1E-125	1	Basp1
2,2E-128	1,249504	0,957	0,512	2,8E-124	1	Igj
5,2E-121	1,127697	0,759	0,254	6,5E-117	1	S1pr2

4,3E-119	-1,64943	0,198	0,702	5,4E-115	1	Ly6d
1,8E-117	1,084687	0,833	0,311	2,2E-113	1	Mef2b
2E-117	-1,56831	0,135	0,658	2,5E-113	1	Ifi203
2,8E-117	1,088992	0,774	0,272	3,5E-113	1	Bcl6
6E-102	-1,34956	0,701	0,896	7,48E-98	1	Shisa5
1,2E-100	-1,30719	0,445	0,799	1,51E-96	1	Gimap4
3,8E-99	-1,31506	0,092	0,575	4,64E-95	1	Klf2
2,06E-96	-0,8246	0,991	0,998	2,55E-92	1	B2m
3,37E-94	1,036951	0,784	0,356	4,17E-90	1	Aicda
4,29E-94	-1,35277	0,164	0,626	5,32E-90	1	Pml
8,17E-91	0,913663	0,777	0,277	1,01E-86	1	Rgs13
4,79E-90	0,748077	1	0,999	5,93E-86	1	Cfl1
3E-86	-1,46978	0,334	0,708	3,72E-82	1	Cmah
6,82E-85	-1,12675	0,133	0,575	8,44E-81	1	Sub1
7,13E-85	-1,44608	0,139	0,566	8,83E-81	1	Sell
1,6E-81	-1,05878	0,132	0,554	1,98E-77	1	Capg
5,48E-80	-0,97816	0,059	0,478	6,79E-76	1	S100a10
6,39E-77	0,694645	0,515	0,131	7,91E-73	1	Nuggc
3,1E-76	-0,84005	0,963	0,99	3,84E-72	1	H2-K1
1,54E-75	-1,10171	0,059	0,463	1,91E-71	1	Bcl2
4,51E-69	0,742405	0,882	0,588	5,58E-65	1	Arpc5l
3,91E-68	0,817823	0,796	0,453	4,84E-64	1	Tmem131
7,1E-68	0,802094	0,88	0,594	8,79E-64	1	Rhoh
1,98E-67	-1,05729	0,104	0,488	2,45E-63	1	Gm1966
1,34E-66	0,791526	0,669	0,295	1,65E-62	1	Mbd4
1,27E-65	0,706819	0,558	0,189	1,58E-61	1	Eaf2
2,79E-65	0,747692	0,694	0,299	3,46E-61	1	8430410A17Rik
8,43E-63	0,728238	0,775	0,415	1,04E-58	1	Apobec1
1,2E-62	0,556074	0,997	0,984	1,48E-58	1	Ms4a1
4,73E-60	0,477442	0,376	0,08	5,85E-56	1	Neil1
1,06E-57	-0,68027	0,984	0,992	1,31E-53	1	H2-D1
1,22E-57	0,818402	0,611	0,27	1,51E-53	1	Lpp
1,99E-57	-0,90422	0,161	0,514	2,46E-53	1	Snn
4,6E-57	0,642053	0,999	0,972	5,7E-53	1	Laptm5
1,89E-56	0,655766	0,513	0,185	2,34E-52	1	Rassf6
2,91E-55	0,638379	0,552	0,214	3,6E-51	1	Lpin2
7,72E-55	0,662768	0,632	0,29	9,56E-51	1	Dcaf12
1,34E-54	0,698227	0,524	0,194	1,66E-50	1	Rgs2
1,5E-54	-0,85506	0,124	0,46	1,86E-50	1	Map3k1
1,9E-54	0,645972	0,488	0,172	2,35E-50	1	Lipc
3,31E-54	-0,77998	0,036	0,35	4,1E-50	1	Gpr183
5,28E-54	0,560145	0,99	0,92	6,54E-50	1	Nap1l1
8,15E-54	0,764388	0,743	0,414	1,01E-49	1	Mtf2
1,03E-53	-0,77468	0,037	0,349	1,27E-49	1	Cd55
4,88E-53	0,500949	0,388	0,098	6,04E-49	1	Mybl1
2,45E-52	0,739134	0,648	0,309	3,04E-48	1	Gcsam
1,87E-51	-0,6907	0,061	0,372	2,31E-47	1	Itgb7
7,02E-51	0,529923	0,981	0,944	8,69E-47	1	Myl6
5,14E-50	0,637887	0,756	0,428	6,37E-46	1	Ppp4r2
1,16E-49	-0,51968	0,997	1	1,43E-45	1	Ly6e

1,36E-48	-0,72052	0,03	0,314	1,68E-44	1	Arhgef18
1,38E-48	0,617356	0,55	0,234	1,71E-44	1	Ell3
1,71E-48	0,749567	0,778	0,5	2,11E-44	1	Bach2
4,28E-48	0,570378	0,509	0,197	5,29E-44	1	Dstn
1,35E-47	-0,93526	0,402	0,662	1,68E-43	1	Bank1
1,43E-47	-0,62764	0,022	0,298	1,78E-43	1	Ccr6
4,43E-47	-0,81305	0,209	0,516	5,49E-43	1	Add3
6,27E-47	-0,89161	0,286	0,588	7,77E-43	1	Fam65b
3,91E-46	0,770695	0,664	0,379	4,84E-42	1	Hpse
4,58E-45	-0,38124	1	0,999	5,66E-41	1	Rps24
3,39E-44	-0,81777	0,982	0,967	4,19E-40	1	Malat1
5,28E-44	-0,72168	0,04	0,309	6,53E-40	1	S1pr1
7,99E-44	0,576277	0,945	0,792	9,89E-40	1	Sypl
9,44E-44	-0,77076	0,216	0,509	1,17E-39	1	Anxa6
2,05E-43	-0,78517	0,359	0,626	2,53E-39	1	Gimap3
2,75E-42	-0,67547	0,055	0,327	3,41E-38	1	Zfp318
8,07E-42	-0,71161	0,747	0,847	9,99E-38	1	Btg1
1,32E-41	-0,90286	0,612	0,792	1,64E-37	1	Ly6a
1,57E-41	0,645741	0,74	0,47	1,94E-37	1	Foxo1
2,58E-41	0,418087	0,314	0,079	3,19E-37	1	Efnb1
3,54E-41	0,474331	0,994	0,977	4,38E-37	1	Arpc2
5,39E-41	0,654701	0,793	0,566	6,67E-37	1	Top1
5,4E-41	0,541492	0,874	0,601	6,68E-37	1	Txn1
3,28E-40	0,505756	0,538	0,244	4,07E-36	1	Dap
4,73E-40	0,519976	0,686	0,397	5,86E-36	1	Mtss1
4,98E-40	0,597851	0,858	0,691	6,16E-36	1	Bptf
6,86E-40	0,436607	0,999	0,996	8,49E-36	1	Actg1
8,39E-40	-0,66339	0,09	0,363	1,04E-35	1	Serpinb1a
1,04E-39	0,542745	0,583	0,285	1,29E-35	1	Anxa2
2,75E-39	0,623743	0,802	0,559	3,4E-35	1	Erp44
3,97E-39	0,37567	0,254	0,051	4,92E-35	1	Gm2447
4,22E-39	-0,78326	0,343	0,603	5,22E-35	1	Itm2b
5,16E-39	-0,48907	0,016	0,25	6,39E-35	1	Cd97
8,99E-39	0,497357	0,433	0,168	1,11E-34	1	Emid1
1,79E-38	-0,58885	0,107	0,376	2,22E-34	1	Esyt1
4,57E-38	0,556738	0,84	0,649	5,66E-34	1	Ube2d2a
5,38E-38	0,640249	0,92	0,828	6,66E-34	1	Cnn2
6,17E-38	0,531696	0,794	0,544	7,64E-34	1	Pold4
6,73E-38	-0,59895	0,041	0,283	8,34E-34	1	Scml4
1,12E-37	0,512758	0,404	0,152	1,39E-33	1	Sh2b2
1,17E-37	0,512266	0,593	0,303	1,45E-33	1	Helq
1,42E-37	0,555863	0,692	0,4	1,76E-33	1	Pitpnc1
1,78E-37	-0,62901	0,192	0,463	2,2E-33	1	Cyb561a3
2,23E-37	0,487877	0,629	0,332	2,77E-33	1	Glrx3
3,52E-37	-0,49734	0,047	0,289	4,35E-33	1	Sbk1
7,89E-37	0,524663	0,651	0,349	9,77E-33	1	Dck
9,89E-37	-0,76964	0,036	0,267	1,22E-32	1	Plac8
1,14E-36	-0,55154	0,083	0,337	1,41E-32	1	Cdkn1b
1,37E-36	0,546385	0,768	0,478	1,7E-32	1	Klhl6
5,16E-36	0,469752	0,926	0,726	6,39E-32	1	Pou2af1

1,02E-35	0,406492	0,999	0,988	1,26E-31	1	Cd79b
1,04E-35	0,514229	0,701	0,431	1,29E-31	1	Syvn1
1,46E-35	0,511638	0,642	0,359	1,81E-31	1	Rfc1
1,65E-35	-0,58985	0,142	0,402	2,04E-31	1	Lrrc33
2,11E-35	-0,77845	0,22	0,481	2,61E-31	1	Dgka
3,65E-35	0,413352	0,407	0,157	4,52E-31	1	Ccnd3
4,49E-35	0,478145	0,435	0,184	5,56E-31	1	Havcr1
5,7E-35	0,517684	0,991	0,979	7,06E-31	1	Ucp2
6,02E-35	0,447147	0,354	0,123	7,46E-31	1	Cnst
9,16E-35	0,467507	0,466	0,205	1,13E-30	1	Bzw2
2,4E-34	0,354957	0,287	0,08	2,98E-30	1	2510009E07Rik
2,42E-34	-0,83051	0,346	0,571	3E-30	1	Itga4
2,72E-34	-0,7049	0,439	0,656	3,37E-30	1	Gimap6
8,26E-34	0,416658	0,293	0,086	1,02E-29	1	Ccdc17
1,02E-33	0,517325	0,833	0,635	1,27E-29	1	Tnfaip8
1,3E-33	-0,71622	0,763	0,855	1,61E-29	1	Macf1
1,34E-33	0,438665	0,447	0,196	1,66E-29	1	Mtmr14
1,42E-33	-0,68342	0,226	0,489	1,76E-29	1	B3gnt5
3,25E-33	-0,52884	0,09	0,331	4,02E-29	1	Itpr1
4,53E-33	-0,71075	0,293	0,543	5,6E-29	1	Evi2b
8,63E-33	0,328396	0,256	0,065	1,07E-28	1	Gnb4
1,11E-32	0,60351	0,704	0,49	1,38E-28	1	Scaf11
1,65E-32	-0,59698	0,111	0,351	2,04E-28	1	Fgd2
3,83E-32	-0,48646	0,021	0,222	4,74E-28	1	Cd38
5,5E-32	0,489831	0,895	0,733	6,81E-28	1	Ccdc50
7,72E-32	0,446784	0,391	0,159	9,55E-28	1	Cecr2
1,02E-31	0,472576	0,53	0,267	1,26E-27	1	Atp8a1
1,12E-31	-0,55881	0,111	0,349	1,38E-27	1	Man1a
2,1E-31	0,400119	0,377	0,147	2,6E-27	1	Ildr1
6,92E-31	-0,46345	0,092	0,33	8,57E-27	1	Hhex
8,1E-31	0,369774	0,346	0,128	1E-26	1	Lst1
1,07E-30	0,321187	0,188	0,034	1,32E-26	1	Cacna1h
1,37E-30	0,524737	0,882	0,733	1,69E-26	1	Mzb1
2,66E-30	0,471325	0,5	0,257	3,29E-26	1	Brwd1
4,31E-30	0,47363	0,917	0,807	5,34E-26	1	Limd2
4,52E-30	0,413773	0,33	0,121	5,6E-26	1	Endou
6,03E-30	-0,46003	0,018	0,205	7,47E-26	1	Ahnak
6,42E-30	-0,6946	0,731	0,827	7,95E-26	1	Mycbp2
8,32E-30	0,348576	0,226	0,057	1,03E-25	1	Pdzd2
1,07E-29	-0,61749	0,064	0,274	1,32E-25	1	Irgm1
1,12E-29	0,437546	0,506	0,257	1,39E-25	1	Sema7a
1,25E-29	-0,58183	0,308	0,53	1,54E-25	1	Psap
1,83E-29	-0,51983	0,234	0,471	2,27E-25	1	2310034O05Rik
3,38E-29	-0,60376	0,226	0,466	4,19E-25	1	Clic4
4,49E-29	-0,37631	0,01	0,187	5,56E-25	1	Plbd1
5,1E-29	0,393425	0,297	0,102	6,31E-25	1	Cpne5
5,38E-29	0,441564	0,874	0,735	6,66E-25	1	Clic1
7,09E-29	0,397074	0,358	0,142	8,78E-25	1	Rgs10
8,87E-29	-0,42286	0,027	0,214	1,1E-24	1	Ckap4
1,32E-28	-0,57632	0,179	0,407	1,64E-24	1	Ier5

1,37E-28	0,453361	0,475	0,237	1,69E-24	1	Lta
1,69E-28	-0,44645	0,038	0,231	2,09E-24	1	Bhlhe41
2,34E-28	-0,72002	0,336	0,559	2,9E-24	1	Dock10
2,53E-28	0,408561	0,808	0,544	3,14E-24	1	Hmgn1
4,56E-28	-0,4705	0,101	0,32	5,64E-24	1	Notch2
5,26E-28	0,419649	0,485	0,245	6,51E-24	1	Synrg
6,06E-28	-0,50579	0,203	0,435	7,51E-24	1	Myl12b
6,87E-28	-0,59827	0,59	0,729	8,5E-24	1	Scd1
1,42E-27	0,344705	0,365	0,151	1,75E-23	1	Dennd3
1,5E-27	0,462582	0,889	0,744	1,85E-23	1	Spib
2,09E-27	-0,41477	0,025	0,204	2,59E-23	1	Kctd14
2,51E-27	-0,38837	0,028	0,21	3,11E-23	1	Cnn3
2,72E-27	-0,37606	0,021	0,196	3,37E-23	1	Ccr7
2,75E-27	0,2625	0,249	0,074	3,4E-23	1	Clip2
2,92E-27	0,418393	0,441	0,212	3,61E-23	1	Eif2ak3
3,15E-27	0,418105	0,362	0,156	3,9E-23	1	Gadd45b
3,6E-27	-0,38309	0,987	0,998	4,45E-23	1	Rps21
3,75E-27	0,465761	0,602	0,367	4,64E-23	1	Cerk
3,75E-27	0,386303	0,908	0,726	4,65E-23	1	Slc25a5
5,18E-27	0,407971	0,32	0,124	6,42E-23	1	Btnl2
5,7E-27	0,380999	0,383	0,166	7,05E-23	1	Fut8
8,75E-27	0,333198	0,996	0,991	1,08E-22	1	Rpl28
8,81E-27	0,379629	0,293	0,107	1,09E-22	1	Stxbp1
1,04E-26	0,512225	0,922	0,849	1,29E-22	1	Stt3b
1,07E-26	0,462463	0,735	0,505	1,32E-22	1	Mbd2
1,71E-26	0,42594	0,916	0,823	2,12E-22	1	Arpc4
1,77E-26	0,420878	0,49	0,259	2,19E-22	1	Fas
2,59E-26	0,430082	0,673	0,458	3,2E-22	1	Lpxn
3,55E-26	0,382933	0,985	0,956	4,4E-22	1	Calm1
4,39E-26	0,430071	0,744	0,51	5,43E-22	1	Usp7
4,52E-26	-0,47928	0,062	0,255	5,59E-22	1	Kctd12
5,08E-26	0,334987	0,297	0,11	6,29E-22	1	Cep164
1E-25	-0,64745	0,349	0,558	1,24E-21	1	Txnip
1,51E-25	0,379293	0,652	0,404	1,87E-21	1	Arpc3
1,51E-25	0,321562	0,306	0,117	1,88E-21	1	Parp8
3,26E-25	0,403214	0,643	0,411	4,04E-21	1	1700021K19Rik
3,29E-25	0,399393	0,925	0,83	4,07E-21	1	Tnfrsf13c
3,31E-25	0,442694	0,84	0,721	4,09E-21	1	Capzb
3,35E-25	-0,69362	0,737	0,824	4,15E-21	1	Faim3
4,07E-25	0,381085	0,43	0,212	5,03E-21	1	Xrcc1
4,09E-25	-0,40878	0,058	0,244	5,06E-21	1	Il10rb
5,67E-25	0,439826	0,425	0,213	7,02E-21	1	Tgfbr1
6,14E-25	-0,56896	0,219	0,433	7,6E-21	1	Tubb4b
8,64E-25	0,44157	0,484	0,268	1,07E-20	1	Bcl7a
8,91E-25	0,438451	0,744	0,519	1,1E-20	1	Lrmp
1,18E-24	0,477644	0,754	0,577	1,47E-20	1	Crip1
1,31E-24	0,439235	0,601	0,373	1,62E-20	1	Rere
1,39E-24	0,659318	0,697	0,484	1,72E-20	1	Dusp2
2,26E-24	0,443108	0,5	0,281	2,8E-20	1	Pafah1b3
2,93E-24	0,35748	0,333	0,142	3,62E-20	1	Abr

4,2E-24	-0,36431	0,976	0,995	5,2E-20	1	Rpl38
6,38E-24	-0,43703	0,043	0,214	7,9E-20	1	Kbtbd11
6,94E-24	-0,42133	0,01	0,158	8,59E-20	1	Sspn
9,43E-24	0,410317	0,426	0,215	1,17E-19	1	Ehd4
1,13E-23	-0,41264	0,03	0,191	1,4E-19	1	Myo1f
1,3E-23	-0,7629	0,391	0,571	1,61E-19	1	Vim
1,59E-23	0,374691	0,688	0,444	1,97E-19	1	Marcksl1
2,18E-23	-0,6285	0,536	0,693	2,69E-19	1	B4galnt1
2,28E-23	-0,50288	0,342	0,555	2,82E-19	1	Hsp90b1
2,46E-23	-0,43646	0,043	0,211	3,05E-19	1	Marcks
2,47E-23	-0,5251	0,268	0,47	3,06E-19	1	Ubl3
2,56E-23	0,37719	0,93	0,837	3,17E-19	1	Arpc1b
3,44E-23	-0,31535	0,03	0,19	4,26E-19	1	Fxyd5
4,64E-23	0,250031	1	1	5,74E-19	1	Actb
4,98E-23	-0,34081	0,019	0,17	6,17E-19	1	Tmem154
5,56E-23	0,297277	0,997	0,98	6,88E-19	1	Fth1
6,12E-23	-0,33473	0,033	0,194	7,58E-19	1	Gpr174
6,73E-23	0,270454	0,271	0,1	8,34E-19	1	Ada
7,24E-23	0,468277	0,865	0,751	8,96E-19	1	Trp53i11
8,48E-23	-0,53621	0,655	0,778	1,05E-18	1	Stk17b
1,41E-22	-0,55061	0,254	0,459	1,74E-18	1	Stk10
1,86E-22	0,4001	0,639	0,421	2,3E-18	1	Ssbp3
1,97E-22	-0,41465	0,027	0,18	2,44E-18	1	Ccnd2
2,46E-22	0,287574	0,257	0,094	3,05E-18	1	Itgb3
2,48E-22	0,254694	0,141	0,026	3,07E-18	1	Chrna9
2,65E-22	0,273272	0,166	0,04	3,29E-18	1	Usp2
2,77E-22	-0,39128	0,047	0,211	3,43E-18	1	Ddx58
3,29E-22	-0,31926	0,025	0,177	4,08E-18	1	Ski
4,66E-22	0,382593	0,806	0,639	5,77E-18	1	Plekha2
6,69E-22	-0,31649	0,04	0,201	8,29E-18	1	Setd1b
7,51E-22	-0,50756	0,43	0,621	9,29E-18	1	Samd9l
7,65E-22	0,307699	0,234	0,08	9,47E-18	1	Nlrx1
9,1E-22	0,426334	0,707	0,521	1,13E-17	1	Hdac7
9,32E-22	-0,41481	0,127	0,316	1,15E-17	1	2900060B14Rik
9,57E-22	-0,43829	0,132	0,325	1,19E-17	1	Emp3
1,18E-21	0,382415	0,768	0,603	1,46E-17	1	Epn1
1,47E-21	0,402992	0,71	0,517	1,81E-17	1	Tcea1
1,5E-21	0,342833	0,33	0,148	1,86E-17	1	Osbpl3
1,58E-21	0,296711	0,263	0,1	1,96E-17	1	Smco4
1,79E-21	0,407614	0,543	0,33	2,21E-17	1	Odc1
1,99E-21	0,389294	0,568	0,362	2,47E-17	1	Phf6
2,15E-21	0,31042	0,889	0,712	2,67E-17	1	Gapdh
2,35E-21	0,260254	0,266	0,103	2,91E-17	1	Rev1
2,68E-21	0,359278	0,543	0,337	3,32E-17	1	Plekho1
3,21E-21	0,380425	0,49	0,281	3,97E-17	1	Atad1
3,25E-21	0,418226	0,527	0,319	4,02E-17	1	Rev3l
3,33E-21	0,339371	0,401	0,206	4,12E-17	1	Smagp
3,56E-21	-0,53698	0,299	0,491	4,41E-17	1	Ncf1
3,59E-21	-0,42013	0,03	0,177	4,44E-17	1	Serpina3g
3,7E-21	-0,32943	0,012	0,145	4,58E-17	1	Fam46a

3,95E-21	-0,26406	0,018	0,157	4,89E-17	1	Fam43a
4,16E-21	0,321075	0,342	0,159	5,15E-17	1	Rnf41
6,81E-21	-0,4832	0,098	0,273	8,44E-17	1	Lgals9
8,76E-21	-0,58576	0,51	0,652	1,08E-16	1	Lmo2
1,04E-20	0,365564	0,577	0,365	1,29E-16	1	Cdk2ap2
1,58E-20	-0,3855	0,109	0,288	1,96E-16	1	Ppcs
1,84E-20	0,417459	0,426	0,229	2,28E-16	1	Lacc1
2,4E-20	0,343213	0,978	0,947	2,97E-16	1	Pfn1
2,62E-20	-0,43914	0,286	0,488	3,24E-16	1	S100a11
2,86E-20	-0,3714	0,075	0,243	3,54E-16	1	Abcg1
3,1E-20	0,310153	0,331	0,155	3,84E-16	1	4833439L19Rik
3,22E-20	-0,55705	0,339	0,517	3,99E-16	1	Filip1l
3,98E-20	0,394874	0,698	0,507	4,93E-16	1	Polr1d
5,72E-20	0,326877	0,379	0,191	7,08E-16	1	Dtx1
5,99E-20	0,346849	0,509	0,298	7,42E-16	1	Asap1
6,99E-20	-0,3196	0,053	0,208	8,66E-16	1	Arhgef3
7,27E-20	0,322537	0,482	0,278	9E-16	1	Trim35
7,36E-20	0,338668	0,95	0,869	9,11E-16	1	Grb2
9,3E-20	-0,32278	0,016	0,146	1,15E-15	1	Slfn5
1,12E-19	0,340556	0,348	0,172	1,38E-15	1	Ccdc71l
1,32E-19	0,335843	0,988	0,988	1,64E-15	1	Coro1a
1,38E-19	0,323276	0,515	0,314	1,7E-15	1	Scimp
1,74E-19	0,329595	0,377	0,189	2,16E-15	1	Ogfrl1
1,82E-19	-0,33989	0,074	0,238	2,25E-15	1	Evi2a
2,38E-19	0,322959	0,929	0,835	2,94E-15	1	Cd24a
2,65E-19	0,291747	0,365	0,183	3,28E-15	1	1110018G07Rik
2,78E-19	0,261262	0,376	0,187	3,44E-15	1	Plxnb2
3,54E-19	0,373595	0,817	0,642	4,38E-15	1	Pten
3,9E-19	0,352035	0,75	0,561	4,82E-15	1	Cox6b1
4,25E-19	-0,55329	0,405	0,554	5,26E-15	1	Gimap8
5,47E-19	-0,414	0,201	0,384	6,77E-15	1	Tspan32
1E-18	0,329367	0,405	0,218	1,24E-14	1	C130026I21Rik
1,42E-18	-0,39082	0,115	0,285	1,76E-14	1	Neat1
1,77E-18	0,320931	0,88	0,733	2,2E-14	1	Anp32b
1,81E-18	-0,2558	0,027	0,159	2,25E-14	1	Gm15987
1,97E-18	-0,28837	0,062	0,215	2,43E-14	1	Pdcd4
2,28E-18	-0,25419	0,018	0,141	2,82E-14	1	Irak3
2,66E-18	-0,47686	0,266	0,453	3,29E-14	1	Ctsc
2,79E-18	0,320947	0,49	0,29	3,46E-14	1	Arl5a
2,83E-18	0,39718	0,771	0,607	3,51E-14	1	Ppp1ca
2,94E-18	0,354311	0,541	0,352	3,63E-14	1	Rbm38
3,58E-18	0,359142	0,663	0,479	4,43E-14	1	Sema4d
3,94E-18	-0,41804	0,083	0,238	4,87E-14	1	Pydc3
4,96E-18	-0,34255	0,016	0,136	6,14E-14	1	Ptpn22
5,62E-18	0,358645	0,513	0,326	6,96E-14	1	Commd4
5,89E-18	0,319058	0,541	0,347	7,29E-14	1	D930015E06Rik
6,14E-18	0,294678	0,999	0,989	7,6E-14	1	Pabpc1
6,45E-18	0,358276	0,678	0,498	7,98E-14	1	Clta
6,56E-18	0,350674	0,692	0,513	8,12E-14	1	Akr1a1
7,19E-18	-0,28601	0,028	0,156	8,9E-14	1	Spn

7,82E-18	0,415159	0,235	0,093	9,68E-14	1	Egr1
8,15E-18	-0,2848	0,049	0,19	1,01E-13	1	Il27ra
9,2E-18	-0,26175	0,999	0,999	1,14E-13	1	Rpl13
1,46E-17	-0,40481	0,034	0,163	1,8E-13	1	Gbp7
1,59E-17	0,334001	0,635	0,455	1,96E-13	1	Dbnl
1,88E-17	0,355154	0,754	0,586	2,32E-13	1	Ikzf1
2,83E-17	0,344711	0,482	0,299	3,5E-13	1	Ube2h
3,07E-17	-0,44295	0,17	0,338	3,8E-13	1	Chd2
3,81E-17	-0,46891	0,41	0,576	4,71E-13	1	Nfkbia
3,86E-17	0,265146	0,3	0,144	4,78E-13	1	Adam9
4,19E-17	0,275101	0,401	0,226	5,19E-13	1	Abhd17a
5,53E-17	-0,38033	0,136	0,3	6,85E-13	1	Mgat4a
7,56E-17	0,581391	0,359	0,201	9,36E-13	1	Nrgn
8,44E-17	0,311413	0,516	0,328	1,04E-12	1	Cxcr4
8,61E-17	0,277245	0,336	0,172	1,07E-12	1	Bbx
9,42E-17	0,298557	0,283	0,133	1,17E-12	1	Cobl
9,59E-17	0,25139	0,179	0,061	1,19E-12	1	Fam135a
1,02E-16	0,341936	0,865	0,754	1,26E-12	1	Atp5a1
1,24E-16	0,278446	0,361	0,19	1,54E-12	1	Ddx26b
1,39E-16	-0,40714	0,5	0,654	1,72E-12	1	Ddx3x
1,81E-16	0,33889	0,454	0,274	2,24E-12	1	Bid
1,88E-16	0,426086	0,716	0,546	2,33E-12	1	Gna13
1,91E-16	-0,45575	0,219	0,379	2,36E-12	1	Rasgrp2
1,92E-16	0,34718	0,346	0,186	2,37E-12	1	Pik3cg
2,11E-16	-0,5447	0,425	0,567	2,61E-12	1	Pyhin1
2,35E-16	-0,42777	0,777	0,834	2,9E-12	1	Ctss
2,37E-16	-0,33328	0,189	0,365	2,94E-12	1	Gmfg
2,84E-16	0,285558	0,976	0,967	3,51E-12	1	Rac2
3,28E-16	0,292505	0,642	0,445	4,06E-12	1	Shfm1
4,45E-16	-0,31609	0,095	0,243	5,5E-12	1	Sema4b
4,63E-16	-0,2624	0,083	0,228	5,73E-12	1	6030440G07Rik
6,23E-16	0,302262	0,398	0,229	7,72E-12	1	Trib2
6,29E-16	0,357854	0,525	0,346	7,79E-12	1	Edem1
6,62E-16	0,344596	0,512	0,34	8,2E-12	1	Aldh2
8,24E-16	0,30653	0,53	0,355	1,02E-11	1	Sfpi1
8,5E-16	-0,2844	0,033	0,152	1,05E-11	1	Zmynd11
9,66E-16	-0,41092	0,398	0,568	1,2E-11	1	Cd47
9,78E-16	0,276584	0,49	0,313	1,21E-11	1	Dbi
1,11E-15	-0,31635	0,055	0,185	1,38E-11	1	Arap2
1,17E-15	0,28179	0,868	0,718	1,45E-11	1	Atp5b
1,2E-15	0,345365	0,765	0,617	1,48E-11	1	Smim14
1,27E-15	-0,57738	0,97	0,97	1,57E-11	1	Gm17821
1,34E-15	0,306036	0,506	0,328	1,66E-11	1	Pptc7
1,43E-15	-0,30438	0,021	0,13	1,77E-11	1	Fcrl5
1,43E-15	-0,52305	0,095	0,236	1,77E-11	1	Ms4a4c
1,48E-15	0,341736	0,464	0,289	1,83E-11	1	Ehd3
1,66E-15	0,268297	0,268	0,124	2,05E-11	1	H1fx
1,78E-15	0,273855	0,34	0,178	2,2E-11	1	Gsn
2,04E-15	-0,30217	0,973	0,983	2,52E-11	1	Rpl37a
2,45E-15	0,378285	0,584	0,426	3,03E-11	1	Rftn1

2,58E-15	-0,42862	0,223	0,382	3,2E-11	1	Pde4b
2,64E-15	0,373759	0,558	0,386	3,27E-11	1	Akt1
2,71E-15	-0,56192	0,93	0,949	3,36E-11	1	A630089N07Rik
2,82E-15	0,362443	0,703	0,573	3,49E-11	1	Rhog
2,91E-15	0,294292	0,553	0,371	3,6E-11	1	Fam134b
3,2E-15	0,308613	0,621	0,433	3,96E-11	1	Pgk1
3,79E-15	-0,48668	0,747	0,816	4,69E-11	1	Tubb5
4,1E-15	0,298195	0,408	0,241	5,07E-11	1	Akap2
6,55E-15	0,266166	0,389	0,224	8,11E-11	1	Hmgn3
6,73E-15	0,321305	0,763	0,608	8,34E-11	1	Eif3h
7,82E-15	-0,52498	0,919	0,957	9,68E-11	1	Ptma
9,5E-15	-0,3908	0,195	0,35	1,18E-10	1	Gimap7
9,64E-15	-0,41086	0,274	0,424	1,19E-10	1	Ppp3ca
9,66E-15	-0,32167	0,132	0,281	1,2E-10	1	Clec2g
9,69E-15	-0,30232	0,111	0,256	1,2E-10	1	Phf1
9,93E-15	0,317443	0,513	0,348	1,23E-10	1	Tpd52
1,09E-14	0,310653	0,72	0,562	1,35E-10	1	Map4k1
1,15E-14	0,294481	0,571	0,395	1,42E-10	1	Coro1b
1,21E-14	0,365845	0,472	0,31	1,49E-10	1	Rraga
1,38E-14	0,314917	0,672	0,505	1,71E-10	1	Psmb7
1,44E-14	0,300902	0,648	0,478	1,78E-10	1	Pik3c2b
1,51E-14	0,306863	0,669	0,531	1,87E-10	1	Oaz1
1,52E-14	-0,27217	0,996	0,998	1,89E-10	1	Ddx5
1,55E-14	-0,36726	0,263	0,422	1,92E-10	1	Mllt6
1,58E-14	0,306489	0,439	0,272	1,96E-10	1	Stap1
1,6E-14	-0,42531	0,592	0,721	1,98E-10	1	Fam111a
1,91E-14	0,25884	0,322	0,171	2,36E-10	1	Ralgds
2,04E-14	-0,37727	0,152	0,303	2,52E-10	1	Pecam1
2,16E-14	0,28551	0,488	0,316	2,68E-10	1	Ktn1
2,19E-14	0,300125	0,432	0,273	2,71E-10	1	Rnf19b
2,34E-14	-0,45379	0,429	0,568	2,89E-10	1	Flna
2,93E-14	0,302698	0,777	0,623	3,62E-10	1	Purb
2,93E-14	-0,38447	0,568	0,699	3,63E-10	1	Ifi30
2,95E-14	0,326833	0,504	0,329	3,65E-10	1	Pnp
3,03E-14	0,302683	0,673	0,512	3,75E-10	1	Lat2
3,2E-14	0,324066	0,609	0,446	3,97E-10	1	Syngr2
3,93E-14	-0,30071	0,93	0,956	4,87E-10	1	Rpl36a
4,16E-14	0,282776	0,951	0,904	5,15E-10	1	Rbm3
4,62E-14	-0,5039	0,429	0,57	5,72E-10	1	Foxp1
4,94E-14	0,267913	0,497	0,313	6,11E-10	1	2700029M09Rik
5,31E-14	-0,33347	0,071	0,2	6,58E-10	1	A630033H20Rik
5,78E-14	0,286589	0,787	0,629	7,15E-10	1	Atp5c1
5,99E-14	-0,30387	0,118	0,259	7,42E-10	1	Tle3
6,53E-14	0,329743	0,596	0,43	8,09E-10	1	Fermt3
8,08E-14	-0,38479	0,661	0,759	1E-09	1	Iqgap1
1,09E-13	0,304836	0,476	0,31	1,35E-09	1	Tmem64
1,1E-13	0,317254	0,571	0,388	1,36E-09	1	Psip1
1,1E-13	0,330525	0,624	0,456	1,36E-09	1	Lsm14a
1,39E-13	-0,28659	0,099	0,234	1,72E-09	1	Cyfip1
1,43E-13	0,310473	0,757	0,61	1,77E-09	1	Cox6c

1,62E-13	0,294064	0,393	0,239	2E-09	1	Eif2a
1,72E-13	0,305778	0,629	0,464	2,13E-09	1	Nedd8
1,75E-13	0,28964	0,552	0,385	2,16E-09	1	Cmpk1
1,98E-13	-0,26087	0,087	0,217	2,45E-09	1	Casp4
2,1E-13	-0,27543	0,09	0,219	2,6E-09	1	Gm2a
2,24E-13	0,282055	0,459	0,295	2,77E-09	1	Ublcp1
2,27E-13	-0,7089	0,735	0,783	2,82E-09	1	Tuba1b
2,31E-13	0,32488	0,604	0,436	2,86E-09	1	Srpk3
2,31E-13	0,26032	0,337	0,187	2,86E-09	1	Slamf7
2,34E-13	0,29054	0,42	0,264	2,89E-09	1	Naa40
2,6E-13	-0,35765	0,214	0,36	3,22E-09	1	Malt1
2,78E-13	-0,45768	0,296	0,439	3,44E-09	1	Gns
2,82E-13	0,286313	0,393	0,24	3,49E-09	1	Scai
2,9E-13	-0,36573	0,488	0,631	3,59E-09	1	Mat2a
2,9E-13	0,274102	0,484	0,32	3,59E-09	1	Ube2n
3,01E-13	-0,25784	0,089	0,219	3,73E-09	1	S1pr4
3,07E-13	0,258441	0,206	0,091	3,8E-09	1	H1f0
3,34E-13	0,27775	0,426	0,271	4,13E-09	1	Cbx4
3,71E-13	-0,43109	0,309	0,453	4,59E-09	1	Mylip
4,06E-13	-0,36561	0,536	0,656	5,03E-09	1	Gimap1
4,84E-13	-0,27274	0,068	0,189	6E-09	1	Irf4
4,92E-13	-0,3673	0,825	0,872	6,1E-09	1	D4Wsu53e
5,58E-13	0,274036	0,541	0,379	6,91E-09	1	Pomp
7,01E-13	0,288543	0,586	0,422	8,68E-09	1	2410006H16Rik
1,04E-12	0,284069	0,518	0,356	1,29E-08	1	Gabarapl2
1,09E-12	0,252298	0,425	0,266	1,35E-08	1	Sidt2
1,15E-12	-0,32165	0,24	0,385	1,43E-08	1	Rabac1
1,15E-12	0,263313	0,485	0,321	1,43E-08	1	Sdhb
1,21E-12	-0,31625	0,123	0,254	1,5E-08	1	Tcp11l2
1,3E-12	0,260421	0,38	0,23	1,61E-08	1	Sdhaf2
1,36E-12	0,273877	0,834	0,698	1,68E-08	1	Serf2
1,53E-12	-0,39457	0,714	0,795	1,89E-08	1	Prkcb
1,65E-12	-0,32718	0,18	0,323	2,04E-08	1	Tagln2
2,02E-12	0,253548	0,34	0,198	2,5E-08	1	Nxpe2
2,06E-12	-0,42877	0,291	0,432	2,55E-08	1	Cbfa2t3
2,15E-12	0,257325	0,296	0,165	2,66E-08	1	Pqlc3
2,19E-12	0,261303	0,337	0,198	2,71E-08	1	Mprip
2,66E-12	0,260768	0,55	0,388	3,3E-08	1	Ube2k
2,78E-12	0,262615	0,382	0,239	3,44E-08	1	Arpc1a
3,03E-12	0,301677	0,864	0,785	3,75E-08	1	Arpc5
3,12E-12	0,299411	0,584	0,432	3,86E-08	1	Hspa4
3,52E-12	-0,39455	0,555	0,659	4,36E-08	1	Snx2
4,01E-12	0,295927	0,675	0,533	4,97E-08	1	Mif4gd
4,02E-12	-0,33721	0,808	0,876	4,97E-08	1	Eif4a2
4,1E-12	-0,43525	0,308	0,44	5,08E-08	1	Trim34a
4,24E-12	-0,3721	0,241	0,385	5,25E-08	1	Scd2
4,32E-12	-0,25862	0,102	0,226	5,35E-08	1	Tuba4a
4,96E-12	0,276653	0,506	0,346	6,13E-08	1	Ubqln1
5,22E-12	0,27701	0,49	0,329	6,46E-08	1	Il21r
5,69E-12	0,314158	0,537	0,379	7,04E-08	1	Ube2j1

6,14E-12	0,350963	0,29	0,163	7,61E-08	1	Eif5a2
6,9E-12	0,27946	0,991	0,989	8,54E-08	1	Cd53
7,1E-12	0,282471	0,817	0,703	8,8E-08	1	Dynll1
7,27E-12	-0,35342	0,75	0,827	9E-08	1	Ly86
8,27E-12	-0,3454	0,172	0,305	1,02E-07	1	Csnk1g3
9,34E-12	0,30352	0,58	0,436	1,16E-07	1	Parp1
9,92E-12	0,26942	0,509	0,358	1,23E-07	1	Slc25a19
1,04E-11	-0,25166	0,052	0,156	1,29E-07	1	Cd72
1,11E-11	0,253899	0,544	0,398	1,37E-07	1	Yeats4
1,16E-11	0,272785	0,676	0,529	1,44E-07	1	Ppp1r16b
1,63E-11	0,305627	0,525	0,376	2,02E-07	1	Mapre2
1,83E-11	-0,48463	0,695	0,787	2,27E-07	1	Il2rg
1,89E-11	0,262787	0,416	0,274	2,33E-07	1	Cpsf2
2,11E-11	-0,57395	0,743	0,83	2,61E-07	1	Grk4
2,22E-11	-0,2882	0,862	0,91	2,75E-07	1	Eef1g
2,43E-11	-0,45515	0,994	0,991	3,01E-07	1	Kcnq1ot1
2,66E-11	0,254113	0,831	0,714	3,29E-07	1	Btf3
2,74E-11	0,255184	0,642	0,5	3,39E-07	1	Eif3k
3,61E-11	-0,27314	0,247	0,386	4,47E-07	1	Cdc42se2
4,05E-11	0,250842	0,978	0,965	5,02E-07	1	Gnai2
4,15E-11	0,307955	0,602	0,457	5,14E-07	1	Cd2ap
4,45E-11	0,271511	0,512	0,364	5,51E-07	1	Cnot2
5,52E-11	0,27637	0,643	0,497	6,84E-07	1	Gga2
5,53E-11	0,312112	0,441	0,312	6,85E-07	1	Sh3bgrl
5,55E-11	-0,27288	0,917	0,945	6,87E-07	1	Rpl36
6,08E-11	0,363377	0,589	0,446	7,53E-07	1	Wdr92
6,72E-11	0,258937	0,355	0,223	8,32E-07	1	Slamf1
7,18E-11	-0,26496	0,158	0,289	8,89E-07	1	Ugcg
7,78E-11	0,284852	0,586	0,448	9,63E-07	1	Taldo1
8,35E-11	-0,27656	0,074	0,179	1,03E-06	1	Trim30b
8,37E-11	0,261701	0,645	0,505	1,04E-06	1	Cotl1
1,26E-10	0,264777	0,559	0,418	1,55E-06	1	Pik3cd
1,48E-10	0,25446	0,692	0,568	1,83E-06	1	Myl12a
1,63E-10	-0,36587	0,169	0,288	2,01E-06	1	Irf1
1,78E-10	-0,30421	0,297	0,428	2,2E-06	1	Sptbn1
1,95E-10	-0,29473	0,669	0,745	2,41E-06	1	Gm9846
2,06E-10	-0,2531	0,855	0,891	2,55E-06	1	Rpl14
2,14E-10	0,261133	0,53	0,394	2,65E-06	1	Hip1r
2,19E-10	-0,37604	0,315	0,425	2,71E-06	1	Chd7
2,48E-10	-0,30741	0,401	0,512	3,07E-06	1	Acap1
2,48E-10	0,323388	0,624	0,493	3,07E-06	1	Cs
2,76E-10	-0,28463	0,179	0,306	3,42E-06	1	Ifngr2
3,15E-10	0,283692	0,556	0,428	3,9E-06	1	Tspan13
3,69E-10	-0,27772	0,172	0,292	4,56E-06	1	Clec2d
4,33E-10	0,289257	0,737	0,635	5,36E-06	1	Cox6a1
4,66E-10	0,262657	0,849	0,741	5,77E-06	1	Tma7
5,42E-10	0,275326	0,641	0,51	6,72E-06	1	St6gal1
6,15E-10	0,262026	0,41	0,279	7,61E-06	1	Lrch1
9,03E-10	0,261048	0,451	0,313	1,12E-05	1	Anapc16
9,52E-10	-0,31477	0,044	0,129	1,18E-05	1	Ccna2

9,67E-10	0,255343	0,732	0,624	1,2E-05	1	Syk
1,29E-09	0,250457	0,478	0,34	1,6E-05	1	Vdac3
1,41E-09	0,268327	0,575	0,431	1,75E-05	1	Cd86
1,73E-09	0,268115	0,438	0,303	2,14E-05	1	Zfp710
1,96E-09	-0,3363	0,324	0,435	2,43E-05	1	Rasa3
2,23E-09	0,255991	0,79	0,694	2,76E-05	1	Tecr
2,6E-09	-0,3595	0,296	0,403	3,22E-05	1	Cytip
2,99E-09	0,253331	0,679	0,566	3,71E-05	1	Prkcd
3,66E-09	-0,30399	0,216	0,331	4,53E-05	1	Jhdm1d
4,25E-09	-0,30109	0,257	0,372	5,26E-05	1	Zmym5
5,03E-09	-0,37951	0,558	0,638	6,22E-05	1	Mll5
5,06E-09	-0,33289	0,88	0,894	6,27E-05	1	Zfp36l1
5,71E-09	-0,25353	0,118	0,222	7,07E-05	1	Morc3
9,24E-09	-0,5711	0,467	0,548	0,000114	1	Stat1
9,8E-09	-0,25964	0,061	0,146	0,000121	1	Osbpl5
1,07E-08	-0,33473	0,676	0,712	0,000132	1	Smg1
1,56E-08	-0,27369	0,188	0,296	0,000193	1	Smarca2
1,63E-08	0,250746	0,66	0,542	0,000202	1	Mdh1
2,02E-08	-0,27032	0,18	0,287	0,000251	1	Aff3
2,74E-08	-0,30181	0,469	0,565	0,00034	1	Wdfy4
3,41E-08	-0,30459	0,229	0,334	0,000423	1	4930523C07Rik
3,48E-08	-0,27031	0,222	0,33	0,00043	1	Tmem173
4,19E-08	0,257789	0,806	0,721	0,000518	1	Sp110
4,26E-08	-0,25266	0,047	0,123	0,000527	1	Nr1d2
5,15E-08	-0,33013	0,584	0,667	0,000637	1	Lbh
7,06E-08	-0,29573	0,348	0,456	0,000874	1	Mll3
7,65E-08	0,277223	0,524	0,411	0,000947	1	Dync1i2
1,08E-07	-0,27783	0,096	0,183	0,001337	1	Slfn8
1,2E-07	-0,27913	0,512	0,596	0,001485	1	Napsa
1,56E-07	-0,42808	0,379	0,479	0,001935	1	Ifi47
1,56E-07	0,25675	0,54	0,436	0,001937	1	Uvrag
1,62E-07	-0,35797	0,852	0,894	0,00201	1	Mir5109
2,12E-07	-0,29097	0,633	0,708	0,002631	1	Srrm2
2,39E-07	-0,50908	0,59	0,684	0,002953	1	Vmn2r55
2,65E-07	0,264473	0,518	0,397	0,003286	1	Prkacb
2,91E-07	-0,49138	0,605	0,644	0,003607	1	Fcer2a
2,99E-07	-0,34232	0,408	0,493	0,003706	1	Tlr1
3,03E-07	0,259854	0,537	0,432	0,003749	1	Max
3,26E-07	-0,65889	0,175	0,255	0,004036	1	2810417H13Rik
6,08E-07	-0,27767	0,487	0,57	0,00753	1	Sec11c
6,09E-07	-0,26028	0,192	0,285	0,007537	1	Mdn1
6,2E-07	-0,278	0,287	0,378	0,007677	1	Mtdh
1E-06	-0,27753	0,291	0,387	0,012392	1	Ankrd44
1,45E-06	-0,32827	0,191	0,28	0,017952	1	Gbp4
1,77E-06	0,287757	0,286	0,195	0,021887	1	Slpi
1,97E-06	-0,27463	0,293	0,38	0,024379	1	Klf13
2,29E-06	-0,2514	0,528	0,597	0,028328	1	Tapbp
3,28E-06	-0,26201	0,275	0,365	0,040607	1	Zfp217
3,43E-06	-0,33559	0,734	0,784	0,042495	1	Btla
4,02E-06	-0,28078	0,689	0,761	0,049744	1	Cybb

5,73E-06	-0,28089	0,163	0,24	0,07094	1	Rrm1
1,14E-05	-0,25377	0,538	0,616	0,141477	1	Nme2
3,01E-05	-0,25674	0,361	0,432	0,372587	1	Rapgef6
3,65E-05	-0,28394	0,439	0,506	0,451745	1	Abca1
3,9E-05	-0,25218	0,364	0,439	0,482971	1	Stk24
5,08E-05	-0,25827	0,812	0,822	0,629389	1	Hsp90aa1
6,66E-05	-0,30086	0,308	0,382	0,824391	1	Abhd17b
8,15E-05	-0,38186	0,519	0,576	1	1	Gm10785
8,8E-05	-0,3005	0,405	0,47	1	1	Nfkb1
0,000109	-0,34761	0,42	0,484	1	1	A130077B15Rik
0,000135	-0,25295	0,456	0,512	1	1	Tmpo
0,000439	-0,27721	0,303	0,369	1	1	Nfatc1
0,002344	-0,35099	0,172	0,215	1	1	Rrm2
9,5E-153	2,085635	0,82	0,143	1,2E-148	2	2810417H13Rik
7,2E-137	1,527705	0,833	0,16	9E-133	2	Mcm5
2E-124	1,130054	0,657	0,089	2,5E-120	2	Uhrf1
4,4E-120	0,957762	0,584	0,063	5,4E-116	2	Tcf19
8,8E-120	1,179277	0,796	0,158	1,1E-115	2	Mcm3
1,5E-114	1,784441	0,8	0,197	1,8E-110	2	Top2a
1,2E-110	2,241904	0,959	0,563	1,5E-106	2	Pcna
5,6E-110	1,789567	0,906	0,308	7E-106	2	Stmn1
7,7E-109	1,184605	0,682	0,119	9,5E-105	2	Dhfr
1,6E-107	1,498385	0,686	0,131	1,9E-103	2	Rrm2
1,2E-103	0,83423	0,612	0,086	1,5E-99	2	Hells
1,6E-103	1,140532	0,686	0,128	1,9E-99	2	Lig1
3,9E-103	0,551959	0,343	0,012	4,8E-99	2	E2f8
9,2E-102	1,501894	1	0,936	1,14E-97	2	Ptma
7,4E-101	1,079291	0,755	0,171	9,21E-97	2	Dut
2,3E-100	1,027192	0,673	0,125	2,81E-96	2	Cdca7
3,4E-99	1,837016	0,98	0,638	4,18E-95	2	H2afz
3,5E-99	0,521557	0,327	0,011	4,38E-95	2	Kntc1
1,92E-98	0,624174	0,437	0,036	2,38E-94	2	Cdc45
4,68E-98	1,013028	0,747	0,168	5,79E-94	2	Mcm2
6,52E-97	1,568766	0,873	0,326	8,07E-93	2	Mcm6
2,86E-93	1,220828	0,743	0,185	3,55E-89	2	Asf1b
6,58E-91	1,084496	0,714	0,168	8,14E-87	2	Mcm4
9,34E-91	1,611648	0,902	0,398	1,16E-86	2	Hmgb2
1,25E-90	1,801265	0,967	0,738	1,55E-86	2	Tuba1b
2,71E-90	0,654119	0,461	0,05	3,35E-86	2	Mybl2
4,06E-90	0,583114	0,38	0,027	5,03E-86	2	Rad51
2,56E-88	1,082462	0,665	0,149	3,17E-84	2	Rrm1
3,35E-88	0,469651	0,314	0,014	4,15E-84	2	Rad51ap1
2,57E-87	1,314371	0,976	0,58	3,18E-83	2	Ran
7,17E-87	0,529274	0,4	0,034	8,87E-83	2	Chaf1a
5,34E-85	0,531502	0,339	0,021	6,61E-81	2	Cdc6
8,77E-85	0,577281	0,31	0,015	1,09E-80	2	Esco2
4,3E-84	1,186947	0,8	0,264	5,32E-80	2	Mcm7
2,79E-83	0,79883	0,604	0,112	3,46E-79	2	Fen1
1,55E-81	1,603438	0,767	0,25	1,92E-77	2	Mki67
2,98E-81	0,891585	0,457	0,06	3,69E-77	2	Clspn

2,15E-80	0,944315	0,633	0,136	2,67E-76	2	Prim1
1,13E-79	0,728175	0,543	0,093	1,4E-75	2	Gmnn
1,07E-78	0,598469	0,355	0,03	1,32E-74	2	Ncapg2
1,21E-78	0,673877	0,437	0,053	1,49E-74	2	Atad5
1,08E-76	0,465526	0,302	0,018	1,33E-72	2	Mcm10
3,48E-75	0,818119	0,465	0,069	4,31E-71	2	Cdk1
5,08E-75	0,532382	0,359	0,033	6,29E-71	2	Chek1
1,28E-74	0,695723	0,588	0,116	1,59E-70	2	Tipin
3,35E-73	0,82408	0,42	0,054	4,15E-69	2	Ccna2
4,86E-73	0,736384	0,502	0,085	6,02E-69	2	Dtl
2,01E-72	0,591445	0,367	0,038	2,49E-68	2	Brca1
6,44E-71	0,595192	0,437	0,061	7,97E-67	2	Fignl1
1,2E-69	0,556556	0,408	0,054	1,49E-65	2	Orc6
2,43E-69	1,18135	0,955	0,682	3,01E-65	2	Hnrnpab
4,31E-68	0,58534	0,416	0,058	5,34E-64	2	Pole
1,06E-67	1,06471	0,808	0,32	1,31E-63	2	Slbp
1,64E-67	0,643367	0,441	0,068	2,03E-63	2	Ncaph
3,04E-67	0,467786	0,367	0,042	3,77E-63	2	Rfc4
2,13E-65	0,43118	0,282	0,02	2,64E-61	2	Cenpk
4,62E-64	1,098987	0,967	0,705	5,73E-60	2	Hmgn2
1,38E-63	0,94994	0,747	0,259	1,71E-59	2	Dnmt1
1,83E-63	0,710937	0,555	0,125	2,27E-59	2	Rpa2
2,84E-63	0,953579	0,882	0,451	3,51E-59	2	Rbbp7
6,89E-63	0,569176	0,38	0,051	8,52E-59	2	Spc24
1,36E-62	0,393722	0,233	0,012	1,69E-58	2	Pbk
2,32E-62	-1,58134	0,861	0,988	2,87E-58	2	Malat1
3,32E-60	0,883675	0,984	0,903	4,12E-56	2	Ppia
1,34E-59	0,747376	0,645	0,187	1,66E-55	2	Nasp
1,69E-59	0,790889	0,653	0,197	2,09E-55	2	Hat1
3,4E-59	0,493517	0,335	0,041	4,21E-55	2	Tyms
1,39E-57	1,057153	0,955	0,774	1,72E-53	2	Hmgb1
1,06E-56	1,140015	0,947	0,77	1,32E-52	2	Tubb5
1,7E-56	0,56527	0,437	0,081	2,11E-52	2	Pola1
1,81E-56	0,715657	0,629	0,184	2,24E-52	2	Ranbp1
1,02E-55	0,977935	0,882	0,437	1,26E-51	2	Tmpo
3,26E-55	0,8921	0,771	0,32	4,03E-51	2	2700029M09Rik
9,02E-55	0,558674	0,4	0,066	1,12E-50	2	Cdca3
1,07E-54	0,717859	0,588	0,162	1,32E-50	2	Ezh2
2,04E-54	0,486322	0,347	0,049	2,53E-50	2	Plk4
1,28E-53	0,650143	0,392	0,069	1,58E-49	2	Stil
6,31E-53	0,612577	0,457	0,097	7,82E-49	2	Tacc3
8,81E-53	0,547963	0,257	0,024	1,09E-48	2	Neil3
2,01E-52	0,738505	0,506	0,125	2,49E-48	2	Nmral1
1,21E-51	0,849491	0,751	0,299	1,5E-47	2	Snrpd1
3,01E-51	0,302382	0,204	0,012	3,73E-47	2	Rad54l
4,24E-51	0,901423	0,955	0,747	5,25E-47	2	Gapdh
7,58E-51	-1,48375	0,576	0,865	9,38E-47	2	Shisa5
8,34E-51	0,44807	0,294	0,036	1,03E-46	2	Cdca2
1,11E-50	0,323278	0,2	0,012	1,38E-46	2	Cdca5
2,06E-50	0,466418	0,318	0,044	2,55E-46	2	Kif15

2,51E-50	0,480693	0,294	0,037	3,1E-46	2	Cenph
3,08E-50	0,931067	0,873	0,388	3,81E-46	2	Rgs13
3,56E-50	0,589678	0,416	0,084	4,4E-46	2	Gins1
8,07E-50	0,651759	0,563	0,159	9,99E-46	2	Rfc3
8,78E-50	0,307905	0,216	0,016	1,09E-45	2	Exo1
9,04E-50	0,368004	0,286	0,034	1,12E-45	2	Gins2
1,02E-49	0,591997	0,371	0,066	1,27E-45	2	Fbxo5
2,9E-49	0,345886	0,204	0,013	3,59E-45	2	Mxd3
9,04E-49	0,887284	0,951	0,659	1,12E-44	2	Txn1
2,56E-48	0,813986	0,865	0,458	3,17E-44	2	Erh
8,78E-48	0,745799	0,71	0,274	1,09E-43	2	Topbp1
1,18E-47	0,746452	0,735	0,287	1,46E-43	2	Dnajc9
1,39E-47	0,257051	0,167	0,007	1,72E-43	2	E2f7
2,31E-47	0,898964	0,976	0,666	2,86E-43	2	Basp1
3,46E-47	0,881284	0,959	0,798	4,28E-43	2	Hsp90aa1
4E-47	0,830974	0,963	0,75	4,95E-43	2	Srsf2
8,95E-47	0,412865	0,388	0,075	1,11E-42	2	Siva1
1,18E-46	0,672632	0,694	0,263	1,47E-42	2	Cbx5
5,04E-46	0,451775	0,335	0,056	6,24E-42	2	Ncapg
6,78E-46	0,767668	0,678	0,262	8,39E-42	2	Whsc1
1,81E-45	0,702021	0,588	0,189	2,24E-41	2	Tk1
2,63E-45	-1,23366	0,555	0,861	3,25E-41	2	Macf1
4,33E-45	0,392133	0,29	0,041	5,35E-41	2	Cenpn
5,26E-45	-1,13906	0,894	0,981	6,51E-41	2	Gm17821
7,39E-45	0,728993	0,604	0,2	9,15E-41	2	Psat1
1,59E-44	0,905355	0,91	0,586	1,97E-40	2	Ldha
1,79E-44	0,522561	0,335	0,059	2,21E-40	2	Aurkb
3,03E-44	-1,63829	0,073	0,56	3,75E-40	2	Mndal
1,76E-43	-1,01156	0,518	0,854	2,18E-39	2	Btg1
7,57E-43	0,752862	0,996	0,97	9,37E-39	2	Hspa8
1,11E-42	0,511032	0,314	0,054	1,38E-38	2	Kif11
1,82E-42	0,73685	0,829	0,44	2,25E-38	2	Cbx3
2,39E-42	0,643699	0,469	0,128	2,96E-38	2	Gatm
9,65E-42	0,781355	0,551	0,185	1,19E-37	2	Ncapd2
1,1E-41	0,511111	0,445	0,114	1,36E-37	2	Paxip1
1,2E-41	0,419011	0,363	0,074	1,48E-37	2	Wdhd1
1,34E-41	0,85968	0,759	0,392	1,66E-37	2	Pa2g4
1,62E-41	1,093553	0,788	0,456	2E-37	2	Mif
2,19E-41	0,754486	0,935	0,666	2,71E-37	2	Sumo2
4,45E-41	0,386102	0,367	0,075	5,51E-37	2	Rfc5
4,71E-41	0,568742	0,294	0,05	5,83E-37	2	Spc25
1,6E-40	0,42323	0,286	0,045	1,98E-36	2	Slc43a3
1,7E-40	0,87525	0,853	0,482	2,1E-36	2	Marcksl1
1,76E-40	0,623607	1	0,999	2,17E-36	2	Cfl1
2,29E-40	0,327503	0,224	0,026	2,83E-36	2	Sgol1
2,56E-40	0,765656	0,71	0,33	3,17E-36	2	Cdk4
3,93E-40	0,470736	0,327	0,063	4,87E-36	2	Ung
4,72E-40	-0,96638	0,959	0,997	5,85E-36	2	Kcnq1ot1
1,23E-39	0,350809	0,249	0,034	1,53E-35	2	Bard1
1,24E-39	0,56356	0,371	0,084	1,53E-35	2	Incenp

1,67E-39	0,767632	0,722	0,336	2,07E-35	2	Tpi1
2,33E-39	0,442925	0,392	0,092	2,88E-35	2	Nup85
3,99E-39	0,455399	0,388	0,091	4,94E-35	2	E2f1
4,58E-39	-1,45852	0,216	0,631	5,68E-35	2	Cmah
4,75E-39	0,367546	0,261	0,039	5,89E-35	2	Timeless
5,54E-39	-0,85995	0,967	0,992	6,86E-35	2	H2-D1
6,08E-39	0,638185	0,539	0,174	7,53E-35	2	Cks2
7,41E-39	0,517087	0,359	0,079	9,17E-35	2	Pole2
9,13E-39	-1,39355	0,143	0,584	1,13E-34	2	Ly6d
2,36E-38	0,695988	0,518	0,174	2,92E-34	2	Mad2l1
2,43E-38	0,507218	0,498	0,151	3,01E-34	2	Cdk2
1,96E-37	0,739521	0,967	0,764	2,43E-33	2	Slc25a5
2,21E-37	0,591733	0,4	0,103	2,74E-33	2	Smc2
2,37E-37	0,330756	0,208	0,024	2,94E-33	2	Bub1
3,6E-37	0,848714	0,808	0,479	4,46E-33	2	Paics
4,76E-37	0,70992	0,661	0,289	5,9E-33	2	Dek
5,63E-37	0,739937	0,992	0,955	6,97E-33	2	Npm1
5,72E-37	0,502705	0,286	0,052	7,08E-33	2	Nusap1
7,94E-37	0,503314	0,322	0,068	9,83E-33	2	Mms22l
1,17E-36	0,712866	0,951	0,795	1,45E-32	2	Hnrnpa3
2,38E-36	-1,33354	0,102	0,531	2,94E-32	2	Ifi203
2,71E-36	0,451484	0,4	0,104	3,36E-32	2	Alyref
5,82E-36	0,316085	0,22	0,029	7,2E-32	2	Ckap2l
6,76E-36	0,374334	0,273	0,048	8,37E-32	2	Chaf1b
7,66E-36	0,555027	0,555	0,202	9,48E-32	2	Nup62
8,29E-36	0,720583	0,812	0,453	1,03E-31	2	Pgk1
9,24E-36	0,322297	0,192	0,021	1,14E-31	2	Foxm1
1,71E-35	0,495726	0,408	0,109	2,12E-31	2	Idi1
1,85E-35	-0,59381	0,992	0,998	2,29E-31	2	Cd37
1,96E-35	0,699738	0,743	0,359	2,43E-31	2	Nme1
2,21E-35	-1,26762	0,102	0,519	2,73E-31	2	Pml
4,33E-35	0,650494	0,918	0,627	5,36E-31	2	Prdx1
6,12E-35	0,72783	0,886	0,599	7,57E-31	2	Hmgn1
7,52E-35	0,724399	0,955	0,759	9,31E-31	2	Anp32b
1,05E-34	0,364522	0,204	0,026	1,3E-30	2	Ttk
1,52E-34	0,607006	0,518	0,182	1,88E-30	2	Atad2
1,53E-34	0,64627	0,955	0,694	1,89E-30	2	Atp5g3
1,89E-34	0,601555	0,804	0,422	2,34E-30	2	Cox5a
2,84E-34	0,284862	0,188	0,021	3,52E-30	2	Zwilch
3,24E-34	0,795363	0,943	0,626	4,01E-30	2	Igj
3,38E-34	0,466644	0,322	0,071	4,18E-30	2	Kif23
3,92E-34	0,405681	0,412	0,114	4,85E-30	2	Csrp1
4,63E-34	0,387847	0,327	0,073	5,74E-30	2	Wdr76
6,59E-34	0,553815	0,731	0,323	8,15E-30	2	Dbi
8,34E-34	0,465297	0,465	0,143	1,03E-29	2	Cdt1
8,41E-34	0,745107	0,824	0,443	1,04E-29	2	Mef2b
1,06E-33	-0,98431	0,833	0,958	1,31E-29	2	A630089N07Rik
1,64E-33	0,357048	0,151	0,012	2,04E-29	2	Ankle1
1,93E-33	0,850296	0,857	0,538	2,39E-29	2	Klhl6
1,97E-33	0,286269	0,261	0,046	2,43E-29	2	Hirip3

2,05E-33	0,389575	0,261	0,046	2,54E-29	2	C330027C09Rik
3,31E-33	0,258957	0,188	0,022	4,1E-29	2	Psmc3ip
3,37E-33	0,672388	0,939	0,712	4,17E-29	2	Ywhae
4,47E-33	0,583041	0,988	0,91	5,54E-29	2	Rbm3
6,93E-33	0,320442	0,257	0,045	8,58E-29	2	4930422G04Rik
8,03E-33	0,613689	0,653	0,292	9,94E-29	2	H2afv
8,27E-33	0,338519	0,253	0,044	1,02E-28	2	Slc29a1
9,48E-33	0,349605	0,249	0,043	1,17E-28	2	Casc5
1,39E-32	-1,11015	0,253	0,618	1,72E-28	2	Bank1
2,6E-32	0,546815	0,465	0,151	3,22E-28	2	Pla2g12a
3,59E-32	-1,09808	0,38	0,719	4,44E-28	2	Gimap4
3,77E-32	0,815939	0,951	0,842	4,67E-28	2	Eif5a
4,09E-32	0,308153	0,196	0,026	5,07E-28	2	Ccnf
4,14E-32	0,452637	0,437	0,134	5,13E-28	2	Lmnb1
4,35E-32	0,565426	0,527	0,199	5,38E-28	2	Rfwd3
4,47E-32	0,478829	0,478	0,161	5,54E-28	2	Msh6
4,83E-32	0,712685	0,808	0,477	5,99E-28	2	Cycs
5,13E-32	-0,8477	0,494	0,784	6,35E-28	2	Hmha1
5,59E-32	0,492704	0,6	0,235	6,92E-28	2	Ahcy
1,47E-31	0,278791	0,22	0,034	1,82E-27	2	Pkmyt1
1,5E-31	0,358948	0,269	0,053	1,85E-27	2	Ube2t
1,57E-31	0,606344	0,849	0,51	1,94E-27	2	Snrpe
1,8E-31	0,62226	0,792	0,434	2,23E-27	2	Rbbp4
2,96E-31	0,634552	0,804	0,456	3,67E-27	2	Hprt
5,59E-31	0,521275	0,58	0,235	6,92E-27	2	Rbmxl1
5,83E-31	0,584688	0,869	0,528	7,22E-27	2	Hnrnpd
7,45E-31	0,431861	0,327	0,079	9,23E-27	2	Cks1b
1,31E-30	0,666432	0,955	0,828	1,63E-26	2	Ybx1
2,45E-30	-1,11947	0,078	0,455	3,04E-26	2	Klf2
2,84E-30	0,364252	0,224	0,038	3,52E-26	2	Kif4
3,26E-30	-0,79088	0,951	0,985	4,04E-26	2	H2-K1
4,15E-30	-0,87461	0,653	0,838	5,13E-26	2	Ctss
5,19E-30	0,415793	0,314	0,075	6,43E-26	2	Cdca8
5,98E-30	0,557217	0,722	0,361	7,4E-26	2	Anapc5
7,85E-30	0,716786	0,792	0,465	9,72E-26	2	Pgam1
8,58E-30	0,409476	0,355	0,097	1,06E-25	2	Mthfd2
8,92E-30	-0,88518	0,171	0,55	1,1E-25	2	Gimap8
8,96E-30	-1,23254	0,086	0,467	1,11E-25	2	Sell
9,71E-30	0,420854	0,343	0,092	1,2E-25	2	Nelfe
1,07E-29	0,509734	0,996	0,979	1,32E-25	2	Hnrnpa2b1
1,07E-29	0,302793	0,224	0,038	1,33E-25	2	4930427A07Rik
1,08E-29	0,527927	0,527	0,203	1,34E-25	2	Ybx3
1,19E-29	0,557253	0,535	0,212	1,47E-25	2	2700094K13Rik
1,27E-29	0,615836	0,853	0,507	1,58E-25	2	Atp5j
1,35E-29	0,626368	0,571	0,238	1,67E-25	2	Usp1
2,58E-29	0,335944	0,347	0,093	3,19E-25	2	Lsm2
3,42E-29	-0,99532	0,58	0,832	4,23E-25	2	Grk4
3,42E-29	0,459013	0,633	0,266	4,24E-25	2	Eif1ax
3,54E-29	-0,99294	0,163	0,54	4,39E-25	2	Itga4
4,1E-29	0,571824	0,592	0,257	5,07E-25	2	Samd1

4,42E-29	0,31002	0,204	0,032	5,47E-25	2	Espl1
5,12E-29	-0,92678	0,584	0,824	6,33E-25	2	Mycbp2
6,48E-29	0,295034	0,224	0,04	8,02E-25	2	Ndc80
9,9E-29	-0,91719	0,082	0,455	1,23E-24	2	Capg
1,04E-28	-0,60007	0,98	0,994	1,29E-24	2	H2-Ob
2,24E-28	-0,67809	0,988	0,997	2,78E-24	2	B2m
2,26E-28	0,486389	0,408	0,136	2,8E-24	2	Phgdh
4,27E-28	0,289876	0,22	0,04	5,29E-24	2	Cenpm
6,82E-28	-0,96676	0,322	0,644	8,44E-24	2	Lmo2
6,83E-28	-0,57089	1	0,999	8,46E-24	2	Ly6e
9,74E-28	0,592289	0,971	0,829	1,21E-23	2	Set
1E-27	0,323452	0,257	0,055	1,24E-23	2	Cenpw
1,68E-27	0,415071	0,388	0,123	2,08E-23	2	Rpa3
1,78E-27	0,60664	0,661	0,35	2,2E-23	2	Ssrp1
1,82E-27	0,60288	0,845	0,545	2,26E-23	2	Mdh1
1,94E-27	0,408992	0,453	0,158	2,4E-23	2	Uchl5
1,97E-27	0,626721	0,731	0,427	2,44E-23	2	Eif4a1
3,34E-27	0,440928	0,58	0,245	4,14E-23	2	Pold1
3,66E-27	-1,0007	0,18	0,527	4,53E-23	2	Fam65b
6,18E-27	0,515349	0,816	0,469	7,65E-23	2	Snrpb
7,02E-27	0,373463	0,457	0,161	8,69E-23	2	Ppil1
7,02E-27	0,549474	0,959	0,765	8,69E-23	2	Srsf3
1,08E-26	-0,78037	0,71	0,877	1,34E-22	2	D4Wsu53e
1,12E-26	-0,88698	0,237	0,577	1,39E-22	2	Gimap3
1,34E-26	0,322192	0,22	0,041	1,66E-22	2	Mis18bp1
1,74E-26	0,293337	0,233	0,047	2,15E-22	2	Brca2
2,68E-26	0,566901	0,943	0,801	3,32E-22	2	Serbp1
3,19E-26	0,517689	0,653	0,318	3,95E-22	2	Rfc2
3,2E-26	0,570327	0,465	0,178	3,96E-22	2	Pim1
3,32E-26	0,487681	0,612	0,275	4,11E-22	2	Naa40
3,68E-26	0,628634	0,988	0,955	4,56E-22	2	Hsp90ab1
3,79E-26	0,259875	0,208	0,038	4,69E-22	2	Dctpp1
4,34E-26	-0,8073	0,416	0,716	5,37E-22	2	Pisd-ps1
6,01E-26	-0,4226	1	1	7,44E-22	2	H2-Aa
7,63E-26	0,424444	0,535	0,218	9,45E-22	2	Exosc8
8,34E-26	0,429549	0,408	0,138	1,03E-21	2	Tfrc
8,68E-26	0,565387	0,939	0,763	1,07E-21	2	Pkm
1,08E-25	-0,83088	0,261	0,597	1,34E-21	2	Samd9l
1,13E-25	0,543218	0,898	0,703	1,4E-21	2	Hnrnpu
1,51E-25	-0,87492	0,31	0,62	1,87E-21	2	Gimap6
2,65E-25	0,268977	0,208	0,039	3,28E-21	2	Ccne1
2,82E-25	0,41155	0,441	0,16	3,5E-21	2	Ipo5
3,04E-25	0,40649	0,237	0,052	3,76E-21	2	Spag5
4,66E-25	0,425174	0,363	0,116	5,77E-21	2	Birc5
5,33E-25	-0,79567	0,041	0,376	6,6E-21	2	S100a10
5,71E-25	0,567759	0,714	0,401	7,07E-21	2	Anp32e
5,74E-25	0,427079	0,351	0,109	7,1E-21	2	Racgap1
7,13E-25	0,555896	0,927	0,685	8,83E-21	2	Srsf7
8E-25	0,416167	0,322	0,093	9,9E-21	2	Prc1
9,09E-25	-0,488	1	0,997	1,13E-20	2	Ddx5

1,01E-24	-0,81048	0,457	0,713	1,25E-20	2	Scd1
1,03E-24	0,697609	0,743	0,439	1,28E-20	2	Nop56
1,05E-24	-0,92532	0,212	0,525	1,3E-20	2	Txnip
1,08E-24	0,318315	0,298	0,079	1,33E-20	2	Ube2e3
1,1E-24	-0,82242	0,086	0,436	1,36E-20	2	Snn
1,42E-24	-0,93464	0,061	0,398	1,76E-20	2	Gm1966
1,49E-24	0,694083	0,784	0,5	1,85E-20	2	Smc4
1,59E-24	-0,89961	0,612	0,82	1,97E-20	2	Faim3
1,62E-24	0,439695	0,473	0,186	2,01E-20	2	Rbl1
1,96E-24	0,569382	0,441	0,172	2,43E-20	2	H2afx
2,24E-24	-0,79312	0,11	0,445	2,77E-20	2	Mylip
2,62E-24	0,250679	0,167	0,026	3,24E-20	2	Trip13
2,9E-24	0,29147	0,257	0,062	3,58E-20	2	Cdc25a
3E-24	-0,93664	0,041	0,365	3,72E-20	2	Bcl2
3,01E-24	0,292337	0,335	0,099	3,73E-20	2	Pola2
3,51E-24	0,469697	0,763	0,411	4,35E-20	2	Cct8
4,64E-24	-0,79542	0,539	0,764	5,75E-20	2	Stk17b
4,67E-24	0,405139	0,396	0,138	5,79E-20	2	Rnaseh2b
4,71E-24	0,591252	0,902	0,751	5,83E-20	2	Atp5b
7,48E-24	0,504851	0,947	0,771	9,26E-20	2	Atp5a1
7,99E-24	0,555246	0,739	0,412	9,89E-20	2	Dck
9,8E-24	0,533968	0,669	0,348	1,21E-19	2	Suz12
1,12E-23	-0,37026	1	1	1,38E-19	2	H2-Eb1
1,24E-23	0,344966	0,392	0,136	1,54E-19	2	Mrpl12
1,25E-23	0,530913	0,731	0,418	1,55E-19	2	Rfc1
1,36E-23	0,451267	0,596	0,274	1,68E-19	2	Phf5a
1,46E-23	-0,80536	0,122	0,449	1,8E-19	2	Anxa6
1,73E-23	-0,7944	0,102	0,441	2,14E-19	2	B3gnt5
1,76E-23	0,5572	0,665	0,361	2,18E-19	2	Smc1a
1,76E-23	0,499467	1	0,991	2,18E-19	2	Pabpc1
2,02E-23	0,362604	0,404	0,143	2,5E-19	2	Dtymk
2,21E-23	0,541573	0,727	0,412	2,74E-19	2	Psip1
2,42E-23	0,478265	0,996	0,937	3E-19	2	Nap1l1
3,34E-23	0,298305	0,257	0,064	4,13E-19	2	Ccdc34
4,55E-23	0,478943	0,722	0,405	5,63E-19	2	Azin1
4,59E-23	0,406938	0,445	0,174	5,68E-19	2	C1qbp
4,67E-23	0,391184	0,347	0,113	5,78E-19	2	Tpx2
5,05E-23	0,569324	0,722	0,411	6,26E-19	2	Nhp2l1
6,06E-23	0,387525	0,433	0,164	7,51E-19	2	Wbp5
7,97E-23	0,273761	0,257	0,065	9,87E-19	2	Rbbp8
8,08E-23	0,540635	0,894	0,642	1E-18	2	Pcbp1
1,01E-22	0,456685	0,624	0,308	1,25E-18	2	Rps27l
1,03E-22	0,462079	0,706	0,384	1,27E-18	2	Syncrip
1,07E-22	0,467092	0,694	0,386	1,33E-18	2	Tcp1
1,09E-22	-0,5021	0,996	0,999	1,36E-18	2	Cd19
1,31E-22	0,461465	0,624	0,315	1,63E-18	2	Mrpl18
1,37E-22	-0,64902	0,902	0,965	1,69E-18	2	Cd22
1,43E-22	0,508882	0,829	0,524	1,77E-18	2	Psmb7
1,46E-22	0,377562	0,306	0,093	1,81E-18	2	Rcc1
1,59E-22	0,462046	0,657	0,337	1,97E-18	2	Taf9

1,6E-22	0,478139	0,49	0,208	1,98E-18	2	Nop58
2,08E-22	0,505256	0,486	0,212	2,58E-18	2	Kpna2
2,33E-22	-0,75927	0,192	0,502	2,89E-18	2	Pld4
2,73E-22	0,453456	0,653	0,331	3,38E-18	2	Usmg5
2,9E-22	0,271885	0,237	0,057	3,59E-18	2	Tmem48
3,31E-22	0,492632	0,518	0,235	4,1E-18	2	Ndufab1
3,45E-22	0,410977	0,396	0,142	4,27E-18	2	H1fx
3,54E-22	0,295804	0,208	0,045	4,38E-18	2	Cep55
3,89E-22	0,450926	0,498	0,215	4,82E-18	2	Hspd1
4,24E-22	0,396368	0,514	0,227	5,25E-18	2	Uchl3
4,36E-22	-0,91773	0,212	0,522	5,4E-18	2	Abca1
6,76E-22	0,485671	0,731	0,42	8,37E-18	2	Nucks1
1,1E-21	0,448343	0,543	0,25	1,36E-17	2	Xrcc1
1,28E-21	-0,49294	0,98	0,993	1,59E-17	2	Cd79b
1,92E-21	-0,70704	0,42	0,687	2,38E-17	2	H2-T23
1,92E-21	0,2675	0,265	0,072	2,38E-17	2	Haus4
2,03E-21	0,47472	0,718	0,432	2,51E-17	2	Kpnb1
2,56E-21	0,42693	1	0,997	3,17E-17	2	Rplp0
2,61E-21	0,343734	0,404	0,151	3,23E-17	2	Casp8ap2
3,44E-21	0,433775	0,661	0,351	4,26E-17	2	Pnp
3,53E-21	0,400584	0,437	0,179	4,37E-17	2	Nhp2
3,54E-21	-0,85792	0,127	0,429	4,38E-17	2	Dgka
4,75E-21	0,481991	0,322	0,109	5,88E-17	2	Nuf2
4,89E-21	-0,87566	0,216	0,52	6,06E-17	2	Dock10
5E-21	0,482743	0,314	0,104	6,19E-17	2	Srm
5,03E-21	0,306038	0,302	0,092	6,23E-17	2	Arhgap11a
5,18E-21	0,476604	0,633	0,342	6,41E-17	2	Tkt
7,33E-21	-0,6265	0,016	0,3	9,07E-17	2	Itgb7
7,41E-21	0,358502	0,482	0,2	9,17E-17	2	Cisd1
7,59E-21	0,338716	0,335	0,112	9,4E-17	2	Zfp367
8,13E-21	-0,80567	0,147	0,447	1,01E-16	2	Add3
8,66E-21	0,393999	0,473	0,207	1,07E-16	2	Ncaph2
1,03E-20	0,322462	0,363	0,129	1,27E-16	2	Ada
1,05E-20	0,472684	0,771	0,478	1,3E-16	2	Cct5
1,06E-20	0,618546	1	0,992	1,32E-16	2	Rps2
1,15E-20	0,28275	0,241	0,063	1,42E-16	2	Cdkn2c
1,19E-20	0,503966	0,931	0,761	1,48E-16	2	Atp5g2
1,32E-20	0,475149	0,873	0,641	1,63E-16	2	Cox6a1
1,42E-20	0,388	0,588	0,287	1,76E-16	2	Arpp19
1,59E-20	0,292638	0,18	0,037	1,97E-16	2	Sgol2
1,67E-20	0,298911	0,253	0,069	2,07E-16	2	Ncapd3
1,67E-20	0,31744	0,416	0,16	2,07E-16	2	Pbdc1
1,93E-20	0,431879	0,702	0,396	2,39E-16	2	Glrx3
3,66E-20	0,348307	0,449	0,183	4,53E-16	2	Rgs10
3,79E-20	0,393398	0,563	0,268	4,69E-16	2	Tceb1
4,09E-20	0,428965	0,722	0,414	5,06E-16	2	Psma1
4,35E-20	-0,8687	0,547	0,756	5,38E-16	2	Ly6a
4,74E-20	0,459033	0,514	0,245	5,86E-16	2	Mrpl42
4,75E-20	0,355378	0,429	0,172	5,88E-16	2	Snrpa1
4,95E-20	0,397289	1	0,997	6,13E-16	2	Actg1

5,5E-20	-0,67714	0,543	0,764	6,81E-16	2	Siglecg
7,11E-20	0,2874	0,245	0,068	8,81E-16	2	Hmgb3
8,26E-20	0,427479	0,547	0,263	1,02E-15	2	Uba2
1,29E-19	-0,67357	0,008	0,275	1,6E-15	2	Cd55
1,3E-19	0,297165	0,278	0,084	1,62E-15	2	Tinf2
1,76E-19	0,415107	0,429	0,176	2,18E-15	2	Cyp51
1,97E-19	0,375335	0,531	0,252	2,43E-15	2	Impdh2
2,02E-19	0,423226	0,547	0,27	2,5E-15	2	Rpa1
2,1E-19	-0,61235	0,771	0,894	2,59E-15	2	Gm10845
3,14E-19	0,566212	0,922	0,777	3,89E-15	2	Pou2af1
3,17E-19	0,459317	0,531	0,265	3,92E-15	2	Ube2s
3,21E-19	0,453021	0,62	0,34	3,97E-15	2	Naa50
4,02E-19	0,378245	0,514	0,24	4,98E-15	2	Tagln2
5,8E-19	0,375509	0,58	0,286	7,18E-15	2	Cse1l
5,93E-19	0,357022	0,42	0,176	7,34E-15	2	Lsm3
6,83E-19	0,28624	0,253	0,074	8,46E-15	2	Pold2
6,84E-19	-0,75025	0,102	0,379	8,46E-15	2	Map3k1
7,64E-19	-0,80801	0,176	0,458	9,46E-15	2	Sub1
9,82E-19	0,397367	0,384	0,156	1,22E-14	2	Wee1
1,05E-18	0,492824	0,71	0,404	1,29E-14	2	Cmpk1
1,3E-18	0,605347	0,853	0,698	1,61E-14	2	Ncl
1,41E-18	-0,53976	0,02	0,28	1,75E-14	2	Hhex
1,5E-18	0,309876	0,473	0,203	1,86E-14	2	Ubap2
1,51E-18	0,482015	0,698	0,431	1,86E-14	2	Ptbp1
1,66E-18	0,759984	0,861	0,683	2,05E-14	2	Erdr1
1,68E-18	0,372375	0,486	0,228	2,08E-14	2	Nudt21
1,96E-18	0,347986	0,155	0,031	2,43E-14	2	Gstt1
2,13E-18	-0,64912	0,216	0,487	2,64E-14	2	Psap
2,31E-18	-0,55075	0,082	0,355	2,86E-14	2	Tspan32
2,52E-18	-0,72566	0,204	0,493	3,12E-14	2	Evi2b
2,81E-18	0,437614	0,878	0,633	3,48E-14	2	Ppp1ca
3,3E-18	0,61745	0,592	0,355	4,09E-14	2	Shmt2
3,59E-18	0,380147	0,563	0,277	4,45E-14	2	1810037I17Rik
3,9E-18	0,401297	0,665	0,366	4,82E-14	2	Odc1
4,22E-18	0,40364	0,804	0,528	5,23E-14	2	Tra2b
4,28E-18	0,407022	0,531	0,265	5,3E-14	2	Txnl1
4,34E-18	0,372075	0,363	0,142	5,37E-14	2	Pno1
4,66E-18	0,426179	0,322	0,118	5,77E-14	2	Cenpe
4,94E-18	0,395712	0,567	0,288	6,12E-14	2	Hdgf
5,59E-18	0,351292	0,437	0,194	6,92E-14	2	Pdap1
5,76E-18	-0,58529	0,392	0,632	7,13E-14	2	Dok3
7,07E-18	0,44135	0,727	0,448	8,76E-14	2	Psma2
7,72E-18	-0,78559	0,306	0,543	9,56E-14	2	Itm2b
8,34E-18	0,25121	0,261	0,081	1,03E-13	2	Cdc7
8,7E-18	0,38451	0,637	0,352	1,08E-13	2	Vdac3
9,15E-18	-0,68276	0,596	0,791	1,13E-13	2	Prkcb
1,01E-17	-0,71915	0,282	0,552	1,25E-13	2	Pyhin1
1,6E-17	0,418384	0,698	0,415	1,98E-13	2	Cdk2ap1
1,7E-17	0,389624	0,702	0,421	2,1E-13	2	Atp5j2
1,84E-17	-0,64329	0,045	0,301	2,27E-13	2	Serpinb1a

1,84E-17	-0,62201	0,396	0,65	2,28E-13	2	Tbc1d10c
2,12E-17	0,324209	0,576	0,291	2,63E-13	2	Uqcr10
2,29E-17	0,329624	0,322	0,122	2,84E-13	2	Brip1
2,54E-17	0,445607	0,514	0,264	3,15E-13	2	Dkc1
2,63E-17	0,286389	0,286	0,096	3,26E-13	2	Mis18a
2,77E-17	0,53502	0,657	0,393	3,43E-13	2	Gcsam
3,32E-17	-0,62562	0,163	0,434	4,11E-13	2	Ubl3
3,48E-17	0,381894	0,624	0,347	4,31E-13	2	Snrpf
3,93E-17	0,410061	0,649	0,389	4,86E-13	2	Lsm4
4,29E-17	0,27079	0,322	0,118	5,31E-13	2	Atic
4,41E-17	0,294886	1	1	5,46E-13	2	Actb
4,71E-17	0,34734	0,449	0,202	5,83E-13	2	Etfa
4,96E-17	0,486287	0,988	0,953	6,14E-13	2	Pfn1
5,17E-17	0,303116	0,298	0,106	6,4E-13	2	Hjurp
5,32E-17	0,42978	0,849	0,64	6,59E-13	2	Tardbp
5,51E-17	0,252465	0,261	0,083	6,83E-13	2	Ttf2
5,95E-17	0,352286	1	0,991	7,37E-13	2	H3f3a
6,57E-17	-0,7947	0,302	0,553	8,13E-13	2	Foxp1
7,48E-17	0,319341	0,596	0,313	9,26E-13	2	Bub3
8,34E-17	-0,65876	0,163	0,427	1,03E-12	2	Trim34a
8,38E-17	-0,81018	0,273	0,542	1,04E-12	2	Vim
9,48E-17	-0,60535	0,58	0,771	1,17E-12	2	Ltb
9,58E-17	-0,66814	0,294	0,536	1,19E-12	2	Ralgps2
9,74E-17	0,487108	0,865	0,628	1,21E-12	2	Banf1
9,94E-17	0,421915	0,878	0,725	1,23E-12	2	Hnrnpf
1,01E-16	0,397689	0,853	0,595	1,26E-12	2	Snrpg
1,08E-16	0,454014	0,633	0,361	1,34E-12	2	Cct6a
1,09E-16	0,463599	0,637	0,389	1,35E-12	2	Eif2s1
1,18E-16	0,412306	0,78	0,531	1,46E-12	2	Nono
1,25E-16	-0,70591	0,4	0,641	1,55E-12	2	Mll5
1,33E-16	0,342713	1	0,992	1,64E-12	2	Rpl28
1,34E-16	-0,602	0,012	0,245	1,66E-12	2	Arhgef18
1,38E-16	0,347244	0,555	0,291	1,71E-12	2	Nsmce4a
1,59E-16	-0,66774	0,718	0,843	1,97E-12	2	Lax1
1,89E-16	0,356399	0,535	0,273	2,34E-12	2	Sfxn1
1,91E-16	-0,59232	0,143	0,402	2,37E-12	2	Cyb561a3
2,14E-16	0,358944	1	0,999	2,65E-12	2	Rpl41
2,19E-16	0,31582	0,384	0,164	2,71E-12	2	Naa38
2,25E-16	0,295787	0,522	0,255	2,78E-12	2	Ddx39
2,32E-16	0,433263	0,796	0,535	2,88E-12	2	Trim28
2,56E-16	0,366489	0,551	0,287	3,17E-12	2	Atp1b3
2,86E-16	0,360976	0,51	0,252	3,54E-12	2	Snhg1
4E-16	0,284016	0,298	0,108	4,95E-12	2	Tex30
4,18E-16	0,270017	0,282	0,1	5,18E-12	2	Prdx4
4,35E-16	0,3274	0,498	0,241	5,38E-12	2	Smagp
5,79E-16	-0,83495	0,486	0,676	7,17E-12	2	Vmn2r55
5,79E-16	0,38722	0,592	0,329	7,17E-12	2	AI662270
5,82E-16	0,344695	0,62	0,341	7,21E-12	2	Ube2n
5,85E-16	-0,67046	0,233	0,487	7,25E-12	2	Filip1l
5,98E-16	0,356892	0,478	0,235	7,4E-12	2	U2af1

6,66E-16	0,285306	0,42	0,185	8,24E-12	2	Tmem256
6,85E-16	-0,51581	0,008	0,231	8,47E-12	2	Ccr6
7,7E-16	0,363567	0,359	0,15	9,54E-12	2	Ngfrap1
8,88E-16	-0,6888	0,351	0,586	1,1E-11	2	Gm10785
9,25E-16	-0,3294	1	0,999	1,15E-11	2	Rps24
9,75E-16	0,34745	0,796	0,504	1,21E-11	2	Ppp4r2
1,03E-15	0,347767	0,502	0,26	1,28E-11	2	Anapc11
1,07E-15	0,318266	0,404	0,177	1,32E-11	2	Asf1a
1,1E-15	0,36396	0,392	0,172	1,36E-11	2	Suv39h1
1,12E-15	0,299905	0,233	0,074	1,39E-11	2	Timm8a1
1,12E-15	-0,6165	0,751	0,873	1,39E-11	2	Samhd1
1,14E-15	0,43563	0,563	0,326	1,41E-11	2	Atp5g1
1,18E-15	-0,60166	0,037	0,271	1,46E-11	2	Gpr183
1,19E-15	-0,60618	0,033	0,262	1,47E-11	2	Zfp318
1,25E-15	0,36973	0,449	0,214	1,55E-11	2	Ccnd3
1,27E-15	0,367285	0,653	0,401	1,58E-11	2	Khsrp
1,28E-15	0,289054	0,424	0,191	1,58E-11	2	Eri1
1,34E-15	0,382377	0,878	0,63	1,66E-11	2	Cox6c
1,39E-15	0,476084	0,665	0,402	1,72E-11	2	8430410A17Rik
1,51E-15	0,407165	0,837	0,565	1,87E-11	2	Hnrnpa0
1,57E-15	0,413666	0,62	0,366	1,95E-11	2	Cct3
1,8E-15	0,286733	0,331	0,133	2,23E-11	2	Nt5c3b
1,81E-15	0,371784	0,49	0,262	2,24E-11	2	Tmem14c
1,87E-15	-0,58933	0,437	0,639	2,32E-11	2	Gimap1
2,25E-15	0,413754	0,751	0,508	2,79E-11	2	Psma7
2,31E-15	-0,58563	0,18	0,421	2,86E-11	2	Il4ra
2,55E-15	0,348105	0,527	0,282	3,15E-11	2	Phb2
2,77E-15	0,252654	1	1	3,43E-11	2	mtNd1
2,87E-15	0,325885	0,253	0,089	3,55E-11	2	Gstt2
2,91E-15	-0,64466	0,176	0,419	3,61E-11	2	Stk10
3,08E-15	0,40291	0,735	0,485	3,81E-11	2	Eif4h
3,15E-15	0,293914	0,192	0,053	3,9E-11	2	Ckap2
3,37E-15	-0,60759	0,539	0,722	4,17E-11	2	Smg1
3,37E-15	0,324056	0,273	0,1	4,17E-11	2	Pgp
3,45E-15	0,328182	0,42	0,193	4,27E-11	2	Dpy30
3,69E-15	0,360618	0,682	0,409	4,57E-11	2	Atp5o
4,02E-15	0,423512	0,853	0,613	4,98E-11	2	Erp44
4,5E-15	0,376038	0,996	0,981	5,57E-11	2	Ucp2
4,61E-15	0,250777	0,176	0,047	5,7E-11	2	Blm
4,78E-15	0,43163	0,722	0,486	5,92E-11	2	Eif2s2
5,46E-15	0,447925	0,759	0,491	6,76E-11	2	Tceb2
5,73E-15	0,279529	0,351	0,148	7,09E-11	2	Dera
5,92E-15	0,350754	0,751	0,461	7,33E-11	2	Atp5f1
5,95E-15	-0,60308	0,237	0,494	7,36E-11	2	A130077B15Rik
6,03E-15	0,386239	0,78	0,517	7,47E-11	2	Psmb4
6,6E-15	0,366453	0,437	0,214	8,17E-11	2	Nudc
7,46E-15	0,335028	0,347	0,149	9,24E-11	2	Smc5
8,62E-15	0,325841	0,473	0,235	1,07E-10	2	Sf3a3
8,71E-15	0,333552	0,408	0,191	1,08E-10	2	Hspa14
8,94E-15	0,413872	0,453	0,234	1,11E-10	2	Actn4

9,04E-15	0,441061	0,816	0,551	1,12E-10	2	Mbd2
1,09E-14	-0,6115	0,449	0,666	1,35E-10	2	Lbh
1,15E-14	0,311615	0,298	0,115	1,43E-10	2	Ccnb2
1,26E-14	-0,55969	0,686	0,822	1,56E-10	2	Mll1
1,47E-14	-0,59793	0,029	0,243	1,82E-10	2	S1pr1
1,54E-14	0,289132	0,702	0,389	1,91E-10	2	S1pr2
1,57E-14	0,293128	0,412	0,193	1,95E-10	2	Snrnp40
1,94E-14	0,367826	0,657	0,4	2,4E-10	2	Rnps1
2,08E-14	-0,58703	0,482	0,677	2,57E-10	2	Blk
2,14E-14	0,390706	0,869	0,679	2,65E-10	2	Srsf1
2,17E-14	-0,57286	0,122	0,353	2,69E-10	2	Rasgrp2
2,29E-14	-0,55124	0,808	0,867	2,83E-10	2	Mcl1
2,36E-14	-0,41148	0,963	0,991	2,92E-10	2	Ptprc
2,38E-14	0,273827	0,339	0,144	2,94E-10	2	Mbnl3
2,82E-14	0,315533	0,355	0,156	3,49E-10	2	Ddx19a
2,87E-14	0,363349	0,535	0,285	3,55E-10	2	Eaf2
2,95E-14	0,525751	0,755	0,566	3,66E-10	2	Nme2
3,06E-14	0,409914	0,653	0,401	3,79E-10	2	Xpo1
3,12E-14	0,336757	0,535	0,284	3,86E-10	2	Etf1
3,27E-14	0,374144	0,49	0,26	4,04E-10	2	Sae1
3,3E-14	0,398803	0,637	0,383	4,09E-10	2	Ndufc2
3,37E-14	0,379352	0,669	0,411	4,18E-10	2	Sf3b4
3,5E-14	0,331972	0,445	0,217	4,33E-10	2	Aars
3,96E-14	0,369078	0,62	0,361	4,9E-10	2	Bzw1
3,99E-14	0,399384	0,612	0,361	4,94E-10	2	Pebp1
4,1E-14	0,455756	0,804	0,602	5,07E-10	2	Calm2
4,1E-14	0,374076	0,461	0,239	5,07E-10	2	Eif4e
4,13E-14	0,316784	0,608	0,355	5,11E-10	2	Baz1b
4,37E-14	0,31507	0,347	0,15	5,41E-10	2	Hmgn5
4,38E-14	-0,4939	0,057	0,277	5,42E-10	2	Cdkn1b
4,38E-14	-0,4864	0,78	0,905	5,43E-10	2	Zfp36l1
4,68E-14	0,361509	0,833	0,596	5,8E-10	2	Cox6b1
4,7E-14	0,275643	0,249	0,09	5,81E-10	2	Hmmr
5,01E-14	0,349877	0,571	0,321	6,2E-10	2	Polr2g
5,32E-14	0,306818	1	1	6,59E-10	2	Rpsa
6,21E-14	0,327686	0,604	0,349	7,68E-10	2	Psmc2
6,45E-14	0,345074	0,739	0,48	7,99E-10	2	Cox5b
6,87E-14	0,259055	0,249	0,09	8,51E-10	2	Carhsp1
7,36E-14	-0,59472	0,645	0,784	9,11E-10	2	Btla
8,36E-14	-0,53144	0,159	0,383	1,03E-09	2	Myl12b
9,24E-14	0,332419	0,535	0,295	1,14E-09	2	Got2
1,07E-13	-0,42595	0,943	0,985	1,32E-09	2	Rpl37a
1,27E-13	0,326941	0,665	0,409	1,57E-09	2	Ptges3
1,28E-13	-0,45931	0,11	0,338	1,59E-09	2	Malt1
1,3E-13	0,310341	0,433	0,216	1,61E-09	2	Smarcc1
1,35E-13	0,272338	0,424	0,202	1,67E-09	2	Actl6a
1,36E-13	0,274115	0,482	0,252	1,69E-09	2	Eif4a3
1,39E-13	-0,50408	0,588	0,737	1,72E-09	2	Gm9846
1,39E-13	0,308382	0,461	0,235	1,72E-09	2	Larp7
1,62E-13	0,329987	0,184	0,055	2E-09	2	Tnfsf9

1,63E-13	-0,5054	0,024	0,225	2,01E-09	2	Scml4
2,02E-13	-0,45856	0,102	0,328	2,5E-09	2	Pbxip1
2,86E-13	0,348854	0,906	0,722	3,54E-09	2	Serf2
3,14E-13	0,342649	0,367	0,172	3,88E-09	2	Tuba1c
3,3E-13	-0,4406	0,057	0,271	4,08E-09	2	Notch2
3,45E-13	-0,28094	1	0,999	4,27E-09	2	Fau
3,45E-13	0,389157	0,829	0,618	4,27E-09	2	Top1
3,5E-13	-0,60711	0,269	0,492	4,33E-09	2	Zfp71-rs1
3,71E-13	0,340795	0,718	0,454	4,59E-09	2	Sec61b
3,73E-13	-0,61562	0,192	0,418	4,62E-09	2	Gns
3,75E-13	0,383526	0,796	0,595	4,65E-09	2	Ppp1cc
3,94E-13	0,400175	0,763	0,53	4,88E-09	2	Vcp
4,03E-13	-0,4725	0,167	0,397	4,99E-09	2	Trim12c
4,21E-13	-0,47004	0,135	0,361	5,21E-09	2	Zmym5
4,33E-13	-0,6178	0,278	0,498	5,36E-09	2	March1
4,49E-13	0,322702	0,559	0,32	5,56E-09	2	Cuta
4,64E-13	0,294047	0,502	0,266	5,74E-09	2	Bzw2
4,73E-13	0,322498	0,445	0,228	5,85E-09	2	Rif1
5,67E-13	0,413859	0,739	0,497	7,02E-09	2	Mtf2
6,17E-13	-0,55766	0,457	0,644	7,64E-09	2	Clk1
6,19E-13	0,36253	0,612	0,373	7,66E-09	2	Helq
6,86E-13	-0,58582	0,273	0,491	8,49E-09	2	Tlr1
6,93E-13	-0,48206	0,127	0,358	8,57E-09	2	Ier5
7,07E-13	0,311645	0,473	0,252	8,75E-09	2	Stra13
7,32E-13	-0,63516	0,02	0,211	9,06E-09	2	Plac8
7,98E-13	0,299149	0,49	0,266	9,88E-09	2	Eed
8,85E-13	-0,48967	0,298	0,499	1,1E-08	2	Acap1
9,37E-13	0,310752	0,894	0,666	1,16E-08	2	Hnrnpk
9,97E-13	0,364685	0,951	0,856	1,23E-08	2	Cd24a
1,03E-12	-0,46393	0,849	0,926	1,27E-08	2	Ptpn6
1,07E-12	-0,26744	1	1	1,32E-08	2	Rpl17
1,27E-12	0,4885	0,31	0,141	1,58E-08	2	Pnkd
1,3E-12	0,29267	0,461	0,239	1,6E-08	2	Ruvbl2
1,32E-12	0,341107	0,702	0,458	1,63E-08	2	Minos1
1,33E-12	0,331075	0,465	0,248	1,65E-08	2	Aldh9a1
1,35E-12	0,327212	0,424	0,22	1,67E-08	2	Isyna1
1,36E-12	0,286901	0,318	0,142	1,68E-08	2	Itpa
1,51E-12	-0,49545	0,122	0,34	1,87E-08	2	Lrrc33
1,65E-12	0,330351	0,437	0,228	2,04E-08	2	Hspa9
1,66E-12	0,326203	0,653	0,404	2,06E-08	2	Pomp
1,85E-12	0,343139	0,461	0,252	2,29E-08	2	Rbm8a
1,99E-12	-0,55098	0,592	0,766	2,46E-08	2	Fcrla
2,22E-12	-0,52633	0,465	0,647	2,75E-08	2	Helz2
2,23E-12	0,266613	0,498	0,272	2,76E-08	2	Prdx2
2,29E-12	-0,5124	0,143	0,361	2,83E-08	2	Rapgef4
2,64E-12	-0,60558	0,245	0,451	3,27E-08	2	Ncf1
2,73E-12	0,306321	0,396	0,199	3,38E-08	2	Snhg5
2,88E-12	-0,52483	0,4	0,593	3,56E-08	2	Arhgap4
2,99E-12	0,250069	0,494	0,257	3,7E-08	2	Tcerg1
3,16E-12	-0,58599	0,486	0,66	3,91E-08	2	B4galnt1

3,32E-12	0,267875	0,514	0,281	4,11E-08	2	Snrpc
3,62E-12	-0,51143	0,167	0,381	4,48E-08	2	Ankrd44
3,77E-12	0,346601	0,71	0,485	4,67E-08	2	Shfm1
3,97E-12	0,315917	0,588	0,358	4,92E-08	2	eGFP
4,53E-12	0,298379	0,465	0,251	5,61E-08	2	Timm23
5,23E-12	0,371765	0,845	0,649	6,48E-08	2	Hspe1
6,02E-12	0,47478	0,429	0,231	7,46E-08	2	Nrgn
6,09E-12	0,338378	0,559	0,341	7,54E-08	2	Smc3
6,15E-12	0,298476	0,882	0,662	7,61E-08	2	Arpc5l
6,26E-12	0,355115	0,718	0,52	7,75E-08	2	Tomm20
6,34E-12	0,282818	0,457	0,243	7,85E-08	2	Snrpd2
6,39E-12	-0,45063	0,11	0,317	7,91E-08	2	Jhdm1d
6,9E-12	-0,48756	0,38	0,573	8,55E-08	2	Wdr26
6,94E-12	-0,43259	0,151	0,361	8,59E-08	2	Rabac1
7,59E-12	0,296924	0,437	0,234	9,39E-08	2	Smarca5
7,61E-12	0,30749	0,547	0,318	9,42E-08	2	Psmb5
8,03E-12	0,278192	0,482	0,267	9,94E-08	2	Oxct1
8,93E-12	0,304014	0,678	0,427	1,11E-07	2	Pmf1
9,36E-12	0,289736	0,571	0,339	1,16E-07	2	Rad23b
1E-11	0,288448	0,327	0,152	1,24E-07	2	Slmo2
1,06E-11	0,288229	0,506	0,285	1,32E-07	2	Kars
1,09E-11	0,291852	0,992	0,962	1,35E-07	2	Calm1
1,1E-11	-0,40465	0,078	0,276	1,36E-07	2	2900060B14Rik
1,11E-11	0,303276	0,404	0,209	1,37E-07	2	Gars
1,11E-11	-0,49701	0,286	0,495	1,37E-07	2	Rasal3
1,27E-11	0,301603	0,416	0,22	1,57E-07	2	Pfkp
1,28E-11	0,315931	0,49	0,274	1,58E-07	2	Cetn3
1,29E-11	0,265755	0,343	0,161	1,6E-07	2	Nup205
1,33E-11	0,289333	0,412	0,214	1,65E-07	2	Slamf7
1,35E-11	-0,46617	0,592	0,744	1,68E-07	2	Iqgap1
1,36E-11	0,285474	0,518	0,295	1,68E-07	2	Ak2
1,4E-11	-0,5451	0,441	0,658	1,73E-07	2	Fcer2a
1,47E-11	-0,46838	0,049	0,236	1,82E-07	2	Lgals9
1,56E-11	0,258643	0,355	0,173	1,93E-07	2	Eftud2
1,71E-11	-0,46845	0,469	0,637	2,12E-07	2	Bin1
1,76E-11	-0,31665	1	1	2,18E-07	2	Cd79a
1,81E-11	-0,59603	0,449	0,615	2,24E-07	2	Zbtb20
1,84E-11	-0,50705	0,098	0,291	2,27E-07	2	Man1a
1,89E-11	-0,40562	0,008	0,173	2,34E-07	2	Cd38
2,1E-11	0,30716	0,457	0,26	2,6E-07	2	Fbl
2,12E-11	-0,32494	0,98	0,991	2,63E-07	2	Rps14
2,16E-11	0,292656	0,318	0,15	2,68E-07	2	Irak1bp1
2,3E-11	0,250393	0,4	0,203	2,85E-07	2	Rangap1
2,31E-11	0,326833	0,367	0,186	2,86E-07	2	Pdk3
2,35E-11	-0,46612	0,106	0,305	2,91E-07	2	Chd2
2,5E-11	0,281789	0,624	0,377	3,09E-07	2	Dcaf12
2,53E-11	0,325331	0,776	0,546	3,13E-07	2	Cyfip2
2,54E-11	0,358762	0,841	0,656	3,14E-07	2	Atp5h
3,03E-11	0,286261	0,433	0,235	3,75E-07	2	Cops6
3,27E-11	0,328161	0,927	0,763	4,04E-07	2	Cox8a

3,33E-11	0,307632	1	0,984	4,13E-07	2	Eef1b2
3,58E-11	0,325141	0,629	0,404	4,43E-07	2	Rcc2
3,66E-11	0,310178	0,416	0,219	4,53E-07	2	Usp14
3,67E-11	0,331226	0,535	0,324	4,55E-07	2	Psma5
3,87E-11	0,290791	0,563	0,351	4,79E-07	2	Sdhb
3,93E-11	0,300973	0,588	0,357	4,86E-07	2	Calm3
4,09E-11	0,354634	0,376	0,191	5,07E-07	2	Siah2
4,16E-11	-0,50041	0,249	0,442	5,14E-07	2	S100a11
4,2E-11	0,294769	0,776	0,55	5,2E-07	2	Oaz1
4,27E-11	0,34453	0,759	0,547	5,29E-07	2	Cox7b
4,32E-11	0,287005	0,616	0,381	5,34E-07	2	Abracl
4,36E-11	0,294257	1	0,995	5,4E-07	2	Tmsb10
4,44E-11	-0,491	0,665	0,784	5,49E-07	2	Arhgef1
4,93E-11	0,283408	0,584	0,365	6,1E-07	2	Hnrnpl
5,02E-11	0,325015	0,727	0,513	6,22E-07	2	Fus
5,09E-11	0,304333	0,518	0,303	6,31E-07	2	Atp2a2
5,47E-11	0,275142	0,355	0,177	6,77E-07	2	Mrpl17
5,51E-11	0,269596	0,551	0,325	6,82E-07	2	Cox7a2
5,64E-11	-0,36589	0,033	0,207	6,98E-07	2	Abcg1
5,75E-11	-0,3983	0,053	0,231	7,12E-07	2	Tcp11l2
6,23E-11	-0,51681	0,58	0,711	7,71E-07	2	Chst3
6,55E-11	-0,35655	0,049	0,228	8,11E-07	2	Phf1
6,6E-11	-0,43168	0,359	0,56	8,17E-07	2	Gm19757
7,63E-11	-0,47769	0,645	0,77	9,45E-07	2	Hvcn1
7,66E-11	0,311822	0,698	0,468	9,49E-07	2	Ssbp3
7,71E-11	0,292588	0,498	0,291	9,55E-07	2	Psmd1
7,92E-11	0,329635	0,42	0,231	9,81E-07	2	Tfg
8,26E-11	0,271602	0,641	0,404	1,02E-06	2	Atp5k
8,36E-11	0,341734	0,784	0,555	1,04E-06	2	Tcea1
8,48E-11	0,260363	0,498	0,281	1,05E-06	2	Sf3a2
8,6E-11	-0,41798	0,139	0,339	1,06E-06	2	Rnase6
9,16E-11	-0,37103	0,024	0,19	1,13E-06	2	Cd97
9,32E-11	-0,47009	0,498	0,651	1,15E-06	2	Unc93b1
1,01E-10	0,360126	0,637	0,427	1,25E-06	2	Fkbp1a
1,06E-10	-0,53541	0,306	0,497	1,32E-06	2	Slc12a6
1,21E-10	0,312416	0,322	0,157	1,5E-06	2	Gnl3
1,23E-10	-0,42115	0,188	0,393	1,53E-06	2	Mllt6
1,23E-10	0,258502	0,49	0,277	1,53E-06	2	Thoc7
1,3E-10	-0,49692	0,224	0,422	1,61E-06	2	Rasa3
1,4E-10	-0,42814	0,706	0,825	1,73E-06	2	H2-DMb1
1,44E-10	-0,50724	0,196	0,39	1,79E-06	2	Cytip
1,46E-10	-0,41006	0,069	0,249	1,8E-06	2	Neat1
1,51E-10	-0,36219	0,004	0,154	1,87E-06	2	Myo1f
1,53E-10	0,272269	0,588	0,36	1,89E-06	2	Rtn3
1,54E-10	0,317501	0,816	0,611	1,9E-06	2	Hnrpdl
1,54E-10	-0,45114	0,102	0,292	1,91E-06	2	Fgd2
1,55E-10	0,309452	0,767	0,557	1,92E-06	2	Hn1
1,56E-10	0,268029	0,453	0,257	1,93E-06	2	Rsl1d1
1,59E-10	0,255373	0,331	0,161	1,96E-06	2	Nolc1
1,68E-10	-0,31264	0,016	0,174	2,08E-06	2	Arhgef3

1,69E-10	0,280543	0,8	0,585	2,09E-06	2	Sod1
1,77E-10	-0,4429	0,706	0,795	2,2E-06	2	Lsp1
1,82E-10	0,321936	0,682	0,455	2,25E-06	2	Lbr
1,96E-10	-0,47088	0,604	0,751	2,43E-06	2	Ablim1
2,21E-10	-0,3666	0,024	0,185	2,73E-06	2	Bhlhe41
2,37E-10	0,275868	0,412	0,225	2,93E-06	2	Gspt1
2,4E-10	-0,30961	0	0,144	2,97E-06	2	Plbd1
2,43E-10	0,269417	0,424	0,228	3,01E-06	2	Ndufb6
2,5E-10	0,264623	0,539	0,316	3,09E-06	2	Lsm6
2,5E-10	0,273983	0,784	0,543	3,1E-06	2	Polr1d
2,6E-10	-0,47734	0,376	0,543	3,22E-06	2	Rbm5
2,63E-10	0,29555	0,661	0,453	3,26E-06	2	Eif3c
2,94E-10	-0,40225	0,171	0,368	3,64E-06	2	Gramd1a
3,02E-10	0,3255	0,592	0,383	3,74E-06	2	Ndufa4
3,61E-10	-0,38694	0,02	0,175	4,47E-06	2	A630033H20Rik
3,9E-10	0,267822	0,714	0,493	4,83E-06	2	Nedd8
3,93E-10	-0,38316	0,02	0,174	4,86E-06	2	Kbtbd11
3,97E-10	0,251881	0,535	0,319	4,92E-06	2	Dap
4,09E-10	0,330252	0,604	0,394	5,07E-06	2	Cacybp
4,11E-10	0,301164	0,776	0,563	5,09E-06	2	Taf10
4,23E-10	0,339551	0,616	0,412	5,24E-06	2	Srsf10
4,35E-10	-0,3924	0,82	0,89	5,39E-06	2	Son
4,39E-10	-0,48408	0,053	0,223	5,43E-06	2	Irgm1
4,42E-10	0,29371	0,812	0,616	5,47E-06	2	Ywhaq
4,44E-10	0,279693	0,347	0,176	5,49E-06	2	Mybl1
4,49E-10	-0,40274	0,367	0,55	5,56E-06	2	Arhgap30
4,84E-10	0,3402	0,653	0,45	6E-06	2	Cbfb
5,01E-10	0,305876	0,633	0,422	6,21E-06	2	Csnk2b
5,02E-10	-0,38322	0,167	0,355	6,22E-06	2	Slc28a2
5,3E-10	0,278755	0,58	0,377	6,57E-06	2	Uqcrq
5,78E-10	-0,27444	0,045	0,209	7,16E-06	2	Gm5595
5,83E-10	0,268943	0,192	0,073	7,21E-06	2	Cenpf
6,28E-10	-0,48215	0,216	0,401	7,78E-06	2	Kif21b
6,31E-10	-0,32847	0,016	0,166	7,82E-06	2	Cnn3
6,34E-10	-0,50475	0,514	0,638	7,85E-06	2	Snx2
6,5E-10	0,347665	0,486	0,297	8,05E-06	2	Fkbp4
6,61E-10	0,277665	0,861	0,713	8,19E-06	2	Sfpq
6,87E-10	0,263848	0,727	0,501	8,5E-06	2	Ewsr1
7,39E-10	0,317183	0,963	0,928	9,15E-06	2	H3f3b
7,5E-10	0,293697	0,71	0,5	9,29E-06	2	Pbrm1
7,86E-10	-0,55282	0,384	0,552	9,73E-06	2	Sfrs18
7,98E-10	0,276541	0,437	0,256	9,88E-06	2	Strap
8,15E-10	-0,44419	0,416	0,593	1,01E-05	2	Ypel3
8,54E-10	-0,3831	0,078	0,248	1,06E-05	2	Ppcs
9,14E-10	0,250287	0,351	0,185	1,13E-05	2	Mrpl40
9,31E-10	-0,36883	0,188	0,373	1,15E-05	2	Uba7
9,4E-10	0,255594	0,641	0,415	1,16E-05	2	Cdv3
9,53E-10	0,276153	0,637	0,423	1,18E-05	2	Ndufa12
9,62E-10	0,27084	0,322	0,16	1,19E-05	2	Hmgcs1
1,05E-09	0,33966	0,298	0,15	1,3E-05	2	Ddt

1,05E-09	-0,39662	0,045	0,204	1,3E-05	2	Pydc3
1,15E-09	-0,38165	0,098	0,277	1,43E-05	2	Cyp4f18
1,19E-09	-0,39785	0,159	0,348	1,47E-05	2	AB124611
1,22E-09	-0,42598	0,478	0,637	1,51E-05	2	H2-Oa
1,25E-09	-0,29442	1	0,993	1,54E-05	2	Rps21
1,45E-09	-0,39741	0,224	0,413	1,8E-05	2	2310034O05Rik
1,46E-09	0,312265	0,576	0,377	1,81E-05	2	Ap2s1
1,55E-09	-0,30622	0,012	0,153	1,92E-05	2	Ccr7
1,55E-09	0,253684	0,429	0,243	1,92E-05	2	Psmd13
1,55E-09	0,250141	0,347	0,181	1,92E-05	2	Tomm40
1,61E-09	0,252161	0,384	0,207	2E-05	2	Casp2
1,68E-09	-0,48101	0,327	0,498	2,08E-05	2	Fcrl1
1,94E-09	-0,33592	0,053	0,212	2,4E-05	2	AI467606
2,04E-09	-0,36218	0,016	0,158	2,52E-05	2	Ahnak
2,04E-09	-0,36946	0,065	0,226	2,52E-05	2	Sbk1
2,06E-09	-0,5405	0,388	0,539	2,55E-05	2	Flna
2,4E-09	-0,4146	0,282	0,455	2,98E-05	2	Mll2
2,58E-09	-0,42753	0,102	0,273	3,2E-05	2	Pecam1
2,66E-09	-0,44146	0,257	0,43	3,29E-05	2	Tnrc6b
2,76E-09	0,28204	0,649	0,436	3,42E-05	2	2010107E04Rik
2,8E-09	0,258963	0,996	0,995	3,47E-05	2	Rpl3
3E-09	0,276379	0,465	0,281	3,72E-05	2	Dstn
3,01E-09	-0,43763	0,163	0,348	3,73E-05	2	Cd2
3,2E-09	0,272983	0,633	0,426	3,96E-05	2	Spcs2
3,21E-09	0,273057	0,784	0,563	3,97E-05	2	Tpm4
3,25E-09	0,280067	0,645	0,44	4,03E-05	2	Hint1
3,84E-09	0,295176	0,608	0,419	4,75E-05	2	G3bp1
4,07E-09	0,275013	0,527	0,331	5,04E-05	2	Smarca4
4,5E-09	-0,54821	0,633	0,773	5,58E-05	2	Il2rg
4,94E-09	-0,32467	0,02	0,16	6,12E-05	2	Kctd14
5,1E-09	-0,40585	0,29	0,468	6,31E-05	2	Tgfbr2
5,55E-09	-0,41522	0,322	0,498	6,87E-05	2	Mgat1
7,4E-09	-0,34767	0,057	0,215	9,16E-05	2	Hbp1
7,49E-09	0,323957	0,669	0,479	9,27E-05	2	Syngr2
7,77E-09	-0,43017	0,833	0,856	9,62E-05	2	Eif4a2
8,48E-09	-0,28891	0,045	0,193	0,000105	2	Gm2a
8,67E-09	0,31992	0,588	0,407	0,000107	2	Psma4
8,76E-09	0,314761	1	1	0,000108	2	mtNd3
9,59E-09	-0,32826	0,196	0,384	0,000119	2	Lrrc2
9,66E-09	0,251175	0,873	0,692	0,00012	2	Ube2d2a
1,06E-08	-0,43499	0,224	0,393	0,000131	2	Ppp3ca
1,07E-08	-0,35172	0,918	0,93	0,000133	2	Sf3b1
1,08E-08	-0,34842	0,11	0,28	0,000133	2	Smarca2
1,09E-08	0,25767	0,388	0,218	0,000135	2	Elavl1
1,09E-08	-0,37348	0,229	0,405	0,000135	2	Use1
1,23E-08	-0,31019	0,024	0,16	0,000153	2	Trim30b
1,34E-08	-0,32122	0,033	0,172	0,000166	2	Ddx58
1,39E-08	-0,31035	0,029	0,167	0,000172	2	Ckap4
1,39E-08	-0,3856	0,229	0,408	0,000173	2	Zbtb4
1,46E-08	0,291053	0,755	0,589	0,000181	2	Ube2i

1,58E-08	-0,34888	0,155	0,326	0,000195	2	Gmfg
1,61E-08	-0,4394	0,196	0,361	0,000199	2	Gtpbp2
1,67E-08	-0,4507	0,404	0,564	0,000206	2	Akna
1,67E-08	0,279244	0,759	0,564	0,000206	2	Caprin1
1,75E-08	0,2755	0,331	0,181	0,000216	2	Cdca4
1,92E-08	0,263433	0,645	0,439	0,000238	2	Eif3l
2,03E-08	-0,38979	0,237	0,408	0,000252	2	Vmn1r65
2,03E-08	-0,46562	0,527	0,642	0,000252	2	Ciita
2,13E-08	-0,32054	0,122	0,285	0,000264	2	Arap1
2,38E-08	-0,28075	0,041	0,179	0,000295	2	Pdcd4
2,39E-08	0,250889	0,767	0,581	0,000296	2	Gna13
2,39E-08	0,268066	0,747	0,552	0,000296	2	Ssb
2,48E-08	0,258021	0,498	0,313	0,000307	2	Ndufv2
2,52E-08	-0,33657	0,004	0,122	0,000312	2	Sspn
2,74E-08	-0,35832	0,016	0,142	0,000339	2	Serpina3g
3,07E-08	-0,34042	0,122	0,285	0,00038	2	Zmym2
3,1E-08	-0,41863	0,445	0,585	0,000384	2	Napsa
3,24E-08	-0,46034	0,743	0,868	0,000401	2	Apoe
3,3E-08	0,288253	0,771	0,595	0,000408	2	Cct2
3,35E-08	0,253311	0,494	0,309	0,000415	2	Nop10
3,41E-08	0,385464	0,412	0,256	0,000422	2	Vpreb3
3,69E-08	0,255756	0,384	0,224	0,000456	2	Nrm
3,94E-08	-0,27363	0,012	0,133	0,000487	2	Tmem154
3,98E-08	-0,41483	0,224	0,382	0,000492	2	Ikbkb
4,19E-08	-0,33441	0,11	0,27	0,000519	2	Aff3
4,6E-08	0,255069	0,539	0,362	0,00057	2	Il21r
4,81E-08	-0,27032	0,029	0,157	0,000595	2	Il27ra
4,9E-08	0,259636	0,576	0,388	0,000606	2	Slc25a4
4,93E-08	0,329777	0,335	0,188	0,000611	2	Eif5a2
6,55E-08	-0,38542	0,155	0,317	0,000811	2	Gimap7
6,78E-08	0,288732	0,669	0,485	0,000839	2	Thrap3
7,68E-08	0,265801	0,514	0,335	0,000951	2	Pkig
8,57E-08	-0,39514	0,151	0,309	0,001061	2	Cd84
8,79E-08	-0,35218	0,216	0,382	0,001088	2	Ip6k1
8,85E-08	-0,30009	0,943	0,955	0,001096	2	Arhgdib
9,4E-08	-0,35317	0,376	0,534	0,001164	2	Ptpn18
9,42E-08	0,250786	0,461	0,29	0,001167	2	Snrpb2
9,76E-08	-0,26595	0,004	0,113	0,001208	2	Fam46a
9,92E-08	-0,43644	0,241	0,404	0,001229	2	Cbfa2t3
1E-07	-0,3896	0,176	0,339	0,001243	2	Trim30a
1,01E-07	-0,44787	0,473	0,61	0,001245	2	Etnk1
1,04E-07	-0,30052	0,069	0,209	0,00129	2	Sema4b
1,04E-07	0,257994	0,461	0,288	0,001292	2	Mcmbp
1,05E-07	-0,36146	0,102	0,254	0,001301	2	Fam214a
1,07E-07	-0,3434	0,016	0,133	0,00132	2	Gbp7
1,09E-07	-0,25149	0,012	0,126	0,001345	2	Spn
1,12E-07	-0,32893	0,135	0,29	0,001384	2	Sdf2
1,18E-07	-0,2886	0,078	0,22	0,00146	2	Lgals8
1,2E-07	-0,3728	0,633	0,73	0,001485	2	Gpx1
1,29E-07	-0,25353	0,02	0,139	0,001595	2	Ski

1,31E-07	0,252894	0,735	0,557	0,001627	2	Ppp2ca
1,4E-07	0,258136	0,392	0,24	0,001729	2	Ube2m
1,41E-07	-0,28159	0,086	0,229	0,001747	2	Suv420h1
1,44E-07	0,260178	0,612	0,423	0,001779	2	Plek
1,48E-07	-0,36431	0,118	0,267	0,001827	2	Itpr1
1,57E-07	-0,29716	0,086	0,228	0,001941	2	Tle3
1,59E-07	0,428271	0,71	0,577	0,001965	2	Hspa5
1,66E-07	0,265815	0,482	0,309	0,002055	2	Sar1b
1,67E-07	-0,32495	0,143	0,295	0,002065	2	Rassf5
1,7E-07	-0,31187	0,171	0,327	0,0021	2	Dennd1c
1,91E-07	-0,31238	0,143	0,298	0,002363	2	Ppp3cc
1,97E-07	-0,38762	0,184	0,335	0,002436	2	Ssh2
2,2E-07	-0,33567	0,286	0,434	0,002728	2	Tug1
2,62E-07	-0,28378	0,086	0,226	0,003238	2	Rnf167
2,96E-07	-0,33438	0,171	0,324	0,00366	2	Trim12a
3,03E-07	-0,39712	0,253	0,407	0,003746	2	Chd7
3,24E-07	-0,31334	0,163	0,317	0,00401	2	4930523C07Rik
3,34E-07	-0,39363	0,616	0,723	0,004139	2	Akap13
3,43E-07	0,25077	1	0,991	0,004241	2	Rpl15
3,55E-07	0,251041	0,669	0,506	0,004397	2	Canx
3,7E-07	-0,3058	0,045	0,168	0,004578	2	Slfn8
3,74E-07	-0,36366	0,151	0,296	0,004633	2	Trim25
4,34E-07	-0,30573	0,212	0,368	0,005369	2	Baz2a
4,54E-07	-0,34593	0,122	0,261	0,005625	2	Mgat4a
4,89E-07	-0,32421	0,135	0,281	0,006049	2	St8sia4
5,06E-07	-0,26833	0,098	0,235	0,006264	2	Tmem229b
5,42E-07	0,267547	0,722	0,562	0,006705	2	Mat2a
6,03E-07	-0,32185	0,127	0,267	0,007468	2	Plec
6,07E-07	-0,28229	0,122	0,27	0,007511	2	Cyth4
6,2E-07	-0,40593	0,58	0,656	0,007679	2	Gdi1
6,21E-07	-0,38303	0,363	0,507	0,007685	2	Irf2
6,26E-07	-0,33134	0,131	0,273	0,007751	2	Nfat5
6,78E-07	-0,35316	0,155	0,298	0,008396	2	Parp14
7,16E-07	-0,41978	0,298	0,436	0,008858	2	Mll3
7,48E-07	-0,43529	0,237	0,373	0,009255	2	Crebrf
7,53E-07	-0,38663	0,224	0,373	0,009324	2	Zfp292
7,59E-07	-0,2924	0,069	0,197	0,009398	2	Evi2a
7,67E-07	-0,39003	0,347	0,475	0,009494	2	Slc44a2
9,03E-07	-0,32232	0,49	0,607	0,011183	2	Pfdn5
9,53E-07	0,255905	0,967	0,944	0,011804	2	Rps26
9,59E-07	-0,2973	0,078	0,207	0,011878	2	Vamp1
9,78E-07	-0,35561	0,106	0,238	0,012106	2	Phf21a
9,89E-07	-0,27423	0,065	0,189	0,012239	2	S1pr4
1E-06	-0,29077	0,069	0,196	0,012388	2	Il10rb
1,01E-06	-0,34586	0,078	0,204	0,012552	2	Kctd12
1,13E-06	-0,26199	0,902	0,94	0,014029	2	Rpl36
1,14E-06	-0,42097	0,661	0,757	0,014155	2	Bcl11a
1,22E-06	-0,38072	0,151	0,287	0,01515	2	Klf6
1,4E-06	-0,28829	0,114	0,25	0,017307	2	Hps3
1,47E-06	-0,3397	0,824	0,844	0,018169	2	Rbm39

1,49E-06	-0,26388	0,045	0,16	0,018425	2	Cacna1i
1,61E-06	-0,37043	0,339	0,478	0,019952	2	Baz2b
1,68E-06	-0,32632	0,184	0,325	0,02081	2	Fli1
1,72E-06	-0,35065	0,257	0,398	0,021288	2	Btg2
1,74E-06	-0,37352	0,71	0,779	0,021518	2	Jak1
1,8E-06	-0,33162	0,482	0,613	0,022282	2	Ddx17
1,87E-06	-0,39706	0,38	0,498	0,023168	2	Mgea5
1,89E-06	-0,3372	0,298	0,439	0,023415	2	Jmjd1c
1,94E-06	-0,42637	0,457	0,559	0,023978	2	Lmbrd1
2,02E-06	-0,25182	0,078	0,2	0,025003	2	Mir690
2,09E-06	-0,31901	0,171	0,315	0,025888	2	Sgpl1
2,13E-06	-0,275	0,012	0,107	0,026431	2	Ptpn22
2,19E-06	-0,34984	0,371	0,497	0,027087	2	Il16
2,24E-06	-0,26399	0,061	0,177	0,027721	2	5830428M24Rik
2,27E-06	-0,35656	0,396	0,528	0,028152	2	Gmip
2,34E-06	-0,2899	0,118	0,25	0,028988	2	Neu1
2,4E-06	0,2606	0,58	0,419	0,029702	2	Pdia6
2,47E-06	-0,37402	0,429	0,538	0,030557	2	Dnajc7
2,79E-06	-0,42052	0,278	0,405	0,03456	2	Ctsc
2,92E-06	-0,31727	0,282	0,422	0,036098	2	Itsn2
2,94E-06	-0,357	0,265	0,4	0,036342	2	Sptbn1
3,04E-06	-0,25397	0,078	0,198	0,037642	2	Card6
3,09E-06	-0,39117	0,212	0,352	0,038199	2	St3gal1
3,19E-06	-0,33272	0,224	0,364	0,039485	2	Mgrn1
3,24E-06	-0,33382	0,567	0,677	0,040063	2	Zc3h11a
3,26E-06	-0,33027	0,143	0,275	0,040344	2	Emp3
3,29E-06	-0,38069	0,4	0,521	0,040763	2	Gpr18
3,37E-06	-0,25427	0,012	0,104	0,041759	2	Fcrl5
3,4E-06	-0,40224	0,78	0,807	0,042122	2	Gabarap
3,5E-06	-0,34595	0,327	0,454	0,043324	2	Ogt
3,5E-06	-0,28157	0,155	0,295	0,043333	2	Cep350
3,53E-06	-0,25894	0,114	0,246	0,043666	2	Clec2g
3,57E-06	-0,27284	0,102	0,228	0,044225	2	Cnr2
3,87E-06	-0,37158	0,494	0,6	0,047871	2	Fmnl1
3,99E-06	-0,39129	0,363	0,472	0,049345	2	Dgkd
4,03E-06	0,251231	0,824	0,649	0,049911	2	Cnbp
4,49E-06	-0,2873	0,514	0,617	0,055558	2	Myh9
4,54E-06	-0,27121	0,082	0,203	0,056239	2	Mbnl2
4,54E-06	-0,34514	0,514	0,616	0,056257	2	Gltscr2
4,61E-06	-0,25585	0,094	0,214	0,057089	2	Dcaf11
5,07E-06	-0,34255	0,294	0,421	0,062724	2	Zcchc7
5,66E-06	-0,29992	0,151	0,279	0,070055	2	Pcf11
5,88E-06	-0,37826	0,567	0,638	0,072761	2	Wipf1
5,95E-06	-0,26634	0,122	0,249	0,073683	2	Acp5
6,45E-06	-0,37229	0,498	0,581	0,0799	2	H2-Eb2
6,62E-06	-0,28882	0,176	0,308	0,081918	2	Cbx7
6,8E-06	-0,32409	0,18	0,309	0,084243	2	Tmem173
7,39E-06	-0,35013	0,237	0,361	0,091499	2	Nedd9
8,81E-06	-0,40929	0,731	0,804	0,109031	2	6820431F20Rik
9,24E-06	-0,35566	0,694	0,766	0,114351	2	Grk6

9,97E-06	-0,26847	0,943	0,97	0,123438	2	Ptprcap
1,01E-05	-0,30842	0,257	0,393	0,12565	2	Sorl1
1,06E-05	-0,35826	0,359	0,474	0,130651	2	Il17ra
1,22E-05	-0,41305	0,29	0,403	0,151374	2	Trim26
1,23E-05	-0,35135	0,306	0,433	0,152103	2	Ash1l
1,23E-05	-0,39497	0,245	0,372	0,152832	2	Abhd17b
1,24E-05	-0,29174	0,224	0,362	0,154099	2	Atp6ap1
1,45E-05	-0,25705	0,135	0,255	0,179294	2	Slfn9
1,49E-05	-0,34029	0,702	0,778	0,184201	2	Rasgrp3
1,55E-05	-0,35036	0,657	0,706	0,19202	2	Ctsh
1,58E-05	-0,33861	0,437	0,545	0,196087	2	Wdfy4
1,75E-05	-0,27905	0,094	0,207	0,216559	2	Camta2
1,8E-05	-0,28827	0,22	0,351	0,222985	2	Arid4b
1,92E-05	-0,30547	0,322	0,437	0,237091	2	Gpsm3
2,08E-05	-0,3514	0,282	0,404	0,257592	2	Kdm5a
2,13E-05	-0,34971	0,09	0,202	0,263483	2	Ms4a4c
2,21E-05	-0,33124	0,298	0,419	0,273867	2	Itpr2
2,25E-05	-0,26593	0,18	0,304	0,27869	2	Cdc42se1
2,28E-05	-0,33034	0,416	0,534	0,282383	2	Med13
2,47E-05	-0,34993	0,22	0,342	0,305249	2	Pde4b
2,74E-05	-0,26059	0,229	0,354	0,339324	2	Cdc42se2
2,83E-05	-0,33509	0,698	0,735	0,349882	2	Tmbim6
2,91E-05	-0,28983	0,147	0,261	0,360149	2	Iqsec1
3,03E-05	-0,28472	0,159	0,277	0,375446	2	Ifngr2
3,05E-05	-0,26256	0,131	0,246	0,377522	2	Ints4
3,3E-05	-0,26832	0,984	0,993	0,408874	2	Srgn
3,45E-05	-0,27179	0,065	0,165	0,427028	2	Marcks
3,62E-05	-0,35663	0,612	0,676	0,448387	2	Dock8
3,87E-05	-0,28919	0,241	0,366	0,478984	2	Klf13
3,93E-05	-0,29067	0,122	0,235	0,486966	2	Cdk19
4E-05	-0,30664	0,869	0,881	0,494783	2	Apobec3
4,03E-05	-0,32258	0,494	0,593	0,49936	2	Dock2
4,19E-05	-0,26763	0,282	0,399	0,51885	2	Gpcpd1
4,26E-05	-0,29724	0,6	0,663	0,527482	2	Srsf5
5E-05	-0,31468	0,527	0,594	0,618859	2	Ddx6
5,16E-05	-0,27049	0,392	0,494	0,6387	2	Man2b1
5,21E-05	-0,33329	0,792	0,892	0,644559	2	Mir5109
5,27E-05	-0,34288	0,367	0,488	0,652216	2	Stat3
5,3E-05	-0,34966	0,273	0,389	0,656031	2	Tmem30a
5,52E-05	-0,38093	0,433	0,52	0,683074	2	Cd47
6,24E-05	-0,25583	0,963	0,974	0,773103	2	Serp1
6,76E-05	-0,32487	0,396	0,501	0,836385	2	Sh3bp5
7,14E-05	-0,26311	0,106	0,208	0,884414	2	Phc3
7,71E-05	-0,29699	0,237	0,349	0,954918	2	Adcy7
7,9E-05	-0,34695	0,351	0,465	0,978388	2	Syne1
8,28E-05	-0,2789	0,306	0,421	1	2	Cd44
8,4E-05	-0,29751	0,771	0,84	1	2	Sipa1
8,62E-05	-0,25658	0,771	0,789	1	2	Mbnl1
8,92E-05	-0,34858	0,71	0,74	1	2	Cybb
9,21E-05	-0,28061	0,612	0,698	1	2	Smap2

9,22E-05	-0,31272	0,159	0,264	1	2	Plekhm3
9,85E-05	-0,25283	0,155	0,264	1	2	Clec2d
0,000105	-0,30054	0,286	0,386	1	2	Lpgat1
0,000109	-0,28503	0,171	0,279	1	2	Atp5sl
0,00011	-0,29648	0,416	0,514	1	2	Vasp
0,000115	-0,29988	0,49	0,572	1	2	Rcsd1
0,000132	-0,29292	0,482	0,576	1	2	Pnrc2
0,000139	-0,33999	0,412	0,506	1	2	Srpk3
0,000157	-0,2528	0,102	0,198	1	2	Morc3
0,000159	-0,27997	0,596	0,66	1	2	Myo1g
0,000164	-0,25589	0,278	0,407	1	2	Zfp36
0,000171	-0,28388	0,151	0,255	1	2	Glcci1
0,000175	-0,3668	0,302	0,395	1	2	Clic4
0,000181	-0,26334	0,208	0,315	1	2	Tmem59
0,000191	-0,29642	0,155	0,26	1	2	Irf1
0,0002	-0,32316	0,437	0,508	1	2	Tnrc6a
0,000203	-0,28022	0,384	0,481	1	2	BC017643
0,000208	-0,28415	0,176	0,276	1	2	Zzef1
0,000213	-0,27449	0,212	0,312	1	2	Vamp2
0,000223	-0,28869	0,641	0,7	1	2	Ets1
0,000227	-0,25711	0,278	0,39	1	2	Elf4
0,000228	-0,30071	0,527	0,585	1	2	Prr13
0,000231	-0,30068	0,196	0,294	1	2	Aplf
0,000233	-0,36406	0,192	0,291	1	2	Sh2d3c
0,000236	-0,31263	0,2	0,295	1	2	Esyt1
0,00024	-0,28724	0,31	0,416	1	2	Zc3h7a
0,000258	-0,29982	0,469	0,568	1	2	Ankrd12
0,000269	-0,27843	0,188	0,288	1	2	Irf9
0,000275	-0,28119	0,404	0,496	1	2	Kdm2a
0,000279	-0,27418	0,857	0,895	1	2	Mef2c
0,000327	0,256064	0,531	0,423	1	2	Ddx21
0,000334	-0,27786	0,184	0,281	1	2	Gm15800
0,000335	-0,34259	0,253	0,359	1	2	Nfatc1
0,00034	-0,27951	0,237	0,335	1	2	Phf14
0,000365	-0,25328	0,196	0,295	1	2	D14Abb1e
0,000375	-0,30427	0,31	0,404	1	2	Add1
0,000422	-0,34229	0,327	0,425	1	2	Stk24
0,000435	-0,25659	0,551	0,63	1	2	Atp2a3
0,00045	-0,29012	0,38	0,468	1	2	Eif4g3
0,000458	-0,26551	0,776	0,804	1	2	Nsa2
0,00048	-0,28344	0,412	0,474	1	2	Tmem50a
0,000519	-0,28043	0,261	0,355	1	2	Pik3ca
0,00052	-0,29906	0,494	0,567	1	2	Crlf3
0,000541	-0,2953	0,224	0,319	1	2	Zmiz1
0,000543	-0,29091	0,767	0,805	1	2	Ly86
0,000551	-0,28376	0,547	0,602	1	2	Cst3
0,000573	-0,29936	0,322	0,404	1	2	Icosl
0,000599	-0,31693	0,294	0,38	1	2	Slc4a7
0,000615	-0,31022	0,343	0,43	1	2	Stk38
0,000633	-0,32369	0,253	0,338	1	2	4932438A13Rik

0,000644	-0,28676	0,269	0,359	1	2	Nxpe3
0,000647	-0,28115	0,269	0,357	1	2	Zkscan3
0,000655	-0,26185	0,249	0,348	1	2	Scd2
0,00067	-0,27786	0,176	0,267	1	2	Wdfy1
0,00072	-0,26851	0,637	0,685	1	2	Cox7a2l
0,00074	-0,25102	0,306	0,394	1	2	Zdhhc20
0,00077	-0,32927	0,376	0,45	1	2	Ubr4
0,000849	-0,28345	0,567	0,602	1	2	Ncor1
0,000895	-0,27232	0,51	0,582	1	2	Tapbp
0,00093	-0,26125	0,269	0,361	1	2	Gm7609
0,000982	-0,27862	0,457	0,543	1	2	Sqstm1
0,00121	-0,28645	0,486	0,555	1	2	Cr2
0,001477	-0,27507	0,273	0,353	1	2	Tax1bp1
0,001568	-0,25922	0,31	0,386	1	2	Zbtb7a
0,001627	-0,25509	0,257	0,342	1	2	Nisch
0,001928	-0,26227	0,188	0,274	1	2	Sfi1
0,001929	-0,25813	0,302	0,377	1	2	Itgal
0,001958	-0,26063	0,355	0,444	1	2	Ccdc82
0,002384	-0,26135	0,314	0,395	1	2	Ubn2
0,002879	-0,28956	0,314	0,391	1	2	Mia3
0,003195	-0,25801	0,322	0,398	1	2	Bin2
0,003414	-0,27882	0,453	0,524	1	2	Akap8
0,003493	-0,27614	0,465	0,537	1	2	Sat1
0,003607	-0,29411	0,706	0,758	1	2	Ptp4a3
0,00396	-0,25323	0,535	0,585	1	2	Zcchc11
0,004306	-0,31132	0,351	0,416	1	2	Rhof
0,004813	-0,25171	0,229	0,299	1	2	Galnt11
0,004914	-0,25372	0,482	0,536	1	2	Tap1
0,005472	-0,27021	0,294	0,363	1	2	Bnip3l
0,007073	-0,25954	0,188	0,254	1	2	Parm1
0,007136	-0,28118	0,188	0,258	1	2	Gbp4
0,008803	-0,25389	0,963	0,964	1	2	mtNd4l
0,008968	-0,46765	0,514	0,521	1	2	Stat1
1,57E-91	1,062268	0,5	0,052	1,95E-87	3	Ptpn22
2,33E-89	1,620162	0,691	0,134	2,88E-85	3	Plac8
1,14E-83	0,627115	0,388	0,029	1,41E-79	3	Zbtb32
1,51E-67	0,885473	0,436	0,056	1,87E-63	3	Fcrl5
8,28E-64	0,689308	0,388	0,045	1,03E-59	3	Ptprj
1,53E-59	0,68561	0,346	0,037	1,9E-55	3	Nid1
5,82E-59	0,855683	0,527	0,099	7,2E-55	3	Ahnak
7,5E-52	0,624062	0,351	0,047	9,29E-48	3	Csf2rb
2,18E-47	0,708797	0,511	0,114	2,69E-43	3	Cd38
9,7E-46	0,911506	0,489	0,117	1,2E-41	3	Marcks
4,33E-43	1,04787	0,931	0,486	5,36E-39	3	Ly6d
2,35E-40	0,684696	0,394	0,08	2,91E-36	3	Sla
1,1E-39	-1,54489	0,309	0,746	1,36E-35	3	Basp1
9,4E-38	0,85073	0,521	0,153	1,16E-33	3	Kctd12
8,37E-37	0,897137	0,814	0,381	1,04E-32	3	Sub1
1,07E-36	0,416314	0,165	0,011	1,33E-32	3	Actn1
4,95E-34	0,817836	0,617	0,231	6,13E-30	3	Fgd2

1,05E-33	1,032585	0,846	0,487	1,31E-29	3	Foxp1
9,8E-30	0,35477	0,197	0,025	1,21E-25	3	Rin3
1,66E-29	-0,89351	0,995	1	2,05E-25	3	Cfl1
3,01E-29	1,082341	0,649	0,314	3,73E-25	3	Nfatc1
3,86E-29	0,57128	0,463	0,138	4,77E-25	3	Cd97
1,25E-28	0,76376	0,766	0,42	1,55E-24	3	Psap
1,4E-28	-1,46783	0,351	0,699	1,73E-24	3	Igj
2,93E-27	0,337737	0,128	0,01	3,63E-23	3	Ccr1
5,42E-27	0,649404	0,995	0,979	6,71E-23	3	H2-K1
8,2E-27	0,847629	0,649	0,29	1,02E-22	3	Bcl2
4,3E-26	0,551656	0,346	0,092	5,32E-22	3	Mpeg1
9,77E-26	0,47772	0,282	0,062	1,21E-21	3	Fam129c
4,11E-25	-0,7549	0,926	0,987	5,09E-21	3	Laptm5
4,91E-25	0,722169	0,723	0,357	6,07E-21	3	Snn
7,13E-25	-1,01519	0,096	0,482	8,82E-21	3	Bcl6
5,6E-24	0,289098	0,176	0,025	6,93E-20	3	Mcoln2
4,47E-23	0,541106	0,282	0,071	5,54E-19	3	Emb
4,6E-23	0,505527	0,324	0,09	5,69E-19	3	Spn
6,86E-23	0,515311	0,335	0,095	8,49E-19	3	Tmem154
1,14E-22	0,31662	0,16	0,022	1,41E-18	3	Dpp4
1,28E-22	0,576914	1	0,988	1,59E-18	3	H2-D1
2,15E-22	0,439835	0,25	0,056	2,66E-18	3	Fgl2
2,87E-22	0,586329	0,66	0,311	3,56E-18	3	Map3k1
3,33E-22	0,65985	0,532	0,225	4,12E-18	3	Cyp4f18
5,14E-22	-1,11873	0,122	0,484	6,36E-18	3	Rgs13
9,54E-22	0,53316	0,33	0,098	1,18E-17	3	Cd72
1,33E-21	0,661852	0,479	0,188	1,64E-17	3	S1pr1
1,64E-21	0,363983	0,266	0,065	2,03E-17	3	Aff1
1,84E-21	0,932296	0,926	0,78	2,28E-17	3	Faim3
1,98E-21	0,265667	0,144	0,019	2,45E-17	3	Cd1d1
4,33E-21	0,629669	0,995	0,811	5,36E-17	3	Shisa5
4,38E-21	0,713699	0,835	0,545	5,42E-17	3	Bank1
6E-21	0,580733	0,399	0,14	7,43E-17	3	Bhlhe41
7,42E-21	-0,99728	0,181	0,524	9,18E-17	3	Mef2b
8,13E-21	0,774622	0,543	0,254	1,01E-16	3	Aplf
8,18E-21	0,293041	0,245	0,056	1,01E-16	3	Lilrb3
8,72E-21	0,760219	0,91	0,711	1,08E-16	3	Ly6a
1,98E-20	0,622644	0,92	0,651	2,45E-16	3	Gimap4
2,45E-20	0,262823	0,117	0,012	3,03E-16	3	Ppap2b
2,76E-20	-0,86214	0,117	0,461	3,41E-16	3	S1pr2
6,51E-20	0,533832	0,824	0,441	8,06E-16	3	Ifi203
1,51E-19	-0,69269	0,66	0,865	1,87E-15	3	Sypl
1,61E-19	0,273154	0,149	0,023	1,99E-15	3	Hck
2,29E-19	0,646648	0,995	0,97	2,84E-15	3	Malat1
2,8E-19	0,738471	0,66	0,361	3,47E-15	3	Gns
2,82E-19	0,637322	0,793	0,507	3,49E-15	3	Gimap3
2,86E-19	0,353572	0,335	0,104	3,54E-15	3	Plbd1
4,05E-19	0,310052	0,218	0,05	5,01E-15	3	Aldh3b1
8,8E-19	0,677303	0,745	0,467	1,09E-14	3	Itga4
1,01E-18	-0,88566	0,133	0,457	1,25E-14	3	Gcsam

1,13E-18	-0,85244	0,473	0,719	1,4E-14	3	Txn1
1,3E-18	0,558573	0,782	0,433	1,61E-14	3	Pml
1,58E-18	0,250376	0,207	0,046	1,96E-14	3	Adam19
2,89E-18	0,580613	0,819	0,465	3,57E-14	3	Mndal
3,45E-18	0,458415	0,388	0,143	4,27E-14	3	Inpp5f
4,28E-18	0,320062	0,181	0,036	5,3E-14	3	Arhgap24
5,76E-18	-0,69527	0,457	0,714	7,13E-14	3	Arpc5l
1,03E-17	0,614378	0,537	0,266	1,28E-13	3	Jhdm1d
1,05E-17	0,328631	0,218	0,054	1,3E-13	3	Sirpa
1,21E-17	-0,88523	0,234	0,532	1,49E-13	3	Aicda
1,27E-17	0,516353	0,713	0,376	1,57E-13	3	Klf2
1,29E-17	0,44347	1	0,995	1,6E-13	3	B2m
3,14E-17	0,430847	0,277	0,083	3,88E-13	3	Ephx1
4,72E-17	0,794878	0,883	0,721	5,84E-13	3	Cybb
8,73E-17	0,391017	0,362	0,132	1,08E-12	3	Arhgef3
1,3E-16	0,583227	0,665	0,392	1,62E-12	3	S100a11
1,62E-16	0,348384	0,207	0,052	2,01E-12	3	Cd300lf
1,96E-16	0,467553	0,516	0,241	2,42E-12	3	Man1a
3,71E-16	0,652239	0,899	0,728	4,59E-12	3	Fcrla
5,78E-16	0,567883	0,75	0,455	7,16E-12	3	Fam65b
6,62E-16	-0,48062	0,973	0,998	8,19E-12	3	Tmsb10
1,07E-15	0,300104	0,218	0,059	1,32E-11	3	Rilpl2
1,85E-15	0,507161	0,473	0,22	2,29E-11	3	Notch2
2,3E-15	0,473853	0,654	0,364	2,84E-11	3	Dgka
3,19E-15	0,484523	0,59	0,307	3,95E-11	3	S100a10
3,55E-15	0,436263	0,33	0,123	4,4E-11	3	Trim30b
3,88E-15	0,609565	0,654	0,401	4,8E-11	3	Ncf1
4,08E-15	0,387566	0,17	0,039	5,05E-11	3	Dnase1l3
4,62E-15	-0,53709	0,021	0,289	5,71E-11	3	Nuggc
5,3E-15	-1,10151	0,218	0,487	6,56E-11	3	Hmgb2
7,86E-15	0,282051	0,149	0,03	9,73E-11	3	Tmem163
8,1E-15	-0,71095	0,356	0,608	1E-10	3	Mbd2
9,26E-15	0,376995	1	0,999	1,15E-10	3	Ly6e
1,17E-14	0,371239	0,16	0,036	1,45E-10	3	S100a6
1,24E-14	0,388244	0,298	0,106	1,53E-10	3	Ski
1,56E-14	0,52586	0,324	0,125	1,93E-10	3	Grn
1,8E-14	0,505662	0,665	0,383	2,22E-10	3	Add3
2,38E-14	0,487881	0,644	0,375	2,95E-10	3	Ubl3
4,54E-14	0,52081	0,463	0,218	5,62E-10	3	Cd55
6,22E-14	0,512296	0,83	0,552	7,71E-10	3	Cmah
7,49E-14	-0,642	0,66	0,797	9,27E-10	3	Anp32b
1,04E-13	0,549015	0,495	0,261	1,28E-09	3	Esyt1
1,1E-13	-0,62638	0,755	0,887	1,36E-09	3	Stt3b
1,19E-13	0,435085	0,67	0,38	1,48E-09	3	Capg
1,23E-13	-0,65674	0,335	0,596	1,52E-09	3	Tmem131
1,41E-13	1,30572	0,979	0,939	1,74E-09	3	A630089N07Rik
1,65E-13	0,586228	0,91	0,752	2,04E-09	3	Prkcb
2,06E-13	0,416971	0,436	0,205	2,56E-09	3	Neat1
2,42E-13	0,422255	0,271	0,097	2,99E-09	3	Ceacam1
3,22E-13	-0,58541	0,085	0,34	3,99E-09	3	Eaf2

3,86E-13	0,27372	0,266	0,092	4,77E-09	3	Fam43a
5,93E-13	-0,88104	0,601	0,753	7,35E-09	3	Hmgn2
5,98E-13	-0,6074	0,154	0,412	7,4E-09	3	Anxa2
6,96E-13	-0,47954	0,851	0,939	8,62E-09	3	Psmb8
8,36E-13	0,422832	0,42	0,194	1,03E-08	3	Arhgef18
1,13E-12	0,444189	0,606	0,366	1,4E-08	3	2310034O05Rik
1,24E-12	0,337196	0,229	0,076	1,54E-08	3	Prkca
1,32E-12	0,405565	0,314	0,127	1,63E-08	3	Cacna1i
1,66E-12	0,566373	0,66	0,426	2,05E-08	3	Nfkb1
1,72E-12	-0,66897	0,447	0,663	2,13E-08	3	Erp44
1,89E-12	0,458402	0,489	0,261	2,34E-08	3	Fcgr2b
2,37E-12	0,369588	0,356	0,155	2,93E-08	3	Itm2c
2,97E-12	1,421432	1	0,967	3,67E-08	3	Gm17821
3,13E-12	0,520634	0,713	0,493	3,88E-08	3	Itm2b
4,05E-12	-0,50258	0,037	0,268	5,01E-08	3	Parm1
4,07E-12	0,415707	0,388	0,187	5,04E-08	3	Sbk1
4,85E-12	0,456911	0,957	0,809	6,01E-08	3	Macf1
5,47E-12	-0,55348	0,213	0,456	6,77E-08	3	Phf6
6,99E-12	-0,6762	0,367	0,6	8,66E-08	3	Klhl6
7,05E-12	0,389786	0,287	0,114	8,73E-08	3	Ahcyl2
7,49E-12	-0,64076	0,309	0,551	9,27E-08	3	Mtf2
7,52E-12	-0,61863	0,229	0,477	9,31E-08	3	Dck
8,26E-12	-0,59073	0,436	0,656	1,02E-07	3	Hmgn1
8,99E-12	-0,659	0,165	0,411	1,11E-07	3	Lpp
1,07E-11	0,452993	0,585	0,347	1,33E-07	3	Cyb561a3
1,21E-11	0,28031	0,149	0,038	1,5E-07	3	Zfp385a
1,21E-11	-0,47889	0,085	0,321	1,5E-07	3	Rassf6
1,26E-11	0,336857	0,255	0,095	1,55E-07	3	Zmynd11
1,29E-11	0,394717	0,447	0,229	1,6E-07	3	Aff3
1,37E-11	-0,58712	0,67	0,808	1,7E-07	3	Pou2af1
1,4E-11	0,287307	0,197	0,061	1,74E-07	3	Faah
1,63E-11	0,274271	0,17	0,048	2,02E-07	3	Il10ra
1,64E-11	-0,92865	0,176	0,405	2,03E-07	3	Stmn1
1,71E-11	0,390978	0,356	0,163	2,12E-07	3	Evi2a
1,9E-11	-0,54013	0,505	0,687	2,35E-07	3	Cox6a1
1,94E-11	0,415526	0,782	0,531	2,4E-07	3	Samd9l
2,04E-11	-0,42306	0,952	0,99	2,53E-07	3	Gnas
2,12E-11	-0,50137	0,654	0,803	2,62E-07	3	Slc25a5
2,47E-11	0,348589	0,452	0,228	3,06E-07	3	Cdkn1b
3,19E-11	-0,59654	0,261	0,477	3,95E-07	3	Rfc1
3,6E-11	-0,53047	0,612	0,79	4,45E-07	3	Gapdh
3,87E-11	-0,50974	0,186	0,431	4,79E-07	3	Dcaf12
4,49E-11	-0,43506	0,074	0,294	5,56E-07	3	Smagp
4,61E-11	-0,51338	0,543	0,733	5,71E-07	3	Ube2d2a
4,64E-11	0,388977	0,516	0,288	5,75E-07	3	Malt1
4,76E-11	-0,47598	0,128	0,361	5,89E-07	3	Fas
4,82E-11	0,38315	0,293	0,123	5,96E-07	3	Rnf13
5,15E-11	-0,63079	0,053	0,264	6,38E-07	3	Mcm5
5,36E-11	0,263003	0,995	1	6,63E-07	3	Rps24
5,84E-11	-0,71383	0,234	0,456	7,23E-07	3	8430410A17Rik

1,13E-10	-0,48341	0,713	0,825	1,39E-06	3	Hnrnpa3
1,2E-10	0,371703	0,261	0,103	1,48E-06	3	Gbp7
1,39E-10	-0,54552	0,335	0,561	1,72E-06	3	Apobec1
1,48E-10	0,309503	0,255	0,1	1,83E-06	3	Gm11648
1,67E-10	0,49573	0,644	0,444	2,07E-06	3	Pld4
1,97E-10	-0,63499	0,91	0,948	2,44E-06	3	Ptma
2,02E-10	-0,52847	0,335	0,563	2,5E-06	3	Ppp4r2
2,02E-10	-0,45673	0,064	0,274	2,5E-06	3	Ogfrl1
2,04E-10	0,50726	0,574	0,364	2,53E-06	3	Cbfa2t3
2,07E-10	0,254381	0,287	0,12	2,56E-06	3	Ccr7
2,11E-10	-0,39573	0,963	0,985	2,62E-06	3	Arpc2
2,23E-10	-0,44891	0,676	0,854	2,76E-06	3	Irf8
2,36E-10	-0,43297	0,069	0,28	2,93E-06	3	Emid1
2,52E-10	-0,62781	0,468	0,647	3,12E-06	3	Ran
2,64E-10	-0,49935	0,239	0,455	3,27E-06	3	Glrx3
2,65E-10	-0,41231	0,059	0,263	3,29E-06	3	Ccnd3
2,74E-10	-0,44103	0,335	0,578	3,39E-06	3	St6gal1
3,46E-10	-0,4964	0,314	0,551	4,28E-06	3	Marcksl1
4,17E-10	-0,64178	0,505	0,659	5,16E-06	3	Top1
4,59E-10	-0,42723	0,915	0,962	5,69E-06	3	Pfn1
4,8E-10	-0,38245	0,027	0,216	5,94E-06	3	Mybl1
5,13E-10	0,332463	0,452	0,247	6,35E-06	3	Rassf4
5,6E-10	0,432104	0,91	0,854	6,94E-06	3	Mcl1
6,09E-10	0,279008	0,186	0,063	7,54E-06	3	Dopey2
6,48E-10	-0,45035	0,149	0,366	8,03E-06	3	Dap
7,06E-10	0,35988	0,34	0,163	8,74E-06	3	Il10rb
8,06E-10	-0,53911	0,41	0,616	9,98E-06	3	Bach2
8,13E-10	0,366889	0,378	0,193	1,01E-05	3	Tle3
8,68E-10	-0,32382	0,064	0,272	1,07E-05	3	Plxnb2
9,64E-10	0,465277	0,266	0,112	1,19E-05	3	Ccnd2
9,74E-10	-0,43793	0,277	0,508	1,21E-05	3	Parp1
9,82E-10	-0,37428	0,09	0,303	1,22E-05	3	Mtmr14
1,05E-09	-0,47638	0,207	0,424	1,3E-05	3	Helq
1,05E-09	0,254822	0,223	0,086	1,3E-05	3	Gucd1
1,13E-09	-0,47548	0,654	0,805	1,4E-05	3	Trp53i11
1,36E-09	0,39442	0,819	0,666	1,69E-05	3	Scd1
1,53E-09	-0,51744	0,473	0,643	1,89E-05	3	Ldha
1,65E-09	-0,4086	0,106	0,315	2,04E-05	3	Bzw2
1,86E-09	0,321692	0,298	0,134	2,31E-05	3	Ckap4
1,9E-09	0,434404	0,654	0,436	2,35E-05	3	Evi2b
2,04E-09	0,308743	0,367	0,185	2,52E-05	3	Glt25d1
2,11E-09	0,298382	0,213	0,082	2,62E-05	3	Stoml1
2,12E-09	-0,28606	1	1	2,62E-05	3	Actb
2,29E-09	0,398759	0,42	0,238	2,83E-05	3	Nfat5
2,67E-09	-0,43071	0,899	0,949	3,3E-05	3	Nap1l1
2,82E-09	-0,39362	0,064	0,259	3,49E-05	3	Mcm2
3,37E-09	0,550569	0,553	0,371	4,17E-05	3	Ctsc
3,41E-09	0,384429	0,479	0,286	4,23E-05	3	Tmod3
4,13E-09	-0,47966	0,622	0,807	5,11E-05	3	Ccdc50
4,26E-09	-0,36821	0,91	0,962	5,27E-05	3	Myl6

5,12E-09	-0,43306	0,681	0,804	6,33E-05	3	Atp5a1
5,7E-09	0,343345	0,372	0,192	7,06E-05	3	Tcp11l2
6,51E-09	-0,39443	0,84	0,928	8,05E-05	3	Rbm3
7,48E-09	-0,50734	0,319	0,517	9,26E-05	3	Pgk1
8,16E-09	-0,47314	0,34	0,527	0,000101	3	Erh
8,19E-09	-0,4341	0,149	0,349	0,000101	3	Lpin2
8,34E-09	-0,51211	0,729	0,804	0,000103	3	Hmgb1
8,5E-09	-0,38678	0,186	0,404	0,000105	3	Scimp
9,04E-09	0,344846	0,362	0,19	0,000112	3	Herc1
9,59E-09	0,416584	0,532	0,348	0,000119	3	Cytip
1,06E-08	-0,48833	0,559	0,707	0,000131	3	Rhoh
1,13E-08	0,391825	0,511	0,317	0,00014	3	Scd2
1,16E-08	-0,44271	0,383	0,581	0,000143	3	Psmb7
1,25E-08	0,427808	0,702	0,553	0,000155	3	Napsa
1,59E-08	-0,41009	0,761	0,854	0,000197	3	Limd2
1,7E-08	0,330248	0,495	0,293	0,00021	3	Lrrc33
1,94E-08	0,267079	0,165	0,057	0,00024	3	Relt
1,94E-08	-0,32692	0,968	0,994	0,00024	3	H3f3a
1,97E-08	-0,36583	0,936	0,969	0,000244	3	Calm1
2,12E-08	-0,43416	0,707	0,791	0,000263	3	Clic1
2,35E-08	-0,37732	0,91	0,962	0,000291	3	Cd22
2,5E-08	-0,39137	0,41	0,61	0,00031	3	Usp7
2,71E-08	0,374393	0,606	0,421	0,000335	3	Ogt
2,89E-08	0,433344	0,543	0,37	0,000357	3	Vmn1r65
2,99E-08	-0,52441	0,606	0,728	0,000371	3	Hnrnpab
3,02E-08	0,416246	0,777	0,575	0,000374	3	Zbtb20
3,03E-08	-0,42779	0,223	0,423	0,000376	3	Odc1
3,13E-08	-0,46856	0,628	0,762	0,000387	3	Bptf
3,14E-08	-0,30209	0,053	0,227	0,000389	3	Osbpl3
3,42E-08	-0,42234	0,319	0,515	0,000424	3	Ssbp3
3,47E-08	-0,43814	0,452	0,612	0,00043	3	Lrmp
3,62E-08	-0,43905	0,239	0,443	0,000448	3	Mbd4
3,75E-08	0,476263	0,654	0,503	0,000464	3	Pyhin1
4,04E-08	-0,38125	0,117	0,305	0,0005	3	Xrcc1
4,05E-08	-0,42357	0,862	0,919	0,000501	3	Ppia
4,07E-08	-0,3982	0,144	0,336	0,000504	3	Naa40
4,18E-08	0,374876	0,92	0,801	0,000517	3	Btg1
4,33E-08	-0,30167	0,027	0,184	0,000536	3	Cpne5
4,61E-08	0,387385	0,819	0,715	0,000571	3	Iqgap1
4,71E-08	0,394787	0,654	0,463	0,000584	3	Hsp90b1
4,96E-08	0,299846	0,197	0,078	0,000614	3	Rb1
5,05E-08	0,299114	0,213	0,09	0,000625	3	Ms4a6c
5,11E-08	-0,28375	0,037	0,197	0,000632	3	Neil1
5,34E-08	-0,43545	0,707	0,776	0,000662	3	Atp5b
5,42E-08	0,41235	0,745	0,639	0,000672	3	Tcf4
6,14E-08	0,266175	1	1	0,000761	3	Cd79a
6,17E-08	-0,3574	0,106	0,287	0,000763	3	Havcr1
7,13E-08	-0,39379	0,426	0,61	0,000883	3	Eml4
7,26E-08	-0,46335	0,324	0,52	0,000899	3	Pitpnc1
8,13E-08	1,005726	0,628	0,445	0,001007	3	A130077B15Rik

8,38E-08	-0,63557	0,415	0,573	0,001038	3	Dusp2
8,43E-08	-0,39436	0,165	0,362	0,001044	3	Ell3
8,47E-08	-0,34307	0,207	0,409	0,001048	3	Pnp
8,82E-08	-0,32077	0,043	0,2	0,001093	3	Cobl
9,23E-08	0,443254	0,426	0,266	0,001142	3	Ctsb
9,97E-08	1,423671	0,787	0,638	0,001234	3	Vmn2r55
1,03E-07	-0,33529	0,059	0,222	0,001273	3	H2afx
1,03E-07	-0,4371	0,303	0,499	0,001278	3	Cd86
1,23E-07	-0,36553	0,309	0,509	0,001517	3	Arpc3
1,33E-07	-0,35353	0,037	0,188	0,001647	3	H1fx
1,39E-07	-0,34341	0,766	0,864	0,001719	3	Arpc4
1,43E-07	0,291122	0,324	0,173	0,001774	3	Cyfip1
1,44E-07	0,341169	0,846	0,725	0,001786	3	Siglecg
1,45E-07	0,401768	0,569	0,386	0,001796	3	Itpr2
1,46E-07	-0,33463	0,941	0,976	0,001807	3	Hspa8
1,51E-07	1,450581	0,91	0,789	0,001864	3	Grk4
1,63E-07	0,335108	0,25	0,119	0,002015	3	Prkce
1,71E-07	0,314362	0,319	0,171	0,002118	3	Abcg1
1,76E-07	-0,39529	0,415	0,592	0,002185	3	Cyfip2
1,87E-07	-0,37859	0,266	0,464	0,00232	3	Akt1
1,99E-07	-0,25001	0,027	0,172	0,002467	3	Ada
2,02E-07	-0,33233	0,814	0,868	0,002505	3	Tnfrsf13c
2,05E-07	-0,36546	0,404	0,604	0,002534	3	Hdac7
2,09E-07	0,427623	0,553	0,378	0,002583	3	Trim34a
2,18E-07	0,273305	0,346	0,184	0,002693	3	Scml4
2,42E-07	-0,38141	0,191	0,37	0,00299	3	Atad1
2,48E-07	0,379614	0,883	0,784	0,003076	3	Mycbp2
2,49E-07	-0,35228	0,207	0,392	0,003086	3	Dbi
2,49E-07	-0,42859	0,383	0,548	0,003087	3	Lpxn
2,51E-07	-0,63145	0,245	0,41	0,003103	3	Mcm6
2,66E-07	0,290953	0,298	0,156	0,003297	3	H2-K2
2,68E-07	0,323663	0,59	0,389	0,003321	3	Anxa6
2,81E-07	-0,79181	0,17	0,33	0,003474	3	Mki67
2,86E-07	-0,38894	0,415	0,578	0,003539	3	Tra2b
2,92E-07	0,527516	0,255	0,121	0,003613	3	F830016B08Rik
2,95E-07	-0,41688	0,622	0,738	0,003651	3	Atp5g3
3,12E-07	-0,34045	0,096	0,262	0,003858	3	Aars
3,19E-07	-0,32212	0,186	0,378	0,003954	3	Rfc2
3,21E-07	-0,35046	0,122	0,298	0,003969	3	Lipc
3,35E-07	-0,43194	0,356	0,518	0,004144	3	Syngr2
3,35E-07	-0,75407	0,564	0,693	0,004149	3	H2afz
3,46E-07	1,258223	1	0,991	0,004282	3	Kcnq1ot1
3,73E-07	-0,3808	0,5	0,656	0,00462	3	Ywhaq
3,77E-07	-0,2962	0,037	0,182	0,004667	3	Lcp2
4E-07	-0,34563	0,399	0,586	0,004958	3	Lat2
4,5E-07	-0,43057	0,223	0,397	0,005571	3	Slbp
4,56E-07	0,391182	0,431	0,274	0,005649	3	Cd84
4,73E-07	-0,36574	0,223	0,401	0,005855	3	Il21r
4,74E-07	-0,40843	0,096	0,253	0,005871	3	Mcm3
5,25E-07	-0,29702	0,059	0,213	0,006505	3	Prim1

5,65E-07	-0,35391	0,697	0,791	0,006993	3	Atp5g2
5,89E-07	0,288122	0,314	0,169	0,007297	3	Tuba4a
5,97E-07	-0,29292	0,074	0,232	0,00739	3	Enpp1
6,15E-07	-0,33426	0,287	0,462	0,00761	3	Azin1
6,42E-07	-0,36059	0,202	0,388	0,007951	3	Asap1
6,55E-07	0,275137	0,484	0,307	0,008109	3	Rasgrp2
6,69E-07	-0,30089	0,144	0,315	0,008278	3	Akap2
6,84E-07	0,291531	0,75	0,585	0,008467	3	Ddx3x
7E-07	-0,27863	0,096	0,263	0,008671	3	Sav1
7,08E-07	0,323637	0,314	0,172	0,008765	3	Morc3
7,11E-07	-0,2681	0,08	0,238	0,008801	3	Ralgds
7,47E-07	-0,35079	0,793	0,9	0,009244	3	Mef2c
7,59E-07	-0,36037	0,101	0,259	0,009401	3	Dut
7,81E-07	0,273825	0,309	0,169	0,009669	3	Slfn2
8,47E-07	-0,33933	0,096	0,254	0,010492	3	Sh2b2
8,69E-07	-0,35183	0,957	0,986	0,010757	3	Ucp2
9,47E-07	-0,36998	0,138	0,306	0,011729	3	Rnf157
9,6E-07	0,295304	0,372	0,218	0,011887	3	Acp5
9,78E-07	0,289925	0,388	0,233	0,012112	3	Itpr1
1,03E-06	-0,45464	0,085	0,234	0,012754	3	Cks2
1,06E-06	-0,28625	0,957	0,984	0,013178	3	Hnrnpa2b1
1,09E-06	-0,4369	0,202	0,373	0,013475	3	Pafah1b3
1,09E-06	-0,39403	0,787	0,863	0,013506	3	Eif5a
1,09E-06	-0,36521	0,282	0,451	0,013534	3	Pomp
1,11E-06	-0,30352	0,261	0,437	0,013733	3	Psmb3
1,12E-06	-0,28508	0,122	0,292	0,013824	3	Epb4.1
1,13E-06	-0,36514	0,271	0,455	0,013953	3	Cdk2ap2
1,18E-06	1,033893	0,686	0,543	0,014577	3	Gm10785
1,19E-06	-0,34828	0,186	0,359	0,014767	3	Bcl7a
1,21E-06	0,279985	0,277	0,142	0,014993	3	Kbtbd11
1,22E-06	-0,35566	0,064	0,207	0,015057	3	Cdca7
1,25E-06	-0,27023	0,064	0,208	0,015528	3	Rtca
1,26E-06	0,414827	0,5	0,341	0,01559	3	Abhd17b
1,3E-06	0,590978	0,904	0,847	0,016133	3	Apoe
1,36E-06	-0,29071	0,037	0,173	0,016787	3	Uhrf1
1,38E-06	-0,29464	0,862	0,901	0,017075	3	Grb2
1,38E-06	0,269142	0,25	0,126	0,017092	3	Gpr174
1,4E-06	0,34167	0,548	0,381	0,017335	3	Rasa3
1,48E-06	0,404818	0,793	0,647	0,018326	3	Syk
1,55E-06	-0,35185	0,191	0,359	0,019162	3	Sema7a
1,56E-06	-0,34108	0,681	0,796	0,01934	3	Pkm
1,75E-06	0,286054	0,431	0,264	0,021608	3	Chd2
1,77E-06	0,341591	0,394	0,244	0,021968	3	Emp3
1,78E-06	-0,38892	0,112	0,27	0,022085	3	Asf1b
1,81E-06	-0,33521	0,42	0,586	0,022351	3	Hnrnpd
1,84E-06	-0,30481	0,122	0,284	0,022831	3	Slamf1
1,85E-06	-0,26769	0,059	0,199	0,022872	3	Vopp1
1,88E-06	0,255126	1	0,998	0,023307	3	Cd19
1,89E-06	-0,40329	0,202	0,365	0,023389	3	Ehd3
1,95E-06	-0,27407	1	0,997	0,024194	3	Actg1

1,96E-06	-0,34214	0,383	0,555	0,024236	3	Psma7
2,07E-06	0,345971	0,628	0,474	0,02562	3	Sh3bp5
2,09E-06	0,259373	0,245	0,125	0,025826	3	Chd3
2,18E-06	-0,25692	0,037	0,169	0,026948	3	Smco4
2,27E-06	-0,3245	0,16	0,329	0,028154	3	Cdc25b
2,36E-06	-0,7546	0,489	0,626	0,029203	3	Pcna
2,46E-06	0,25795	0,41	0,249	0,030451	3	Itgb7
2,47E-06	0,295879	0,649	0,487	0,030555	3	Gimap8
2,53E-06	0,33345	0,473	0,32	0,031338	3	Adcy7
2,54E-06	-0,32878	0,202	0,379	0,031436	3	Ube2h
2,67E-06	0,287294	0,489	0,322	0,033021	3	Cdc42se2
2,69E-06	-0,35102	0,33	0,495	0,033277	3	Rbbp4
2,7E-06	0,287202	0,553	0,371	0,033408	3	Stk10
2,8E-06	-0,31959	0,638	0,782	0,034618	3	Rasgrp3
2,83E-06	0,341492	0,66	0,518	0,034996	3	Wdfy4
2,85E-06	-0,27863	0,09	0,241	0,035221	3	Ildr1
2,99E-06	-0,30034	0,181	0,358	0,036962	3	Brwd1
3,07E-06	0,395657	0,617	0,458	0,038033	3	Slc12a6
3,29E-06	0,255363	0,995	0,994	0,040742	3	Rps18
3,42E-06	-0,34738	0,032	0,156	0,04233	3	Cenpe
3,47E-06	-0,36464	0,34	0,508	0,042947	3	Herpud1
3,49E-06	-0,26668	0,037	0,164	0,04318	3	Hells
3,57E-06	-0,37137	0,463	0,62	0,044251	3	Gna13
3,81E-06	-0,31006	0,096	0,241	0,047215	3	Gadd45b
3,88E-06	-0,3414	0,186	0,349	0,048015	3	Rapgef4
3,89E-06	0,342225	0,441	0,281	0,048173	3	Sgpl1
3,9E-06	0,322749	0,739	0,605	0,048305	3	Tbc1d10c
3,96E-06	-0,37226	0,702	0,785	0,049082	3	Srsf2
3,98E-06	-0,33438	0,186	0,345	0,049293	3	Stap1
4,03E-06	-0,39662	0,191	0,346	0,049867	3	Mcm7
4,05E-06	-0,36454	0,096	0,24	0,050164	3	Slpi
4,16E-06	-0,34387	0,064	0,202	0,051466	3	Dhfr
4,17E-06	-0,36075	0,207	0,373	0,051668	3	Atp8a1
4,29E-06	-0,28351	0,069	0,206	0,053136	3	Endou
4,36E-06	-0,32473	0,335	0,5	0,053988	3	Hspa4
4,6E-06	-0,49949	0,356	0,49	0,056913	3	Hpse
4,73E-06	-0,33121	0,239	0,392	0,058572	3	Sdhb
4,74E-06	-0,63182	0,101	0,241	0,058741	3	2810417H13Rik
4,87E-06	-0,35512	0,229	0,392	0,060333	3	2700029M09Rik
4,92E-06	0,323931	0,271	0,149	0,060954	3	Rbms1
4,94E-06	-0,28243	0,112	0,26	0,061113	3	Mprip
5,13E-06	0,277308	1	0,988	0,063559	3	Serinc3
5,14E-06	0,262517	0,505	0,339	0,063608	3	Myl12b
5,24E-06	-0,2506	0,101	0,256	0,064821	3	Fut8
5,99E-06	-0,26907	0,08	0,222	0,07413	3	Pdk3
6,11E-06	-0,36913	0,367	0,519	0,075637	3	Rbbp7
6,11E-06	-0,47505	0,324	0,464	0,075698	3	Syne1
6,38E-06	-0,36262	0,303	0,467	0,079042	3	Psip1
6,39E-06	-0,2663	0,085	0,229	0,079123	3	4833439L19Rik
7,44E-06	0,283858	0,367	0,231	0,092168	3	Mgat4a

7,51E-06	-0,33683	0,207	0,366	0,092943	3	Ublcp1
7,63E-06	-0,29926	0,601	0,744	0,094422	3	Ptk2b
7,7E-06	0,291689	0,676	0,53	0,095345	3	Akna
7,71E-06	-0,2796	0,149	0,303	0,095501	3	Sh3pxd2a
7,85E-06	0,393087	0,622	0,497	0,09723	3	Cd47
7,96E-06	-0,34122	0,181	0,334	0,098596	3	Dnmt1
8,05E-06	0,369394	0,628	0,506	0,099688	3	Tmem123
8,24E-06	-0,34801	0,574	0,673	0,102027	3	Prdx1
8,38E-06	-0,31596	0,106	0,247	0,103706	3	Gsn
8,8E-06	0,390042	0,585	0,451	0,108954	3	Tlr1
9,12E-06	-0,29218	0,074	0,204	0,112936	3	Btnl2
9,49E-06	-0,31102	0,165	0,319	0,11747	3	Dstn
9,54E-06	-0,27058	0,234	0,397	0,118048	3	Snrpf
9,73E-06	0,330295	0,596	0,441	0,120477	3	Filip1l
1,02E-05	0,348976	0,883	0,792	0,125808	3	Ly86
1,03E-05	-0,28736	0,106	0,253	0,127357	3	Slamf7
1,08E-05	-0,34843	0,112	0,249	0,133861	3	Mcm4
1,09E-05	0,355485	0,383	0,24	0,134479	3	Gm8369
1,15E-05	-0,2722	0,255	0,418	0,142266	3	Aff4
1,23E-05	-0,39648	0,399	0,558	0,152397	3	Fchsd2
1,23E-05	-0,32165	0,452	0,614	0,152636	3	Hnrnpa0
1,26E-05	0,322705	0,319	0,191	0,156603	3	Rbpj
1,32E-05	0,273001	0,202	0,101	0,162934	3	5830416P10Rik
1,32E-05	0,262972	0,191	0,092	0,163671	3	Kat6b
1,35E-05	0,314419	0,729	0,598	0,166558	3	Mll5
1,35E-05	-0,30695	0,516	0,657	0,167437	3	Atp5e
1,36E-05	0,329372	0,42	0,289	0,168639	3	Rasgrp1
1,37E-05	0,33593	0,239	0,128	0,16952	3	Nedd4
1,38E-05	0,262318	0,287	0,167	0,170485	3	Gm13157
1,39E-05	-0,25554	0,08	0,209	0,172534	3	Amz2
1,41E-05	0,262207	0,324	0,193	0,174503	3	Lmo4
1,44E-05	-0,34853	0,309	0,454	0,178004	3	Anp32e
1,51E-05	-0,34727	0,213	0,35	0,187544	3	H2afv
1,52E-05	-0,29794	0,383	0,526	0,188643	3	Shfm1
1,56E-05	-0,28695	0,457	0,591	0,192612	3	Oaz1
1,6E-05	-0,28475	0,074	0,202	0,197726	3	Pla2g12a
1,69E-05	-0,30862	0,553	0,689	0,208746	3	Purb
1,74E-05	0,271722	0,426	0,288	0,214974	3	Tmem59
1,82E-05	-0,26652	0,894	0,936	0,225136	3	H3f3b
1,86E-05	-0,29753	0,771	0,824	0,230709	3	Hsp90aa1
1,86E-05	-0,31653	0,128	0,264	0,230771	3	Psat1
1,9E-05	0,258409	0,84	0,738	0,234627	3	Hmha1
1,92E-05	-0,31613	0,181	0,328	0,237112	3	Whsc1
1,92E-05	0,252819	0,372	0,233	0,23745	3	Iqsec1
1,92E-05	0,286872	0,186	0,091	0,237467	3	Ptpn12
2,09E-05	-0,36544	0,787	0,868	0,258866	3	Cnn2
2,18E-05	-0,32701	0,553	0,672	0,270004	3	Cox6c
2,21E-05	0,300188	0,33	0,2	0,274129	3	Lgals9
2,24E-05	-0,29511	0,473	0,602	0,27677	3	Taf10
2,29E-05	-0,28917	0,356	0,496	0,283992	3	Psma2

2,32E-05	0,379932	0,601	0,484	0,286874	3	Wdr92
2,34E-05	1,027134	0,585	0,452	0,289315	3	Zfp71-rs1
2,37E-05	0,327952	0,537	0,389	0,293759	3	Mylip
2,41E-05	0,540908	0,633	0,495	0,298683	3	Vim
2,43E-05	-0,29172	0,282	0,422	0,301069	3	Plekho1
2,56E-05	-0,27404	0,979	0,994	0,316603	3	Pabpc1
2,7E-05	-0,31081	0,25	0,405	0,334118	3	Rev3l
2,72E-05	0,358199	0,372	0,243	0,336525	3	Wdfy1
2,72E-05	0,252942	0,457	0,318	0,337221	3	Slc28a2
2,83E-05	-0,2598	0,362	0,529	0,350138	3	Snrpb
2,91E-05	-0,27487	0,239	0,391	0,360839	3	Ube2n
2,94E-05	-0,27974	0,229	0,375	0,364433	3	Cwc15
2,96E-05	-0,25778	0,378	0,539	0,365924	3	Syvn1
2,96E-05	0,350676	0,92	0,84	0,366172	3	Pou2f2
3E-05	0,312305	0,468	0,338	0,371415	3	Klf13
3,03E-05	-0,3456	0,564	0,674	0,374872	3	Ppp1ca
3,12E-05	0,303611	0,707	0,568	0,385687	3	Gimap6
3,15E-05	0,311691	0,324	0,21	0,390346	3	E330020D12Rik
3,19E-05	0,319827	0,766	0,642	0,395417	3	Ifi30
3,19E-05	-0,26492	0,064	0,184	0,395512	3	Nmral1
3,28E-05	-0,30983	0,101	0,229	0,40575	3	Mad2l1
3,39E-05	-0,25948	0,064	0,182	0,419247	3	Gatm
3,55E-05	-0,33799	0,457	0,596	0,439926	3	Hn1
3,9E-05	0,412333	0,638	0,541	0,483047	3	Myo1e
4,01E-05	-0,35846	0,468	0,596	0,496349	3	Tcea1
4,16E-05	-0,25667	0,096	0,223	0,514827	3	Sik3
4,3E-05	-0,32497	0,463	0,585	0,532197	3	Cox7b
4,5E-05	-0,32056	0,128	0,25	0,557468	3	Tk1
4,74E-05	-0,33377	0,441	0,561	0,586982	3	Atp5j
4,74E-05	-0,31688	0,351	0,499	0,587065	3	Strbp
5,47E-05	-0,27431	0,654	0,765	0,677661	3	Btf3
5,9E-05	-0,26303	0,069	0,182	0,730067	3	Stxbp1
5,91E-05	-0,35416	0,186	0,321	0,731861	3	Rgs2
6,1E-05	-0,27905	0,282	0,432	0,755433	3	Rbm38
6,22E-05	-0,31449	0,09	0,209	0,770579	3	Lig1
6,38E-05	-0,27276	0,197	0,336	0,789332	3	Cpsf2
6,63E-05	-0,5183	0,16	0,285	0,82131	3	Top2a
6,78E-05	-0,26037	0,612	0,727	0,839092	3	Srsf7
7,14E-05	-0,2829	0,644	0,753	0,883334	3	Dynll1
7,56E-05	-0,54029	0,149	0,267	0,935415	3	Nrgn
7,82E-05	0,266019	0,489	0,356	0,967607	3	Trim12c
7,96E-05	-0,27265	0,149	0,279	0,985108	3	Psmd13
7,98E-05	-0,29491	0,255	0,398	0,987551	3	Tpi1
8,09E-05	-0,29767	0,271	0,415	1	3	Pxk
8,52E-05	-0,29058	0,686	0,8	1	3	Srsf3
8,53E-05	-0,28688	0,431	0,562	1	3	Psmb4
8,67E-05	-0,25716	0,383	0,536	1	3	Nedd8
9,06E-05	-0,37595	0,101	0,218	1	3	Eif5a2
9,16E-05	-0,28702	0,362	0,502	1	3	Cbx3
9,21E-05	0,294621	0,431	0,304	1	3	Tank

9,32E-05	-0,31573	0,335	0,467	1	3	Cct8
9,36E-05	-0,25953	0,644	0,766	1	3	Hvcn1
9,4E-05	-0,25521	0,367	0,493	1	3	Rftn1
0,000102	-0,26812	0,527	0,639	1	3	Atp5l
0,000105	0,26473	0,314	0,195	1	3	Ccng2
0,000108	-0,31292	0,319	0,446	1	3	Ube2j1
0,000111	-0,33416	0,484	0,592	1	3	Mif4gd
0,000141	-0,27115	0,452	0,573	1	3	Nono
0,000145	-0,37809	0,479	0,573	1	3	Scaf11
0,00015	-0,29429	0,511	0,611	1	3	Hnrnpm
0,000151	0,256416	0,862	0,809	1	3	Ctss
0,000152	-0,26324	0,58	0,715	1	3	Pten
0,000166	-0,26122	0,255	0,387	1	3	Naa50
0,000167	-0,28831	0,447	0,585	1	3	Atp6v1f
0,000167	-0,29029	0,665	0,749	1	3	Ywhae
0,000167	-0,27134	0,681	0,732	1	3	Hnrnpu
0,00017	-0,25448	0,995	0,989	1	3	Cd53
0,00017	-0,28861	0,229	0,361	1	3	Trim35
0,000178	-0,26722	0,218	0,339	1	3	Synrg
0,000179	-0,26091	0,181	0,311	1	3	Txnl1
0,000179	-0,27558	0,665	0,753	1	3	Serf2
0,00019	0,288895	0,707	0,612	1	3	Clk1
0,000206	-0,38896	0,394	0,508	1	3	Mif
0,000207	0,270328	0,527	0,408	1	3	Stk38
0,000231	0,252098	0,42	0,305	1	3	Fbxo11
0,000237	-0,2579	0,165	0,285	1	3	Slc30a5
0,000238	-0,26543	0,266	0,391	1	3	Fam105b
0,000247	-0,31649	0,383	0,506	1	3	Srpk3
0,000263	-0,28041	0,245	0,368	1	3	Smarca4
0,000282	0,275346	0,436	0,313	1	3	Cd2
0,000294	0,328755	0,431	0,321	1	3	Lck
0,0003	0,27825	0,463	0,35	1	3	Atp6v0b
0,000302	-0,31178	0,41	0,53	1	3	Cycs
0,000314	-0,30632	0,053	0,147	1	3	Ccnb2
0,000315	0,250185	0,239	0,144	1	3	Smpdl3a
0,000322	0,252594	0,441	0,324	1	3	Rabac1
0,000322	-0,2828	0,362	0,481	1	3	Cox5a
0,000334	-0,26842	0,245	0,373	1	3	Tmf1
0,000344	-0,27066	0,027	0,108	1	3	Ccna2
0,00035	-0,25341	0,415	0,56	1	3	Cr2
0,000362	-0,25408	0,16	0,275	1	3	Larp7
0,000372	0,260386	0,543	0,426	1	3	Gigyf1
0,000374	0,258307	0,271	0,174	1	3	Card6
0,000394	-0,27336	0,245	0,364	1	3	Polr2g
0,000395	-0,26696	0,144	0,257	1	3	Kpna2
0,000399	0,272567	0,34	0,242	1	3	Cyth4
0,000403	-0,37596	0,106	0,21	1	3	Rrm2
0,000412	0,288943	0,702	0,614	1	3	Snx2
0,000465	-0,32957	0,059	0,151	1	3	Egr1
0,000484	0,295136	0,729	0,629	1	3	Lbh

0,000493	-0,29601	0,495	0,599	1	3	Ube2l3
0,000511	-0,26791	0,34	0,465	1	3	Srp14
0,000532	0,276863	0,463	0,358	1	3	Itgal
0,000543	0,288292	0,399	0,295	1	3	Trim12a
0,000591	-0,29376	0,505	0,591	1	3	Mdh1
0,000649	-0,30806	0,266	0,377	1	3	Rraga
0,000671	0,26232	0,628	0,532	1	3	Sec11c
0,000805	-0,25847	0,468	0,591	1	3	Ppp1r16b
0,000833	-0,28288	0,41	0,525	1	3	Lsm14a
0,000862	0,357632	0,356	0,263	1	3	1110059E24Rik
0,000865	0,253654	0,436	0,337	1	3	Mtdh
0,000869	-0,26894	0,122	0,224	1	3	Siah2
0,000941	-0,27612	0,351	0,461	1	3	Nhp2l1
0,000946	-0,25426	0,436	0,539	1	3	Ewsr1
0,000967	-0,29389	0,415	0,508	1	3	Hprt
0,001072	0,269841	0,606	0,51	1	3	Huwe1
0,001091	0,250241	0,824	0,727	1	3	Stk17b
0,001099	-0,28353	0,495	0,598	1	3	Dnaja1
0,001175	0,283867	0,372	0,273	1	3	C130026I21Rik
0,001192	-0,2607	0,33	0,454	1	3	Cmpk1
0,001245	-0,25687	0,42	0,529	1	3	Hcfc1
0,001428	-0,25125	0,617	0,703	1	3	Cpne8
0,001455	-0,25421	0,176	0,273	1	3	Icam1
0,001455	-0,26167	0,473	0,581	1	3	H2-Eb2
0,001597	-0,27263	0,564	0,653	1	3	Ikzf1
0,001696	0,32429	0,324	0,229	1	3	Zscan26
0,001899	-0,26828	0,644	0,709	1	3	Tnfaip8
0,002071	0,265085	0,351	0,255	1	3	Nucb1
0,002374	0,264968	0,91	0,863	1	3	Cd24a
0,002392	-0,25996	0,628	0,689	1	3	Atp5c1
0,002553	0,298073	0,436	0,344	1	3	Itgb1
0,002655	-0,29105	0,42	0,501	1	3	Tmpo
0,002953	0,263275	0,457	0,372	1	3	Btg2
0,003179	-0,26656	0,282	0,377	1	3	Tmem64
0,003903	0,255019	0,606	0,547	1	3	Ikzf3
0,004066	0,25345	0,372	0,291	1	3	Vamp2
0,00455	0,261866	0,612	0,519	1	3	Cxcr5
0,004762	-0,29629	0,42	0,512	1	3	Cd83
0,004771	-0,25365	0,516	0,6	1	3	Zfp207
0,006692	0,250624	0,734	0,645	1	3	Ezr
0,008021	-0,29874	0,755	0,796	1	3	Tubb5
2,52E-68	1,886342	0,786	0,169	3,12E-64	4	Irgm1
6,14E-67	1,377802	0,631	0,09	7,6E-63	4	Gbp7
1,48E-54	1,738545	0,903	0,325	1,83E-50	4	Gm1966
1,36E-53	0,510086	0,272	0,015	1,68E-49	4	Serpina3f
2,4E-51	1,90237	0,971	0,495	2,97E-47	4	Stat1
3,48E-48	0,652151	0,252	0,015	4,3E-44	4	Ifit3
6,57E-47	1,133149	0,553	0,101	8,14E-43	4	Igtp
3,6E-46	1,387479	0,553	0,103	4,46E-42	4	Serpina3g
1,17E-42	1,034648	0,485	0,08	1,45E-38	4	Irgm2

1,84E-39	0,854115	0,32	0,036	2,28E-35	4	Gbp6
1,34E-38	0,505432	0,146	0,004	1,66E-34	4	Iigp1
2,12E-38	1,206865	0,631	0,16	2,62E-34	4	Pydc3
5,1E-37	0,36336	0,136	0,004	6,31E-33	4	I830012O16Rik
1,58E-36	0,383734	0,243	0,02	1,96E-32	4	Gm12250
6,43E-36	1,313116	0,961	0,473	7,96E-32	4	Mndal
4,64E-35	1,202867	0,699	0,222	5,75E-31	4	Irf1
2,07E-34	1,360224	0,913	0,453	2,56E-30	4	Ifi203
6,46E-34	1,262413	0,427	0,079	7,99E-30	4	Gbp2
1,68E-33	1,690414	0,835	0,423	2,09E-29	4	Ifi47
3,19E-33	1,541199	0,66	0,226	3,95E-29	4	Gbp4
1,76E-32	1,30051	0,971	0,717	2,18E-28	4	Ly6a
3,87E-30	1,170517	0,893	0,443	4,79E-26	4	Pml
6,8E-30	0,814737	1	0,995	8,42E-26	4	B2m
5,72E-27	0,738547	0,437	0,098	7,08E-23	4	Mpeg1
7,53E-27	0,377783	0,175	0,014	9,33E-23	4	Rtp4
7,29E-26	0,97756	0,99	0,82	9,03E-22	4	Shisa5
8,77E-26	1,086746	0,99	0,85	1,09E-21	4	Samhd1
4,86E-23	0,970333	0,932	0,506	6,02E-19	4	Ly6d
7,13E-23	0,274574	0,126	0,008	8,83E-19	4	Csf1
7,19E-23	0,575042	0,165	0,016	8,91E-19	4	Ifit2
4,19E-22	0,748341	0,476	0,137	5,19E-18	4	Ddx58
1,08E-21	0,737493	1	0,989	1,33E-17	4	H2-D1
2,71E-21	-1,7359	0,272	0,687	3,35E-17	4	Igj
1,22E-20	0,785621	0,359	0,087	1,52E-16	4	Cd274
1,34E-20	0,944234	0,631	0,26	1,66E-16	4	Parp14
5,63E-19	0,967133	0,796	0,398	6,97E-15	4	Sell
6,57E-19	0,816551	0,961	0,784	8,13E-15	4	Mycbp2
1,07E-18	-1,42997	0,398	0,721	1,32E-14	4	Basp1
1,27E-18	0,771516	0,359	0,095	1,57E-14	4	Zbp1
1,31E-18	0,706685	1	0,98	1,62E-14	4	H2-K1
8,13E-18	0,701762	0,476	0,164	1,01E-13	4	Nlrc5
1,33E-17	0,939448	0,893	0,562	1,65E-13	4	Cmah
1,8E-17	0,604645	0,33	0,083	2,23E-13	4	Irf7
3,93E-17	-1,2058	0,078	0,514	4,87E-13	4	Mef2b
5,34E-17	0,90102	0,33	0,089	6,61E-13	4	Slfn5
5,68E-17	-0,87846	1	0,999	7,03E-13	4	Cfl1
1,06E-16	0,819056	0,913	0,664	1,31E-12	4	Gimap4
1,42E-16	-1,29771	0,049	0,471	1,76E-12	4	Rgs13
1,45E-16	0,653271	0,33	0,086	1,79E-12	4	Ffar2
7,3E-16	0,31995	0,184	0,03	9,04E-12	4	BC094916
3,74E-15	0,654646	0,408	0,139	4,64E-11	4	Slfn8
4,61E-15	0,744039	0,816	0,513	5,71E-11	4	Tap1
5,28E-15	0,394558	0,107	0,01	6,54E-11	4	Ifit1
7,39E-15	0,482919	1	0,999	9,15E-11	4	Ly6e
5,35E-14	-1,11909	0,485	0,752	6,63E-10	4	Hmgn2
7,22E-14	0,547891	0,456	0,166	8,94E-10	4	Evi2a
7,92E-14	1,020878	0,757	0,505	9,8E-10	4	Pyhin1
9,33E-14	-0,94816	0,068	0,448	1,16E-09	4	S1pr2
1,87E-13	0,621713	0,534	0,233	2,32E-09	4	Cdkn1b

2,2E-13	0,310393	0,243	0,058	2,73E-09	4	St3gal6
3,47E-13	0,445705	0,165	0,029	4,29E-09	4	Oasl2
4,14E-13	0,645684	0,757	0,389	5,13E-09	4	Klf2
5,35E-13	-0,75038	0,961	0,984	6,63E-09	4	Ucp2
5,38E-13	0,557732	0,32	0,1	6,66E-09	4	Gm14446
5,59E-13	0,736311	0,485	0,197	6,91E-09	4	Lgals9
9,81E-13	0,620413	1	0,97	1,21E-08	4	Malat1
1,05E-12	0,639832	0,553	0,26	1,3E-08	4	Irf9
1,09E-12	0,551805	0,748	0,405	1,35E-08	4	Sub1
1,8E-12	0,515599	0,563	0,248	2,23E-08	4	Itgb7
5,82E-12	-0,95652	0,117	0,452	7,21E-08	4	8430410A17Rik
8,57E-12	0,763527	0,534	0,254	1,06E-07	4	Serpinb1a
1,44E-11	0,26171	0,175	0,036	1,78E-07	4	H2-T24
2,61E-11	0,381053	0,272	0,082	3,23E-07	4	Xaf1
2,78E-11	0,588159	0,777	0,499	3,45E-07	4	Itm2b
2,93E-11	0,503486	0,728	0,391	3,63E-07	4	Capg
3,01E-11	-0,76072	0,718	0,876	3,73E-07	4	Cd24a
3,35E-11	0,754828	0,748	0,544	4,15E-07	4	Samd9l
5,77E-11	0,661947	0,796	0,569	7,15E-07	4	Gimap6
5,95E-11	0,414765	0,291	0,093	7,37E-07	4	Gbp9
6,97E-11	0,34199	0,214	0,055	8,63E-07	4	Tlr7
8,16E-11	0,618527	0,495	0,234	1,01E-06	4	Ifi27l2a
1,13E-10	0,62759	0,874	0,642	1,4E-06	4	H2-T23
1,22E-10	0,556639	0,641	0,355	1,51E-06	4	Cyb561a3
1,37E-10	0,467805	0,505	0,23	1,7E-06	4	Notch2
1,73E-10	0,456398	0,456	0,202	2,14E-06	4	Stat2
2,14E-10	0,553265	0,689	0,376	2,65E-06	4	Snn
2,17E-10	0,532683	0,612	0,318	2,69E-06	4	S100a10
2,32E-10	0,68212	0,34	0,137	2,87E-06	4	Wars
2,75E-10	0,295489	0,214	0,057	3,4E-06	4	Bst2
3,45E-10	-0,70123	0,592	0,8	4,27E-06	4	Slc25a5
4,11E-10	0,502384	0,641	0,328	5,09E-06	4	Map3k1
4,38E-10	0,61905	0,748	0,522	5,42E-06	4	Gimap3
5,31E-10	0,281793	0,136	0,026	6,58E-06	4	Lgals3bp
5,86E-10	0,556497	0,932	0,852	7,25E-06	4	D4Wsu53e
6,24E-10	0,59758	0,67	0,394	7,73E-06	4	Anxa6
6,75E-10	-0,84382	0,146	0,442	8,35E-06	4	Gcsam
7,3E-10	0,712176	0,408	0,175	9,03E-06	4	Ms4a4c
8,05E-10	0,52326	0,65	0,374	9,97E-06	4	Ctsc
1,33E-09	0,280831	0,175	0,043	1,64E-05	4	Fcgrt
1,38E-09	0,390533	0,388	0,166	1,71E-05	4	6030440G07Rik
1,56E-09	0,551689	0,718	0,442	1,93E-05	4	Evi2b
1,73E-09	0,508972	0,621	0,377	2,14E-05	4	2310034O05Rik
1,81E-09	-0,563	0,068	0,361	2,23E-05	4	Dap
2,15E-09	-0,66721	0,631	0,804	2,66E-05	4	Pou2af1
2,31E-09	-0,49429	0,087	0,39	2,86E-05	4	Dbi
2,44E-09	0,747876	0,602	0,383	3,01E-05	4	Trim34a
2,7E-09	-0,55672	0,01	0,277	3,34E-05	4	Nuggc
2,81E-09	0,382154	0,35	0,136	3,47E-05	4	Cnn3
2,87E-09	-0,72235	0,408	0,647	3,55E-05	4	Hmgn1

3,36E-09	0,626161	0,398	0,175	4,16E-05	4	Plac8
3,42E-09	-0,77297	0,252	0,543	4,23E-05	4	Mtf2
3,55E-09	-0,58822	0,408	0,708	4,4E-05	4	Rhoh
3,63E-09	0,423039	0,485	0,228	4,49E-05	4	Cd55
3,77E-09	0,643862	0,583	0,31	4,67E-05	4	Bcl2
4,15E-09	-0,5947	0,126	0,419	5,14E-05	4	Helq
4,41E-09	-0,49833	0,981	0,998	5,46E-05	4	Actg1
5,36E-09	-0,81816	0,32	0,592	6,64E-05	4	Klhl6
5,8E-09	0,583675	0,524	0,284	7,18E-05	4	Gimap7
5,99E-09	-0,62124	0,728	0,852	7,41E-05	4	Sypl
6,2E-09	0,481134	0,971	0,815	7,68E-05	4	Macf1
6,49E-09	-0,68547	0,272	0,542	8,03E-05	4	Marcksl1
9,02E-09	0,281049	0,99	0,969	0,000112	4	Gm17821
9,2E-09	0,591704	0,65	0,385	0,000114	4	B3gnt5
9,37E-09	0,417271	0,485	0,235	0,000116	4	Itpr1
9,53E-09	-0,79724	0,204	0,458	0,000118	4	Bcl6
9,63E-09	-0,56934	0,165	0,449	0,000119	4	Glrx3
1,03E-08	0,610209	0,874	0,811	0,000128	4	Ctss
1,21E-08	-1,05683	0,505	0,691	0,00015	4	H2afz
1,25E-08	0,445395	0,32	0,13	0,000154	4	Parp9
1,56E-08	0,366456	0,35	0,144	0,000193	4	Kbtbd11
1,91E-08	-0,97694	0,136	0,396	0,000236	4	Stmn1
2,08E-08	0,593267	0,718	0,491	0,000258	4	Gimap8
2,29E-08	0,50264	0,466	0,242	0,000283	4	Gm8369
2,7E-08	-0,66893	0,437	0,654	0,000335	4	Erp44
3,05E-08	-0,57157	0,155	0,422	0,000378	4	Dcaf12
3,29E-08	0,60593	0,718	0,471	0,000407	4	Fam65b
3,61E-08	0,599062	0,311	0,13	0,000446	4	Gbp8
3,69E-08	-1,02287	0,214	0,475	0,000457	4	Hmgb2
3,95E-08	-0,54168	0,058	0,311	0,000489	4	Rassf6
4,61E-08	0,353915	0,417	0,191	0,00057	4	Ccr6
5,62E-08	-0,64077	0,379	0,643	0,000695	4	Ran
6,01E-08	0,468018	0,66	0,412	0,000744	4	Ncf1
7,36E-08	0,250383	0,194	0,059	0,000911	4	Ppa1
7,43E-08	-0,54077	0,641	0,801	0,00092	4	Atp5a1
7,88E-08	0,514379	0,825	0,644	0,000976	4	Ifi30
9,37E-08	-0,79349	0,291	0,515	0,00116	4	Aicda
1,09E-07	0,430669	0,32	0,133	0,001354	4	Trim30b
1,12E-07	-0,50915	0,602	0,792	0,001382	4	Atp5g2
1,33E-07	-0,52112	0,078	0,317	0,001644	4	Dstn
1,41E-07	0,489087	0,437	0,222	0,001749	4	Zfp318
1,49E-07	-0,66027	0,505	0,706	0,001842	4	Txn1
1,51E-07	-0,47268	0,951	0,967	0,001875	4	Calm1
1,62E-07	0,540734	0,777	0,561	0,002003	4	Bank1
1,7E-07	0,640085	0,524	0,307	0,002106	4	Trim30a
1,75E-07	-0,31004	1	1	0,002164	4	Actb
1,99E-07	0,482915	0,68	0,462	0,002469	4	Stat3
2,27E-07	-0,68025	0,155	0,4	0,002816	4	Lpp
2,34E-07	0,548468	0,883	0,796	0,002901	4	Ly86
2,52E-07	-0,54557	0,573	0,75	0,003116	4	Ywhae

3,25E-07	-0,61325	0,437	0,637	0,00403	4	Ldha
3,46E-07	-0,50833	0,078	0,321	0,004289	4	Rgs2
3,62E-07	-0,43596	0,99	0,992	0,004478	4	Pabpc1
4,05E-07	0,602304	0,806	0,614	0,005014	4	Helz2
4,32E-07	0,437513	0,262	0,104	0,005343	4	Gbp3
4,36E-07	0,543562	0,388	0,208	0,005394	4	Nampt
4,93E-07	-0,86361	0,388	0,625	0,006106	4	Pcna
4,98E-07	-0,56534	0,544	0,697	0,006167	4	Arpc5l
5,39E-07	-0,43676	0,825	0,925	0,006673	4	Rbm3
5,39E-07	-0,83533	0,097	0,327	0,006677	4	Mki67
5,6E-07	-0,47663	0,398	0,639	0,006935	4	Cox6b1
5,67E-07	0,313302	0,398	0,189	0,007014	4	Scml4
6,07E-07	-0,53664	0,097	0,328	0,007516	4	Eaf2
6,19E-07	-0,70811	0,35	0,569	0,007657	4	Dusp2
6,68E-07	0,399523	0,146	0,041	0,008272	4	Cmpk2
6,91E-07	-0,44347	0,291	0,534	0,00856	4	Nedd8
7,19E-07	-0,55332	0,194	0,436	0,008907	4	Mbd4
7,48E-07	-0,44938	0,903	0,946	0,009264	4	Nap1l1
7,49E-07	0,409354	0,718	0,511	0,009273	4	Psmb9
7,58E-07	-0,44746	0,738	0,861	0,009382	4	Arpc4
9,09E-07	-0,39195	0,01	0,208	0,011258	4	Mybl1
9,4E-07	0,442925	0,757	0,606	0,011635	4	Gimap1
9,63E-07	-0,36663	1	1	0,011926	4	mtNd3
9,73E-07	-0,54643	0,66	0,779	0,01205	4	Gapdh
1,05E-06	-0,38214	0,971	0,987	0,012972	4	Fth1
1,24E-06	-0,45234	0,631	0,798	0,015393	4	Srsf3
1,39E-06	0,494759	0,505	0,314	0,017255	4	Rasgrp2
1,4E-06	0,310972	0,981	0,94	0,017279	4	A630089N07Rik
1,51E-06	0,46041	0,35	0,173	0,018671	4	Slfn2
1,57E-06	-0,4622	0,117	0,339	0,019478	4	Synrg
1,57E-06	-0,44142	0,087	0,307	0,019494	4	Bzw2
1,82E-06	0,474746	0,67	0,472	0,022591	4	Dock10
1,87E-06	-0,39862	0,029	0,227	0,023191	4	Rgs10
1,9E-06	-0,38271	0,981	0,983	0,023464	4	Arpc2
1,98E-06	-0,41401	0,922	0,959	0,024484	4	Myl6
2,1E-06	0,371592	0,466	0,257	0,025947	4	Fgd2
2,19E-06	-0,43839	0,553	0,755	0,027151	4	Serf2
2,23E-06	-0,50211	0,621	0,792	0,027639	4	Anp32b
2,24E-06	0,406144	0,417	0,223	0,027743	4	Eif2ak2
2,3E-06	0,369606	0,718	0,514	0,028454	4	Dnajc7
2,64E-06	-0,42488	0,126	0,355	0,032668	4	Sema7a
2,77E-06	-0,47813	0,136	0,354	0,03435	4	Ell3
2,9E-06	0,278163	0,233	0,092	0,035939	4	Fam46a
3,14E-06	0,501435	0,903	0,747	0,03882	4	Il2rg
3,15E-06	0,314737	0,505	0,287	0,039002	4	Acad9
3,2E-06	-0,41163	0,136	0,364	0,039609	4	Polr2g
3,83E-06	-0,45568	0,573	0,722	0,047378	4	Ube2d2a
4E-06	0,353213	0,942	0,805	0,049565	4	Btg1
4,14E-06	-0,52853	0,35	0,575	0,051307	4	Foxo1
4,15E-06	0,385726	0,786	0,609	0,05135	4	Tbc1d10c

4,18E-06	-0,43159	0,049	0,248	0,05179	4	Mcm3
4,39E-06	0,35517	0,398	0,206	0,054388	4	Arhgef18
4,65E-06	-0,58574	0,476	0,654	0,057598	4	Top1
4,73E-06	-0,42108	0,427	0,641	0,058603	4	Pold4
4,77E-06	0,498288	0,641	0,445	0,059014	4	Filip1l
4,86E-06	0,47096	0,689	0,534	0,060193	4	Psme2
4,95E-06	-0,49824	0,233	0,465	0,061242	4	Dck
5,37E-06	-0,4079	0,311	0,524	0,066441	4	Shfm1
5,43E-06	-0,44828	0,369	0,594	0,067227	4	Mdh1
5,66E-06	-0,4539	0,087	0,292	0,070039	4	Lipc
5,7E-06	0,323044	0,33	0,164	0,070531	4	Casp4
5,82E-06	-0,4678	0,379	0,609	0,072047	4	Lrmp
5,84E-06	-0,56972	0,398	0,607	0,072256	4	Bach2
5,99E-06	-0,42186	0,621	0,792	0,074213	4	Cox8a
6,17E-06	0,334442	0,398	0,206	0,076342	4	S1pr1
6,41E-06	0,330296	0,427	0,231	0,079406	4	Gpr183
6,43E-06	-0,28689	0	0,167	0,079559	4	Ada
8,06E-06	0,35406	0,223	0,094	0,099803	4	Mllt3
8,13E-06	-0,4853	0,262	0,467	0,100638	4	Rfc1
8,35E-06	0,406297	0,796	0,593	0,10339	4	Lmo2
8,59E-06	-0,34975	0,01	0,179	0,106393	4	Gatm
8,96E-06	-0,52152	0,379	0,574	0,11093	4	Scaf11
9,03E-06	-0,67131	0,204	0,405	0,111766	4	Mcm6
9,03E-06	0,537501	0,291	0,144	0,111828	4	Socs1
9,49E-06	-0,48975	0,184	0,387	0,117447	4	2700029M09Rik
9,9E-06	-0,40319	0,408	0,588	0,122593	4	Oaz1
1,06E-05	-0,40524	0,078	0,269	0,13077	4	Emid1
1,06E-05	0,513524	0,485	0,318	0,130932	4	Adar
1,14E-05	0,434347	0,67	0,476	0,1412	4	Txnip
1,21E-05	-0,36185	0,291	0,511	0,149747	4	Hprt
1,31E-05	-0,35153	0,029	0,202	0,161734	4	Endou
1,32E-05	-0,33424	1	0,997	0,162895	4	Rplp0
1,33E-05	0,263281	0,155	0,053	0,164296	4	Gm6548
1,36E-05	-0,66779	0,097	0,282	0,167771	4	Top2a
1,39E-05	0,32051	0,427	0,238	0,171768	4	Hhex
1,44E-05	-0,41245	0,913	0,962	0,178328	4	Npm1
1,52E-05	-0,3089	1	0,991	0,188106	4	H3f3a
1,61E-05	0,430783	0,67	0,5	0,198964	4	Cd47
1,67E-05	0,256599	0,126	0,039	0,206423	4	Cdh17
1,7E-05	-0,39333	0,068	0,254	0,210281	4	Dut
1,71E-05	-0,41152	0,961	0,982	0,211606	4	Laptm5
1,76E-05	-0,42526	0,583	0,735	0,217786	4	Atp5g3
1,79E-05	-0,46396	0,214	0,417	0,221834	4	Nme1
1,88E-05	-0,35596	0,175	0,398	0,232439	4	Calm3
1,9E-05	-0,39885	0,34	0,556	0,235292	4	Tomm20
1,92E-05	-0,34975	0,01	0,168	0,23717	4	Uhrf1
1,92E-05	-0,4352	0,136	0,328	0,237433	4	Naa40
1,99E-05	-0,3652	0,019	0,182	0,246913	4	H1fx
2,01E-05	-0,33533	0,097	0,293	0,248967	4	Mtmr14
2,05E-05	0,333017	0,524	0,34	0,25401	4	Uba7

2,06E-05	-0,39272	0,194	0,394	0,255485	4	Ubxn1
2,13E-05	-0,42832	0,66	0,791	0,264269	4	Mzb1
2,13E-05	-0,40099	0,447	0,627	0,2643	4	Cct2
2,15E-05	0,374102	0,913	0,856	0,265745	4	Mcl1
2,22E-05	-0,27028	0,029	0,202	0,27522	4	Cdca7
2,75E-05	0,340539	0,874	0,741	0,339908	4	Hmha1
2,75E-05	0,407856	0,456	0,274	0,34064	4	Esyt1
2,76E-05	0,275169	0,311	0,153	0,341378	4	2310001H17Rik
3,14E-05	-0,44666	0,466	0,679	0,389167	4	Smim14
3,35E-05	-0,38473	0,534	0,712	0,415123	4	Tnfaip8
3,36E-05	-0,28376	0,029	0,19	0,41584	4	Neil1
3,55E-05	0,384333	0,845	0,73	0,439382	4	Stk17b
3,55E-05	0,388086	0,583	0,381	0,439739	4	Dgka
3,59E-05	0,278607	0,35	0,192	0,444132	4	Lime1
3,6E-05	-0,60294	0,087	0,265	0,445918	4	Nrgn
3,63E-05	-0,28003	0,136	0,341	0,44975	4	Lpin2
4,19E-05	-0,68999	0,068	0,236	0,518133	4	2810417H13Rik
4,23E-05	0,3266	0,233	0,106	0,523689	4	Got1
4,52E-05	-0,34709	0,961	0,99	0,560112	4	Coro1a
4,73E-05	0,342647	0,786	0,63	0,585692	4	Lbh
5,09E-05	-0,44393	0,204	0,398	0,630613	4	Anxa2
5,48E-05	-0,35864	0,534	0,703	0,678988	4	Hnrnpk
5,57E-05	-0,44213	0,282	0,459	0,689651	4	Hipk3
5,59E-05	-0,39658	0,874	0,916	0,692618	4	Ppia
5,6E-05	0,397396	0,903	0,738	0,693715	4	Ltb
5,62E-05	-0,50881	0,408	0,594	0,695324	4	Mbd2
5,78E-05	-0,38322	0,078	0,249	0,715316	4	Mcm2
6,16E-05	-0,3159	0,718	0,852	0,763049	4	Limd2
6,31E-05	-0,37697	0,757	0,869	0,781682	4	Tnfrsf13c
6,47E-05	0,408015	0,563	0,379	0,800637	4	Stk10
6,51E-05	-0,3728	0,049	0,207	0,806078	4	Prim1
6,57E-05	-0,4088	0,602	0,75	0,813426	4	Dynll1
6,66E-05	-0,37368	0,01	0,151	0,825048	4	Cenpe
6,8E-05	-0,49295	0,427	0,579	0,841683	4	Tmem131
6,82E-05	0,28142	0,311	0,161	0,844539	4	Cd97
6,95E-05	-0,37194	0,078	0,251	0,860468	4	Cdc14b
6,95E-05	-0,35971	0,078	0,248	0,860528	4	Sh2b2
7,1E-05	-0,39613	0,951	0,958	0,878591	4	Pfn1
7,11E-05	0,325702	0,835	0,672	0,879731	4	Scd1
7,11E-05	-0,40284	0,126	0,296	0,880709	4	Xrcc1
7,28E-05	-0,30547	0,019	0,165	0,90099	4	Ccdc17
7,38E-05	-0,43176	0,417	0,58	0,913078	4	Atp6v1f
7,39E-05	0,401009	0,262	0,133	0,915379	4	Trim30d
7,44E-05	0,312709	0,515	0,318	0,920542	4	Ier5
7,44E-05	-0,25774	0,039	0,198	0,920873	4	Pla2g12a
7,54E-05	-0,39753	0,204	0,394	0,933508	4	Tpi1
7,66E-05	-0,37739	0,68	0,822	0,947759	4	Hnrnpa3
7,75E-05	-0,38684	0,379	0,55	0,960022	4	Ppp4r2
8,13E-05	0,30504	0,67	0,484	1	4	Itga4
8,13E-05	-0,36644	0,524	0,722	1	4	Arhgdia

9,04E-05	-0,43802	0,223	0,399	1	4	Pnp
9,13E-05	0,301905	0,68	0,523	1	4	Wdfy4
9,13E-05	-0,29604	0,184	0,387	1	4	Taf9
9,17E-05	0,260017	0,175	0,071	1	4	Mfhas1
9,29E-05	-0,37463	0,165	0,346	1	4	Bid
9,34E-05	-0,3231	0,078	0,247	1	4	Cecr2
9,39E-05	-0,3468	0,243	0,456	1	4	Akt1
9,6E-05	-0,25216	0,049	0,211	1	4	Cnst
9,61E-05	-0,28132	0,029	0,178	1	4	Rnaseh2b
9,78E-05	-0,26642	0,049	0,206	1	4	Gng12
9,95E-05	-0,36394	0,058	0,216	1	4	Cyp51
0,0001	-0,28261	0,049	0,205	1	4	Amz2
0,0001	-0,2738	0,087	0,255	1	4	Mprip
0,000101	0,262555	0,282	0,142	1	4	Ckap4
0,000101	-0,38817	0,214	0,4	1	4	Rev3l
0,000102	0,420192	0,825	0,725	1	4	Tmbim6
0,000104	0,253326	0,437	0,264	1	4	Lypla1
0,000105	-0,34043	0,408	0,601	1	4	Usp7
0,000106	-0,38726	0,01	0,145	1	4	Ccnb2
0,000107	-0,43196	0,553	0,725	1	4	Hnrnpab
0,000111	-0,27105	0,01	0,144	1	4	Dtl
0,000112	-0,34919	0,049	0,206	1	4	Lig1
0,000113	-0,36103	0,184	0,366	1	4	Sh3bgrl
0,000122	-0,27837	1	0,999	1	4	Rpl41
0,000127	-0,41431	0,068	0,228	1	4	Cks2
0,000129	-0,42207	0,447	0,591	1	4	Tcea1
0,00013	-0,35856	0,272	0,458	1	4	Csnk2b
0,000132	-0,28188	0,117	0,296	1	4	Cnih
0,000132	0,285232	0,262	0,133	1	4	Tsc22d3
0,000136	-0,40369	0,087	0,244	1	4	Mcm4
0,000137	-0,30472	0,107	0,275	1	4	Ruvbl2
0,000137	0,280325	0,398	0,243	1	4	2900060B14Rik
0,000138	-0,25022	0,019	0,157	1	4	Gmnn
0,000143	0,279621	0,883	0,789	1	4	Faim3
0,000145	-0,29849	0,243	0,431	1	4	Lsm4
0,00015	0,340017	0,583	0,399	1	4	Tnrc6b
0,000156	-0,26241	0,155	0,345	1	4	Qars
0,000157	-0,28096	0,272	0,46	1	4	Nhp2l1
0,000158	-0,36535	0,214	0,384	1	4	Ktn1
0,000167	0,312348	0,262	0,133	1	4	Arap2
0,000167	-0,44353	0,136	0,306	1	4	Lacc1
0,000179	-0,37634	0,408	0,582	1	4	Polr1d
0,00018	0,335576	0,515	0,346	1	4	Myl12b
0,00018	-0,43577	0,816	0,862	1	4	Cnn2
0,000181	0,270468	0,505	0,325	1	4	Rabac1
0,000183	-0,28135	0,146	0,319	1	4	Ndufa11
0,000183	-0,52037	0,32	0,486	1	4	Hpse
0,000183	0,28304	0,612	0,444	1	4	Psap
0,000194	0,340165	0,631	0,522	1	4	Cxcr5
0,000194	0,409168	0,728	0,617	1	4	Snx2

0,000197	-0,33533	0,524	0,692	1	4	Atp5c1
0,0002	-0,34486	0,32	0,499	1	4	Arpc3
0,000201	-0,37533	0,398	0,571	1	4	Psmb7
0,00021	0,360494	0,447	0,304	1	4	Birc3
0,000212	-0,34724	0,282	0,464	1	4	Cct8
0,000222	-0,26571	0,136	0,313	1	4	Thoc7
0,000224	-0,28628	0,99	0,993	1	4	Rpl28
0,000234	-0,41335	0,35	0,523	1	4	Lsm14a
0,000235	-0,28385	0,058	0,208	1	4	Rpf1
0,000239	-0,32444	0,175	0,345	1	4	Ndufv2
0,000241	0,30604	0,301	0,169	1	4	Nt5c3
0,00026	-0,29177	0,087	0,25	1	4	Fut8
0,00026	-0,31018	0,233	0,411	1	4	Ap2s1
0,000264	-0,36999	0,136	0,294	1	4	Tgfbr1
0,000268	-0,34169	0,107	0,263	1	4	Hat1
0,000269	-0,35941	0,165	0,325	1	4	Psmd1
0,000278	-0,31059	0,184	0,365	1	4	Snrpd1
0,000279	-0,39351	0,107	0,263	1	4	Asf1b
0,000279	0,269946	0,99	0,998	1	4	Ddx5
0,000283	-0,32047	0,117	0,279	1	4	Havcr1
0,000291	0,25088	0,359	0,21	1	4	Vps13b
0,000292	-0,27704	0,097	0,254	1	4	Mrps18c
0,000294	-0,30878	0,874	0,935	1	4	H3f3b
0,000302	-0,32301	0,359	0,547	1	4	Pik3c2b
0,000304	-0,37472	0,262	0,422	1	4	D930015E06Rik
0,000307	0,25776	0,33	0,188	1	4	P2ry10
0,000322	-0,37314	0,243	0,413	1	4	Odc1
0,000332	-0,35729	0,35	0,518	1	4	Erh
0,000342	0,253926	0,214	0,104	1	4	Runx3
0,000352	-0,28607	0,233	0,409	1	4	Psme3
0,000372	-0,27663	0,602	0,705	1	4	Sumo2
0,000385	-0,28294	0,233	0,42	1	4	Slc25a19
0,000396	-0,44595	0,524	0,644	1	4	Crip1
0,000398	-0,45699	0,068	0,208	1	4	Rrm2
0,0004	-0,35575	0,301	0,464	1	4	Coro1b
0,000409	0,304067	0,155	0,067	1	4	Vps37b
0,000411	-0,36845	0,223	0,389	1	4	Slbp
0,000417	-0,31124	0,66	0,785	1	4	Tma7
0,000421	-0,27558	0,097	0,252	1	4	Nasp
0,000426	-0,27159	0,049	0,182	1	4	Tipin
0,000442	-0,29399	0,126	0,29	1	4	Acadl
0,000443	-0,30019	0,087	0,23	1	4	Rnf41
0,000446	-0,35826	0,126	0,284	1	4	Vpreb3
0,000447	-0,27677	0,01	0,124	1	4	Cdk1
0,000448	0,291851	0,398	0,251	1	4	Smarca2
0,000452	-0,29392	0,184	0,346	1	4	D8Ertd738e
0,000454	0,303169	0,553	0,382	1	4	Zfp36
0,000454	-0,35953	0,466	0,606	1	4	Hnrnpa0
0,000458	-0,42029	0,709	0,802	1	4	Hmgb1
0,00046	-0,27656	0,184	0,35	1	4	Brwd1

0,000465	-0,34063	0,204	0,38	1	4	Asap1
0,000488	0,349049	0,184	0,087	1	4	Fcrl5
0,000496	-0,27763	0,039	0,166	1	4	Efnb1
0,000521	0,290899	0,388	0,244	1	4	Pecam1
0,000523	-0,25276	0,049	0,181	1	4	Cep164
0,000526	-0,29893	0	0,105	1	4	Ccna2
0,000534	-0,36915	0,505	0,666	1	4	Banf1
0,000537	-0,32773	0,534	0,684	1	4	Purb
0,00054	-0,26716	0,117	0,268	1	4	Nudt21
0,000543	-0,31553	0,107	0,251	1	4	Ccnd3
0,000552	-0,25405	0,019	0,137	1	4	H1f0
0,000559	-0,29014	0,087	0,234	1	4	Ildr1
0,000561	0,307794	0,67	0,51	1	4	Nfkbia
0,000563	-0,27233	0,097	0,242	1	4	Brd7
0,000565	-0,26101	0,136	0,301	1	4	Eif2a
0,000568	-0,2879	0,107	0,252	1	4	Ndufb8
0,000572	0,387612	0,786	0,601	1	4	Mll5
0,000602	0,255345	0,563	0,401	1	4	Add3
0,000604	-0,26745	0,194	0,368	1	4	Arl5a
0,000616	-0,29273	0,233	0,395	1	4	eGFP
0,000622	-0,31753	0,146	0,299	1	4	Eif2ak3
0,00063	-0,36619	0,282	0,446	1	4	Cdk2ap2
0,000632	-0,34556	0,282	0,457	1	4	Cerk
0,000636	-0,32526	0,155	0,306	1	4	Prkd3
0,000639	-0,27281	0,049	0,176	1	4	Cpne5
0,000648	-0,25357	0,078	0,221	1	4	Rrm1
0,00065	-0,27785	0,049	0,18	1	4	Nmral1
0,00065	0,269427	0,515	0,352	1	4	Lrrc2
0,000651	0,409364	0,204	0,105	1	4	Herc6
0,000658	-0,25341	0,019	0,134	1	4	Tcf19
0,000669	0,258307	0,359	0,219	1	4	Ppcs
0,00067	-0,26558	0,078	0,215	1	4	Abr
0,000674	-0,38229	0,359	0,508	1	4	Id3
0,000676	0,428988	0,689	0,567	1	4	Tapbp
0,000683	-0,40981	0,214	0,364	1	4	Pafah1b3
0,000701	0,270146	0,816	0,767	1	4	Arhgef1
0,000703	-0,28391	0,301	0,473	1	4	Eif3l
0,000706	-0,31719	0,097	0,234	1	4	Gadd45b
0,000715	-0,29246	0,165	0,328	1	4	Dnmt1
0,000717	-0,27998	0,447	0,629	1	4	Ppp1cc
0,000726	0,278312	0,437	0,292	1	4	Sharpin
0,000731	-0,34263	0,66	0,776	1	4	Atp5b
0,000733	-0,35796	0,34	0,495	1	4	Fermt3
0,000735	0,326704	0,524	0,39	1	4	Rasa3
0,000756	-0,30963	0,689	0,766	1	4	Capzb
0,000759	-0,28855	0,078	0,213	1	4	H2afx
0,000766	-0,36043	0,65	0,784	1	4	Srsf2
0,00077	0,26692	0,282	0,156	1	4	Trim21
0,000786	0,309178	0,437	0,297	1	4	Tmod3
0,000811	-0,35796	0,301	0,459	1	4	Psip1

0,000841	-0,3109	0,252	0,414	1	4	Tpd52
0,000842	0,269545	0,505	0,348	1	4	Abhd17b
0,000844	-0,30847	0,223	0,379	1	4	Usmg5
0,000848	-0,2712	0,175	0,331	1	4	Ak2
0,00085	-0,28442	0,058	0,185	1	4	Smim20
0,000867	-0,26623	1	0,991	1	4	Cd52
0,000882	-0,27622	0,35	0,493	1	4	Parp1
0,00089	-0,27862	0,136	0,287	1	4	Mrpl42
0,00092	-0,253	0,146	0,298	1	4	Tmem14c
0,000932	0,349943	0,573	0,452	1	4	Dgkd
0,000938	-0,27685	0,272	0,442	1	4	Atp5k
0,000941	0,346354	0,33	0,202	1	4	Phf11b
0,000947	-0,27517	0,146	0,304	1	4	Trp53
0,000948	0,290893	0,408	0,271	1	4	Ppp3cc
0,000953	-0,3327	0,709	0,787	1	4	Clic1
0,000958	0,352311	0,466	0,325	1	4	Zmym5
0,000964	-0,25245	0,243	0,411	1	4	Fam107b
0,00097	-0,30892	0,505	0,672	1	4	Prdx1
0,000974	-0,28273	0,214	0,37	1	4	AI662270
0,000982	-0,3559	0,068	0,196	1	4	Dhfr
0,001001	-0,46199	0,689	0,798	1	4	Tubb5
0,001004	-0,35031	0,534	0,671	1	4	Ppp1ca
0,001025	-0,28381	0,243	0,421	1	4	Slc25a4
0,001028	-0,30402	0,553	0,668	1	4	Eif3h
0,001035	-0,30424	0,194	0,351	1	4	Bcl7a
0,001049	-0,30861	0,087	0,221	1	4	Siah2
0,00106	-0,25894	0,165	0,32	1	4	Eif1ax
0,001063	-0,29221	0,204	0,356	1	4	Rps27l
0,001075	0,261273	0,282	0,164	1	4	H2-K2
0,001102	-0,2646	0,058	0,183	1	4	Ngfrap1
0,001127	-0,31496	0,524	0,668	1	4	Cox6c
0,001131	-0,34415	0,35	0,497	1	4	Ghitm
0,001136	0,31151	0,524	0,376	1	4	Cbfa2t3
0,00114	-0,33418	0,379	0,524	1	4	Gng5
0,001146	-0,26433	0,126	0,266	1	4	Ube2m
0,001159	0,37882	0,505	0,384	1	4	Il4ra
0,001161	0,440128	0,68	0,588	1	4	Etnk1
0,001175	0,326023	0,437	0,297	1	4	Trim12a
0,001187	-0,33667	0,485	0,618	1	4	Myl12a
0,001191	0,281631	0,816	0,72	1	4	Iqgap1
0,001216	-0,27925	0,184	0,345	1	4	H2afv
0,001224	-0,27374	0,476	0,612	1	4	Prkcd
0,00125	0,31188	0,612	0,469	1	4	Mgat1
0,001255	0,352909	0,466	0,32	1	4	Pde4b
0,001256	0,318521	0,553	0,422	1	4	Nrd1
0,001271	-0,27158	0,126	0,268	1	4	Trappc1
0,001295	-0,25865	0,33	0,492	1	4	Lbr
0,001296	0,314409	0,66	0,562	1	4	Napsa
0,001332	-0,25732	0,117	0,253	1	4	Isyna1
0,001338	0,261315	0,718	0,561	1	4	Arhgap4

0,001353	0,270026	0,272	0,16	1	4	Gsdmd
0,001372	-0,34455	0,194	0,338	1	4	Mcm7
0,001405	-0,30317	0,301	0,45	1	4	Cmpk1
0,001407	-0,29666	0,262	0,407	1	4	Usf2
0,001434	-0,33236	0,184	0,327	1	4	Lta
0,001444	-0,26414	0,126	0,267	1	4	Zdhhc6
0,001467	-0,31941	0,107	0,238	1	4	Ncapd2
0,001477	-0,34752	0,359	0,506	1	4	Pgk1
0,001569	-0,31863	0,379	0,533	1	4	Tceb2
0,001609	0,334795	0,951	0,886	1	4	Zfp36l1
0,001624	-0,35464	0,252	0,392	1	4	Il21r
0,001639	-0,27638	0,806	0,866	1	4	Naca
0,001663	0,360376	0,767	0,671	1	4	Fam111a
0,00167	-0,26117	0,136	0,272	1	4	Fam96a
0,00168	-0,33165	0,243	0,382	1	4	Naa50
0,001703	-0,29538	0,243	0,385	1	4	Sdhb
0,001719	0,303514	0,379	0,249	1	4	A230046K03Rik
0,001737	-0,26275	0,922	0,948	1	4	Rps26
0,00176	-0,27022	0,039	0,148	1	4	Tpx2
0,00179	0,279019	0,874	0,852	1	4	Eif4a2
0,001798	0,329105	0,621	0,481	1	4	Irf2
0,001827	0,305394	0,631	0,499	1	4	Gpr18
0,001869	-0,35642	0,447	0,556	1	4	Atp5j
0,001879	0,412691	0,466	0,327	1	4	St3gal1
0,001899	0,284765	0,155	0,074	1	4	Map3k8
0,00193	0,318608	0,592	0,452	1	4	Slc44a2
0,001946	0,29562	0,369	0,244	1	4	Aff3
0,001956	-0,31973	0,252	0,383	1	4	Ube2n
0,001972	-0,30235	0,078	0,199	1	4	Cpm
0,00201	-0,36563	0,359	0,5	1	4	Tmpo
0,002049	-0,31339	0,34	0,462	1	4	Atp5j2
0,002071	-0,41895	0,126	0,25	1	4	Mcm5
0,002102	0,32832	0,379	0,253	1	4	Hltf
0,00211	-0,31067	0,165	0,294	1	4	Trib2
0,002127	0,281448	0,379	0,254	1	4	Arhgap15
0,002137	-0,30245	0,175	0,308	1	4	Cetn3
0,002171	-0,32536	0,311	0,441	1	4	Ube2j1
0,002179	-0,27245	0,291	0,441	1	4	Phf6
0,002206	0,298804	0,379	0,249	1	4	Wdfy1
0,002214	-0,30032	0,252	0,401	1	4	Cxcr4
0,002319	-0,27818	0,311	0,473	1	4	Eif4a1
0,002389	0,329226	0,388	0,26	1	4	Man1a
0,002393	0,337825	0,427	0,308	1	4	Rnase6
0,002466	-0,2715	0,252	0,396	1	4	Prelid1
0,002476	-0,33732	0,301	0,434	1	4	Mapre2
0,002512	0,309488	0,505	0,377	1	4	Clic4
0,002604	0,280726	0,485	0,361	1	4	Mllt6
0,00284	-0,26944	0,359	0,505	1	4	Atp5f1
0,002856	-0,39788	0,563	0,671	1	4	Srsf11
0,002891	-0,3108	0,408	0,521	1	4	Eif2s2

0,002994	-0,35895	0,456	0,613	1	4	Gna13
0,003024	0,381592	0,476	0,347	1	4	Ankrd44
0,003066	-0,26347	0,398	0,546	1	4	Psma7
0,00308	0,275382	0,359	0,244	1	4	Clec2d
0,003085	-0,29759	0,155	0,277	1	4	Ap3b1
0,003144	-0,26376	0,155	0,295	1	4	Ehd4
0,003181	-0,26652	0,32	0,452	1	4	Azin1
0,003241	-0,2534	0,252	0,395	1	4	Vdac3
0,003325	0,284478	0,592	0,456	1	4	Pld4
0,003335	-0,29311	0,398	0,519	1	4	Snrpb
0,003438	-0,26992	0,155	0,288	1	4	Cstb
0,003451	-0,30503	0,272	0,415	1	4	Edem1
0,003468	-0,25273	0,146	0,272	1	4	Cyb5b
0,00351	-0,31697	0,136	0,257	1	4	Psat1
0,003613	-0,25201	0,262	0,396	1	4	Pptc7
0,003661	-0,26772	0,136	0,267	1	4	Tti1
0,00367	-0,26374	0,311	0,445	1	4	Pa2g4
0,003694	-0,25529	0,204	0,338	1	4	Lck
0,003696	-0,29216	0,233	0,373	1	4	Rraga
0,003752	-0,31192	0,165	0,289	1	4	C130026I21Rik
0,003755	-0,27398	0,34	0,471	1	4	Ptbp1
0,00378	0,349382	0,456	0,364	1	4	Trim12c
0,003789	-0,30175	0,398	0,547	1	4	Apobec1
0,003824	-0,25558	0,32	0,461	1	4	Ndufa2
0,003835	-0,30078	0,476	0,597	1	4	Ap2m1
0,003888	-0,28479	0,32	0,457	1	4	Cdk2ap1
0,003929	-0,31858	0,126	0,241	1	4	Fam214a
0,003965	0,324724	0,689	0,589	1	4	Zbtb20
0,004077	0,294761	0,728	0,631	1	4	Psme1
0,004106	0,304841	0,524	0,414	1	4	1810026B05Rik
0,004163	-0,28197	0,184	0,305	1	4	Akap2
0,004224	-0,27834	0,806	0,849	1	4	Set
0,004324	-0,28651	0,311	0,426	1	4	Eif2s1
0,004467	-0,34385	0,301	0,421	1	4	Ndufc2
0,004476	0,339656	0,262	0,17	1	4	Dlgap4
0,004656	-0,2598	0,165	0,287	1	4	Ahcy
0,004716	0,36471	0,291	0,19	1	4	Hbp1
0,00478	-0,25147	0,204	0,342	1	4	Dynlt3
0,004993	-0,33281	0,34	0,45	1	4	Sept7
0,005	0,27026	0,35	0,23	1	4	Nin
0,005005	-0,34764	0,379	0,501	1	4	Wdr92
0,005485	-0,32331	0,398	0,51	1	4	Rbbp7
0,005856	-0,27293	0,544	0,65	1	4	Ikzf1
0,005874	0,308091	0,495	0,381	1	4	Chd7
0,005962	-0,26018	0,495	0,591	1	4	Chd4
0,006018	-0,25388	0,35	0,477	1	4	Mapre1
0,00605	-0,28835	0,301	0,416	1	4	Abracl
0,00606	-0,27124	0,476	0,608	1	4	Hnrnpm
0,006334	-0,2961	0,204	0,32	1	4	Whsc1
0,006731	0,252998	0,806	0,747	1	4	Sp110

0,006744	-0,2715	0,612	0,72	1	4	Eif3e
0,006858	-0,30505	0,466	0,557	1	4	Snrpe
0,007005	-0,26496	0,621	0,733	1	4	Hnrnpu
0,007113	0,307926	0,883	0,824	1	4	Lax1
0,007764	0,371467	0,408	0,311	1	4	Atm
0,007913	-0,26374	0,359	0,469	1	4	Hdac1
0,007939	0,27569	0,34	0,232	1	4	Zscan26
0,007997	-0,27094	0,524	0,669	1	4	Syk
0,008409	-0,36288	0,408	0,516	1	4	Xist
0,008438	0,330345	0,738	0,648	1	4	Phip
0,00853	0,276047	0,932	0,928	1	4	Sf3b1
0,008577	-0,26612	0,621	0,707	1	4	Srsf1
0,008616	-0,28057	0,272	0,39	1	4	Tmem179b
0,009083	-0,26634	0,078	0,175	1	4	Pno1
0,009694	0,254885	0,447	0,339	1	4	Pdcd10
0,009811	-0,27754	0,146	0,252	1	4	Med30
0,009849	0,26692	0,408	0,299	1	4	Gmfg
3,7E-175	1,353254	0,783	0,022	4,6E-171	5	Cdc20
1,9E-138	1,320754	0,717	0,026	2,3E-134	5	Aspm
6,8E-117	1,254295	0,717	0,035	8,4E-113	5	Cdkn3
2,84E-91	2,153762	0,95	0,112	3,52E-87	5	Ccnb2
1,04E-87	1,442766	0,867	0,086	1,29E-83	5	Cks1b
9,68E-84	0,703971	0,483	0,021	1,2E-79	5	Kif20a
3,24E-80	0,559016	0,3	0,005	4,01E-76	5	Pif1
4,28E-77	1,319443	0,7	0,059	5,29E-73	5	Ccnb1
1,26E-76	1,44074	0,8	0,083	1,56E-72	5	Cdca8
2,14E-76	1,12227	0,567	0,036	2,65E-72	5	Nek2
1,3E-71	1,152452	0,617	0,048	1,61E-67	5	Cep55
1,36E-70	0,754048	0,383	0,015	1,69E-66	5	Kif2c
3,19E-69	1,667609	0,883	0,12	3,95E-65	5	Cenpe
1,43E-68	1,696759	0,867	0,119	1,77E-64	5	Tpx2
1,82E-68	1,009681	0,467	0,026	2,26E-64	5	Plk1
1,3E-66	1,067909	0,533	0,037	1,61E-62	5	Ckap2l
3,05E-64	1,493736	0,733	0,08	3,78E-60	5	Ccna2
1,5E-59	1,076014	0,6	0,056	1,86E-55	5	C330027C09Rik
4,66E-59	1,755693	0,9	0,164	5,77E-55	5	Cenpa
2,06E-56	0,823725	0,483	0,036	2,55E-52	5	Knstrn
3,35E-55	0,98141	0,55	0,052	4,15E-51	5	Dlgap5
1,19E-54	0,436321	0,267	0,008	1,47E-50	5	Sapcd2
4,56E-52	1,242782	0,683	0,09	5,64E-48	5	Cdca3
8,58E-50	1,248319	0,65	0,085	1,06E-45	5	Kif23
6,43E-49	0,532953	0,283	0,012	7,96E-45	5	Cdc25c
7,8E-49	2,059954	0,867	0,199	9,66E-45	5	Cks2
4,15E-48	1,659385	0,783	0,145	5,14E-44	5	Ube2c
9,03E-47	1,189978	0,75	0,12	1,12E-42	5	Racgap1
4,93E-46	1,270106	0,583	0,073	6,1E-42	5	Cenpf
1,76E-44	0,803662	0,483	0,048	2,18E-40	5	Kif4
2,69E-43	2,135644	0,967	0,294	3,33E-39	5	Mki67
4,85E-43	1,171251	0,733	0,129	6E-39	5	Birc5
3,71E-42	0,768475	0,517	0,059	4,6E-38	5	Bub1b

5,2E-42	0,773028	0,483	0,052	6,43E-38	5	Gpsm2
1,91E-41	2,21351	1	0,444	2,36E-37	5	Hmgb2
2,01E-41	0,955505	0,5	0,057	2,49E-37	5	Ckap2
1,61E-40	0,367607	0,283	0,016	1,99E-36	5	Arhgap19
1,43E-39	0,355187	0,25	0,012	1,78E-35	5	Gtse1
1,93E-39	0,367931	0,2	0,007	2,4E-35	5	Fam64a
5,3E-39	1,233554	0,6	0,094	6,56E-35	5	Hmmr
7,17E-35	0,306087	0,2	0,008	8,87E-31	5	Tbc1d4
2,47E-34	1,812536	1	0,73	3,05E-30	5	Hmgn2
2,53E-33	0,336572	0,233	0,013	3,13E-29	5	Parpbp
7,92E-33	0,561951	0,367	0,037	9,8E-29	5	Bub1
1,93E-32	1,583958	0,95	0,365	2,39E-28	5	Stmn1
2,14E-30	0,987525	0,817	0,212	2,65E-26	5	Ncapd2
1,92E-29	1,216682	0,867	0,28	2,37E-25	5	Ube2s
3,95E-29	1,182075	0,9	0,319	4,89E-25	5	H2afv
9,91E-29	0,836496	0,517	0,088	1,23E-24	5	Dbf4
3,53E-28	0,605128	0,483	0,077	4,37E-24	5	Hspa2
3,91E-28	1,518362	1	0,671	4,84E-24	5	H2afz
6,38E-28	0,466543	0,267	0,023	7,9E-24	5	Gas2l3
1,63E-27	0,603143	0,45	0,068	2,01E-23	5	Cenpw
2,73E-27	0,44978	0,333	0,037	3,38E-23	5	Ccrl1
4,07E-27	0,497878	0,267	0,024	5,04E-23	5	1190002F15Rik
9,94E-27	0,6599	0,583	0,115	1,23E-22	5	Ckap5
2,11E-26	0,708287	0,45	0,072	2,61E-22	5	Nup37
3,31E-26	0,967137	0,767	0,236	4,09E-22	5	2700094K13Rik
6,23E-26	1,047613	0,7	0,19	7,71E-22	5	H2afx
8,78E-26	0,596242	0,35	0,044	1,09E-21	5	Espl1
1,18E-25	0,787694	0,417	0,064	1,46E-21	5	Spag5
1,4E-25	0,416738	0,3	0,032	1,74E-21	5	Kif22
2,02E-25	1,39404	0,867	0,381	2,5E-21	5	Rad21
7,37E-25	0,688273	0,433	0,07	9,13E-21	5	Nusap1
1,09E-24	0,42361	0,217	0,017	1,35E-20	5	Fam72a
1,12E-24	1,198187	0,95	0,443	1,39E-20	5	Nucks1
5,42E-24	0,401448	0,283	0,03	6,71E-20	5	Shcbp1
5,96E-23	0,620981	0,333	0,045	7,38E-19	5	Kif18a
6,06E-23	1,544511	0,85	0,344	7,51E-19	5	Tubb4b
1,46E-22	1,171186	0,85	0,341	1,8E-18	5	Pafah1b3
4,7E-22	-0,91768	1	1	5,82E-18	5	H2-Aa
7,18E-22	0,509597	0,317	0,042	8,89E-18	5	Sgol1
1,66E-21	0,552871	0,283	0,035	2,05E-17	5	Ect2
1,99E-21	1,124904	0,983	0,864	2,46E-17	5	Cd24a
7,02E-21	0,704201	0,55	0,129	8,69E-17	5	Tacc3
7,41E-21	1,087235	0,983	0,791	9,17E-17	5	Hmgb1
1,3E-20	0,561253	0,233	0,025	1,61E-16	5	Troap
2,16E-20	0,831191	0,517	0,124	2,68E-16	5	Nuf2
2,84E-20	1,192104	0,867	0,426	3,52E-16	5	Anp32e
5,3E-20	0,962205	0,85	0,336	6,57E-16	5	Polr2g
7,31E-20	0,969391	0,95	0,41	9,05E-16	5	Gcsam
1,72E-19	1,292901	0,883	0,421	2,13E-15	5	8430410A17Rik
3,63E-19	0,67531	0,4	0,076	4,5E-15	5	Kif11

7,58E-19	1,052299	0,967	0,572	9,38E-15	5	Mbd2
1,92E-18	1,027192	0,917	0,434	2,38E-14	5	Rgs13
2,21E-18	0,967015	0,683	0,24	2,74E-14	5	Acsl5
2,69E-18	0,854199	1	0,942	3,33E-14	5	Ptma
4,18E-18	0,872332	0,567	0,161	5,18E-14	5	H1fx
1,45E-17	0,968225	0,85	0,373	1,79E-13	5	Anxa2
5,45E-17	1,093599	0,9	0,439	6,75E-13	5	Dck
7,82E-17	0,844324	0,45	0,111	9,68E-13	5	Prc1
7,94E-17	0,901496	0,933	0,62	9,83E-13	5	Ran
1,38E-16	0,948276	0,883	0,471	1,71E-12	5	Lbr
1,43E-16	0,997863	0,65	0,229	1,78E-12	5	Cdc14b
2,04E-16	0,712786	0,583	0,185	2,52E-12	5	Amz2
4,13E-16	0,948824	0,983	0,789	5,11E-12	5	Pou2af1
4,83E-16	0,909052	0,767	0,343	5,98E-12	5	Smarca4
4,89E-16	0,627771	0,25	0,037	6,06E-12	5	Ccdc18
8E-16	0,943829	1	0,655	9,9E-12	5	Igj
1,23E-15	-1,55053	0,867	0,975	1,52E-11	5	Malat1
1,6E-15	0,766752	0,983	0,981	1,98E-11	5	Hnrnpa2b1
1,96E-15	0,827245	0,983	0,733	2,43E-11	5	Ywhae
2,09E-15	0,276753	0,3	0,051	2,58E-11	5	Ddah2
2,11E-15	0,528968	0,433	0,108	2,62E-11	5	Arhgap11a
2,48E-15	0,44209	0,2	0,025	3,07E-11	5	Aurka
3,76E-15	0,63524	0,4	0,096	4,65E-11	5	C230052I12Rik
7,92E-15	0,592302	0,617	0,199	9,81E-11	5	Lsm5
1,15E-14	0,839447	0,833	0,422	1,43E-10	5	Glrx3
1,94E-14	0,603744	0,333	0,07	2,41E-10	5	Kif15
2,9E-14	0,768981	0,75	0,321	3,59E-10	5	Dynlt3
3,07E-14	0,710604	1	0,965	3,8E-10	5	Calm1
4,28E-14	0,884109	0,717	0,31	5,3E-10	5	Hdgf
4,65E-14	0,777921	0,783	0,373	5,76E-10	5	Calm3
5,18E-14	0,662342	0,267	0,048	6,41E-10	5	Sgol2
5,39E-14	0,674643	0,417	0,109	6,67E-10	5	Cdk1
6,72E-14	-1,34776	0,533	0,832	8,31E-10	5	Macf1
6,91E-14	0,667889	0,35	0,079	8,56E-10	5	Lig4
7,24E-14	0,524278	0,367	0,086	8,97E-10	5	G2e3
1,55E-13	0,857613	0,733	0,331	1,92E-09	5	Trim59
1,67E-13	1,059099	0,533	0,186	2,07E-09	5	Tuba1c
1,77E-13	0,57702	0,417	0,111	2,19E-09	5	Incenp
2,23E-13	0,582689	0,367	0,089	2,75E-09	5	Kif20b
2,77E-13	0,761887	0,717	0,308	3,43E-09	5	Hp1bp3
3,56E-13	0,42254	0,3	0,06	4,4E-09	5	Reln
4,33E-13	0,698918	1	0,999	5,36E-09	5	Cfl1
5,1E-13	0,412546	0,317	0,068	6,31E-09	5	Otub2
6,53E-13	0,929806	0,95	0,635	8,09E-09	5	Top1
7,36E-13	0,781809	0,967	0,687	9,11E-09	5	Txn1
7,66E-13	0,64979	0,6	0,221	9,48E-09	5	Abl2
7,71E-13	0,512247	0,283	0,057	9,55E-09	5	Mis18bp1
8,58E-13	0,419801	0,35	0,081	1,06E-08	5	Hmgb3
1,02E-12	0,854518	0,917	0,626	1,26E-08	5	Hmgn1
1,79E-12	0,637287	0,65	0,251	2,22E-08	5	Nuggc

1,9E-12	0,67848	0,667	0,263	2,35E-08	5	Vpreb3
3,14E-12	-0,93907	0,867	0,96	3,89E-08	5	Cd22
3,29E-12	0,362396	0,25	0,047	4,08E-08	5	Hist1h2bc
3,66E-12	-1,07617	0,217	0,668	4,53E-08	5	Ifi30
4,05E-12	0,479674	0,25	0,048	5,02E-08	5	Sord
5,33E-12	0,362379	0,317	0,072	6,6E-08	5	Ccdc77
8,4E-12	0,450759	0,35	0,088	1,04E-07	5	L3mbtl2
8,53E-12	0,341842	0,233	0,042	1,06E-07	5	Ttk
9,15E-12	0,774036	0,7	0,334	1,13E-07	5	Dap
1,13E-11	0,544032	0,6	0,216	1,4E-07	5	2810417H13Rik
1,34E-11	0,356699	0,267	0,055	1,66E-07	5	Cenpl
1,58E-11	0,633652	0,567	0,206	1,95E-07	5	Mad2l1
1,68E-11	0,872959	0,867	0,574	2,08E-07	5	Hn1
3,42E-11	0,68969	0,967	0,767	4,24E-07	5	Gapdh
3,81E-11	0,27031	0,15	0,019	4,72E-07	5	Kif14
3,85E-11	0,334739	0,267	0,057	4,76E-07	5	Rnf26
5,2E-11	-0,70437	0,967	0,993	6,44E-07	5	Cd52
5,62E-11	-1,22917	0,317	0,688	6,95E-07	5	Gimap4
5,99E-11	0,629654	0,983	0,944	7,41E-07	5	Eif4g2
6,35E-11	0,449049	0,4	0,118	7,86E-07	5	Fam216a
7,22E-11	0,739609	0,533	0,207	8,94E-07	5	Nde1
7,66E-11	0,712356	0,617	0,295	9,48E-07	5	Vbp1
8,75E-11	-0,45763	1	1	1,08E-06	5	H2-Ab1
9,74E-11	0,532132	0,417	0,134	1,21E-06	5	Odf2
1,14E-10	0,378024	0,267	0,059	1,41E-06	5	Rgcc
1,23E-10	0,57073	0,417	0,134	1,53E-06	5	Uap1
1,27E-10	0,298274	0,2	0,035	1,57E-06	5	4933404O12Rik
1,3E-10	-1,04846	0,45	0,804	1,61E-06	5	Mycbp2
1,33E-10	0,529349	1	0,997	1,65E-06	5	Actg1
1,55E-10	0,771348	0,867	0,566	1,92E-06	5	Cyfip2
1,56E-10	0,642529	0,733	0,365	1,93E-06	5	Taf9
1,78E-10	0,567677	0,667	0,287	2,2E-06	5	Rassf6
1,93E-10	0,387536	0,3	0,076	2,39E-06	5	Akap12
1,98E-10	0,411177	0,417	0,129	2,45E-06	5	Ripk3
2,04E-10	0,870447	0,667	0,32	2,53E-06	5	Ptms
2,7E-10	0,420226	0,267	0,062	3,34E-06	5	Cdca2
2,72E-10	0,414387	0,483	0,168	3,37E-06	5	Acyp1
2,75E-10	-1,10935	0,233	0,615	3,41E-06	5	Lmo2
3,14E-10	-0,90233	0,483	0,763	3,89E-06	5	Hvcn1
3,55E-10	0,542678	0,683	0,295	4,39E-06	5	Rnf10
4,04E-10	0,489081	0,55	0,205	5,01E-06	5	4833439L19Rik
4,15E-10	0,652867	0,783	0,422	5,14E-06	5	Phf6
4,26E-10	0,64935	0,717	0,363	5,27E-06	5	Dbi
4,51E-10	0,472026	0,4	0,129	5,58E-06	5	S100a13
4,76E-10	-0,84723	0,8	0,92	5,89E-06	5	Ptpn6
6,07E-10	-1,42628	0,1	0,512	7,52E-06	5	Mndal
6,84E-10	0,629536	0,967	0,787	8,47E-06	5	Atp5a1
7,12E-10	0,630179	0,767	0,392	8,82E-06	5	Odc1
7,14E-10	0,741651	0,617	0,262	8,84E-06	5	Top2a
7,71E-10	-0,6347	1	0,988	9,54E-06	5	Rpl38

8,37E-10	0,478155	0,467	0,171	1,04E-05	5	Ddx19a
8,51E-10	0,560146	0,6	0,266	1,05E-05	5	Tuba1a
8,62E-10	0,720889	0,733	0,382	1,07E-05	5	Cxcr4
9,23E-10	0,711541	0,933	0,736	1,14E-05	5	Dynll1
1,02E-09	0,96682	0,567	0,236	1,27E-05	5	Kpna2
1,04E-09	0,582137	0,983	0,911	1,29E-05	5	Ppia
1,17E-09	0,343276	0,183	0,033	1,45E-05	5	Adhfe1
1,24E-09	-0,89915	0,317	0,69	1,53E-05	5	Pisd-ps1
1,31E-09	0,439852	0,35	0,106	1,62E-05	5	Csrp2
1,52E-09	0,671909	0,867	0,492	1,88E-05	5	Aicda
1,62E-09	0,578243	0,583	0,263	2,01E-05	5	Smagp
1,98E-09	0,283753	0,15	0,023	2,45E-05	5	1500015A07Rik
2,09E-09	0,250167	0,217	0,045	2,59E-05	5	Ubald2
2,19E-09	0,698678	0,833	0,576	2,71E-05	5	Tcea1
2,26E-09	0,398719	0,3	0,081	2,8E-05	5	Ccdc34
2,53E-09	-0,4344	1	1	3,13E-05	5	H2-Eb1
3,16E-09	0,61256	0,733	0,417	3,92E-05	5	Mapre2
3,49E-09	0,436463	0,517	0,2	4,32E-05	5	Pqlc3
3,58E-09	0,499576	0,7	0,342	4,43E-05	5	Cox7a2
3,85E-09	0,413979	0,533	0,207	4,77E-05	5	Rgs10
4,08E-09	0,311481	0,217	0,046	5,05E-05	5	Ska2
4,45E-09	-1,31641	0,183	0,539	5,51E-05	5	Ly6d
4,88E-09	-1,11249	0,6	0,836	6,04E-05	5	Shisa5
5,29E-09	0,645299	0,533	0,238	6,55E-05	5	Sft2d2
5,33E-09	-0,89862	0,35	0,691	6,6E-05	5	Scd1
5,41E-09	0,476156	1	0,992	6,7E-05	5	Pabpc1
5,86E-09	0,503951	0,567	0,242	7,26E-05	5	Plxnb2
5,98E-09	0,558174	0,683	0,351	7,4E-05	5	Cwc15
5,99E-09	0,585716	0,683	0,346	7,42E-05	5	Gtf2a2
6,02E-09	0,610978	0,85	0,517	7,45E-05	5	Mtf2
6,06E-09	0,607459	0,983	0,809	7,5E-05	5	Hnrnpa3
6,18E-09	0,516012	0,65	0,297	7,66E-05	5	Rgs2
6,23E-09	0,37992	0,5	0,188	7,71E-05	5	Ddx47
6,35E-09	-0,36013	1	1	7,86E-05	5	Cd74
8,18E-09	0,408218	0,3	0,086	0,000101	5	Spc24
9,26E-09	-0,86512	0,683	0,853	0,000115	5	Pou2f2
9,68E-09	0,649578	0,567	0,254	0,00012	5	Larp7
1,03E-08	0,592213	0,683	0,346	0,000127	5	Sh3bgrl
1,05E-08	0,354927	0,383	0,127	0,00013	5	Rabl5
1,06E-08	0,348266	0,2	0,042	0,000131	5	Ccnf
1,21E-08	1,110576	0,75	0,528	0,00015	5	Smc4
1,31E-08	0,358674	0,383	0,126	0,000162	5	Nup107
1,43E-08	0,319258	0,333	0,1	0,000177	5	Emp2
1,45E-08	0,336385	0,283	0,077	0,000179	5	Ift80
2,17E-08	-0,57728	1	0,998	0,000269	5	Cd19
2,29E-08	0,493433	0,567	0,25	0,000284	5	Nudt21
2,51E-08	0,523502	0,5	0,219	0,000311	5	Rangap1
2,52E-08	-1,31326	0,3	0,588	0,000312	5	Cmah
2,7E-08	0,526022	0,633	0,306	0,000334	5	Eaf2
2,71E-08	0,604777	0,65	0,369	0,000335	5	Sdhb

4,13E-08	0,916843	0,8	0,621	0,000511	5	Calm2
4,17E-08	0,506045	0,983	0,778	0,000516	5	Mzb1
4,34E-08	0,440579	0,55	0,251	0,000537	5	Cdkn2aipnl
4,53E-08	0,495457	0,467	0,196	0,000561	5	Eny2
4,72E-08	0,636197	0,867	0,679	0,000584	5	Mtpn
5,03E-08	0,486685	0,917	0,634	0,000623	5	Zfp706
5,05E-08	0,441892	0,467	0,191	0,000625	5	Med10
5,1E-08	0,455896	1	0,989	0,000632	5	Eif1
5,13E-08	-0,89096	0,067	0,419	0,000634	5	Capg
5,15E-08	-0,56849	0,983	0,992	0,000638	5	H2-Ob
5,57E-08	0,813167	0,7	0,442	0,000689	5	Hipk3
5,9E-08	0,572942	0,933	0,815	0,00073	5	Hsp90aa1
6,43E-08	0,376945	0,35	0,12	0,000795	5	Msrb1
6,51E-08	0,459127	0,417	0,164	0,000806	5	Lmnb1
6,63E-08	0,544795	0,317	0,104	0,000821	5	Ccdc104
6,84E-08	-0,99821	0,267	0,582	0,000847	5	Bank1
6,99E-08	0,471983	0,6	0,271	0,000866	5	Lipc
7,08E-08	0,619562	0,717	0,429	0,000876	5	Cdk2ap2
7,33E-08	-0,56827	0,967	0,995	0,000907	5	Rps21
7,52E-08	0,469083	0,6	0,28	0,000931	5	Oat
8,3E-08	-0,56612	0,967	0,98	0,001028	5	Rpl37a
8,36E-08	0,259887	0,25	0,067	0,001035	5	Cenpn
8,82E-08	-1,07051	0,133	0,477	0,001092	5	Pml
9,42E-08	-0,78241	0,767	0,893	0,001166	5	Zfp36l1
1,02E-07	0,547118	0,65	0,353	0,001264	5	Ube2h
1,06E-07	-1,17553	0,167	0,487	0,001313	5	Ifi203
1,15E-07	0,359138	0,4	0,148	0,001427	5	Pih1d1
1,15E-07	0,256285	0,15	0,028	0,001429	5	Dnase1
1,21E-07	0,431822	0,583	0,279	0,001493	5	Ddx39
1,34E-07	0,330783	0,267	0,078	0,00166	5	Ap3s1
1,38E-07	0,603825	0,767	0,461	0,001711	5	Mapre1
1,45E-07	0,557888	0,85	0,571	0,001793	5	Ppp2ca
1,67E-07	0,491519	0,683	0,373	0,002063	5	Uqcrfs1
1,79E-07	0,305013	0,4	0,148	0,002211	5	Rpa3
1,82E-07	0,510137	0,517	0,238	0,002249	5	Med30
1,85E-07	0,370809	0,433	0,172	0,002287	5	Hmgcs1
2,14E-07	0,365285	0,283	0,088	0,002647	5	Aurkb
2,2E-07	0,557476	0,867	0,689	0,002718	5	Hnrnpk
2,36E-07	-0,82732	0,55	0,773	0,002916	5	Btla
2,97E-07	-1,06082	0,35	0,639	0,003682	5	Fcer2a
3,04E-07	-0,80842	0,033	0,354	0,003757	5	Map3k1
3,14E-07	-0,36902	1	0,999	0,003883	5	Rpl27a
3,28E-07	0,494677	0,983	0,783	0,004065	5	Srsf3
3,32E-07	0,521992	0,4	0,16	0,004114	5	Ncbp2
3,33E-07	-0,90821	0,017	0,334	0,004127	5	Bcl2
3,36E-07	0,634532	0,75	0,479	0,004165	5	Wbp2
3,37E-07	0,296413	0,233	0,064	0,004177	5	Casc5
3,5E-07	0,341244	0,333	0,117	0,004329	5	Myef2
3,84E-07	-0,85892	0,833	0,946	0,004756	5	A630089N07Rik
4,12E-07	-1,16184	0,117	0,428	0,005104	5	Sell

4,57E-07	0,3127	0,35	0,124	0,005655	5	Gnb4
4,83E-07	-0,73507	0,35	0,627	0,005979	5	Tbc1d10c
5,43E-07	-0,70542	0,467	0,689	0,006717	5	Srrm2
5,46E-07	0,405495	0,633	0,325	0,006759	5	Qars
5,61E-07	0,566924	0,883	0,722	0,006949	5	Atp5g3
5,63E-07	0,32616	0,4	0,156	0,006972	5	Hmgcr
5,71E-07	-0,66004	0,567	0,763	0,007068	5	Gas5
5,83E-07	0,450661	0,6	0,314	0,007219	5	Cpsf2
5,95E-07	0,27735	0,2	0,052	0,007363	5	Prr11
6,34E-07	0,51706	0,45	0,208	0,007843	5	Cul4b
6,47E-07	0,36997	0,45	0,191	0,00801	5	Suv39h1
6,54E-07	-0,86092	0,633	0,799	0,008091	5	Faim3
6,55E-07	-0,57794	0,867	0,937	0,008108	5	Rpl36
6,93E-07	-0,97644	0,55	0,736	0,008585	5	Ly6a
7,02E-07	0,385776	0,483	0,214	0,008695	5	Snx10
7,8E-07	0,325821	0,467	0,198	0,009662	5	Lsm3
7,81E-07	-0,78005	0,567	0,75	0,009672	5	Bcl11a
7,96E-07	-0,88034	0,05	0,365	0,00986	5	Gm1966
8,03E-07	-0,45309	0,967	0,998	0,009939	5	Cd37
9,59E-07	0,251345	0,2	0,053	0,011868	5	Phf7
9,72E-07	0,499234	0,367	0,147	0,012037	5	Reep4
1E-06	0,284585	0,233	0,068	0,012426	5	Cenpv
1,01E-06	0,33692	0,383	0,152	0,01245	5	Tceanc2
1,03E-06	0,475933	0,817	0,532	0,0128	5	Ppp4r2
1,09E-06	0,382187	0,367	0,145	0,013444	5	Fopnl
1,1E-06	0,637421	0,667	0,426	0,013628	5	Ube2j1
1,14E-06	0,357352	0,433	0,183	0,01407	5	Nrbf2
1,25E-06	-0,33832	1	1	0,015495	5	mtNd2
1,28E-06	-0,95185	0,117	0,417	0,015834	5	Klf2
1,29E-06	0,355981	0,433	0,187	0,015913	5	Nubp2
1,32E-06	-0,46388	0,95	0,983	0,01636	5	Rpl22
1,4E-06	0,313312	0,3	0,105	0,017278	5	Blvrb
1,45E-06	0,780937	0,917	0,788	0,017971	5	Tubb5
1,47E-06	0,489149	0,683	0,409	0,018246	5	Rbm38
1,53E-06	0,432274	0,717	0,402	0,018912	5	Slc25a4
1,68E-06	0,560146	0,85	0,713	0,020801	5	Gpx1
1,69E-06	0,384941	0,483	0,219	0,020928	5	Gadd45b
1,71E-06	-0,75055	0,05	0,343	0,021119	5	S100a10
1,75E-06	0,488077	0,7	0,437	0,021683	5	Azin1
1,76E-06	0,420955	0,583	0,295	0,021747	5	Dstn
1,76E-06	0,427204	0,767	0,467	0,021833	5	Ywhah
1,77E-06	0,39246	0,633	0,32	0,021882	5	Stap1
1,86E-06	0,51478	0,95	0,764	0,022971	5	Atp5b
1,9E-06	0,669782	0,583	0,33	0,023477	5	Fundc2
2,17E-06	0,44395	0,5	0,247	0,026921	5	Asf1b
2,18E-06	0,451658	0,667	0,394	0,027002	5	Fam107b
2,43E-06	0,421056	0,983	0,983	0,030102	5	Ucp2
2,44E-06	0,348671	0,533	0,256	0,030216	5	Fam96a
2,51E-06	-0,90579	0,583	0,761	0,031134	5	Il2rg
2,52E-06	-0,81098	0,183	0,493	0,031211	5	Abca1

2,53E-06	0,414824	0,8	0,504	0,031281	5	Shfm1
2,56E-06	0,492839	0,817	0,635	0,031649	5	Ywhaq
2,57E-06	0,420096	0,783	0,479	0,03182	5	Ubl5
2,71E-06	0,275185	0,283	0,097	0,033603	5	Myl4
2,74E-06	0,392798	0,4	0,172	0,033936	5	Mzt1
2,9E-06	0,418835	0,817	0,571	0,035958	5	Oaz1
2,94E-06	0,390044	0,5	0,242	0,036367	5	Psmd6
2,95E-06	-0,70535	0,967	0,993	0,036492	5	Kcnq1ot1
2,98E-06	0,620439	0,833	0,591	0,036915	5	Hnrnpa0
2,99E-06	0,459003	0,917	0,682	0,037045	5	Arpc5l
3E-06	0,356779	0,5	0,231	0,037182	5	Sh2b2
3,13E-06	0,504395	0,517	0,272	0,038688	5	Fgfr1op
3,17E-06	-0,80765	0,133	0,432	0,039268	5	Sub1
3,17E-06	0,259499	0,283	0,097	0,039306	5	Yipf4
3,22E-06	0,383942	1	0,917	0,039892	5	Rbm3
3,36E-06	0,367425	0,55	0,276	0,041574	5	Nup50
3,44E-06	0,440506	0,667	0,379	0,042606	5	Lpp
3,46E-06	0,269906	0,217	0,064	0,042804	5	Pipox
3,49E-06	0,323873	0,35	0,141	0,043186	5	Ccnh
3,71E-06	0,345875	0,3	0,111	0,045917	5	Man1b1
3,8E-06	-0,33387	1	1	0,047065	5	Rpl17
3,8E-06	0,301913	0,4	0,168	0,047096	5	Snrnp25
3,95E-06	0,52202	0,817	0,611	0,048912	5	Cct2
3,98E-06	0,49149	0,933	0,778	0,049319	5	Clic1
4,03E-06	0,453453	0,85	0,577	0,049844	5	Ddx6
4,23E-06	-0,59136	0,517	0,706	0,052335	5	Ctsh
4,24E-06	0,335596	0,367	0,154	0,052497	5	Rhno1
4,29E-06	0,393496	0,383	0,162	0,053165	5	Cpne5
4,38E-06	0,475821	0,65	0,359	0,054245	5	Tmem64
4,57E-06	0,320252	0,433	0,197	0,056545	5	Snrpa1
4,74E-06	-0,3548	1	0,999	0,058727	5	Rpl13a
4,83E-06	0,280038	0,3	0,108	0,059754	5	Ncaph
4,85E-06	0,373409	0,767	0,455	0,060024	5	Eif3l
4,85E-06	0,277269	0,4	0,168	0,06009	5	Pycr2
4,98E-06	0,347417	0,55	0,283	0,061691	5	Aimp1
5E-06	-0,81776	0,233	0,512	0,061853	5	Cd83
5,04E-06	0,341273	0,333	0,131	0,062343	5	Slc43a2
5,15E-06	0,341353	0,433	0,19	0,063801	5	Gng12
5,22E-06	-0,56818	0,65	0,838	0,064644	5	Sipa1
5,49E-06	-0,77589	0,433	0,645	0,067966	5	Lbh
5,91E-06	0,47378	0,683	0,45	0,073129	5	Fkbp8
5,97E-06	0,477684	0,383	0,177	0,07393	5	Fam103a1
6,36E-06	-0,7812	0,133	0,407	0,07868	5	B3gnt5
6,45E-06	0,488307	0,75	0,507	0,079818	5	Lsm14a
6,74E-06	0,455814	0,783	0,538	0,083404	5	Paip2
6,77E-06	0,505284	0,633	0,38	0,083803	5	Metap2
6,93E-06	0,510518	0,933	0,779	0,085811	5	Anp32b
7,19E-06	0,372939	0,6	0,321	0,089019	5	Osbpl9
7,2E-06	-0,67138	0,35	0,636	0,089158	5	Ciita
7,35E-06	0,391645	0,517	0,257	0,091011	5	Cyb5b

7,44E-06	0,475098	0,7	0,441	0,092108	5	Csnk2b
7,49E-06	-0,57634	0,333	0,575	0,09269	5	Napsa
7,54E-06	0,437037	0,733	0,436	0,09337	5	Bcl6
7,61E-06	0,350902	0,517	0,256	0,094186	5	Ssr3
7,94E-06	0,278972	0,367	0,151	0,098274	5	Prmt5
8,26E-06	0,554213	0,683	0,438	0,102253	5	Tmed5
8,36E-06	-0,56777	0,2	0,483	0,103509	5	Acap1
8,47E-06	0,361379	0,55	0,282	0,104894	5	Hsbp1
8,51E-06	0,405973	0,867	0,583	0,105301	5	Zfp207
8,85E-06	0,448844	0,75	0,48	0,109601	5	Cbx3
9,05E-06	0,267178	0,367	0,149	0,112063	5	Smco4
9,34E-06	0,66208	0,717	0,534	0,115625	5	Apobec1
9,35E-06	0,333756	0,25	0,086	0,11581	5	Ncapg
9,77E-06	0,442285	0,45	0,227	0,120969	5	Atpif1
1E-05	0,427131	0,883	0,674	0,123966	5	Adrbk1
1,01E-05	0,507216	0,683	0,417	0,12482	5	Spcs1
1,02E-05	0,326581	0,433	0,198	0,126149	5	Rars
1,02E-05	-0,36332	1	0,999	0,126854	5	Rps24
1,04E-05	0,384137	0,517	0,269	0,128788	5	Mrpl33
1,05E-05	0,264588	0,317	0,121	0,129627	5	Dzip3
1,05E-05	0,401448	0,517	0,27	0,129834	5	Swi5
1,05E-05	-0,60507	0,117	0,396	0,13037	5	Chd7
1,06E-05	0,430044	0,667	0,381	0,130619	5	Pnp
1,06E-05	-0,75583	0,133	0,401	0,130678	5	Snn
1,06E-05	0,428717	0,767	0,517	0,131184	5	Tceb2
1,08E-05	0,486096	0,933	0,901	0,133958	5	Ywhaz
1,1E-05	-0,77108	0,2	0,491	0,136148	5	Dock10
1,11E-05	-0,76387	0,917	0,972	0,137282	5	Gm17821
1,14E-05	0,250833	0,233	0,077	0,140985	5	Lrwd1
1,18E-05	0,389244	0,45	0,218	0,145926	5	Anapc1
1,22E-05	0,469871	0,85	0,623	0,150494	5	Pold4
1,22E-05	0,415254	0,417	0,195	0,150704	5	Zadh2
1,23E-05	0,426591	0,583	0,332	0,152857	5	Ndufb3
1,25E-05	0,384649	0,883	0,727	0,155334	5	Sfpq
1,26E-05	0,32836	0,317	0,128	0,1561	5	Cenpc1
1,31E-05	0,388038	0,517	0,262	0,162743	5	Klf6
1,32E-05	0,466438	0,367	0,168	0,163198	5	Hmgn5
1,33E-05	0,372834	0,5	0,256	0,16414	5	Uchl3
1,34E-05	0,538598	0,45	0,228	0,166418	5	Atp6v1a
1,38E-05	0,464723	0,65	0,413	0,17122	5	Psmb3
1,4E-05	0,370422	0,333	0,142	0,173149	5	Slc41a2
1,42E-05	0,49792	0,367	0,166	0,175391	5	Ints9
1,47E-05	-0,61851	0,733	0,86	0,181523	5	D4Wsu53e
1,49E-05	0,384013	0,967	0,834	0,183983	5	Chchd2
1,56E-05	0,271034	0,317	0,124	0,193628	5	Hjurp
1,57E-05	0,526979	0,717	0,486	0,194014	5	Tmpo
1,62E-05	0,351237	0,767	0,479	0,2009	5	Minos1
1,63E-05	-0,83684	0,25	0,491	0,20174	5	Fam65b
1,64E-05	0,413076	0,5	0,266	0,202493	5	Psmc1
1,69E-05	0,441461	0,617	0,379	0,208862	5	eGFP

1,69E-05	0,288616	0,283	0,106	0,209174	5	5830416P10Rik
1,78E-05	0,264628	0,25	0,089	0,220977	5	Asrgl1
1,79E-05	0,41101	0,783	0,543	0,221146	5	Atp5j
1,79E-05	0,402605	0,8	0,521	0,221433	5	Ewsr1
1,79E-05	0,412439	0,95	0,784	0,221729	5	Slc25a5
1,84E-05	0,436419	0,867	0,67	0,227936	5	Purb
1,95E-05	0,400336	0,433	0,206	0,241527	5	Rrm1
1,99E-05	0,300071	0,35	0,148	0,246542	5	Ssna1
2E-05	0,453403	0,667	0,405	0,247809	5	Idh3g
2,02E-05	-0,66169	0,417	0,629	0,250288	5	Snx2
2,06E-05	0,406287	0,517	0,273	0,255198	5	Hmgn3
2,07E-05	0,425165	0,933	0,772	0,256671	5	Srsf2
2,14E-05	0,416838	0,783	0,545	0,264447	5	Snrpe
2,15E-05	0,29991	0,5	0,248	0,265683	5	Aurkaip1
2,33E-05	0,327791	0,483	0,246	0,288055	5	Mbtd1
2,34E-05	0,40426	0,467	0,239	0,290089	5	Mprip
2,37E-05	0,318708	0,25	0,089	0,293288	5	Hccs
2,48E-05	0,549294	0,667	0,43	0,307399	5	Psmd14
2,56E-05	0,48251	0,883	0,657	0,316509	5	Prdx1
2,62E-05	0,286574	0,417	0,195	0,323845	5	Ngdn
2,67E-05	0,347284	0,533	0,298	0,330392	5	Tceb1
2,74E-05	0,491755	0,767	0,505	0,338702	5	Cox5b
2,89E-05	-0,70789	0,283	0,521	0,357947	5	Itm2b
2,9E-05	0,443838	0,733	0,47	0,359223	5	Hpse
2,93E-05	0,384606	0,5	0,273	0,362147	5	Ahcy
2,93E-05	0,255952	0,133	0,033	0,3628	5	Anln
3,03E-05	0,32233	0,267	0,102	0,375055	5	Bpgm
3,24E-05	0,39545	0,4	0,191	0,401169	5	Rrm2b
3,36E-05	0,330606	0,483	0,253	0,416297	5	Ndufs8
3,43E-05	0,330019	0,55	0,293	0,424295	5	Cetn3
3,48E-05	0,321673	0,533	0,287	0,430868	5	Dgkz
3,49E-05	0,440293	0,683	0,435	0,432221	5	Cmpk1
3,51E-05	0,409311	0,75	0,502	0,433921	5	Erh
3,64E-05	0,401019	0,95	0,808	0,450329	5	Arpc5
3,64E-05	-0,3146	1	0,998	0,450791	5	Rps29
3,67E-05	0,298556	0,4	0,184	0,454399	5	Bfsp2
3,74E-05	0,289239	0,317	0,135	0,463475	5	Bak1
3,74E-05	0,364359	0,417	0,201	0,463593	5	Cyp51
3,76E-05	-0,76278	0,65	0,805	0,465072	5	Grk4
3,76E-05	-0,5262	0,117	0,375	0,465438	5	Mllt6
3,82E-05	0,350125	0,517	0,264	0,472988	5	Ppp1r2
3,87E-05	0,31481	0,517	0,265	0,478795	5	Lypla1
3,87E-05	-0,64941	0,283	0,542	0,479193	5	Gimap3
4,24E-05	-0,57124	0	0,223	0,524952	5	Arhgef18
4,32E-05	0,356312	1	0,981	0,534578	5	Laptm5
4,35E-05	0,307074	0,45	0,222	0,538016	5	Rbm7
4,42E-05	0,43866	0,55	0,323	0,547002	5	Sar1b
4,42E-05	-0,56078	0,2	0,471	0,547495	5	A130077B15Rik
4,44E-05	0,345443	0,617	0,349	0,549949	5	Pkig
4,56E-05	0,38119	0,817	0,563	0,565005	5	Hnrnpd

4,57E-05	-0,60219	0,6	0,751	0,566109	5	Ltb
4,59E-05	0,306649	0,483	0,251	0,568097	5	Exosc8
4,64E-05	-0,73201	0,317	0,554	0,574369	5	Cr2
4,66E-05	0,400524	0,883	0,654	0,576685	5	Cox6c
4,76E-05	0,435469	0,517	0,28	0,589129	5	Xrcc1
4,99E-05	0,285303	0,267	0,105	0,618224	5	Carhsp1
5E-05	0,323446	0,75	0,487	0,619189	5	Wdr92
5,07E-05	0,295153	0,45	0,225	0,62815	5	Magoh
5,13E-05	0,364759	0,467	0,244	0,63509	5	Psat1
5,33E-05	0,265933	0,5	0,256	0,659352	5	Gnai3
5,39E-05	0,330696	0,683	0,416	0,666979	5	Tcp1
5,42E-05	0,34427	0,45	0,237	0,671082	5	Eif1b
5,42E-05	0,38642	0,983	0,931	0,671352	5	H3f3b
6,05E-05	0,385569	0,6	0,343	0,749288	5	Cep110
6,12E-05	0,284681	0,3	0,126	0,758203	5	Tex30
6,27E-05	0,304587	0,15	0,042	0,77603	5	Ehf
6,28E-05	0,426161	0,833	0,621	0,777027	5	Atp5l
6,43E-05	0,255771	0,25	0,094	0,796555	5	Phf19
6,52E-05	0,327739	0,383	0,184	0,807271	5	Wsb2
6,88E-05	-0,55483	0,417	0,623	0,852296	5	H2-Oa
7,19E-05	0,30441	0,533	0,295	0,890202	5	Ddb1
7,29E-05	0,353966	0,867	0,676	0,902476	5	Cnp
7,4E-05	-0,60189	0,05	0,276	0,916057	5	Serpinb1a
7,49E-05	0,399992	0,75	0,543	0,927846	5	Psmb4
7,56E-05	0,468407	0,667	0,426	0,93574	5	Dynlrb1
7,62E-05	0,419983	0,8	0,555	0,942991	5	Psmb7
7,71E-05	0,439437	0,817	0,644	0,954496	5	Cox4i1
7,75E-05	-0,54047	0,317	0,538	0,959843	5	Wdfy4
7,84E-05	-0,67941	0,217	0,432	0,97108	5	Ncf1
7,86E-05	0,65942	0,833	0,69	0,973318	5	Cpne8
8,11E-05	0,327024	0,25	0,098	1	5	Nudcd2
8,16E-05	0,414229	0,683	0,435	1	5	G3bp1
8,17E-05	-0,48499	0	0,209	1	5	Ccr6
8,23E-05	-0,50232	0,417	0,689	1	5	Swap70
8,23E-05	0,251345	0,167	0,051	1	5	Spdl1
8,36E-05	0,348211	0,317	0,141	1	5	Nol8
8,54E-05	0,30134	0,45	0,232	1	5	Sptssa
8,56E-05	0,432609	0,55	0,33	1	5	Supt4a
8,6E-05	0,3258	0,533	0,307	1	5	Phf5a
8,64E-05	0,27772	0,6	0,332	1	5	Dnajb6
8,76E-05	-0,52087	0,033	0,254	1	5	Irf1
8,99E-05	0,329582	0,367	0,171	1	5	Immp1l
9,05E-05	-0,55053	0,183	0,426	1	5	Mll3
9,19E-05	0,259499	0,25	0,098	1	5	9430023L20Rik
9,29E-05	0,431613	0,683	0,455	1	5	Cox17
9,29E-05	-0,66623	0,45	0,645	1	5	B4galnt1
9,98E-05	-0,52816	0,05	0,271	1	5	Itgb7
0,0001	0,347302	0,9	0,739	1	5	Hnrnpf
0,0001	0,266189	0,183	0,06	1	5	Hmga2-ps1
0,000101	0,379574	0,517	0,292	1	5	Cyb5r4

0,000102	-0,52212	0,067	0,296	1	5	Cd84
0,000102	-0,53986	0,283	0,509	1	5	Vasp
0,000102	-0,67429	0,517	0,711	1	5	Erdr1
0,000102	0,369197	0,333	0,153	1	5	C1d
0,000103	0,353035	0,483	0,262	1	5	Sun2
0,000105	-0,53568	0,05	0,275	1	5	Fgd2
0,00011	-0,45311	0,033	0,254	1	5	Hhex
0,000111	0,34304	0,567	0,342	1	5	Dars
0,000112	-0,58393	0,033	0,248	1	5	Cd55
0,000117	0,385205	0,8	0,61	1	5	Rap1a
0,000118	0,334334	0,333	0,157	1	5	Txndc11
0,000128	0,42776	0,817	0,544	1	5	Rac1
0,000132	-0,3309	1	0,999	1	5	Rplp1
0,000139	0,282193	0,333	0,15	1	5	Gtf2h1
0,000139	0,369329	0,6	0,374	1	5	Psmc2
0,000142	0,270917	0,333	0,148	1	5	Hiat1
0,000143	0,289486	0,983	0,956	1	5	Myl6
0,000146	0,645542	0,95	0,761	1	5	Tuba1b
0,000148	0,324367	1	0,983	1	5	Arpc2
0,00015	0,395577	0,983	0,988	1	5	Lcp1
0,000154	0,285971	0,583	0,34	1	5	Psmb6
0,000158	0,39972	0,667	0,458	1	5	Trmt112
0,000159	0,47031	0,433	0,248	1	5	Ogfrl1
0,00016	0,288958	0,383	0,188	1	5	Cnot6
0,000165	0,296954	0,783	0,513	1	5	Nedd8
0,000166	0,496025	0,633	0,443	1	5	Yeats4
0,000166	-0,49224	0,017	0,219	1	5	Lgals9
0,000167	0,389355	0,35	0,171	1	5	Ngfrap1
0,000167	0,468538	0,783	0,572	1	5	Klhl6
0,000168	0,323446	0,667	0,436	1	5	Atp5o
0,000168	0,350125	0,483	0,277	1	5	Esd
0,00017	0,345794	0,483	0,267	1	5	Slc30a5
0,000175	-0,55625	0,033	0,239	1	5	Zfp318
0,000175	0,353658	0,9	0,78	1	5	Cox8a
0,000178	-0,5633	0,233	0,471	1	5	Pld4
0,000179	-0,41321	0,983	0,987	1	5	Ptprc
0,000181	0,339467	0,517	0,307	1	5	Kars
0,000182	0,318219	0,3	0,135	1	5	4933421O10Rik
0,000185	0,337275	0,583	0,354	1	5	Ppp2r5c
0,000187	-0,29453	1	1	1	5	Rpl5
0,000189	0,306043	0,467	0,252	1	5	Ube2m
0,00019	-0,45673	0,7	0,858	1	5	Eif4a2
0,000192	-0,48694	0,1	0,324	1	5	Usp34
0,000194	0,334171	0,75	0,543	1	5	Eif3k
0,000195	0,379708	0,55	0,321	1	5	Rnf19b
0,0002	0,265446	0,467	0,257	1	5	Pcf11
0,000202	0,405276	0,8	0,555	1	5	Tra2b
0,000206	0,438138	0,7	0,483	1	5	Arpc3
0,000206	-0,47554	0,083	0,298	1	5	Jhdm1d
0,000207	0,30442	0,583	0,35	1	5	Uqcrc1

0,00021	-0,36794	1	0,991	1	5	Rpl11
0,000211	0,363357	0,333	0,16	1	5	Dhx40
0,000212	0,330808	0,683	0,441	1	5	Cerk
0,000212	0,35931	0,367	0,185	1	5	Fam20b
0,000216	-0,46934	0,55	0,724	1	5	Gm9846
0,000217	0,318797	0,483	0,278	1	5	Ap1s3
0,000221	0,300148	0,633	0,394	1	5	Ap2s1
0,000222	0,298693	0,833	0,597	1	5	Gna13
0,000222	0,285534	0,7	0,425	1	5	Atp5k
0,000224	-0,51747	0,117	0,341	1	5	Rapgef4
0,000226	0,367765	0,567	0,362	1	5	Rab1
0,000226	0,338084	0,567	0,339	1	5	Rnf114
0,000234	0,262822	0,5	0,269	1	5	Atp6v1d
0,000237	0,454675	0,75	0,553	1	5	Vcp
0,000237	-0,52386	0,067	0,273	1	5	Man1a
0,000243	-0,29776	1	1	1	5	Cd79a
0,000244	0,346191	0,7	0,476	1	5	Psma2
0,000247	-0,69604	0,467	0,615	1	5	Mll5
0,000247	-0,3921	0	0,186	1	5	Il10rb
0,000262	0,347706	0,417	0,218	1	5	Ube2v2
0,000262	-0,64766	0,217	0,414	1	5	Anxa6
0,000264	0,315507	0,35	0,169	1	5	Zfp120
0,000265	-0,47901	0,217	0,461	1	5	Psap
0,000266	-0,52437	0,117	0,335	1	5	Ier5
0,000266	0,350518	0,45	0,241	1	5	Ifi27l2a
0,000273	-0,51618	0,45	0,659	1	5	Phip
0,000275	0,382447	0,483	0,284	1	5	Anapc11
0,00028	0,363694	0,467	0,274	1	5	Ilk
0,000283	0,253839	0,533	0,303	1	5	Ibtk
0,000284	0,285269	0,183	0,065	1	5	Spata24
0,000286	0,259728	0,333	0,16	1	5	Polr3k
0,000301	-0,56856	0,317	0,499	1	5	Srpk3
0,000302	0,262721	0,633	0,385	1	5	Bzw1
0,000308	-0,56867	0,05	0,248	1	5	Gpr183
0,000309	0,315502	0,567	0,34	1	5	Psmb5
0,000312	0,307472	0,35	0,177	1	5	Ndufa5
0,000313	0,295179	0,7	0,437	1	5	Akt1
0,000315	0,365266	0,833	0,658	1	5	Ppp1ca
0,000318	0,343852	0,25	0,105	1	5	Stil
0,000318	-0,31299	0,967	0,997	1	5	Rps28
0,00032	0,292924	0,283	0,126	1	5	H1f0
0,000328	0,321449	0,367	0,189	1	5	Grpel2
0,00033	0,315558	0,517	0,298	1	5	Selk
0,000333	0,294451	0,483	0,268	1	5	Aldh9a1
0,000336	-0,63158	0,75	0,856	1	5	Apoe
0,00034	-0,60726	0,317	0,508	1	5	Gimap8
0,000344	-0,47068	0,233	0,457	1	5	Dennd5b
0,000345	0,463249	0,683	0,509	1	5	Prrc2a
0,000348	-0,47848	0,167	0,383	1	5	Sorl1
0,000349	0,268608	0,55	0,329	1	5	Ndufv2

0,000351	0,307802	0,483	0,286	1	5	Znrd1
0,000357	0,301662	0,4	0,216	1	5	Vdac1
0,000365	0,412133	0,3	0,146	1	5	Prep
0,000367	0,352388	0,467	0,275	1	5	Psmc3
0,00037	0,274213	0,733	0,509	1	5	Eif4h
0,000372	-0,44773	0,117	0,328	1	5	Tspan32
0,000373	0,256398	0,383	0,191	1	5	Isca1
0,000383	0,25645	0,217	0,087	1	5	Sqle
0,000384	-0,46305	0,083	0,29	1	5	Esyt1
0,000393	0,287513	0,5	0,284	1	5	Eif2ak3
0,000398	0,261185	0,35	0,174	1	5	Nfyb
0,0004	0,315391	0,733	0,508	1	5	Cct5
0,000406	0,291876	0,983	0,929	1	5	Psmb8
0,00041	0,420027	0,55	0,353	1	5	Arl5a
0,000414	0,396499	0,867	0,655	1	5	Eif3h
0,000416	-0,33576	1	0,999	1	5	Rpl12
0,000416	-0,51004	0,767	0,814	1	5	Btg1
0,000423	0,419821	0,6	0,4	1	5	Zfp106
0,000426	0,472035	0,567	0,354	1	5	Anapc16
0,00044	-0,44349	1	0,98	1	5	H2-K1
0,000441	0,346191	0,7	0,491	1	5	Atp5f1
0,000452	-0,46747	0,15	0,361	1	5	Myl12b
0,000457	0,413055	0,383	0,207	1	5	Trp53inp1
0,000459	0,273646	0,667	0,425	1	5	Xpo1
0,000466	0,350002	0,867	0,664	1	5	Cox6a1
0,000477	0,279521	0,483	0,28	1	5	Pak2
0,000481	0,322053	0,5	0,302	1	5	Bnip2
0,00049	0,301527	0,483	0,269	1	5	Mrps14
0,000497	0,316206	0,433	0,237	1	5	Gtf2a1
0,000504	-0,4848	0,4	0,574	1	5	Arhgap4
0,000513	0,253057	0,65	0,4	1	5	Dcaf12
0,000527	-0,48215	0,117	0,319	1	5	Lrrc33
0,000542	0,253181	0,317	0,154	1	5	Fxr1
0,000562	-0,59559	0,017	0,192	1	5	Plac8
0,000564	0,311966	0,217	0,089	1	5	2510003E04Rik
0,000566	-0,37575	0,017	0,193	1	5	Cyfip1
0,000566	-0,45579	0,083	0,287	1	5	Chd2
0,000576	0,483647	0,617	0,444	1	5	Dync1i2
0,000582	-0,55447	0,367	0,568	1	5	Slc38a2
0,000606	0,369812	0,467	0,267	1	5	Arcn1
0,000611	0,339488	0,333	0,171	1	5	Nt5c3
0,000617	-0,42767	0,283	0,513	1	5	Ralgps2
0,00062	0,265789	0,85	0,58	1	5	Dnaja1
0,000627	-0,47346	0,167	0,376	1	5	Cyb561a3
0,000631	-0,3218	0,983	0,988	1	5	Rpl35
0,000633	-0,3746	0,017	0,19	1	5	Abcg1
0,000637	0,309388	0,7	0,464	1	5	Rbx1
0,000637	-0,31368	0,983	0,997	1	5	Rps11
0,000639	0,295476	0,217	0,092	1	5	Flt3
0,000661	0,289057	0,5	0,3	1	5	Adss

0,000682	0,368977	0,417	0,228	1	5	Nt5e
0,000704	0,288463	0,8	0,566	1	5	Cox7b
0,000712	0,28068	0,75	0,533	1	5	Vdac2
0,000713	0,3247	0,467	0,274	1	5	Mdm2
0,000715	0,31959	0,8	0,59	1	5	Lrmp
0,000725	0,37933	0,217	0,094	1	5	Sephs1
0,00075	-0,5653	0,35	0,563	1	5	Gm10785
0,000753	-0,59132	0,433	0,586	1	5	Gimap6
0,000756	0,295797	0,45	0,261	1	5	Eif4e
0,000776	-0,45471	0,767	0,866	1	5	Tnfrsf13c
0,000781	0,400309	0,817	0,636	1	5	Sept6
0,000789	0,258029	0,567	0,347	1	5	Vmp1
0,00079	0,33898	0,6	0,4	1	5	Glud1
0,000791	-0,47634	0,183	0,395	1	5	Stk10
0,000793	0,254077	0,567	0,349	1	5	Snrpd1
0,000794	0,274234	0,567	0,343	1	5	Psma5
0,000794	0,296579	0,233	0,101	1	5	Pspc1
0,000795	-0,3787	0,067	0,257	1	5	2900060B14Rik
0,000805	-0,4222	0,067	0,255	1	5	Cdkn1b
0,000818	-0,49567	0,267	0,451	1	5	Tgfbr2
0,000832	-0,52885	0,183	0,372	1	5	Cytip
0,00084	0,324286	0,517	0,324	1	5	Brd8
0,000866	0,305809	0,933	0,844	1	5	Gdi2
0,00088	-0,29445	1	0,999	1	5	Rps23
0,000909	0,367501	0,667	0,47	1	5	Gnb1
0,000939	0,393911	0,75	0,567	1	5	Polr1d
0,000954	0,301592	0,65	0,434	1	5	Ndufb11
0,000955	0,403935	0,483	0,314	1	5	Tfam
0,000963	-0,39016	0,1	0,297	1	5	Mapk14
0,000979	-0,707	0,35	0,498	1	5	Itga4
0,000996	0,274513	0,233	0,101	1	5	2410004B18Rik
0,001011	-0,36687	0,067	0,256	1	5	Aff3
0,001011	0,300547	0,283	0,137	1	5	Lmo7
0,001025	0,351789	0,767	0,534	1	5	Sec11c
0,001027	0,411779	0,617	0,427	1	5	Rps6kb1
0,001031	0,28651	0,433	0,255	1	5	Trappc1
0,001036	0,2731	0,483	0,276	1	5	Mtmr14
0,001038	0,253728	0,433	0,241	1	5	Sav1
0,001047	0,290139	0,533	0,324	1	5	Lpin2
0,001056	-0,30297	1	0,994	1	5	Rps18
0,001062	0,25355	0,35	0,185	1	5	Mrpl20
0,001063	0,268497	0,45	0,266	1	5	Alkbh5
0,001063	0,265006	0,7	0,462	1	5	Cox5a
0,001096	0,667716	0,35	0,202	1	5	Eif5a2
0,001099	-0,29128	1	0,998	1	5	Uba52
0,001113	0,404314	0,517	0,343	1	5	Trim35
0,001121	-0,32949	0	0,152	1	5	Cnn3
0,001134	0,291618	0,633	0,431	1	5	Ube2v1
0,001142	-0,56489	0,267	0,462	1	5	Filip1l
0,001179	0,293389	0,3	0,154	1	5	l7Rn6

0,001179	0,26799	0,333	0,174	1	5	Gpd1l
0,001187	-0,63019	0,217	0,397	1	5	Dgka
0,00119	0,25184	0,417	0,233	1	5	Otud1
0,001198	0,315961	0,667	0,452	1	5	Atp5d
0,001204	0,385623	0,767	0,655	1	5	Banf1
0,001215	0,302552	0,7	0,481	1	5	Sec61b
0,001218	0,284008	0,517	0,324	1	5	Usp19
0,001232	0,255859	0,367	0,192	1	5	Wbp5
0,001234	-0,47081	0,717	0,803	1	5	Ly86
0,001251	0,253239	0,55	0,343	1	5	Zfp710
0,001263	0,425311	0,517	0,325	1	5	BC031181
0,001274	0,272165	0,25	0,118	1	5	Cnep1r1
0,001291	-0,3281	0,167	0,372	1	5	Psen2
0,001293	0,272165	0,583	0,38	1	5	Ndufa1
0,001317	-0,34395	0,067	0,242	1	5	Cdk17
0,001325	-0,35606	0,017	0,174	1	5	Cd97
0,001333	0,272694	0,467	0,267	1	5	D15Ertd621e
0,001336	0,35426	0,367	0,209	1	5	Cops5
0,001338	0,283398	0,967	0,772	1	5	Tma7
0,001353	0,280764	0,583	0,375	1	5	Snrpf
0,001372	-0,28971	0	0,147	1	5	Pxdc1
0,001414	0,255092	0,367	0,199	1	5	Ggta1
0,001421	0,273904	0,3	0,15	1	5	Cited2
0,001438	0,290979	0,517	0,308	1	5	Krcc1
0,001465	-0,32171	1	0,999	1	5	Ly6e
0,001466	0,308607	0,217	0,095	1	5	Taf13
0,00148	0,289556	0,35	0,19	1	5	Uchl5
0,001487	0,269292	0,283	0,139	1	5	Asun
0,001504	-0,47443	0,467	0,66	1	5	H2-T23
0,001511	-0,32728	0,2	0,413	1	5	Lyl1
0,001514	0,351516	0,917	0,854	1	5	Eif5a
0,001522	0,289115	0,467	0,284	1	5	Atp6v0d1
0,001536	0,322091	0,383	0,223	1	5	Cstf3
0,001538	0,297458	0,4	0,228	1	5	Cetn2
0,001541	0,320405	0,45	0,272	1	5	Cebpg
0,00156	0,34973	0,583	0,422	1	5	Tra2a
0,001561	0,341466	0,417	0,257	1	5	Snx1
0,001615	0,307965	0,883	0,709	1	5	Ube2d2a
0,001646	-0,34237	0,05	0,216	1	5	Tle3
0,001653	-0,46776	0,217	0,396	1	5	Il4ra
0,001693	0,336908	0,667	0,501	1	5	Top2b
0,001735	-0,4508	0,483	0,652	1	5	Gdi1
0,001742	0,278318	0,383	0,218	1	5	Mrps36
0,001742	0,392117	0,883	0,779	1	5	Atp5g2
0,001763	0,258236	0,517	0,323	1	5	Cat
0,001765	-0,38599	0,067	0,243	1	5	Zscan26
0,001776	0,323568	0,9	0,698	1	5	Basp1
0,001809	0,293607	0,417	0,246	1	5	Mbd1
0,001831	0,272551	0,5	0,306	1	5	Eif1ax
0,001834	0,250205	0,4	0,231	1	5	Rap2c

0,00184	0,289304	0,367	0,209	1	5	Pim1
0,001845	0,264051	0,467	0,281	1	5	Cnih
0,001847	0,358026	0,417	0,262	1	5	Ruvbl2
0,001866	0,257613	0,383	0,218	1	5	Rtfdc1
0,001874	0,282053	0,417	0,247	1	5	Hadha
0,001878	-0,28987	0,017	0,167	1	5	9930111J21Rik1
0,001884	0,262103	0,233	0,11	1	5	Rpp14
0,001895	0,387541	0,917	0,842	1	5	Ybx1
0,001909	0,324666	0,433	0,276	1	5	Cstb
0,001935	-0,31766	0,05	0,212	1	5	C1qbp
0,001958	-0,3986	0,083	0,256	1	5	Plekhm3
0,001977	-0,27223	0	0,139	1	5	Fxyd5
0,002002	0,302935	0,433	0,261	1	5	Trpc4ap
0,002008	0,303631	0,367	0,21	1	5	Mid1ip1
0,002012	-0,32239	0,1	0,284	1	5	Dennd4b
0,002044	0,26813	0,45	0,278	1	5	Babam1
0,002058	-0,34822	0,217	0,411	1	5	Zcchc7
0,002061	-0,33552	0,05	0,211	1	5	Phf1
0,002102	-0,33988	0,083	0,257	1	5	Cyth4
0,002116	-0,51091	0,617	0,697	1	5	Chst3
0,002187	0,274103	0,75	0,543	1	5	Cct4
0,002242	-0,35218	0,467	0,675	1	5	Dock8
0,002283	0,29779	0,367	0,21	1	5	Atp6ap2
0,002295	0,350897	0,45	0,28	1	5	Chordc1
0,002304	-0,35158	0,117	0,289	1	5	Gripap1
0,002309	0,303656	0,783	0,572	1	5	Prr13
0,002326	0,328491	0,367	0,214	1	5	Lgmn
0,002331	-0,43899	0,233	0,413	1	5	Rapgef6
0,002333	-0,46098	0,317	0,512	1	5	Gpr18
0,002393	-0,69245	0,533	0,656	1	5	Vmn2r55
0,002421	-0,25708	0	0,134	1	5	Gabbr1
0,002446	0,286669	0,767	0,569	1	5	Akr1a1
0,00245	0,270276	0,5	0,322	1	5	Psmc6
0,002466	0,294617	0,433	0,261	1	5	Psmd13
0,002531	0,289127	0,283	0,154	1	5	Crcp
0,002617	0,265032	0,783	0,557	1	5	Scaf11
0,002622	-0,32185	0,017	0,159	1	5	Ddx58
0,002667	0,285399	0,367	0,215	1	5	Sra1
0,002677	0,296995	0,35	0,202	1	5	Atp6v1h
0,002736	0,274536	0,267	0,137	1	5	Lias
0,002752	0,266486	0,4	0,238	1	5	Nop58
0,002764	-0,3556	0,067	0,232	1	5	Neat1
0,00284	-0,35839	0,017	0,157	1	5	Cd38
0,002841	0,252399	0,8	0,606	1	5	Sod1
0,002873	0,274785	0,817	0,62	1	5	Cox6b1
0,002948	0,375296	0,517	0,372	1	5	Ube2n
0,002962	-0,37072	0,25	0,444	1	5	Ogt
0,003005	-0,41867	0,183	0,36	1	5	Ankrd44
0,003022	-0,40376	0,05	0,205	1	5	Scml4
0,003046	0,269951	0,65	0,433	1	5	Ccm2

0,003096	0,305524	0,75	0,55	1	5	Papola
0,003096	-0,29	0,05	0,211	1	5	Sbk1
0,003099	-0,26657	0	0,128	1	5	Ski
0,00313	0,438729	0,25	0,127	1	5	Ccdc88a
0,00317	0,274712	0,5	0,318	1	5	Hnrnph2
0,003196	0,29392	0,517	0,349	1	5	Chmp2a
0,003225	0,287671	0,367	0,214	1	5	Smim15
0,003228	0,277978	0,317	0,168	1	5	Uhrf2
0,003244	0,334478	0,533	0,342	1	5	Rps27l
0,003275	0,267147	0,217	0,104	1	5	Slc41a1
0,003281	-0,31767	0,017	0,154	1	5	Ckap4
0,003322	-0,41162	0,1	0,258	1	5	Mdn1
0,00334	0,279819	0,45	0,279	1	5	Pias1
0,003375	-0,4711	0,683	0,77	1	5	Prkcb
0,003411	0,297023	0,567	0,39	1	5	Map1lc3b
0,003414	0,262384	0,267	0,138	1	5	Pfdn4
0,003461	-0,44178	0,333	0,493	1	5	Sh3bp5
0,003596	-0,57926	0,267	0,414	1	5	Add3
0,003605	0,251032	0,333	0,19	1	5	Atp6v1e1
0,003678	0,301552	0,483	0,329	1	5	Srp9
0,003726	0,286281	0,983	0,992	1	5	Srgn
0,00376	0,307802	0,383	0,239	1	5	Usp14
0,003816	-0,45435	0,233	0,391	1	5	Slc7a1
0,003822	-0,40785	0,667	0,75	1	5	Hmha1
0,00384	0,290701	0,833	0,671	1	5	Prkar1a
0,003864	-0,37998	0,6	0,693	1	5	Aldoa
0,00398	-0,26959	0,017	0,149	1	5	Setd1b
0,004018	-0,3066	0,05	0,196	1	5	Sema4b
0,004057	-0,33732	0	0,122	1	5	Gbp7
0,004114	0,257698	0,45	0,279	1	5	Gorasp2
0,00414	-0,2672	0	0,121	1	5	Tmem154
0,004143	-0,41276	0,15	0,312	1	5	Zmiz1
0,004148	0,303485	0,383	0,238	1	5	Dpm2
0,004179	0,254508	0,8	0,622	1	5	Snrpg
0,004402	-0,43859	0,45	0,602	1	5	Ncor1
0,004406	0,257927	0,667	0,501	1	5	Rabgap1l
0,004412	-0,3315	0,05	0,193	1	5	Kynu
0,004419	0,250347	0,6	0,412	1	5	Psmd8
0,00445	0,393275	0,4	0,249	1	5	Tmem184c
0,004507	0,259949	0,533	0,363	1	5	Rad23b
0,004508	-0,34193	0,183	0,352	1	5	Itpr3
0,004518	0,407554	0,65	0,466	1	5	Elf1
0,004521	0,29638	0,483	0,329	1	5	Psmd4
0,004524	-0,40297	0,25	0,423	1	5	Ash1l
0,004553	-0,39503	0,6	0,734	1	5	Ptk2b
0,004578	0,28727	0,8	0,617	1	5	Stk4
0,004584	0,32624	0,383	0,234	1	5	Klhl24
0,004621	-0,41486	0,267	0,452	1	5	Nipbl
0,004664	0,281888	0,367	0,218	1	5	Sf3a1
0,004666	-0,32742	0,1	0,258	1	5	Slc25a37

0,004671	0,349093	0,467	0,317	1	5	Sdcbp
0,004689	0,289263	0,6	0,448	1	5	Fkbp1a
0,004701	0,314575	0,35	0,21	1	5	Bag1
0,00472	-0,25454	0	0,118	1	5	Adamts10
0,004812	-0,2782	0,217	0,405	1	5	Cdk13
0,004929	0,26266	0,25	0,131	1	5	Prps1
0,005027	-0,43387	0,2	0,353	1	5	Nxpe3
0,005093	-0,37219	0,1	0,253	1	5	Itpr1
0,005265	0,306785	0,633	0,445	1	5	Psip1
0,005299	-0,29535	0,017	0,142	1	5	Nfic
0,005311	0,289271	0,417	0,266	1	5	Syf2
0,005391	-0,32214	0,067	0,213	1	5	Tcp11l2
0,005394	0,29514	0,233	0,12	1	5	Nup93
0,005443	0,257996	0,5	0,336	1	5	Smarce1
0,005465	-0,32006	0,85	0,897	1	5	H2-DMa
0,005473	-0,39705	0,133	0,292	1	5	Rfx5
0,005506	0,275155	0,333	0,19	1	5	Mtmr3
0,005541	-0,38747	0,583	0,777	1	5	Jak1
0,00603	0,315034	0,433	0,283	1	5	Cdc34
0,006089	-0,27268	0,017	0,139	1	5	Ccr7
0,006104	-0,3031	0,05	0,186	1	5	Slfn2
0,00611	-0,43396	0,467	0,603	1	5	Atrx
0,006166	0,255976	0,567	0,391	1	5	0610009D07Rik
0,006181	0,269495	0,883	0,711	1	5	Hnrnpab
0,006236	-0,29088	0,067	0,207	1	5	Bcor
0,006474	0,350744	0,367	0,234	1	5	Slamf7
0,006572	0,351037	0,467	0,308	1	5	Cdc25b
0,006587	0,289413	0,517	0,364	1	5	Eif3d
0,006639	-0,39379	0,2	0,355	1	5	Klf13
0,006674	-0,38296	0,267	0,428	1	5	Nfkb2
0,006748	0,291578	0,517	0,371	1	5	Ktn1
0,00684	0,363657	0,6	0,46	1	5	Ptbp1
0,006869	0,315541	0,267	0,148	1	5	Mpp1
0,006906	-0,35885	0,15	0,296	1	5	Mga
0,006979	0,418063	0,4	0,251	1	5	Nrgn
0,007018	-0,30737	0,05	0,193	1	5	Kctd12
0,007079	-0,32327	0,1	0,251	1	5	Iqsec1
0,007154	-0,36627	0,333	0,476	1	5	Uvrag
0,007199	0,251836	0,733	0,526	1	5	Sqstm1
0,007268	0,320573	0,35	0,215	1	5	Setd8
0,007286	-0,2534	0,017	0,135	1	5	Prkce
0,007362	-0,33144	0,033	0,159	1	5	Kbtbd11
0,007397	0,304094	0,483	0,34	1	5	Lsm6
0,007398	0,303039	0,533	0,372	1	5	Tmed2
0,007618	-0,26763	0,033	0,159	1	5	Qdpr
0,007621	0,293334	0,617	0,454	1	5	Il17ra
0,007621	0,296152	0,883	0,788	1	5	Trp53i11
0,007642	-0,26641	0,033	0,158	1	5	Arhgef3
0,007792	0,266431	0,517	0,36	1	5	Mrps21
0,0078	-0,25729	0,05	0,181	1	5	Rab24

0,007859	-0,44181	0,433	0,559	1	5	Samd9l
0,00794	0,259438	0,883	0,782	1	5	Pkm
0,007947	0,320333	0,583	0,44	1	5	Sept7
0,008046	-0,40735	0,4	0,532	1	5	Cxcr5
0,008089	0,271174	0,4	0,258	1	5	Psmc4
0,008112	0,321005	0,367	0,236	1	5	Nup88
0,008162	-0,30199	0,433	0,586	1	5	Mif4gd
0,008292	-0,38637	0,5	0,618	1	5	Gimap1
0,008309	0,257848	0,633	0,449	1	5	2810407C02Rik
0,008375	0,314227	0,45	0,303	1	5	Ttc9c
0,00856	0,264209	0,6	0,451	1	5	Atp5j2
0,00857	-0,28625	0,133	0,283	1	5	H13
0,008653	0,253659	0,3	0,177	1	5	Eaf1
0,008724	-0,36455	0,4	0,528	1	5	Ivns1abp
0,008855	-0,36579	0,417	0,584	1	5	Rbm25
0,008938	0,275749	0,567	0,41	1	5	Ppp6r1
0,008951	-0,33256	0,017	0,13	1	5	Serpina3g
0,009048	0,2521	0,45	0,287	1	5	Adnp
0,009118	-0,2698	0,067	0,199	1	5	Ric8
0,00912	-0,34932	0,5	0,64	1	5	Fam49b
0,009122	-0,29664	0,05	0,177	1	5	2310061I04Rik
0,009188	0,276753	0,483	0,338	1	5	Bcl7a
0,009235	-0,25778	0,017	0,129	1	5	Plbd1
0,009285	-0,25708	0	0,102	1	5	Ffar2
0,009364	0,304733	0,567	0,425	1	5	Psma4
0,009375	0,270994	0,6	0,451	1	5	Coro1b
0,009413	-0,54141	0,4	0,525	1	5	Foxp1
0,009587	-0,42631	0,483	0,598	1	5	Zbtb20
0,009717	-0,43556	0,383	0,519	1	5	Akap8
0,009781	0,297701	0,333	0,209	1	5	Cript
0,009814	-0,30563	0,317	0,484	1	5	Lyn
0,009815	-0,32454	0,983	0,99	1	5	Cd53
0,00984	-0,28594	0,15	0,305	1	5	Pbxip1
0,009969	0,316459	0,4	0,268	1	5	Mrpl51
